# *De novo* design and synthesis of dipyridopurinone derivatives as visible-light photocatalysts in productive guanylation reactions†

**DOI:** 10.1039/d1sc05294b

**Published:** 2021-11-13

**Authors:** Yameng Wan, Hao Wu, Nana Ma, Jie Zhao, Zhiguo Zhang, Wenjing Gao, Guisheng Zhang

**Affiliations:** Key Laboratory of Green Chemical Media and Reactions, Ministry of Education, Collaborative Innovation Center of Henan Province for Green Manufacturing of Fine Chemicals, Henan Key Laboratory of Organic Functional Molecules and Drug Innovation, NMPA Key Laboratory for Research and Evaluation of Innovative Drug, School of Chemistry and Chemical Engineering, Henan Normal University 46 East of Construction Road Xinxiang Henan 453007 China zhangzg@htu.edu.cn zgs@htu.cn

## Abstract

Described here is the *de novo* design and synthesis of a series of 6*H*-dipyrido[1,2-*e*:2′,1′-*i*]purin-6-ones (DPs) as a new class of visible-light photoredox catalysts (PCs). The synthesized DP1–5 showed their *λ*_Abs(max)_ values in 433–477 nm, excited state redox potentials 
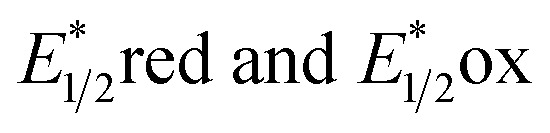
 in 1.15–0.69 eV and −1.41 to −1.77 eV (*vs.* SCE), respectively. As a representative, DP4 enables the productive guanylation of various amines, including 1°, 2°, and 3°-alkyl primary amines, secondary amines, aryl and heteroaryl amines, amino-nitrile, amino acids and peptides as well as propynylamines and α-amino esters giving diversities in biologically important guanidines and cyclic guanidines. The photocatalytic efficacy of DP4 in the guanylation overmatched commonly used Ir and Ru polypyridyl complexes, and some organic PCs. Other salient merits of this method include broad substrate scope and functional group tolerance, gram-scale synthesis, and versatile late-stage derivatizations that led to a derivative 81 exhibiting 60-fold better anticancer activity against Ramos cells with the IC_50_ of 0.086 μM than that of clinical drug ibrutinib (5.1 μM).

## Abstract 2

A novel visible-light photocatalyst was designed and its photocatalytic efficacy in the guanylation of amines overmatched common metal-core and organic photocatalysts.

## Graphical abstract

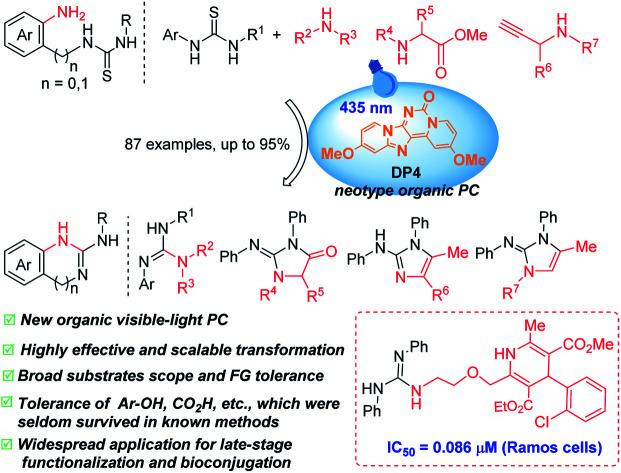

## Introduction

**Fig. 1:**
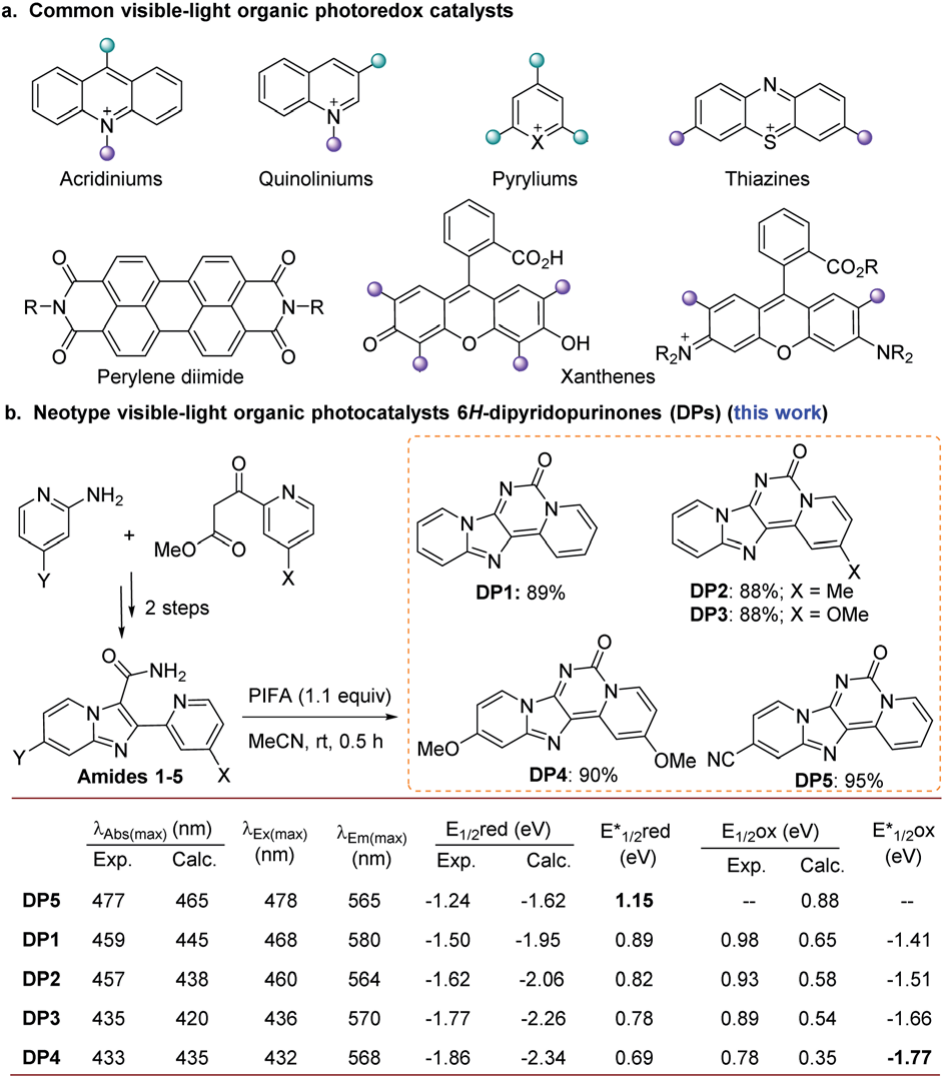
(a) The common organic PCs. (b) The synthesis and overview of the DP-based PCs and comparison of their calculated *vs.* experimental properties. All potential values are reported *vs.* SCE (Saturated Calomel Electrode). Calc. = calculated values. Exp. = experimental values.

In contrast to traditional chemical strategies involving single-electron transfer (SET) processes that require radical initiators and stoichiometric amounts of either strong reductants or oxidants, photocatalysis utilizes visible light as a clean and naturally abundant energy source. In the last decade, visible-light photoredox catalysis has emerged as a powerful strategy in organic synthesis and gained remarkable achievements orchestrating challenging organic transformations under mild reaction conditions.^1^ Ir and Ru polypyridyl complexes stand at the forefront of this class, offering long-lived exciton lifetimes and excellent redox potentials in their excited states.^1*e*–*g*,1*o*–*s*^ However, precious metals such as iridium and ruthenium are amongst the rarest elements on earth, and the main drawbacks associated with their applications are their escalating costs and scarce availability.^1*h*^ These drawbacks drive the need to realize new PCs incorporating non-precious metals^2^ or to develop entirely organic PCs including microporous polymers^3^ and small organic molecules,^1*h*^ providing valuable and inexpensive alternatives to transition metals. Recently, many researches^4^ have demonstrated that visible-light organic PCs offer far more than “metal-free” alternatives to transition metal examples; namely, the potent reactivity of many organic PCs allows access to unique chemistries and a wide range of unreactive substrates in most synthetic contexts.^1*h*^ The known common visible-light organic PCs include pyryliums, thiazines, perylene diimides, acridiniums, and xanthenes (Fig. 1a).^1*h*^ However, the choice of a suitable organic PC for general reactions is still rather limited due to the relatively few catalyst options.^5^

Very recently, we reported a promising lead fluorophore for developing new PCs and disclosed that *N*^5^-methylated pyridoquinazolinone could be served as a PC in several organic transformations.^6^ However, this kind of catalyst was used with UV irradiation (350 nm cobalt lamps). Thus, we became interested in exploring the photocatalytic features of a series of 6*H*-dipyrido[1,2-*e*:2′,1′-*i*]purin-6-one derivatives (DPs) modified with different electron-withdrawing (EWGs) and electron-donating groups (EDGs) (Fig. 1b and S2 in ESI†). We hypothesized that the skeleton of DPs containing dentate N atoms and rigid moiety with N-embedded extended conjugated π-systems might be a promising privileged backbone for potential visible-light organic PCs. In order to quickly evaluate the possibility of such compounds as PCs, we speculated that theoretical computations studies might represent a helpful tool to predict their properties *a priori*. Guided by the density-functional theory (DFT) calculations for our organic PC design, herein, DPs are reported as a new class of visible-light organic PCs (Fig. 1b). As a representative of DPs, DP4 served as a highly effective visible-light PC in the guanylation of various thioureas with an extremely broad range of amines including 1°, 2°, and 3°-alkyl primary amines, secondary amines, aryl (heteroaryl) amines, amino acids and peptides as well as amino-nitrile for the synthesis of structurally diverse guanidines and cyclic guanidines including 2-aminoimidazolese, 2-aminobenzoimidazolese, 2-amino-1,4-dihydro- quinazolines, 2-imino-1,3-dihydroimidazoles, and 2-iminoimidazolidin-4-ones. This preliminary application of DP4 validated our initial hypothesis and predicted a bright future for DPs as a new class of visible organic PCs to realize various organic transformations.

## Results and discussion

### Photocatalyst development

Through computational simulations by the DFT method (computational details see Section 3 in ESI†), the absorption maximum together with their redox potentials of fifty designed compounds was calculated (see Table S1 in ESI† for details). Gratifyingly, the calculated values of maximum absorption of all studied compounds were greater than 400 nm with significant tailing towards the blue light region, thus supporting our initial assumption that such compounds might be valuable in visible-light-driven catalysis reaction. Based on the theoretical information, a cluster of five molecules DP5 and DP1–4 carrying gradually increasing electron-rich properties at 2- and 11-positions were selected to be synthesized (Fig. 1b). They were all obtained in excellent yields through a one-step bis-(trifluoroacetoxy) iodobenzene (PIFA)-mediated Hofmann reaction of 2-(pyridin-2-yl)imidazo-pyridine-3-carboxamides (amides 1–5) which can be readily prepared from 2-aminopyridines and picolinoyl acetic acetates in two steps.^7^ To our delight, the calculated theoretical values for the absorption maximum and redox potentials were found to be in good accordance with the experimental results qualitatively. As expected, all of them showed excited state redox potentials comparable to commonly used PCs.^1*g*,*h*^ From DP5, DP1 to DP4, the reductive potentials of the excited state decrease gradually from 1.15 to 0.69 eV and the oxidative potentials of the excited state increase from −1.44 to −1.77 eV. Notably, DP4 presents a stronger reductive potentiality than representative metal-core or organic visible light PCs. It is also predicted that the compounds DPs bearing EWGs at 2- and 11-positions might possess a stronger oxidative potential. The frontier molecular orbitals (FMOs shown in Table S2†) of DPs also illustrate that the methoxyl group involves and affects the redox properties. Such theoretical information and the experimental results hint at the possibility to rationally design new members of this photocatalytic series with increasing redox potential and the absorption maximum by modulating the nature and position of the substituents on two pyridine rings.

**Scheme 1:**
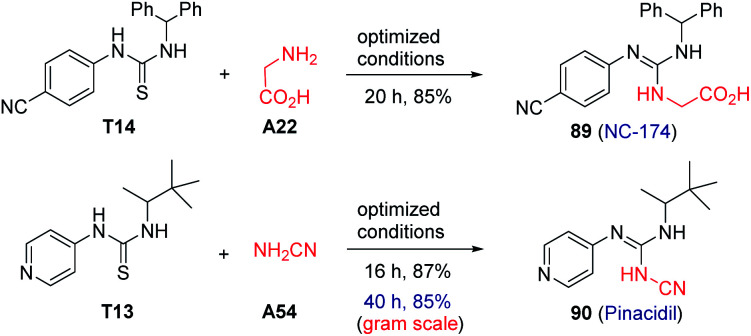
Gram scale study and synthetic applications to drug samples. Reaction conditions: T13–14 (0.3 mmol), A22 and A54 (0.6 mmol), K_2_CO_3_ (0.6 mmol), DP4 (1 mol%), in EtOH–H_2_O (9 : 1; 3.0 mL), air atmosphere, a.t., 435–440 nm blue LED, isolated yields. 5.0 mmol of T15 was used for the gram-scale reaction.

### DP4 enabled the productive guanylations of amines with thioureas through a reductive quenching cycle

**Fig. 2:**
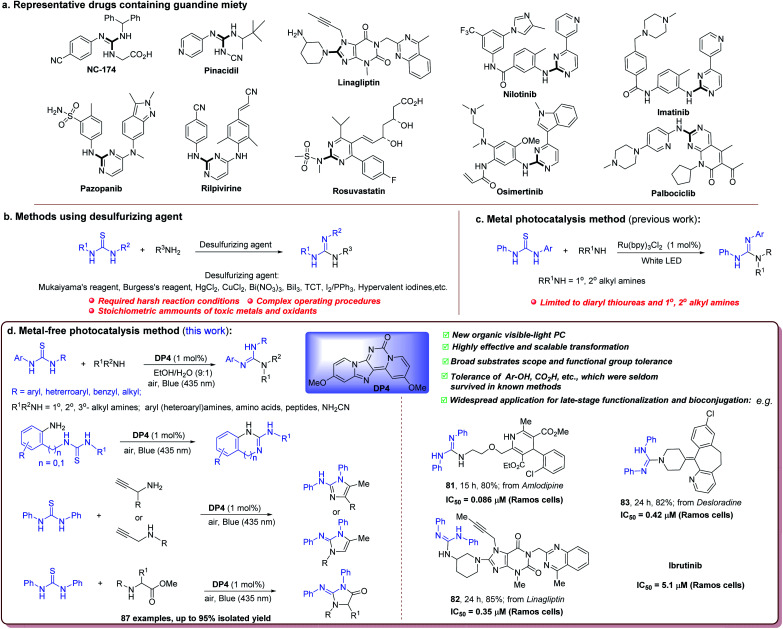
Significances and strategies for guanylation of amines with thioureas. (a) Representative drugs containing guanidine moiety (NC-175, a high potency synthetic sweetener; pinacidil, antihypertensive drug; linagliptin, orally bioavailable hypoglycemic drug; nilotinib, antihypertensive drug; imatinib, antileukemia drug; pazopanib, antineoplastic drug; rilpivirine, antiviral drug; rosuvastatin, a competitive HMG-CoA reductase inhibitor; osimertinib, antihypertensive drug; and palbociclib, orally available antineoplastic drug). (b) Strategies using desulfurizing agents. (c) Catalysis method using metal PC. (d) This work: productive guanylation of diverse amines catalyzed by our new organic visible-light PC (DP4).

Acyclic and cyclic guanidines are a growing number of biologically and pharmaceutically important compounds (Fig. 2a)^8^ and also serve as important building blocks and organocatalysts in organic synthesis.^9^ For example, guanidine NC-174 is a high potency synthetic sweetener,^8*c*^ and pinacidil is a treatment drug for hypertension.^8*d*^ Eight cyclic guanidines including palbociclib, imatinib, pazopanib, linagliptin, rosuvastatin, nilotinib, rilpivirine, and osimertinib (Fig. 2a) are among the top 200 pharmaceutical products by retail sales in 2018. Therefore, their synthesis has been extensively studied. Among the existing synthetic methods of substituted guanidines from amines using various guanylating agents, guanylation of amines with thioureas is the most promising because of the stable, cost-effective and readily accessible thiourea reactants.^10^ The known approach to the guanidines from thioureas and amines involves treatment with various desulfurizing agents (Fig. 2b), including copper chloride,^11^ mercury chloride,^12^ Bi(iii)/BiO_3_,^13^ TCT (trichloro cyanuric acid),^14^ Burgess's reagent,^15^ Mukaiyama's reagent,^16^ I_2_/PPh_3_,^17^ and hypervalent iodine.^18^ Although such reagents are highly efficient and compatible with a variety of functional groups, these systems still suffer from various limitations such as the use of stoichiometric toxic metals, stoichiometric amounts of oxidants, harsh reaction conditions, complex operating procedures, and limited substrate scope. Therefore, a visible-light photocatalytic synthesis of guanidines would be highly attractive for both academic and industrial adoption.

Very recently, Wacharasindhu's group reported the first photocatalytic process for guanylation of thioureas under visible light.^19^ They screened the common visible-light organic PCs including eosin Y, rose bengal, and safranin O, as well as a transition-metal photoredox catalyst Ru(bpy)_3_Cl_2_. Among them, only Ru(bpy)_3_Cl_2_ showed good catalytic activity and enabled the guanylation of diaryl thioureas with 1° and 2°-alkyl amines in high yields (Fig. 2c).^19^ To date, the photocatalytic transformation of alkyl thioureas with aryl amines, biologically important amino acids and aminonitrile to the corresponding guanidines, especially valuable cyclic guanidines remains hugely challenging and is still not realized. Herein, we wish to present a visible-light photocatalytic and productive synthesis of diverse guanidines and cyclic guanidines by employing DP4 as an oxidative PC (Fig. 2d).

### The guanylation of diverse amines with thioureas

To test the visible-light photocatalytic activity of DP1–DP5, we commenced our investigations by employing them in the guanidines formation. After optimization study using diphenyl thiourea (T1) and morpholine (A1) as model substrates for the guanylation (see Table S4 in ESI† for detail), the optimal condition using 1.0 mol% of DP4 as a photocatalyst, 2.0 equiv. of K_2_CO_3_ as a base, under visible-light irradiation (blue 435 nm) in a mixture of ethanol and water as a green solvent and air atmosphere at ambient temperature (a.t.) emerged from these surveys (Table S4,† entry 4). Under the optimized conditions, a less nucleophilic aromatic amine aniline (A17) and some common PCs such as Ru(bpy)_3_Cl_2,_ Ir(ppy)_2_(bpy)PF_4_, pyrene, 4CzTPN (1,2,4,5-tetrakis(carbazol-9-yl)-3,6-dicyanobenzene), eosin Y, and mes-Acr-Me^+^ (9-mesityl-10-methyl acrdinium perchlorate) were further tested for the guanylation with T1, as shown in Table 1. Gratifyingly, our photocatalyst DP4 outperformed these commonly used PCs.

**Table 1:** The guanylation for diphenyl thiourea with aniline photocatalyzed by DP4 and other commonly used PCs

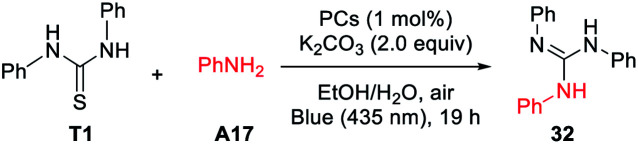							
PCs:	DP4	Ru(bpy)_3_Cl_2_	Ir(ppy)_2_(bpy)PF_6_	Pyrene	4CzTPN	Eosin Y	Mes-Acr-Me^+^
Yields:	70%	60%	59%	9%	28%	61%	58%

With the standard reaction conditions in hand, various amines including 1°, 2°, and 3°-alkyl primary amines, secondary amines (A1–14), amides (A15, A16),aryl and heteroaryl amines (A17–21), and α-amino acids (A22–31) were tested in the guanylation reaction with diaryl thioureas (T1–10), *N*-aryl-*N*-alkyl thioureas (T11–15) and dialkyl thiourea (T16) as shown in Table 2. Except for the inactive substrates dialkyl thiourea (T16) and amides (A15, A16), the reactions of other amines and thioureas substrates produced the corresponding acyclic guanidines 1–15, 17–29, and 32–49 in good to excellent yields and showed excellent compatibility with various reactive functional groups such as hydroxyl, halides, ester, alkene, alkyne, especially phenolic hydroxyl and carboxyl.

**Table 2:** Substrates scope of DP4-catalyzed visible light-drived guanylation of various thioureas with amines^a^

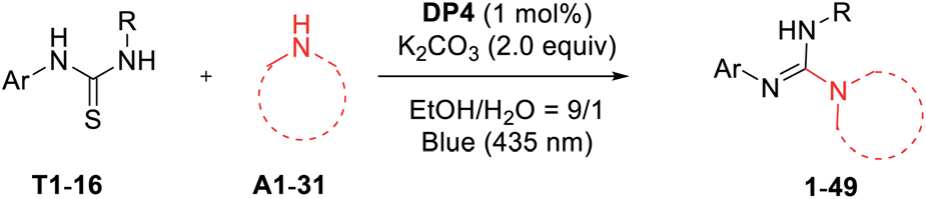
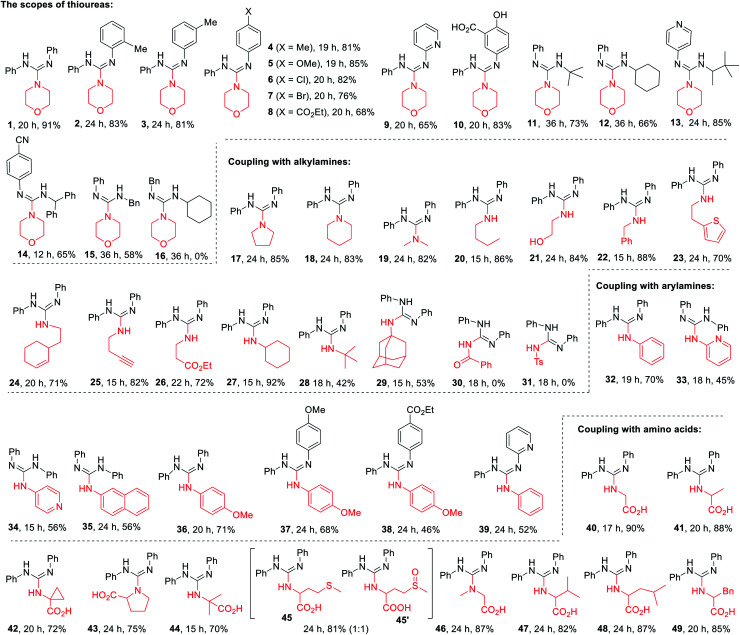

a Reaction conditions: thioureas (0.3 mmol), amines (0.6 mmol), K_2_CO_3_ (0.6 mmol), DP4 (1 mol%), in EtOH–H_2_O (9 : 1; 3 mL), air atmosphere, a.t., 435–440 nm blue LED, isolated yields. DP4 was used in 3 mol% for 6, 7, 12, 13, 15–19, 39, 43, 44, 46. 4.0 equiv. of K_2_CO_3_ was used for 40–49.

The scope of thioureas was firstly explored in the guanylation of morpholine (A1). Diaryl thioureas (T1–10) containing EDGs or EWGs reacted with A1 to provide guanidines 1–10 in good to excellent yields. The electron-defect thioureas afforded the guanidines (8 and 9) in slightly lower yields. Notably, the phenolic OH and carboxyl groups were well tolerated in the guanidine (10) formation, which seldom survived in previous methods. Gratifyingly, in contrast to Ru(bpy)_3_Cl_2_,^19^DP4 enabled the transformation of alkyl and benzyl thioureas in good to excellent yields (11–15). The dialkyl thiourea T16 was inactive for this transformation, the desired product 16 was not observed and T16 was recovered in 91%. Then, we extended the scope of this transformation further to other amine substrates (A2–14). Various primary and secondary amines reacted with T1 affording the corresponding guanidines 17–27 in high to excellent yields, and the reactive thenyl, alkenyl, alkynyl and ester groups were well tolerated (24–27). Particularly worth mentioning is that *t*-butyl amine and amantadine underwent this transformation well with satisfactory yields (28 and 29). Not surprisingly, products 30 and 31 were not obtained when benzamide A15 and *p*-toluenesulfonamide A16.

Of particular note is the photocatalytic guanylation of aryl amines and amino acids has not been reported to date. We were pleased to observe that various arylamines including phenylamines, pyridyl amines, and betanaphthyl amine underwent this transformation well with yields of 45−71% (32–39). Most notably, the reactions proceeded with high to excellent yields (40–49) on a series of amino acids, thus suggesting a possible application of this methodology in bioconjugation chemistry. For the reaction of methionine, the desired product 45 and further oxidative compound 45′ were obtained in a ratio of 1 : 1 with a total yield of 81%. These results highlight the powerful activity of DP4 in this photocatalytic transformation.

### The application to cyclic guanidines 2-amino-benzimidazoles and 2-amino-quinazolines synthesis

2-Amino-benzimidazoles and 2-amino-quinazolines are a hugely important class of compounds that have exhibited analgesic, immunosuppressive, antihistamine, antiinflammatory, and antiviral activities.^8*k*,20^ Inspired by the successful guanylation of arylamines, we extended the application of this methodology to the synthesis of such cyclic guanidines (50–58, Table 3) through an intramolecular guanylation. A series of 1-(2-aminophenyl)-3-arylthioureas (TA1–6), 1-(2-(aminomethyl)phenyl)-3- arylthioureas (TA7–9) carrying EDGs or EWGs had no effect on this transformation affording the corresponding *N*-phenyl-1*H*-benzo[*d*]imidazol-2-amines (50–55) and *N*-phenyl-quinazolin-2-amines (56–58) in excellent yields. The biologically important *N*-phenyl-quinazolin-2-amines (59–61) could be easily obtained through the oxidation of 56–58 by dichlorodicyanobenzoquinone (DDQ).

**Table 3:** The intramolecular guanylation for 2-amino-benzimidazoles and 2-amino-quinazolines synthesis

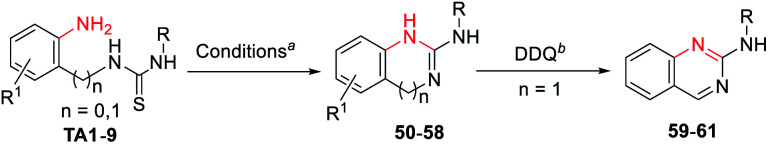
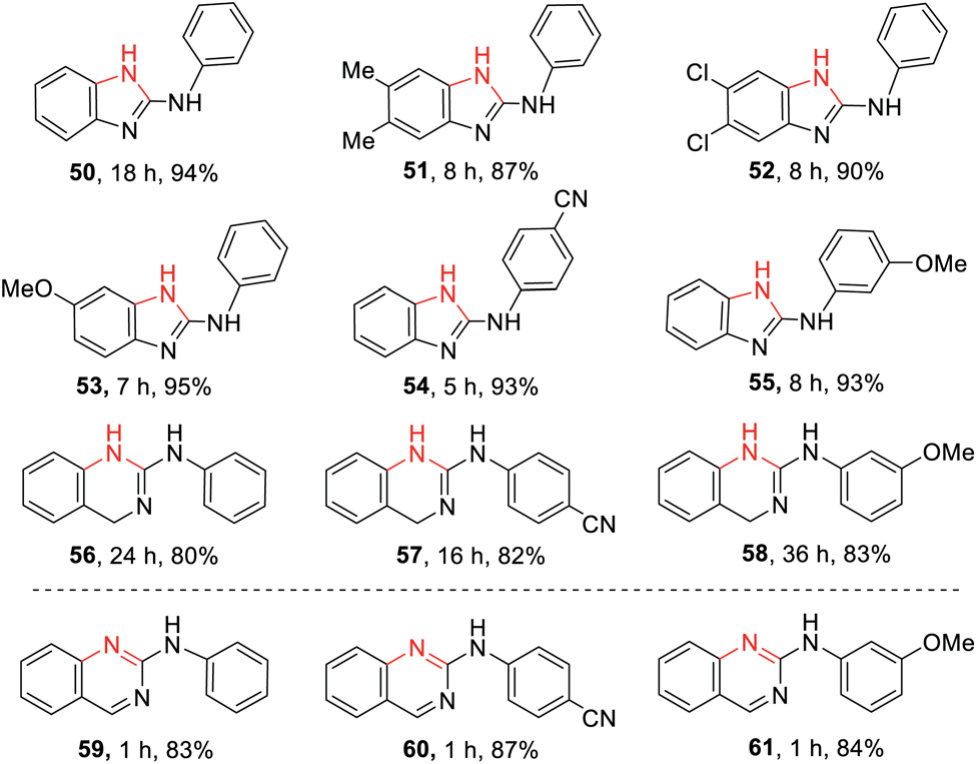

a Reaction conditions: TA1–9 (0.3 mmol), K_2_CO_3_ (0.6 mmol), DP4 (1 mol%), in EtOH–H_2_O (9 : 1; 3.0 mL), air atmosphere, a.t., 435–440 nm blue LED, isolated yields.

b 56–58 (0.3 mmol), DDQ (0.45 mmol), in MeCN (3.0 mL), a.t.

### The application to cyclic guanidines 2-aminoimidazoles and 2- iminoimidazoles synthesis

2-Aminoimidazoles, 2-imino-imidazoles and 2-iminoimidazolin-4-ones are frequently found in the application of coordination chemistry,^21^ and pharmaceutical chemistry.^22^ The scope of this method was further extended toward the cascade synthesis of these cyclic guanidines (Table 4). As representative examples, propargylamines A32–33 and *N*-substituted propargylamines A34–35 underwent this photocatalytic reaction with diphenyl thiourea T1 affording the corresponding imidazoles 62–65 in yields of 55–70%. The cascade process includes the photocatalytic guanylation of T1 with propargylamines and an intramolecular alkyne hydroamination. To our delight, when a series of α-amino acid esters A36–43 were applied to the present conditions, the corresponding 2-iminoimidazolin-4-ones 66–73 were obtained in good to high yields.

**Table 4:** The cascade synthesis of cyclic guanidines.^a^

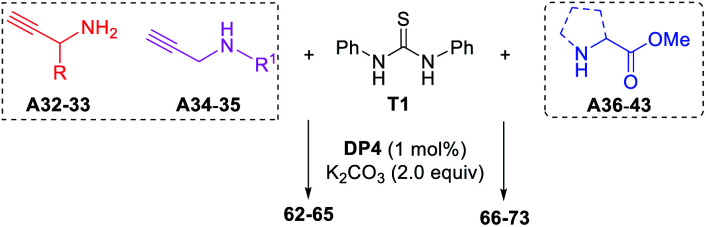
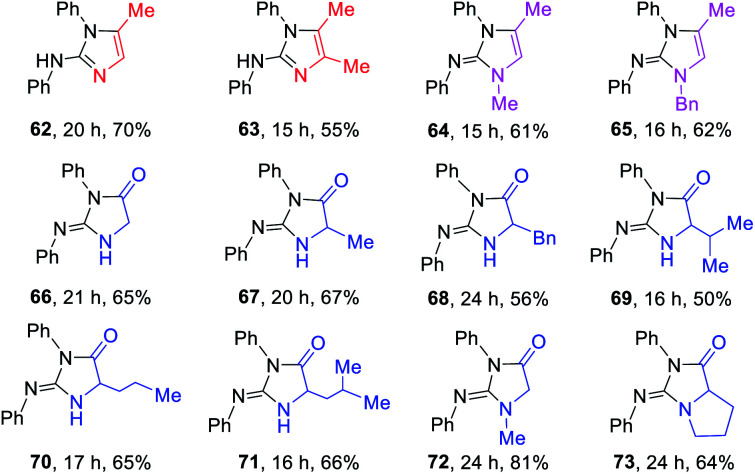

a Reaction conditions: T1 (0.3 mmol), A32–35 (0.6 mmol), K_2_CO_3_ (0.6 mmol), DP4 (1 mol%), in EtOH–H_2_O (9 : 1; 3.0 mL), air atmosphere, a.t., 435–440 nm blue LED, isolated yields. 3.0 equiv. of K_2_CO_3_ was used for 66–73.

### Late-stage functionalization of peptides and medicinally relevant molecules

The late-stage functionalization (LSF) of complex biorelevant molecules is a powerful tool to hasten the identification of structure–activity relationships and to optimize ADME profiles.^1*n*^ To further demonstrate the broad application of this method, the LSF of biologically important peptides and medicinally relevant molecules were conducted (Table 5). Five unprotected peptides underwent this photocatalysis with good to excellent yields (74–78). Likewise, this versatile method can be also highly effectively applied in the LSF of amino-containing bioactive intermediates and complex drugs (79–88). Notably, the advantages of this method were further illustrated by the successful coupling of drugs 4-aminopyridine (4-AP) and 5-aminosalicylic acid (5-ASA) with amlodipine, desloradine, and linagliptin for assembling their drug-like hybrids 84–88, highlighting the potential applications of this method in the discovery of pharmaceutical candidates.

**Table 5:** Late-stage functionalization of peptides and medicinally relevant molecules^a^

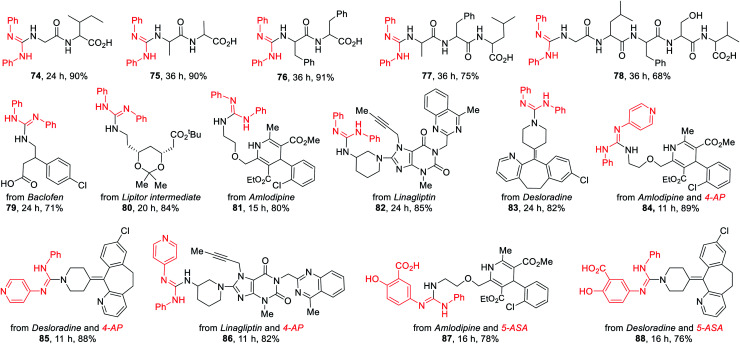

a Reaction conditions: thioureas (0.3 mmol), peptides or drugs (0.6 mmol), K_2_CO_3_ (0.6 mmol), DP4 (1 mol%), in EtOH–H_2_O (9 : 1; 3.0 mL), air atmosphere, a.t., 435–440 nm blue LED, isolated yields. 4.0 equiv. of K_2_CO_3_ was used for 74–78.

### Application to the preparation of drug pinacidil and NC-174 and gram-scale study

It is worth highlighting that this method was amenable to the direct synthesis of the drug pinacidil (90) and NC-174 (89) in excellent yields (Scheme 1). Additionally, a gram-scale reaction was successfully performed for the preparation of pinacidil in 85% yield under the optimized conditions, exemplifying the practicability and scalability of this photocatalytic transformation.

### Mechanism study

**Scheme 2:**
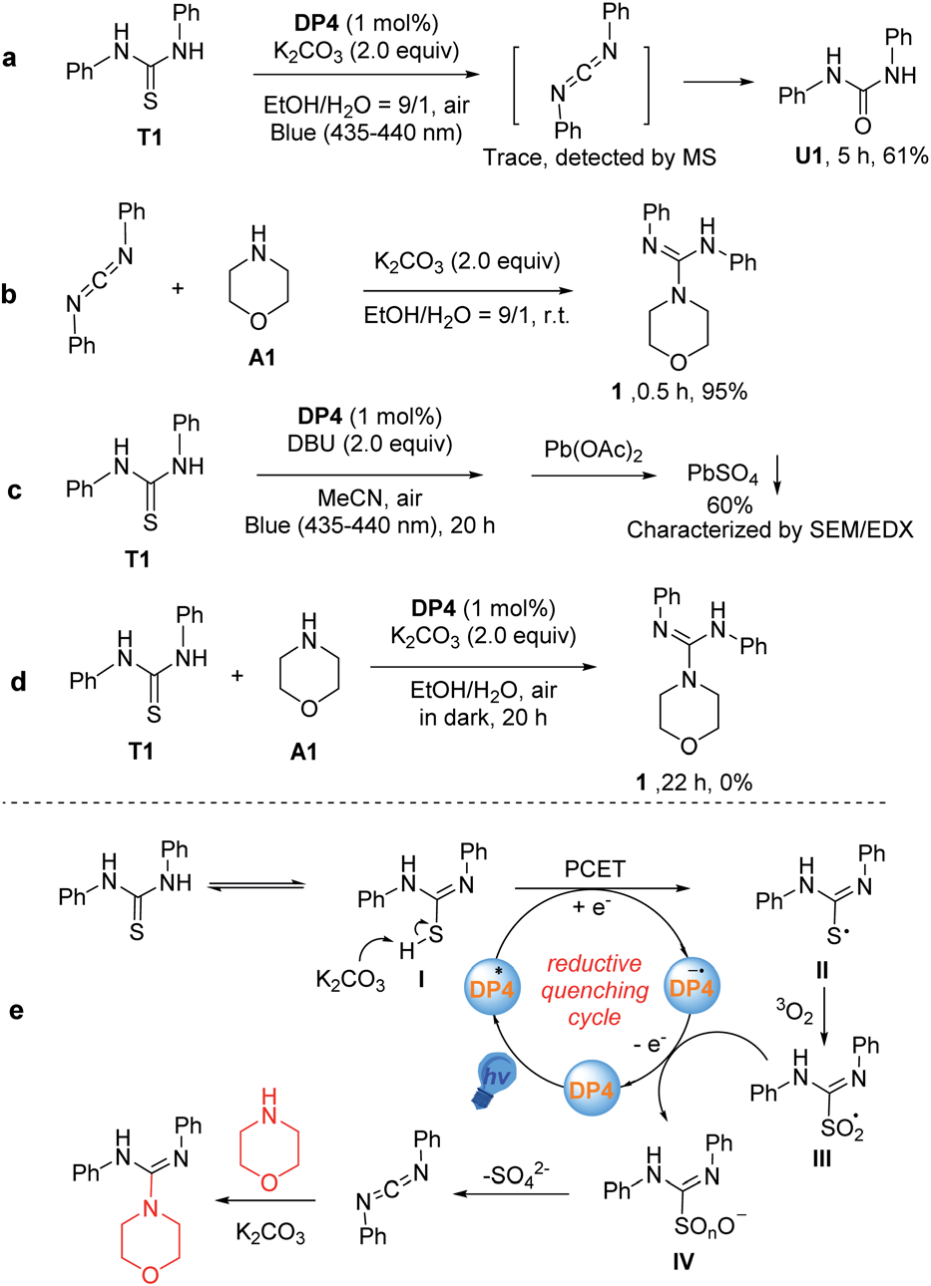
Preliminary mechanistic studies (a–d) and the proposed mechanism (e).

To propose a reasonable reaction mechanism of this guanylation reaction, several control experiments were conducted (Scheme 2a–d). Firstly, we carried out the reaction with T1 under a blue LED in the absence of amine. As a result, we obtained compound 1,3-diphenylurea (U1) in 61% yield after 5 hours under optimal reaction conditions, instead of the target product 1 (Scheme 2a). Importantly, a trace amount of intermediate diphenylmethanediimine was detected by MS. Further investigation disclosed that diphenylmethanediimine, which was prepared following a known procedure,^23^ could have reacted with amine A1 and gave the desired compound 1 in the yield of 95% after 0.5 h without the irradiation by blue LED (Scheme 2b). These observations showed that diphenylmethanediimine may be involved in the transformation. Furthermore, in order to clarify the formation of the S atom at the end of the transformation, we added Pb(OAc)_2_ to the reaction mixture after the reaction of T1 in the absence of amine A1, and use DBU as the base instead of K_2_CO_3_ avoiding the generation of PbCO_3_ precipitate (Scheme 2c). This control experiment gave PbSO_4_ in 60% yield from the reaction mixture, which was confirmed by SEM/EDX (Fig. S19 and Table S5†). The reaction in the absence of light was also conducted and no product was detected, indicating that the current reaction is a blue light-induced photocatalysis (Scheme 2d). The experiments with active oxygen species inhibitors, such as singlet oxygen (^1^O_2_) inhibitors (1,3-diphenylisobenzofuran and 9,10-dimethylanthracene), superoxide radical 
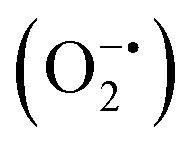
 inhibitors (BQ and 1,3-diphenylisobenzofuran), and hydroxyl radical (˙OH) inhibitor (^*t*^BuOH) showed that such transformations were not obviously affected by the addition of these inhibitors (Fig. S6 in ESI†).^24^ These results indicated that the reactive oxygen species might be ground-state oxygen (^3^O_2_), however, a minor contribution from other active oxygen species could not be completely excluded. Based on the mechanistic studies and previous reports,^19,24*a*^ although the mechanism of this visible-light-catalyzed guanylation reaction was not unequivocally demonstrated, a reasonable possibility is outlined in Scheme 2e. The tautomer I of diphenylthiourea is transformed into a radical intermediate II through a proton-coupled electron transfer (PCET) to the excited state catalyst DP4*. The ensuing coupling between II and ground-state oxygen produces the persulfoxide radical III. Subsequently, DP4^−^˙ donates an electron back to III, recycling the DP4 catalyst and producing a peroxysulfur intermediate IV. Finally, sulfate is released to form a carbodiimide as a key intermediate, which was rapidly attacked by the amine to produce the final product. Alternatively, II might be oxidized to IV by other active oxygen species, which could not be ruled out at present.

### Bioactivities for selected LSF derivatives

**Fig. 3:**
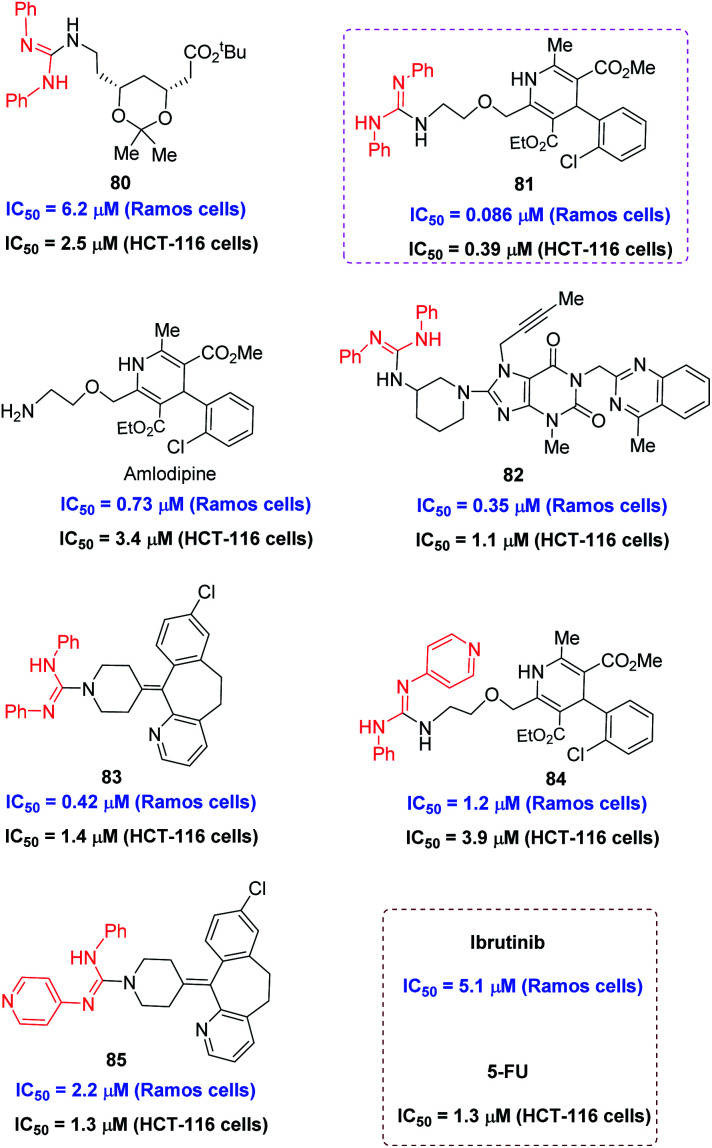
IC_50_ of selected LSF derivatives toward Ramos and HCT-116 cells.

The utility of this protocol was next identified in the preliminary bioactivity study on several selected LSF derivatives. Considering good bioactivities for the molecules containing guanidine moiety, the introduction of the guanyl group in biomolecules may give improved activities. Thus, representative coupled products 80-85 were tested toward the human B lymphoma cell line Ramos cells and human colorectal adenocarcinoma cell line HCT-116 cells, giving the 50% inhibitory concentration (IC_50_) of cell death at micromolar or nanomolar level (Fig. 3, see ESI† for more information). The cell antiproliferative activity of these compounds was evaluated against Ramos cells and HCT-116 cells using the CellTiter-Glo (Promega, USA) assay. As shown in Fig. 3, all the tested compounds are active toward these two cell lines. In Ramos cells, the compounds 81, 82, and 83 exhibited much better activities, with IC_50_ of 0.086, 0.35, and 0.42 μM, respectively, than that by the clinical drug Ibrutini with IC_50_ of 5.1 μM. Notably, the compounds of 81, exhibited potent activity against Ramos cells with the IC_50_ value of 0.086 μM, which was 60-fold better than that of ibrutinib. In HCT-116 cells, all the tested compounds exhibited comparable activities to or better activities than the control drug 5-fluorouracil (5-FU). Of particular note is that the guanylated derivative 81 of amlodipine exhibited more than 8-fold better activities than amlodipine against these two cell lines. These preliminary bioactivity results indicated that the application of this protocol to LSF of known bioactive molecules and drugs may afford a new class of anticancer candidates.

## Experimental

### General procedure for preparation of photoredox catalyst DP4

Amide4 (149.1 mg, 0.5 mmol) and PIFA (236.5 mg, 0.55 mmol) were added to a round-bottom flask (25 mL), and the reaction mixture was stirred about 30 min in CH_3_CN (10 mL) at room temperature (monitored by TLC). Then, the reaction mixture was treated with a saturated NaHCO_3_ solution (10 mL) and extracted with dichloromethane (3 × 10 mL). The combined organic layer was dried over anhydrous Na_2_SO_4_ and concentrated using a rotary evaporator. The crude product was further purified by recrystallization with ethyl ether (15 mL) to produce the desired product DP4 (133.2 mg, 90%).

### General procedure for guanylation amines with thioureas (1 as an example)

A Pyrex glass tube equipped with a magnetic stirring bar was charged with K_2_CO_3_ (82.9 mg, 0.6 mmol), DP4 (0.9 mg, 1 mol%), T1 (68.4 mg, 0.3 mmol), A1 (52.5 μL, 0.6 mmol), and ethanol/H_2_O (9 : 1, 3 mL). Then, the reaction vessel was placed away from blue LED 2.5 cm. The reaction mixture was stirred at room temperature for 20 h irradiated by a blue LED (monitored by TLC). The reaction mixture was treated with H_2_O (5 mL) and extracted with DCM (3 × 10 mL). The combined organic layer was dried over anhydrous Na_2_SO_4_ and concentrated on a rotary evaporator. The residue was purified on silica gel using petroleum ether/ethyl acetate/TEA (10 : 1 : 1) as the eluent. Product 1 was obtained as a white solid (76.7 mg, 91%).

## Conclusions

In summary, we have demonstrated a rational *de novo* design and the synthesis of a new class of DP-based organic PCs. The experimental data on the synthesized PCs DP1–5 were found to be in good agreement with the theoretical values qualitatively. They showed excited state redox potentials comparable to the representative metal-core or organic photoredox catalysts. As a representative, DP4 was then showcased in the guanylation of an extremely broad range of structurally diverse amines with thioureas and more than 87 examples have been presented in good to excellent yields. The virtues of this chemistry including the broad substrate scope, excellent functional group tolerance in both coupling partners, as well as the efficient functionalization of peptides and medicinally relevant molecules that led to several very potent anticancer active molecules Bode well for the widespread applications of this chemistry in constructing materials and fine chemicals, and drug development. The photocatalytic efficacy of DP4 was outperforming some commonly used metal-core and organic PCs in this guanylation, demonstrating once again that organic PCs may offer far more than “metal-free” alternatives to transition metal examples. These results validate the use of the present *de novo* strategy in guiding the rational design of organic photoredox catalysts. In light of their ready synthesis and the highly tunable optical properties of DPs, the strategy disclosed here may pave the way for future discovery of new powerful visible-light PCs and applications in promoting a broader scope of organic transformations.

## Data availability

All experimental procedures and spectroscopic data can be found in the ESI.†

## Author contributions

Z. Z., and G. Z. conceived the idea and guided the project. Y. W., H. W., and W. G. performed the experiments and analyzed the results. Y. W., and N. M. performed the DFT calculations. J. Z., and Y. W. performed the bioassay experiments and analyzed the results. G. Z. wrote the manuscript.

## Conflicts of interest

There are no conflicts to declare.

## Supplementary Material

SC-012-D1SC05294B-s001
