# Supplementary material for: De novo design and synthesis of dipyridopurinone derivatives as visible-light photocatalysts in productive guanylation reactions
Source: Chem Sci. 2021 Nov 13;12(48):15988–97. doi: 10.1039/d1sc05294b (PMC8672711; doi:10.1039/d1sc05294b)
Supplement: SC-012-D1SC05294B-s001 [file SC-012-D1SC05294B-s001.pdf]

Electronic Supplementary Material (ESI) for Chemical Science.
This journal is © The Royal Society of Chemistry 2021

Supplementary Information for

De Novo design and synthesis of dipyridopurinone derivatives as visible-light photocatalysts in productive guanylation reactions

Yameng Wan[1], Hao Wu[1], Nana Ma[1], Jie Zhao[1], Zhiguo Zhang[1,*], Wenjing Gao[1], and Guisheng Zhang[1,*]

[1]Collaborative Innovation Center of Henan Province for Green Manufacturing of Fine Chemicals, Key Laboratory of Green Chemical Media and Reactions, Ministry of Education, NMPA Key Laboratory for Research and Evaluation of Innovative Drug, School of Chemistry and Chemical Engineering, Henan Normal University, Xinxiang, Henan 453007, China

Corresponding authors. E-mail: zhangzg@htu.edu.cn; zgs@htu.cn.

Contents

General information	S3
Materials and Methods	S3-S4
Theoretical calculation	S4-S10
Overview of the photophysical properties of **DP1**-**DP5**	S10-S16
Reaction conditions optimization	S16-S18
Gram-scale preparation of Pinacidil	S18
Mechanistic studies	S18-S20
Characterization of compounds	S20-S50
References	S51-S52
HPLC analysis data of **88**	S52-S53
Bioactivities for Selected Products	S54-S56
NMR spectra of compounds	S57-S141

General information

^{1}H NMR and ^{13}C NMR spectra were recorded on a 400/600 MHz NMR spectrometer (^{1}H NMR, 400/600 MHz; ^{13}C NMR, 100/150 MHz at 25 °C). Coupling constants are reported in Hz. Multiplicities were given as: singlet (s), doublet (d), triplet (t), quartet (q), doublet of doublet (dd), quintet (quint.), septet (sept.), multiplet (m) etc. All high-resolution mass spectra (HRMS) were measured on a mass spectrometer (ESI-oa-TOF). All reagents were purchased from commercial sources and used without further treatment. All reactions were monitored by thin layer chromatography (TLC). UV/Vis spectra were recorded on a Lambd 950 spectrophotometer in CH_2Cl_2. The redox potentials measurements were carried out by cyclicvoltammetry (CV).

Materials and Methods

Amides1-5 (Figure S1) were synthesized following the procedure described in literatures.[1] Unless otherwise noted**,** all thioureas (Figure S1, **T1-T16; TA1-TA9**) were synthesized following the procedure described in literatures.[2] Starting materials such as amines (Figure S1, **A1-A21**; **A32-A43**; **A49-A54**), amino acids (Figure S1, **A22-A31**), and peptides (Figure S1, **A44-A48**) used in the article are purchased commercially. All reactions were irradiated with blue LEDs (435-440 nm, 20 W).

Figure S1. Series of materials used in text.

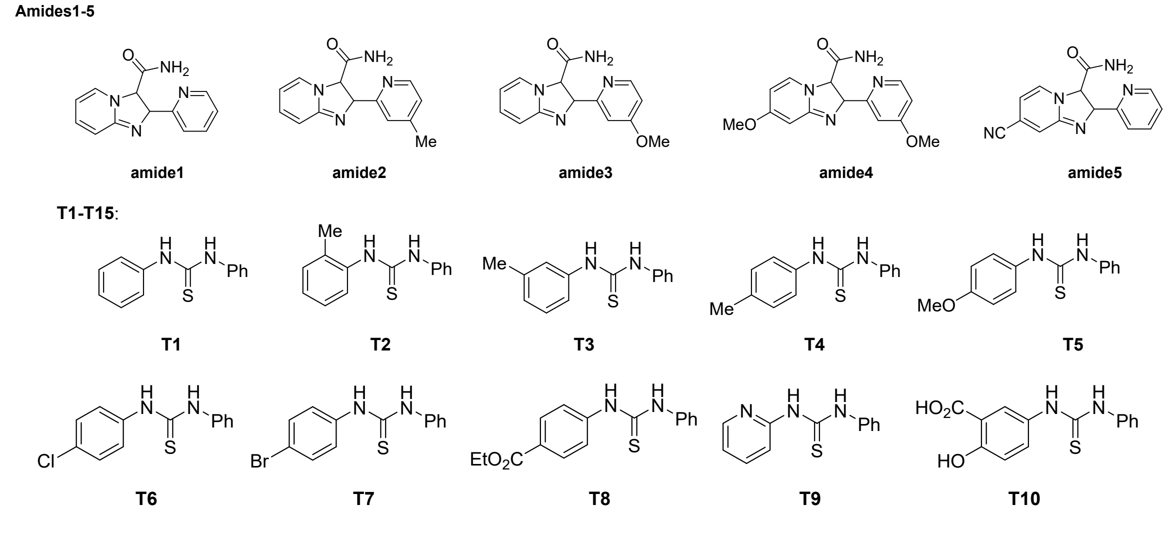

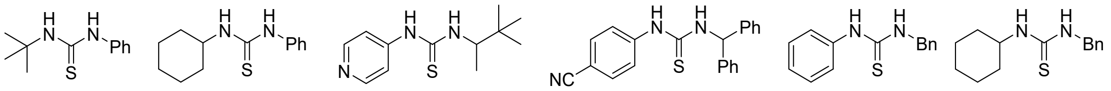

T11 **T12** **T13** **T14** **T15** **T16**

A1-A52:

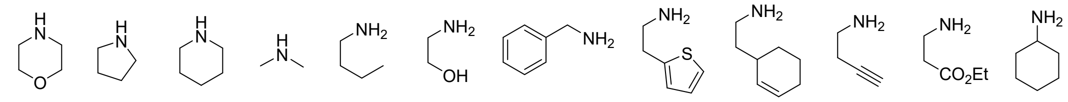

A1 **A2** **A3** **A4** **A5** **A6** **A7** **A8** **A9** **A10** **A11** **A12**

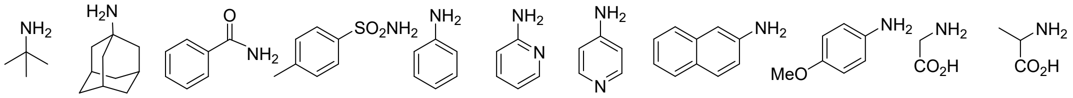

A13 **A14** **A15** **A16** **A17** **A18** **A19** **A20** **A21** **A22** **A23**

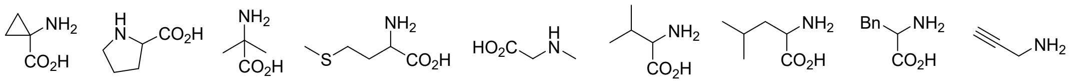

A24 **A25** **A26** **A27** **A28** **A29** **A30** **A31** **A32**

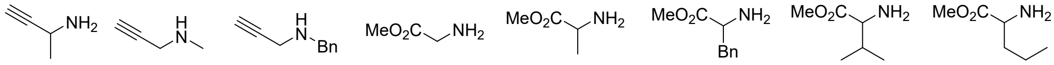

A33 **A34** **A35** **A36** **A37** **A38** **A39** **A40**

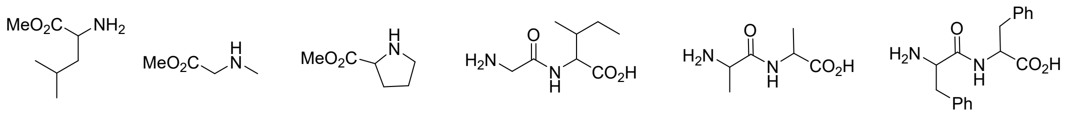

A41 **A42** **A43** **A44** **A45** **A46**

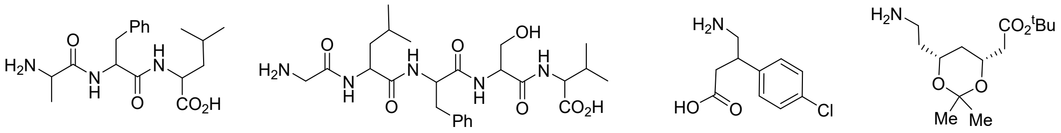

A47 **A48** **A49** **A50**

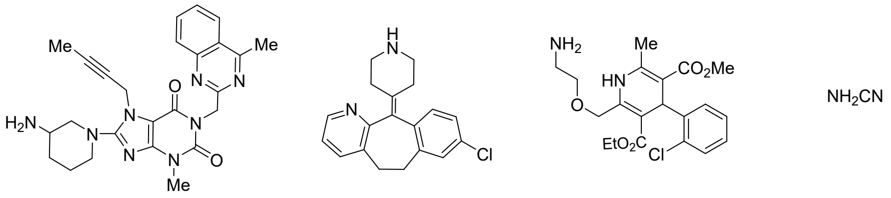

A51 **A52** **A53** **A54**

TA1-TA9:

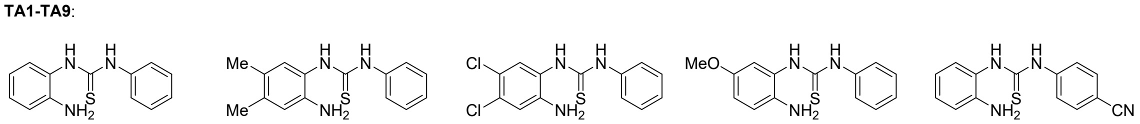

TA1 **TA2** **TA3** **TA4** **TA5**

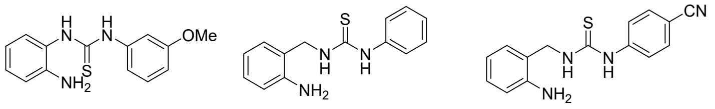

TA6 **TA7** **TA8** **TA9**

Theoretical calculation

For effective synthesizing 6*H*-dipyrido[1,2-e:2',1'-*i*]purin-6-ones photocatalysts (DPs),

we firstly designed a series of derivatives (50 compounds) with different electron withdrawing groups (EWGs) and electron donating groups (EDGs) (Please see the Figure S2). Then, we calculated the absorption maximum of UV-visible spectrum and their redox potentials by density functional theory (DFT) method.

All the geometries were optimized at the B3LYP/6–31+G(d) level in gas phase. To obtain accurate electronic properties, the solvent effect was employed by the continuum solvation model SMD[3] involving non-electrostatic interaction. Therefore, a single point calculation in dichloromethane solution was followed by the geometry in gas phase. Calculations of the absorption spectra were performed within the time-dependent density functional theory (TDDFT). Also, to consider the solvent effects in the energy and excitation properties, the SMD model in dichloromethane solution was employed in TDDFT calculations. PBE0 combined with 6–31+G(d) was employed and has a satisfactory simulation with experimental spectra (**DP1-DP5**).

The theoretical prediction of redox potential requires the difference of the free energy (ΔG) associated with the process:

$$\text{DPs (g)} + \text{e-} \rightarrow \text{DPs}^{-}\text{ (g)}$$

The *ΔG* of the reduction process can be obtained by frequency calculations of DPs and 1T–6T. The solvent effects of acetonitrile were included with the SMD model by single point calculations on the basis of the optimized geometries, i.e. at m06/6–311+G(d,p) level. Therefore, the electronic and thermal free energies in solution, G_{sol}, were obtained by taking account the thermodynamic corrections to the energy in solution, as the equation listed in the following:

$$G_{\mathrm{sol}} = E_{\mathrm{SMD}} + (\mathrm{G} - E)_{\mathrm{g}}$$

The *ΔG* value associated with the reference normal hydrogen electrode (NHE) half-reaction has been calculated to be –4.44 V.[4] So the reference standard calomel electrode (SCE) should be –4.68 V. The reduction potentials ($E_{1/2}$) can be calculated according to the Nernst equation $E_0 = -\Delta G_0/nF$.

Figure S2. Series of DP-based structures proposed as photocatalysts.

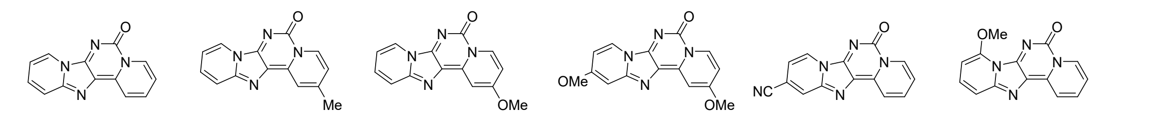

DP1 **DP2** **DP3** **DP4** **DP5** **DP6**

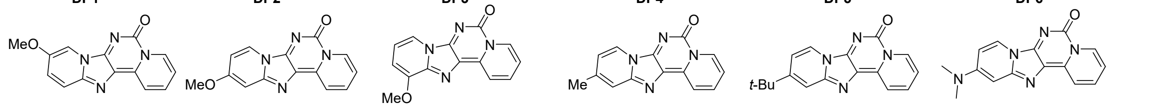

DP7 **DP8** **DP9** **DP10** **DP11** **DP12**

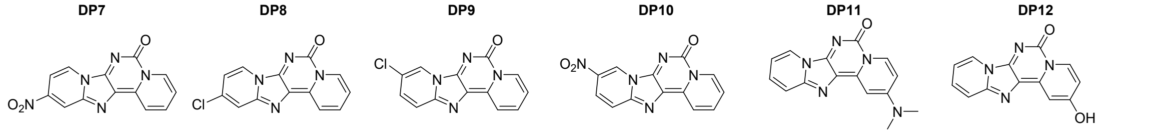

DP13 **DP14** **DP15** **DP16** **DP17** **DP18**

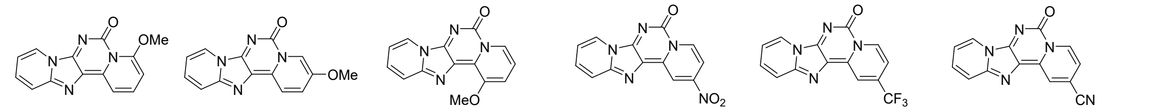

DP19 **DP20** **DP21** **DP22** **DP23** **DP24**

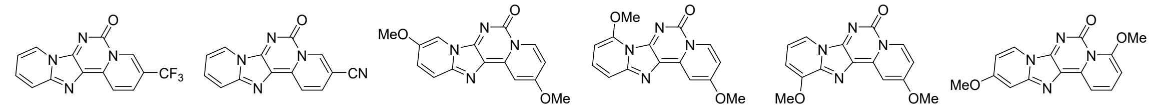

DP25 **DP26** **DP27** **DP28** **DP29** **DP30**

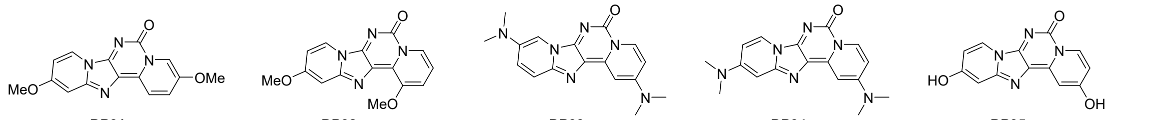

DP31 **DP32** **DP33** **DP34** **DP35**

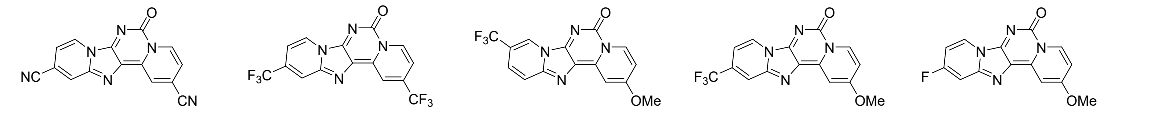

DP36 **DP37** **DP38** **DP39** **DP40**

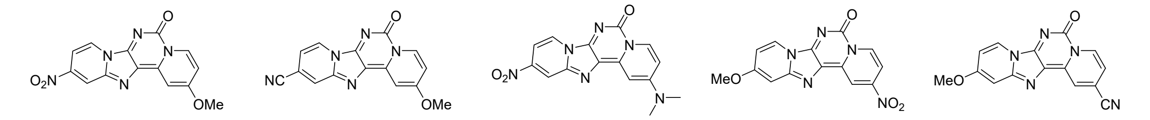

DP41 **DP42** **DP43** **DP44** **DP45**

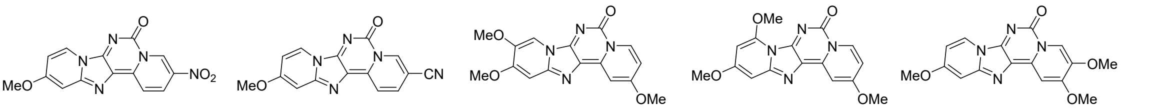

DP46 **DP47** **DP48** **DP49** **DP50**

Table S1. Calculated redox potentials, and absorption maxima (λ_{max}) for the proposed compounds.

Compound	$E_{1/2}$red (eV) [a,b]	$E_{1/2}$ox (eV) [a,c]	$\lambda_{max\ (nm)}$	Compound	$E_{1/2}$red (eV) [a,b]	$E_{1/2}$ox (eV) [a,c]	$\lambda_{max\ (nm)}$
**DP1**	-1.95	0.65	445	**DP26**	-1.64	0.85	467
**DP2**	-2.06	0.58	438	**DP27**	-2.29	0.47	436
**DP3**	-2.26	0.54	420	**DP28**	-2.36	0.37	422
**DP4**	-2.34	0.35	435	**DP29**	-2.30	0.51	411
**DP5**	-1.62	0.88	465	**DP30**	-2.08	0.30	474

**DP6**	-2.05	0.47	453	**DP31**	-2.04	0.37	478
**DP7**	-1.92	0.57	465	**DP32**	-2.06	0.38	466
**DP8**	-2.03	0.45	462	**DP33**	-2.45	0.45	413
**DP9**	-1.99	0.61	442	**DP34**	-2.36	-0.34	412
**DP10**	-1.99	0.56	446	**DP35**	-2.29	0.45	425
**DP11**	-2.0	0.58	446	**DP36**	-1.12	1.05	536
**DP12**	-2.18	0.20	483	**DP37**	-1.45	1.04	477
**DP13**	-0.85	0.87	488	**DP38**	-2.19	0.71	419
**DP14**	-1.89	0.72	445	**DP39**	-2.09	0.73	425
**DP15**	-1.87	0.76	444	**DP40**	-2.25	0.58	417
**DP16**	-1.38	0.93	418	**DP41**	-1.22	0.86	526
**DP17**	-2.44	0.40	414	**DP42**	-1.76	0.78	451
**DP18**	-2.12	0.58	420	**DP43**	-1.00	0.40	427
**DP19**[d]	\	\	\	**DP44**	-0.13	0.59	574
**DP20**	-1.98	0.56	458	**DP45**	-1.36	0.62	574
**DP21**	-1.98	0.56	451	**DP46**	-1.21	0.70	603
**DP22**	-0.55	0.66	477	**DP47**	-1.72	0.64	484
**DP23**	-1.55	0.80	488	**DP48**	-2.33	0.39	420
**DP24**	-1.29	0.82	547	**DP49**	-2.43	0.19	435
**DP25**	-1.71	0.81	461	**DP50**	-2.16	0.32	449

[a]Versus Satured Calomel Electrode (SCE). [b]$DP + 1e^- \rightarrow DP^-$; $E_{1/2}red = E_{1/2}$ (DP/DP^-). [c]$DP^+ + 1e^- \rightarrow DP$; $E_{1/2}ox = E_{1/2}$ (DP^+/DP). [d]The optimized geometry of **DP19** at B3LYP/6-31+G(d) level is different from other DPs, and the optimized geometry was shown in Table S2 and renamed it as **DP19’**.

Table S2. Topology of frontier molecular orbitals computed and values correspond to the orbital energies in eV.

Compound	HOMO	LUMO	Compound	HOMO	LUMO
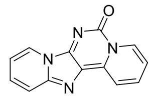 **DP1**	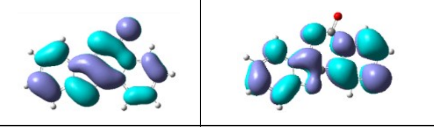 -5.41 eV	-2.45 eV	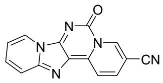 **DP26**	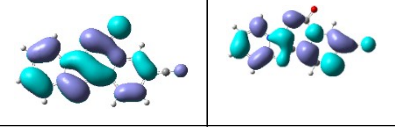 -5.85 eV	-2.98 eV
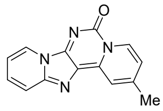 **DP2**	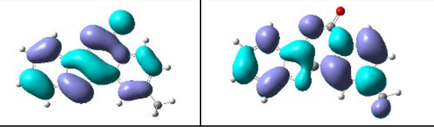 -5.31 eV	-2.31 eV	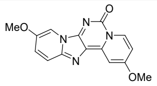 **DP27**	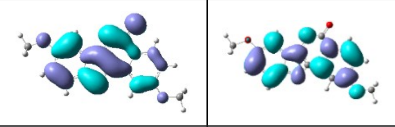 -5.08 eV	-2.07 eV
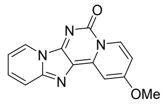 **DP3**	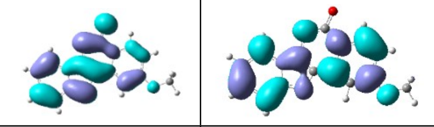 -5.23 eV	-2.10 eV	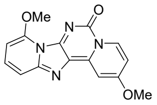 **DP28**	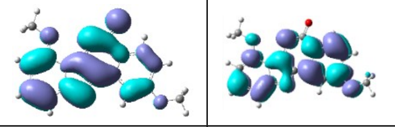 -4.97 eV	-1.85 eV
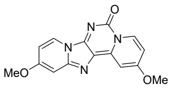 **DP4**	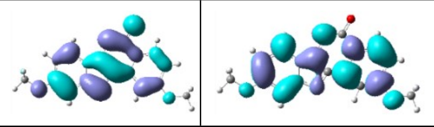 -5.00 eV	-1.97 eV	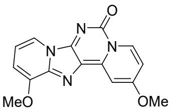 **DP29**	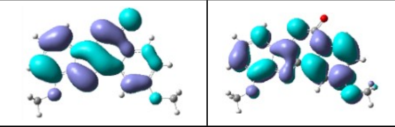 -5.14 eV	-1.94 eV
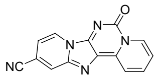 **DP5**	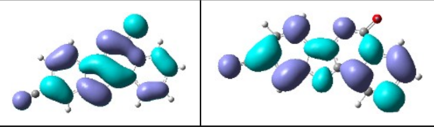 -5.86 eV	-3.01 eV	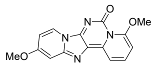 **DP30**	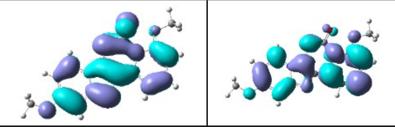 -4.94 eV	-2.12 eV
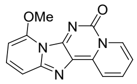 **DP6**	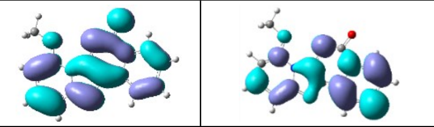 -5.15 eV	-2.22 eV	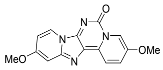 **DP31**	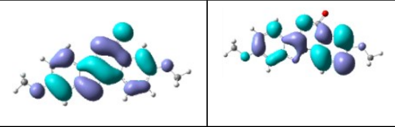 -5.02 eV	-2.25 eV
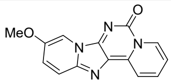 **DP7**	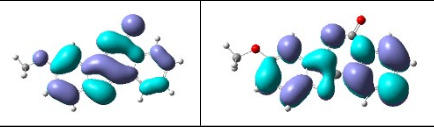 -5.25 eV	-2.41 eV	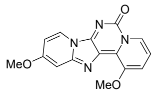 **DP32**	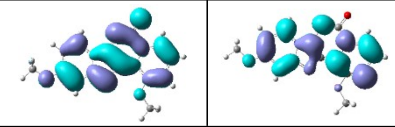 -5.03 eV	-2.20 eV
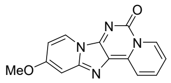 **DP8**	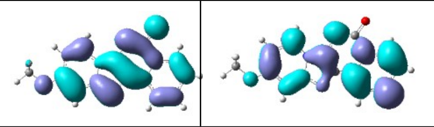 -5.18 eV	-2.32 eV	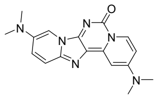 **DP33**	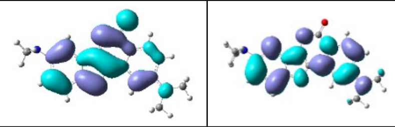 -4.96 eV	-1.79 eV
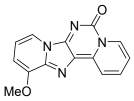 **DP9**	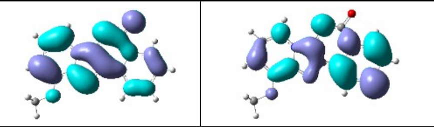 -5.31 eV	-2.32 eV	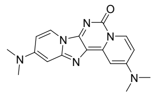 **DP34**	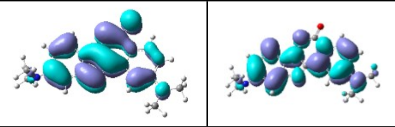 -4.96 eV	-1.77 eV

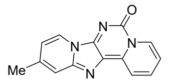 **DP10**	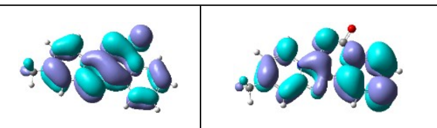	-5.33 eV	-2.36 eV	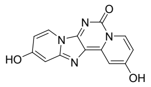 **DP35**	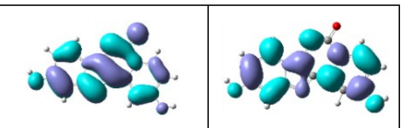	-5.19 eV	-2.10 eV
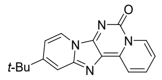 **DP11**	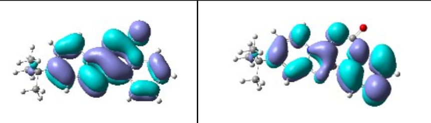	-5.30 eV	-2.34 eV	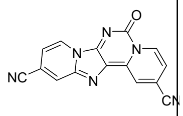 **DP36**	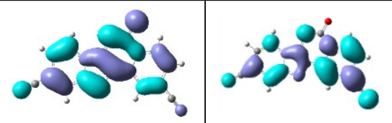	-6.22 eV	-3.70 eV
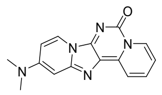 **DP12**	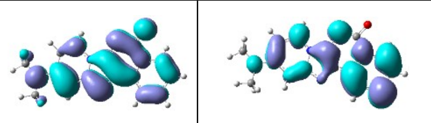	-4.90 eV	-2.10 eV	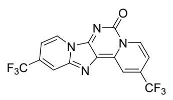 **DP37**	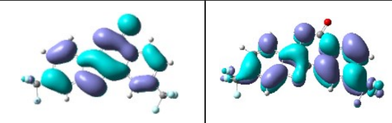	-6.06 eV	-3.26 eV
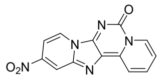 **DP13**	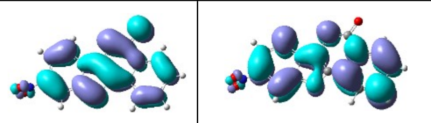	-5.85 eV	-2.86 eV	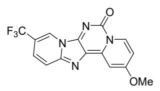 **DP38**	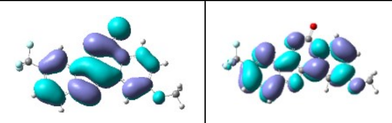	-5.57 eV	-2.40 eV
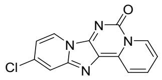 **DP14**	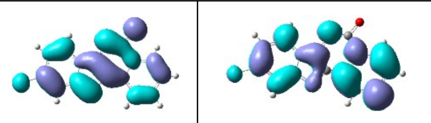	-5.57 eV	-2.60 eV	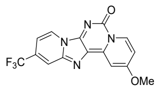 **DP39**	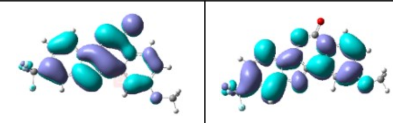	-5.58 eV	-2.46
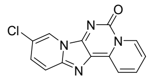 **DP15**	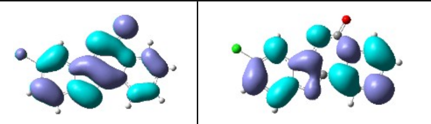	-5.59 eV	-2.62 eV	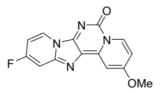 **DP40**	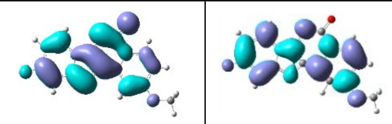	-5.35 eV	-2.19 eV
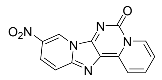 **DP16**	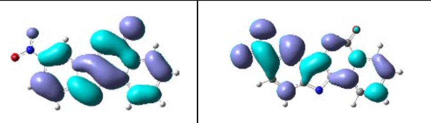	-5.95 eV	-3.14 eV	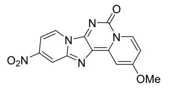 **DP41**	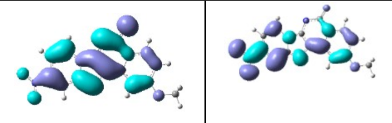	-5.80 eV	-3.20 eV
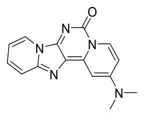 **DP17**	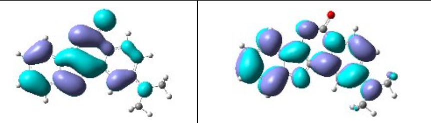	-5.00 eV	-1.83 eV	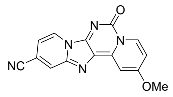 **DP42**	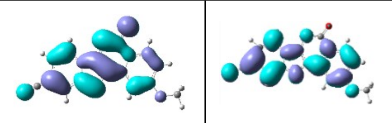	-5.67 eV	-2.71 eV
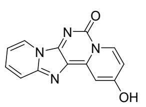 **DP18**	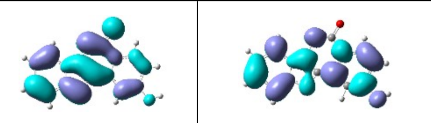	-5.32 eV	-2.19 eV	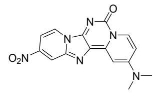 **DP43**	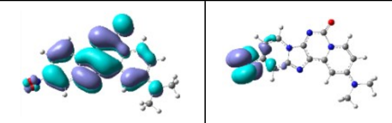	-5.42 eV	-2.59 eV
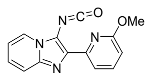 **DP19’**	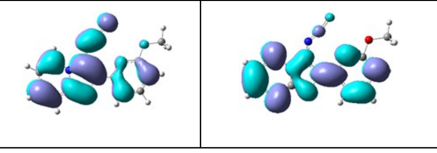			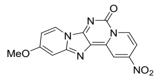 **DP44**	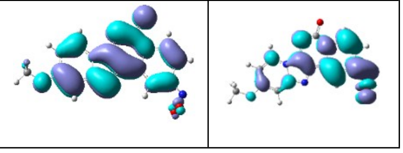		

	-5.65 eV	-1.69 eV		-5.45 eV	-3.06 eV
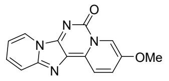 **DP20**	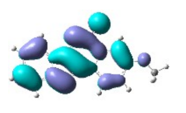	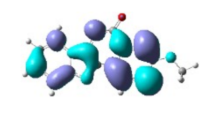	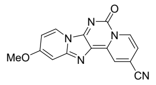 **DP45**	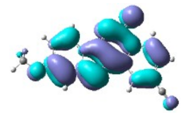	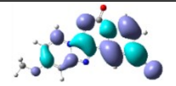
	-5.24 eV	-2.37 eV		-5.56 eV	-3.15 eV
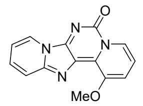 **DP21**	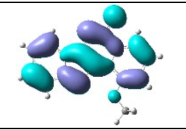	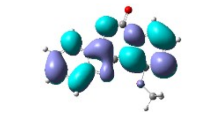	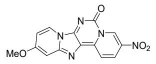 **DP46**	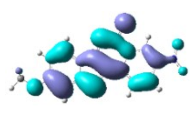	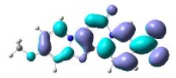
	-5.25 eV	-2.33 eV		-5.69 eV	-3.33 eV
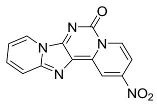 **DP22**	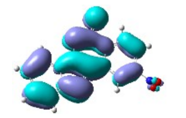	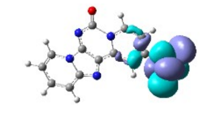	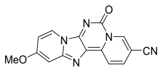 **DP47**	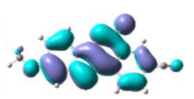	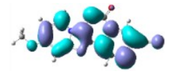
	-5.81 eV	-3.11 eV		-5.60 eV	-2.83 eV
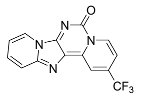 **DP23**	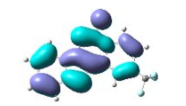	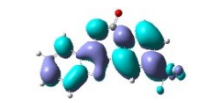	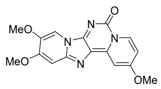 **DP48**	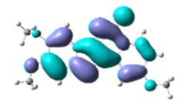	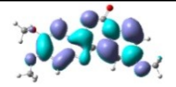
	-5.72 eV	-2.96 eV		-5.05 eV	-0.19 eV
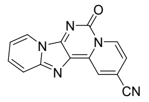 **DP24**	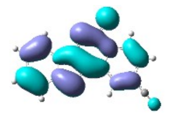	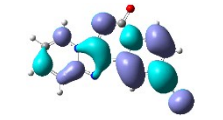	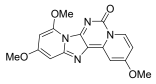 **DP49**	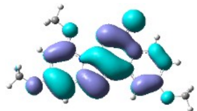	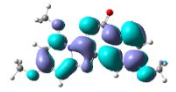
	-5.80	-3.29 eV		-4.77 eV	-1.72 eV
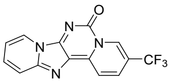 **DP25**	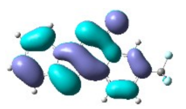	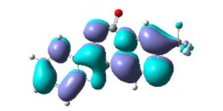	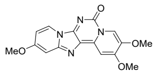 **DP50**	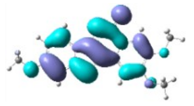	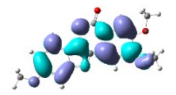
	-5.74 eV	-2.84 eV		-4.96 eV	-2.01 eV

Overview of the photophysical properties of DP1-DP5

Samples for electrochemical measurements were prepared with tetra-*n*-butylammonium hexafluorophosphate solution (0.1 M, 5 mL) in dry, degassed dichloromethane and substrate (1 mM). The CV was performed under nitrogen atmosphere in a one-compartment electrolysis cell consisting of a platinum wire working electrode, a platinum wire counter electrode, and reference saturated calomel electrode (SCE). CV were monitored at scan rates of either 50 mV s^{-1}.[5]

Figure S3. CV of **DP1** (1 mM) in DCM (SCE).

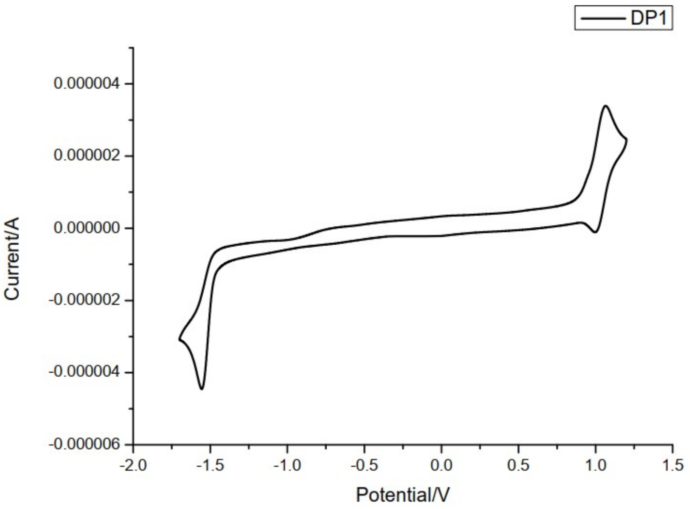

Figure S4. CV of **DP2** (1 mM) in DCM (SCE).

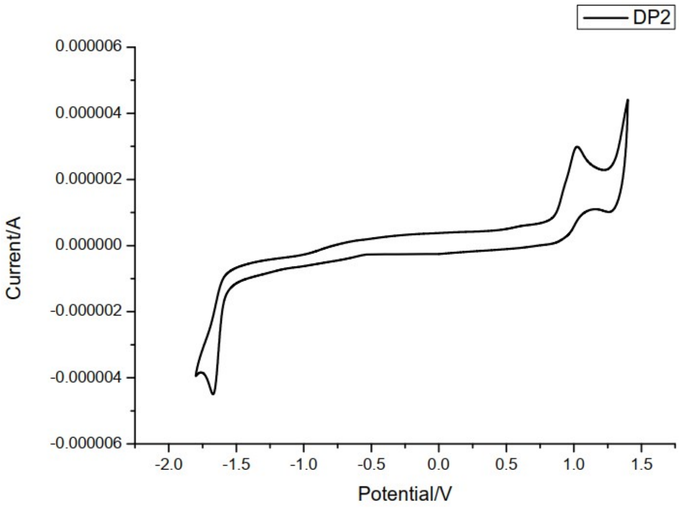

Figure S5. CV of **DP3** (1 mM) in DCM (SCE).

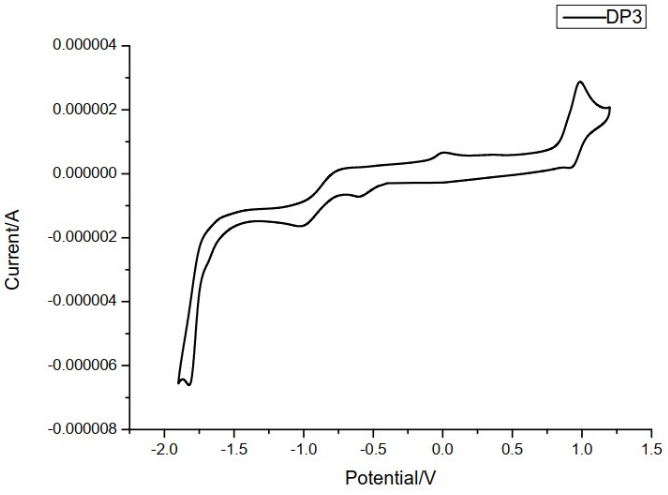

Figure S6. CV of **DP4** (1 mM) in DCM (SCE)

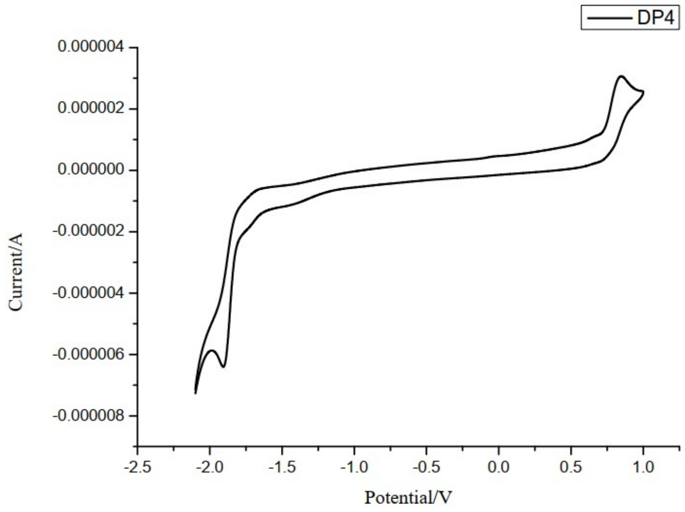

Figure S7. CV of **DP5** (1 mM) in DCM (SCE).

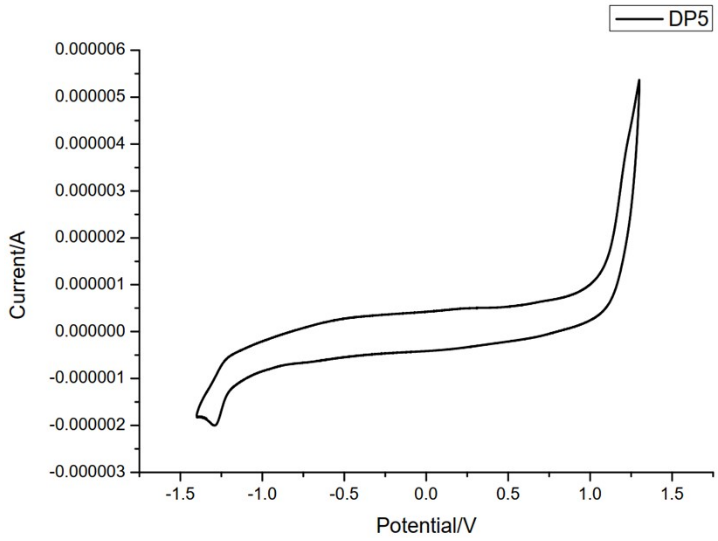

Figure S8. UV-vis absorption spectra of **DP1-DP5** (1 X 10^{-5} M) in DCM.

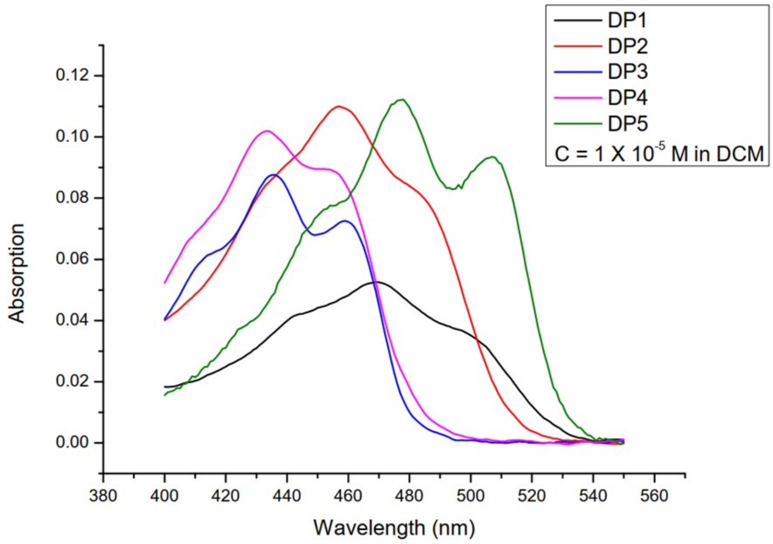

Figure S9. UV-vis absorption spectra of **DP1** (1 X 10^{-5} M) in DCM.

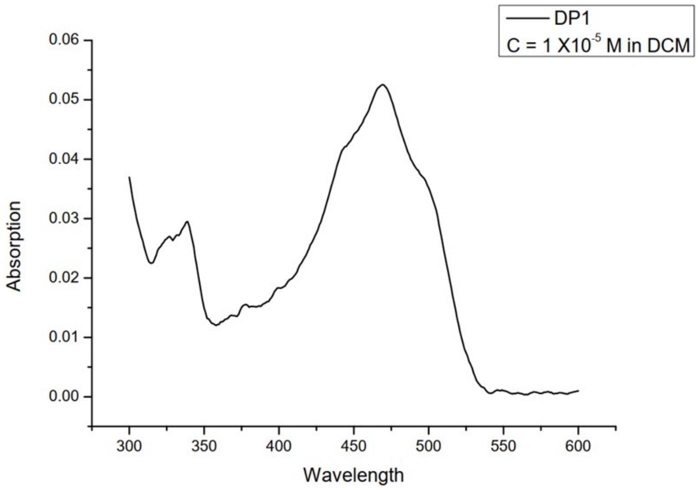

Figure S10. UV-vis absorption spectra of **DP2** (1 X 10^{-5} M) in DCM.

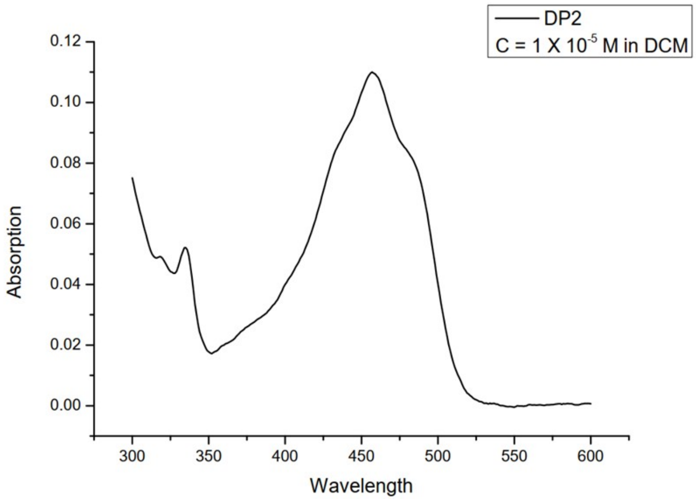

Figure S11. UV-vis absorption spectra of **DP3** (1 X 10^{-5} M) in DCM.

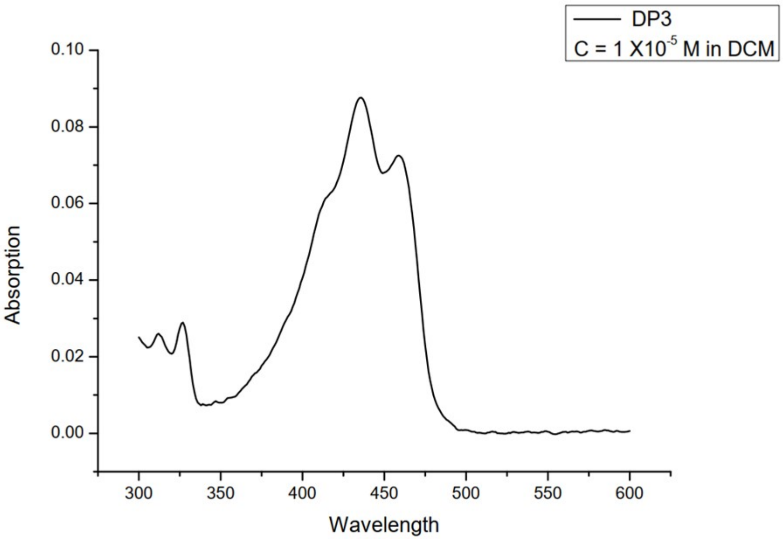

Figure S12. UV-vis absorption spectra of **DP4** (1 X 10^{-5} M) in DCM.

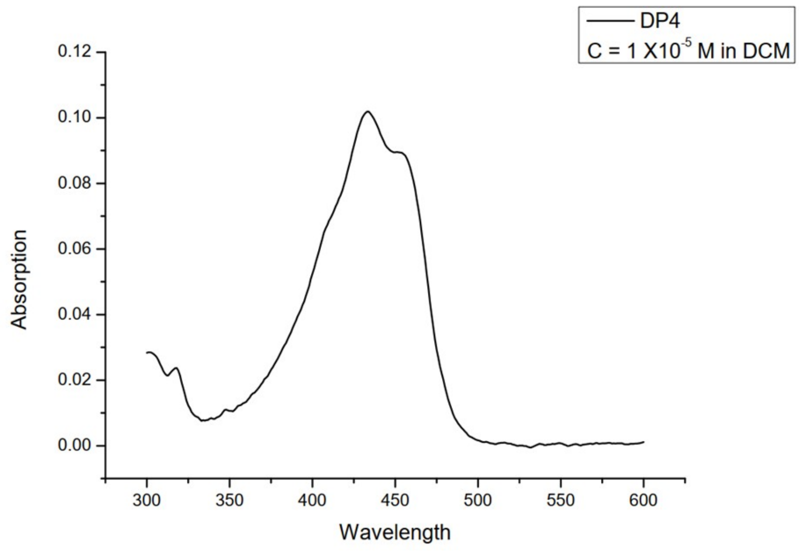

Figure S13. UV-vis absorption spectra of **DP5** (1 X 10^{-5} M) in DCM.

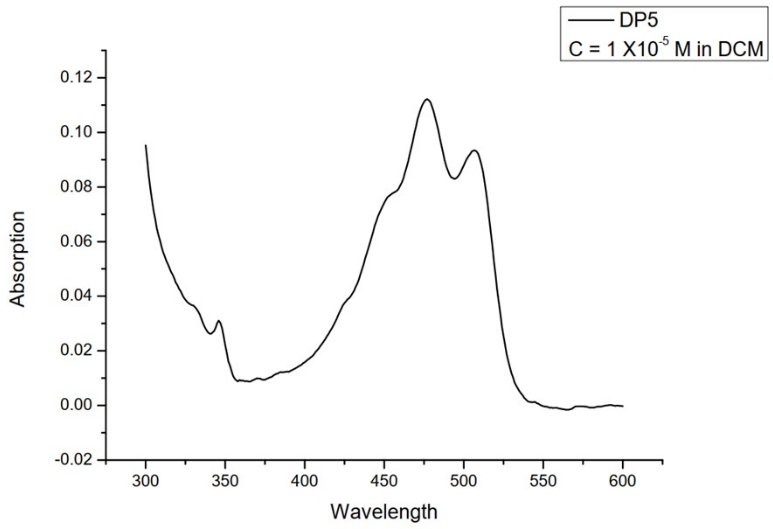

Figure S14. Excitation and emission absorption spectra of **DP1** in DCM.

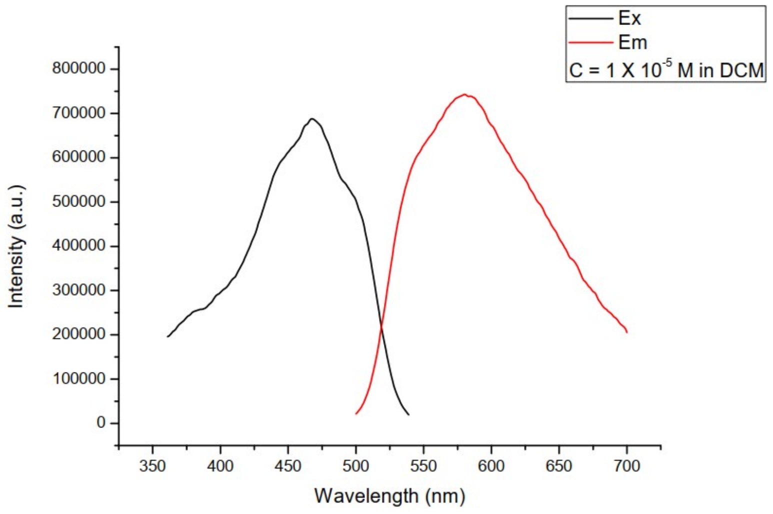

Figure S15. Excitation and emission absorption spectra of **DP2** in DCM.

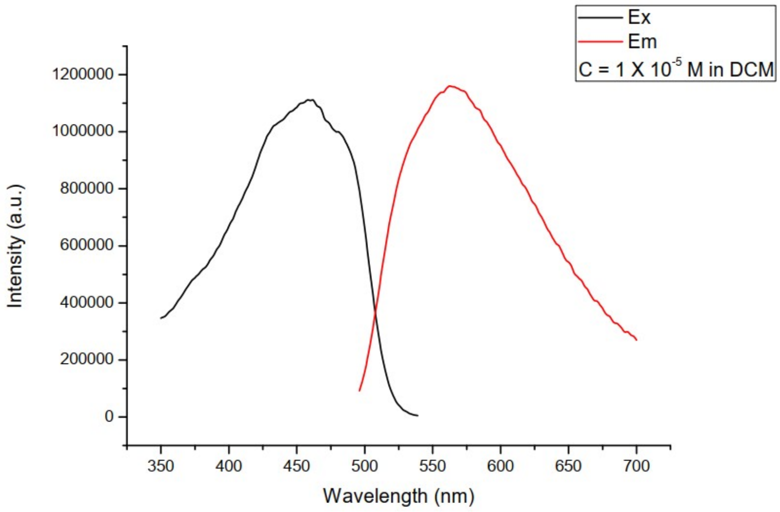

Figure S16. Excitation and emission absorption spectra of **DP3** in DCM.

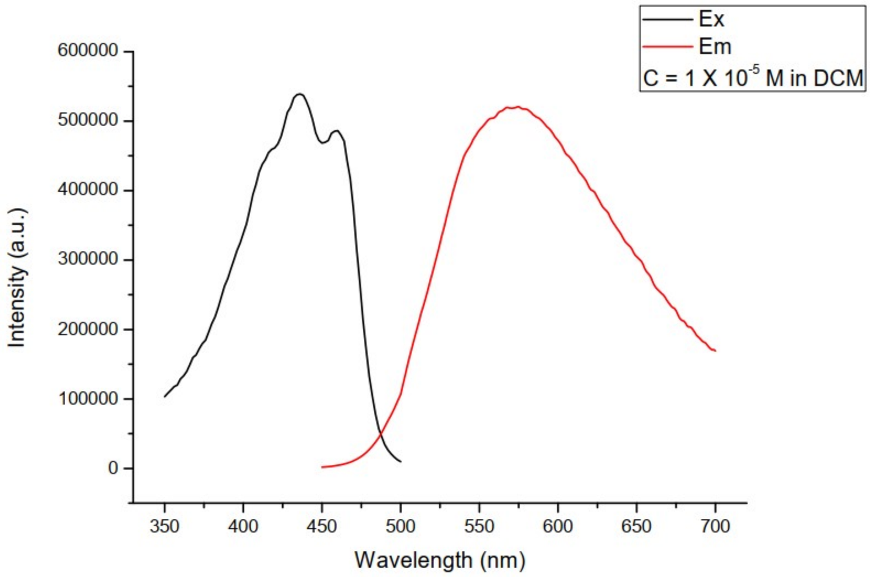

Figure S17. Excitation and emission absorption spectra of **DP4** in DCM.

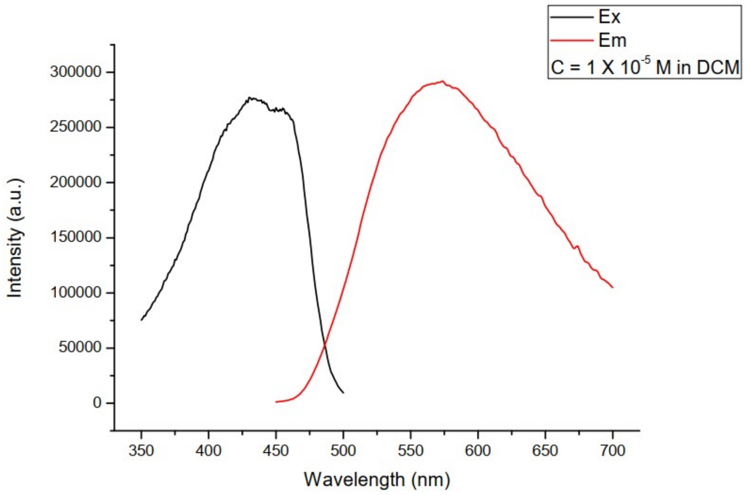

Figure S18. Excitation and emission absorption spectra of **DP5** in DCM.

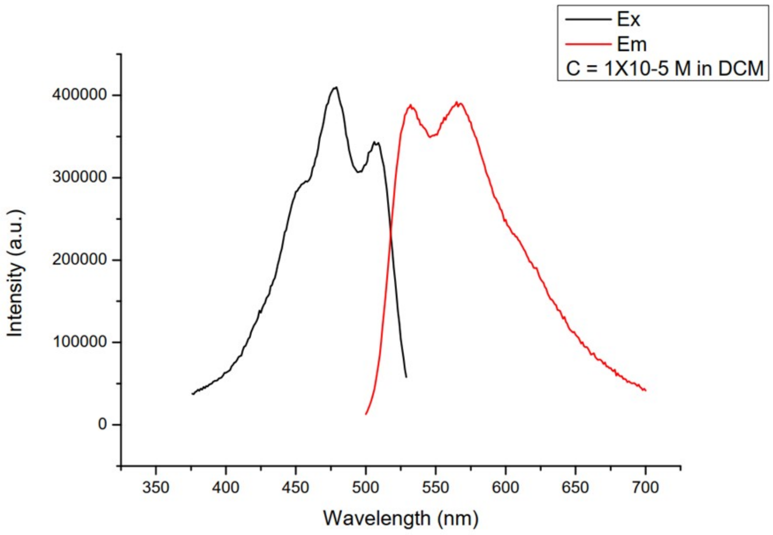

TableS3. Fluorescence Lifetime of **DP1-DP5**

	**DP1**	**DP2**	**DP3**	**DP4**	**DP5**
τ (ns)	2.14	2.13	0.93	0.36	5.13

Reaction conditions optimization

Firstly, the commercially available 1,3-diphenyl thiourea (**T1**) and morpholine (**A1**) were selected as a model substrate to screening the optimal conditions of the guanylation (Table S4). Initially, five photocatalysts were employed to the reaction. After many attempts, we found that compound *N*,*N'*-diphenylmorpholine-4-carboximidamide (**1**) could be isolated in an excellent yield (91%) in the presence of K_2CO_3 (2.0 equiv) in EtOH/H_2O (9/1, 3 mL) when **DP4** was used as the photocatalyst (Table S4, entries 1-5). Moreover, in the absence of catalyst, guanidine **1** were isolated in 8% yield (Table S4, entry 6) suggesting that the photoredox catalyst is essential for guanylation reaction. It was found that the yield of **1** could not be further improved when we increased the amount of **A1** with loading from 2.0 equiv to 4.0 equiv (Table S4, entry 7) or decreased the amount of **A1** form 2.0 equiv to 1.5 equiv (Table S4, entry 8). Experiments conducted under other atmospheres, including N_2 and O_2 respectively, did not give a higher yield of **1** (Table S4, entries 9-10). Other bases including $CsCO_3$, DBU, and triethylamine (TEA) were also screened and they were found to be not as efficient as K_2CO_3 (Table S4, entries 11-13). It was found that the conversion afforded product **1** in lower yields (12-82%) than that in

EtOH/H_2O (9/1) when the solvent was switched to EtOH, DMSO, DMSO/H_2O (9/1), PEG-400, PEG-400/H_2O (9/1), and MeCN, (Table S4, entries 14-19). It worth noting that we also tried to perform the transformation by using DBU base in MeCN solvent. As a result, we obtained compound **1** in 75% yield after 20 h (Table S4, entry 20). Finally, we identified the optimal reaction conditions as **T1** (0.3 mmol), **A1** (2.0 equiv), K_2CO_3 (2.0 equiv), and **DP4** (1 mol%) in EtOH/H_2O (9/1, 3 mL) at room temperature under air atomosphere irradiated by blue LED.

Table S4. Reaction optimization of guanylation.[a]

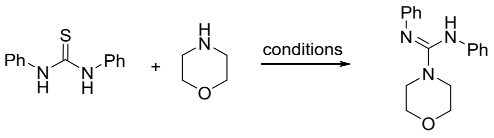

Entry	Solvent	Base	Catal.	Yield of **1**/%[b]
1	EtOH/H_2O	K_2CO_3	**DP1**	35
2	EtOH/H_2O	K_2CO_3	**DP2**	64
3	EtOH/H_2O	K_2CO_3	**DP3**	65
**4**	**EtOH/H_2O**	**K_2CO_3**	**DP4**	**91**
5	EtOH/H_2O	K_2CO_3	**DP5**	31
6	EtOH/H_2O	K_2CO_3	**-**	8
7	EtOH/H_2O	K_2CO_3	**DP4**	88[c]
8	EtOH/H_2O	K_2CO_3	**DP4**	79[d]
9	EtOH/H_2O	K_2CO_3	**DP4**	89[e]
10	EtOH/H_2O	K_2CO_3	**DP4**	20[f]
11	EtOH/H_2O	Cs_2CO_3	**DP4**	60
12	EtOH/H_2O	DBU	**DP4**	77
13	EtOH/H_2O	TEA	**DP4**	12
14	EtOH	K_2CO_3	**DP4**	21
15	DMSO	K_2CO_3	**DP4**	82
16	DMSO/ H_2O	K_2CO_3	**DP4**	30
17	PEG-400	K_2CO_3	**DP4**	12

18	PEG-400/H_2O	K_2CO_3	**DP4**	14
19	MeCN	K_2CO_3	**DP4**	25
20	MeCN	DBU	**DP4**	75

[a]Unless otherwise noted, the reaction was conducted with **T1** (0.3 mmol), **A1** (0.6 mmol), K_2CO_3 (0.6 mmol), **DP4** (1 mol%), in EtOH/H_2O (9/1, 3 mL), air atomosphere, r.t., blue LED, 20 h. [b]Isolated yields. [c]4.0 equuiv of **A1** was used. [d]1.5 equiv of **A1** was used. [e]Under O_2. [f]Under N_2.

Gram-scale preparation of Pinacidil

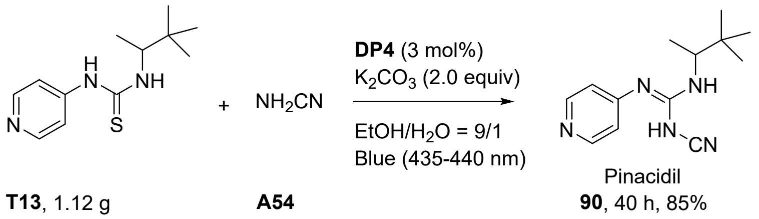

To demonstrate the productive practicability of this reaction, the gram-scale preparation of Pinacidil (**90**) was performed. It gave a satisfactory result. Dedailed opartion: a round-bottom flask (250 mL) was added with K_2CO_3 (10 mmol), **DP4** (3 mol%), **A54** (10 mmol), **T13** (5 mmol), and of ethanol/H_2O (9/1, 50 mL) and the reaction was stirred for 40 h (monitored by TLC) and placed away from the blue LED about 2.5 cm. The reaction mixture was treated with H_2O (50 mL), and extracted with DCM (3 × 50 mL). The combined organic layer was dried over anhydrous Na_2SO_4 (2.5 g), and concentrated by rotary evaporator. The residue was purified on silica gel using CH_2Cl_2/MeOH (20:1) as the eluent. The product **90** was obtained as a white solid (1.04 g, 85%).

Mechanistic studies

A pyrex glass tube equipped with a magnetic stirring bar was charged with DBU (0.6 mmol) and **DP4** (1 mol%), **T1** (0.3 mmol) and MeCN (3 mL) were added and reaction vessel was placed away from blue LED 2.5 cm. The reaction mixture was stirred at room temperature for 20 h under blue LED (monitored by TLC). After **T1** were completely consumed, a lead acetate aqueous solution (0.5 mmol) was added. The mixture was filtered,

washed and dried under vacuum. The precipitate was collected for SEM/EDX characterization. The morphological structure of particles was observed using a scanning electron microscope (SEM, ZEISS, EVO HD15, Figure S19). The elemental composition analysis of the material was investigated using a built-in energy dispersive X-ray spectrometer (EDS, OXFORD, X-Max20, Table S5).[6]

Figure S19. SEM and EDX images of $PbSO_4$ particles.

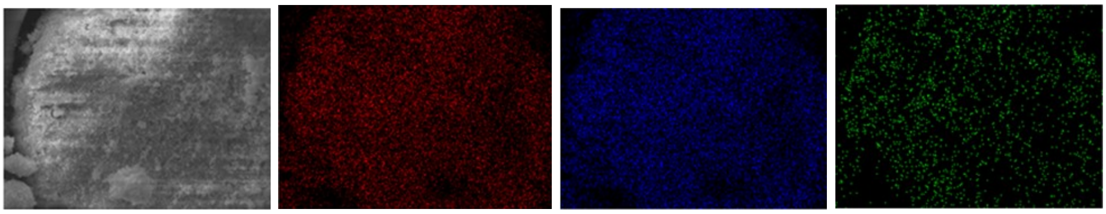

$PbSO_4$ particles Pb S O

Table S5. Quantitative analysis of $PbSO_4$ particles by SEM/EDX.

Formula	Mass%	Atom%
C	9.38	44.21
O	7.68	27.18
S	3.98	7.03
Pb	78.96	21.58
Total	100.00	100.00

Figure S20. Mass spectrum of diphenylmethanediimine

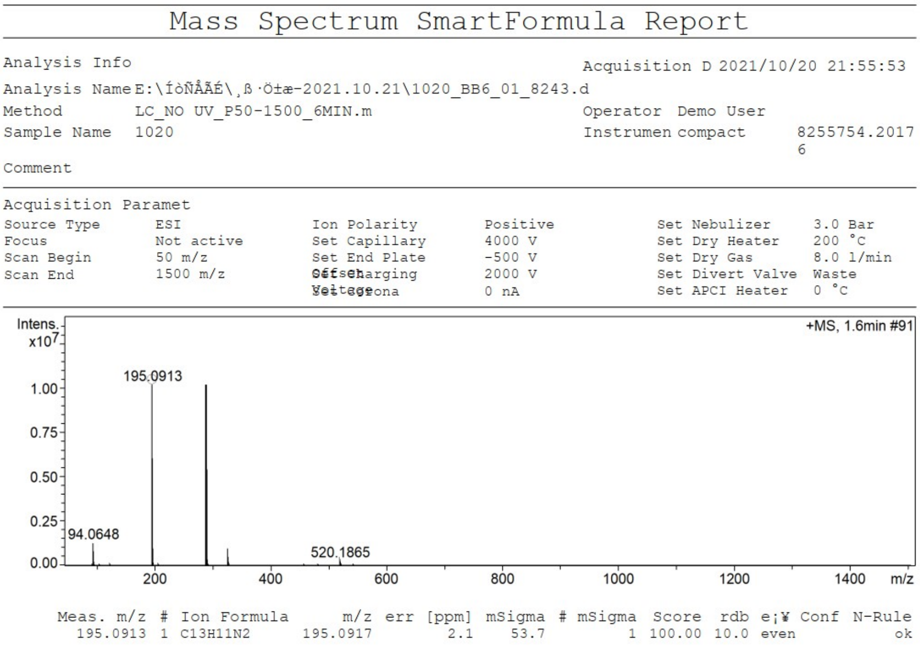

Control Experiments.

All the reactions were conducted under standard conditions with certain amount of additives.

Table S6. Control Experiments.

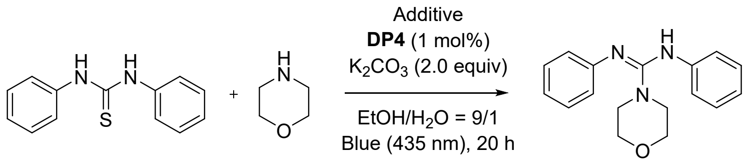

T1 **A1** **1**

Entries	Additives	Functions	Isolated yields of **1**/%
1	BQ (6 mol%)	$O_2^{\cdot -}$ inhibitor	89
2	1,3-diphenylisobenzofuran (1.0 equiv)	$O_2^{\cdot -}$ inhibitor	80
3	9,10-dimethylanthracene (1.0 equiv)	$^1O_2$ inhibitor	79
4	^{t}BuOH (2.0 equiv)	$^1O_2$ inhibitor	75

Characterization of compounds

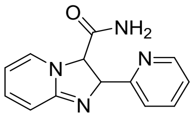

amide1

2-(Pyridin-2-yl)-2,3-dihydroimidazo[1,2-*a*]pyridine-3-carboxamide (amide1)

White solid. Mp: 135-137 °C. ^{1}H NMR (400 MHz, $CDCl_3$) δ 12.07 (s, 1H), 9.91 (d, J = 7.2 Hz, 1H), 8.58 (d, J = 4.4 Hz, 1H), 8.54 (d, J = 8.0 Hz, 1H), 7.90 (td, J = 7.8, 1.8 Hz, 1H), 7.68 (d, J = 8.8 Hz, 1H), 7.44-7.32 (m, 2H), 7.00 (t, J = 7.0 Hz, 1H), 5.88 (s, 1H). ^{13}C NMR (100 MHz, $CDCl_3$) δ 163.5, 153.1, 146.7, 146.0, 145.0, 137.8, 129.8, 127.7, 125.4, 123.6, 117.2, 116.4, 113.8. HRMS (ESI), *m/z* calcd. for $C_{13}H_{13}N_4O$ ($[M+H]^+$) 241.1084, found: 241.1080.

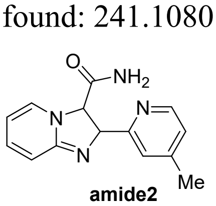

amide2

2-(4-Methylpyridin-2-yl)-2,3-dihydroimidazo[1,2-*a*]pyridine-3-carboxamide (amide2)

White solid. Mp: 119-120 °C. ^{1}H NMR (400 MHz, $CDCl_3$) δ 12.21 (s, 1H), 9.92 (d, J = 6.8 Hz, 1H), 8.45 (d, J = 4.8 Hz, 1H), 8.37 (s, 1H), 7.69 (d, J = 8.8 Hz, 1H), 7.39 (t, J = 7.8 Hz, 1H), 7.18 (d, J = 5.2 Hz, 1H), 6.97 (t, J = 7.0 Hz, 1H), 5.78 (s, 1H), 2.48 (s, 3H). ^{13}C NMR (100 MHz, $CDCl_3$) δ 163.6, 152.8, 149.3, 146.5, 146.0, 145.2, 129.8, 127.7, 125.9, 124.6, 117.1, 116.4, 113.8, 21.3. HRMS (ESI), *m/z* calcd. for $C_{14}H_{15}N_4O$ ($[M+H]^+$) 255.1240, found: 255.1245.

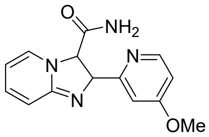

amide3

2-(4-Methoxypyridin-2-yl)-2,3-dihydroimidazo[1,2-*a*]pyridine-3-carboxamide (amide3)

White solid. Mp: 121-123 °C. ^{1}H NMR (600 MHz, $CDCl_3$) δ 12.35 (s, 1H), 9.93 (d, J = 7.2 Hz, 1H), 8.39 (d, J = 6.0 Hz, 1H), 8.09 (d, J = 2.4 Hz, 1H), 7.69 (d, J = 9.0 Hz, 1H), 7.42-7.37 (m, 1H), 6.98 (t, J = 6.9, 1H), 6.89 (dd, J = 6.0, 2.4 Hz, 1H), 5.75 (s, 1H), 4.00 (s, 3H). ^{13}C NMR (100 MHz, $CDCl_3$) δ 166.9, 163.6, 155.0, 147.9, 145.9, 145.1, 129.9, 127.6, 117.1, 113.8, 111.2, 109.7, 55.6. HRMS (ESI), *m/z* calcd. for $C_{14}H_{15}N_4O_2$ ($[M+H]^+$) 271.1190, found: 271.1189.

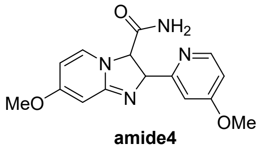

amide4

7-Methoxy-2-(4-methoxypyridin-2-yl)-2,3-dihydroimidazo[1,2-*a*]pyridine-3-carboxamide (amide4)

White solid. Mp: 128-131 °C. ^{1}H NMR (600 MHz, $CDCl_3$) δ 12.31 (s, 1H), 9.76 (d, J = 7.8 Hz, 1H), 8.38 (d, J = 5.4 Hz, 1H), 8.06 (s, 1H), 6.94 (s, 1H), 6.87 (d, J = 3.0 Hz, 1H), 6.71-6.63 (m, 1H), 5.68 (s, 1H), 3.98 (s, 3H), 3.90 (s, 3H). ^{13}C NMR (150 MHz, DMSO) δ 166.9, 163.6, 159.7, 155.0, 147.9, 147.8, 145.0, 130.3, 115.8, 111.0, 109.5, 108.3, 94.4, 55.6, 55.5. HRMS (ESI), *m/z* calcd. for $C_{15}H_{17}N_4O_3$ ($[M+H]^+$) 301.1295, found: 301.1293.

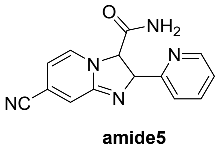

amide5

7-Cyano-2-(pyridin-2-yl)imidazo[1,2-a]pyridine-3-carboxamide (amide5)

White solid. Mp: 281-283 °C. ^{1}H NMR (600 MHz, $CDCl_3$) δ 12.28 (s, 1H), 10.07 (d, J = 7.2 Hz, 1H), 8.63 (d, J = 4.2 Hz, 1H), 8.59 (d, J = 8.4 Hz, 1H), 8.07 (s, 1H), 7.97 (t, J = 7.8 Hz, 1H), 7.44 (t, J = 6.0 Hz, 1H), 7.11 (d, J = 7.2 Hz, 1H), 5.86 (s, 1H). ^{13}C NMR (150 MHz, $CDCl_3$) δ 162.6, 152.2, 146.9, 146.9, 143.8, 138.2, 130.8, 125.6, 124.2, 123.1, 118.1, 117.1, 113.7, 110.5. HRMS (ESI), *m/z* calcd. for $C_{14}H_{10}N_5O$ ($[M+H]^+$) 264.0880, found: 264.0880.

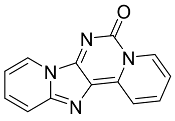

DP1

6*H*-Dipyrido[1,2-*e*:2',1'-*i*]purin-6-one (DP1)

Orange solid. Mp: 237-239 °C. ^{1}H NMR (600 MHz, $CDCl_3$) δ 9.55 (d, J = 7.2 Hz, 1H), 8.52 (d, J = 8.4 Hz, 1H), 8.48 (d, J = 7.2 Hz, 1H), 8.07 (t, J = 7.5 Hz, 1H), 7.52 (d, J = 9.6 Hz, 1H), 7.47 (t, J = 6.6 Hz, 1H), 7.32 (t, J = 7.8 Hz, 1H), 6.82 (t, J = 6.6 Hz, 1H). ^{13}C NMR (150 MHz, $CDCl_3$) δ 149.6, 146.5, 143.9, 143.2, 138.4, 133.3, 129.4, 124.3, 120.6, 118.3, 118.2, 117.8, 111.7. HRMS (ESI), *m/z* calcd. for $C_{13}H_9N_4O$ ($[M+H]^+$) 237.0771, found: 237.0769.

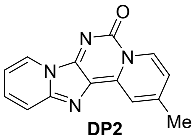

DP2

2-Methyl-6*H*-dipyrido[1,2-*e*:2',1'-*i*]purin-6-one (DP2)

Yellow solid. Mp: 276-278 °C. ^{1}H NMR (600 MHz, $CDCl_3$) δ 9.43 (d, J = 7.2 Hz, 1H), 8.48 (d, J = 6.6 Hz, 1H), 8.32 (s, 1H), 7.52 (d, J = 9.0 Hz, 1H), 7.36-7.27 (m, 2H), 6.82 (t, J = 6.6 Hz, 1H), 2.66 (s, 3H). ^{13}C NMR (150 MHz, $CDCl_3$) δ 151.8, 149.6, 146.1, 143.6, 143.0, 132.9, 129.0, 124.3, 120.6, 119.4, 118.1, 117.3, 111.7, 22.0. HRMS (ESI), *m/z* calcd. for $C_{14}H_{11}N_4O$ ($[M+H]^+$) 251.0927, found: 251.0922.

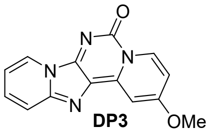

DP3

2-Methoxy-6*H*-dipyrido[1,2-*e*:2',1'-*i*]purin-6-one (DP3)

Yellow solid. Mp: 273-275 °C; ^{1}H NMR (600 MHz, $CDCl_3$) δ 9.41 (d, J = 7.8 Hz, 1H), 8.47 (d, J = 6.0 Hz, 1H), 7.72 (s, 1H), 7.52 (d, J = 9.0 Hz, 1H), 7.36-7.28 (m, 1H), 7.05-

7.00 (m, 1H), 6.82 (t, *J* = 6.3 Hz, 1H), 4.14 (s, 3H). ^{13}C NMR (151 MHz, $CDCl_3$) δ 167.1, 149.6, 145.8, 145.7, 135.5, 130.0, 128.8, 124.3, 118.0, 117.2, 111.7, 111.4, 98.2, 57.2. HRMS (ESI), *m/z* calcd. for $C_{14}H_{10}N_4O_2$ ([M+H]$^+$) 267.0877, found: 267.0875.

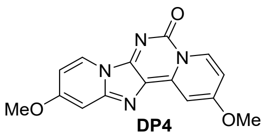

DP4

2,11-Dimethoxy-6*H*-dipyrido[1,2-*e*:2',1'-*i*]purin-6-one (DP4)

Yellow solid. Mp: 279-281 °C. ^{1}H NMR (400 MHz, $CDCl_3$) δ 9.33 (d, *J* = 8.0 Hz, 1H), 8.30 (d, *J* = 7.2 Hz, 1H), 7.60 (d, *J* = 2.8 Hz, 1H), 6.94 (dd, *J* = 8.0, 2.8 Hz, 1H), 6.71 (d, *J* = 2.0 Hz, 1H), 6.55 (dd, *J* = 7.6, 2.4 Hz, 1H), 4.12 (s, 3H), 3.91 (s, 3H). ^{13}C NMR (150 MHz, $CDCl_3$) δ 166.4, 161.0, 150.0, 147.7, 144.7, 135.0, 124.6, 110.9, 108.1, 97.6, 93.6, 57.1, 55.7. HRMS (ESI), *m/z* calcd. for $C_{15}H_{13}N_4O_3$ ([M+H]$^+$) 297.0982, found: 297.0982.

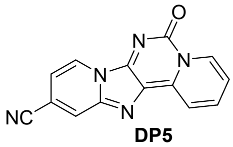

DP5

6-Oxo-6*H*-dipyrido[1,2-e:2',1'-i]purine-11-carbonitrile (DP5)

Orange solid. Mp: >300 °C. ^{1}H NMR (600 MHz, TFA) δ 9.85 (d, *J* = 6.6 Hz, 1H), 9.05 (d, *J* = 7.2 Hz, 1H), 8.89 (d, *J* = 7.8 Hz, 1H), 8.74 (t, *J* = 7.8 Hz, 1H), 8.57 (s, 1H), 8.12 (t, *J* = 6.6 Hz, 1H), 7.67 (dd, *J* = 7.2, 1.2 Hz, 1H). ^{13}C NMR (150 MHz, TFA_1) δ 148.5, 146.1, 140.3, 140.1, 136.6, 136.0, 126.7, 124.5, 122.4, 120.4, 118.4, 117.2, 117.1, 113.2, 111.50, 111.46. HRMS (ESI), *m/z* calcd. for $C_{14}H_8N_5O$ ([M+H]$^+$) 262.0723, found: 262.0725.

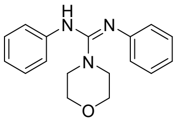

1

***N*,*N'*-Diphenylmorpholine-4-carboximidamide (1)[6]**

White solid. ^{1}H NMR (600 MHz, $CDCl_3$) δ 7.25 (t, *J* = 7.5 Hz, 4H), 7.08-6.80 (m, 6H), 5.51 (s, 1H), 6.82 (t, *J* = 4.8 Hz, 1H), 3.34 (t, *J* = 4.8 Hz, 1H). HRMS (ESI), *m/z* calcd. for $C_{17}H_{20}N_3O$ ([M+H]$^+$) 282.1601, found: 282.1599.

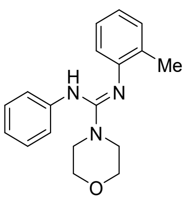

2

***N*-Phenyl-*N′*-(*o*-tolyl)morpholine-4-carboximidamide (2)**[6]

Yellow solid. ^{1}H NMR (400 MHz, DMSO) δ 7.62 (s, 1H), 7.10 (t, J = 7.2 Hz, 2H), 7.01 (d, J = 7.8 Hz, 1H), 6.97-6.81 (m, 3H), 6.78-6.57 (m, 3H), 3.64-3.51 (m, 4H), 3.28-3.19 (m, 4H), 2.09 (s, 3H). HRMS (ESI), *m/z* calcd. for $C_{18}H_{22}N_3O$ ([M+H]$^+$) 296.1757, found: 296.1758.

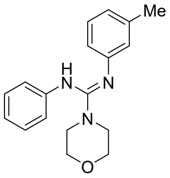

3

***N*-Phenyl-*N′*-(*m*-tolyl)morpholine-4-carboximidamide (3)**

Yellow solid. Mp: 33-36 ^{o}C. ^{1}H NMR (400 MHz, CDCl$_3$) δ 7.33-7.25 (m, 2H), 7.18 (t, J = 7.4 Hz, 1H), 7.07-6.64 (m, 6H), 5.56 (s, 1H), 3.75-3.69 (m, 4H), 3.42-3.33 (m, 4H), 2.33 (s, 3H). ^{13}C NMR (150 MHz, CDCl$_3$) δ 150.9, 139.2, 129.4, 129.4, 129.18, 129.16, 123.3, 122.5, 66.4, 47.0, 21.5. HRMS (ESI), *m/z* calcd. for $C_{18}H_{22}N_3O$ ([M+H]$^+$) 296.1757, found: 296.1756.

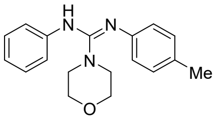

4

***N*-Phenyl-*N′*-(*p*-tolyl)morpholine-4-carboximidamide (4)**[6]

White solid. ^{1}H NMR (600 MHz, CDCl$_3$) δ 7.30-7.23 (m, 2H), 7.13-6.70 (m, 7H), 5.50 (s, 1H), 3.67 (dd, J = 10.4, 5.5 Hz, 4H), 3.38-3.28 (m, 4H), 2.28 (s, 3H). HRMS (ESI), *m/z* calcd. for $C_{18}H_{22}N_3O$ ([M+H]$^+$) 296.1757, found: 296.1754.

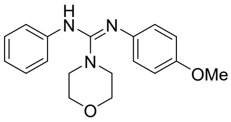

5

***N′*-(4-Methoxyphenyl)-*N*-phenylmorpholine-4-carboximidamide (5)**[6]

Yellow solid. ^{1}H NMR (600 MHz, $CDCl_3$) δ 7.28-7.22 (m, 2H), 7.07-6.72 (m, 7H), 5.49 (s, 1H), 3.77 (s, 3H), 3.70-3.58 (m, 4H), 3.35-3.28 (m, 4H). HRMS (ESI), *m/z* calcd. for $C_{18}H_{22}N_3O_2$ ($[M+H]^+$) 312.1707, found: 312.1702.

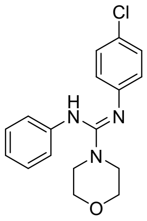

6

N′-(4-Chlorophenyl)-N-phenylmorpholine-4-carboximidamide (6)

Yellow solid. Mp: 39-42 °C. ^{1}H NMR (600 MHz, $CDCl_3$) δ 7.27 (t, J = 8.1 Hz, 2H), 7.20 (d, J = 9.0, 2H), 7.00 (t, J = 7.5 Hz, 1H), 6.94 (d, J = 5.4 Hz, 2H), 6.85 (d, J = 5.4 Hz, 2H), 3.67 (t, J = 4.8 Hz, 4H), 3.33 (t, J = 4.8 Hz, 4H). ^{13}C NMR (150 MHz, $CDCl_3$) δ 129.5, 129.4, 127.7, 122.9, 66.3, 47.1. HRMS (ESI), *m/z* calcd. for $C_{17}H_{19}ClN_3O$ ($[M+H]^+$) 316.1211, found: 316.1214.

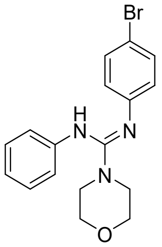

7

N′-(4-Bromophenyl)-N-phenylmorpholine-4-carboximidamide (7)[6]

Yellow solid. ^{1}H NMR (600 MHz, $CDCl_3$) δ 7.34 (d, J = 8.4 Hz, 2H), 7.27 (t, J = 7.5 Hz, 2H), 7.00 (t, J = 7.2 Hz, 1H), 6.95 (br, 2H), 6.79 (br, 2H), 5.40 (s, 1H), 3.68 (t, J = 4.8 Hz, 4H), 3.33 (t, J = 4.8 Hz, 4H). HRMS (ESI), *m/z* calcd. for $C_{17}H_{19}BrN_3O$ ($[M+H]^+$) 360.0706, found: 360.0707.

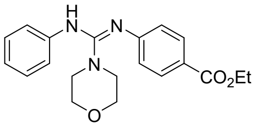

8

Ethyl-4-((morpholino(phenylamino)methylene)amino)benzoate (8)

White solid. Mp: 81-83 °C. ^{1}H NMR (400 MHz, $CDCl_3$) δ 7.92 (d, J = 8.4 Hz, 2H), 7.25 (t, J = 7.6 Hz, 2H), 7.04-6.85 (m, 5H), 4.33 (q, J = 7.2 Hz, 2H), 3.70 (t, J = 4.4 Hz, 4H), 3.36 (t, J = 4.4 Hz, 4H), 1.36 (t, J = 7.2 Hz, 3H). ^{13}C NMR (150 MHz, $CDCl_3$) δ 166.5, 131.2,

129.5, 129.4, 124.2, 122.9, 66.3, 60.7, 47.0, 14.4. HRMS (ESI), *m/z* calcd. for $C_{20}H_{24}N_3O_3$ ($[M+H]^+$) 354.1812, found: 354.1815.

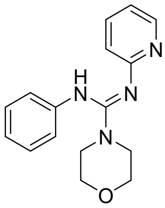

9

N-Phenyl-N′-(pyridin-2-yl)morpholine-4-carboximidamide (9)[6]

White solid. ^{1}H NMR (400 MHz, $CDCl_3$) δ 11.64 (s, 1H), 8.21 (dd, J = 5.2, 1.2 Hz, 1H), 7.60-7.50 (m, 1H), 7.33-7.26 (m, 2H), 7.15 (d, J = 7.2 Hz, 2H), 7.04-6.96 (m, 2H), 6.84-6.77 (m, 1H), 3.69 (t, J = 4.4 Hz, 4H), 3.44 (t, J = 4.4 Hz, 4H). HRMS (ESI), *m/z* calcd. for $C_{16}H_{19}N_4O$ ($[M+H]^+$) 283.1553, found: 283.1552.

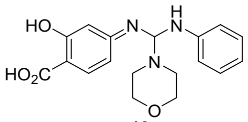

10

2-Hydroxy-5-((morpholino(phenylamino)methylene)amino)benzoic acid (10)

Orange solid. Mp: 255-258 °C. ^{1}H NMR (600 MHz, MeOD) δ 7.60 (d, J = 2.4 Hz, 1H), 7.32 (t, J = 7.8 Hz, 2H), 7.20-7.12 (m, 3H) 7.08 (dd, J = 8.4, 2.4 Hz, 1H), 6.73 (d, J = 8.4 Hz, 1H), 3.77-3.69 (m, 4H), 3.59-3.51 (m, 4H). ^{13}C NMR (151 MHz, MeOD) δ 159.9, 154.4, 137.1, 129.3, 127.2, 126.8, 125.7, 124.8, 122.0, 117.0, 65.4, 48.2. HRMS (ESI), *m/z* calcd. for $C_{18}H_{20}N_3O_3$ ($[M+H]^+$) 342.1448, found: 342.1444.

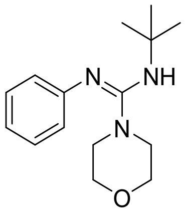

11

N-(Tert-butyl)-N′-phenylmorpholine-4-carboximidamide (11)

White solid. Mp: 109-111 °C; ^{1}H NMR (400 MHz, $CDCl_3$) δ 7.25 (t, J = 7.8 Hz, 1H), 6.93 (t, J = 7.4 Hz, 1H), 6.76 (d, J = 7.2 Hz, 2H), 3.69 (t, J = 4.6 Hz, 4H), 3.60 (s, 1H), 3.24-3.20 (t, J = 4.6 Hz, 4H), 1.20 (s, 9H). ^{13}C NMR (100 MHz, $CDCl_3$) δ 156.4, 150.2, 129.1, 122.2, 121.7, 66.7, 52.7, 49.5, 30.3. HRMS (ESI), *m/z* calcd. for $C_{15}H_{24}N_3O$ ($[M+H]^+$) 362.1914, found: 362.1913.

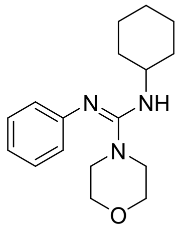

12

2-Cyclohexyl-1-morpholino-*N*-phenylethan-1-imine (12)

White solid. Mp: 70-73 °C. ^{1}H NMR (400 MHz, $CDCl_3$) δ 7.30 (t, J = 7.8 Hz, 2H), 7.00 (t, J = 7.2 Hz, 1H), 6.84 (d, J = 7.6 Hz, 2H), 3.78 (t, J = 4.6 Hz, 4H), 3.48 (s, 1H), 3.35-3.28 (t, J = 4.6 Hz, 4H), 3.15-3.01 (m,1H), 1.98-1.84 (m, 2H), 1.76-1.50 (m, 3H), 1.39-1.18 (m, 2H), 1.13-1.02 (m, 1H), 1.00-0.82 (m, 2H). ^{13}C NMR (150 MHz, $CDCl_3$) δ 156.0, 150.0, 129.2, 122.7, 122.0, 66.8, 53.5, 48.7, 34.0, 25.4, 25.2. HRMS (ESI), *m/z* calcd. for $C_{17}H_{26}N_3O$ ([M+H]$^+$) 288.2070, found: 288.2070.

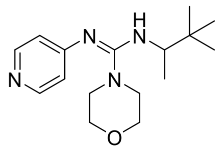

13

***N*-(3,3-Dimethylbutan-2-yl)-*N*'-(pyridin-4-yl)morpholine-4-carboximidamide (13)**

White solid. Mp: 152-156 °C. ^{1}H NMR (600 MHz, $CDCl_3$) δ 8.29 (d, J = 6.0 Hz, 2H), 6.70 (d, J = 6.0 Hz, 2H), 3.68-3.63 (m, 4H), 3.41 (q, J = 6.6 Hz, 1H), 3.26-3.17 (m, 4H), 1.04 (d, J = 6.6 Hz, 3H), 0.83 (s, 9H). ^{13}C NMR (150 MHz, $CDCl_3$) δ 158.2, 156.0, 149.5, 117.0, 66.4, 57.1, 47.9, 35.0 26.2, 16.0. HRMS (ESI), *m/z* calcd. for $C_{16}H_{27}N_4O$ ([M+H]$^+$) 291.2179, found: 291.2175.

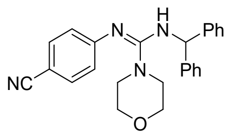

14

***N*-Benzhydryl-*N′*-(4-cyanophenyl)morpholine-4-carboximidamide (14)**

White solid. Mp: 142-145 °C. ^{1}H NMR (400 MHz, $CDCl_3$) δ 7.37-7.26 (m, 8H), 7.15 (d, J = 6.8 Hz, 4H), 6.43 (d, J = 7.2 Hz, 2H), 5.65 (s, 1H), 4.08 (s, 1H), 3.78-3.68 (m, 4H), 3.33-3.18 (m, 4H). ^{13}C NMR (150 MHz, $CDCl_3$) δ 154.7, 154.2, 141.6, 133.2, 128.9, 127.8, 127.1, 122.8, 119.8, 104.1, 66.6, 62.4, 48.1. HRMS (ESI), *m/z* calcd. for $C_{25}H_{25}N_4O$ ([M+H]$^+$) 397.2023, found: 397.2016.

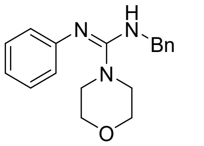

15

***N*-Benzyl-*N′*-phenylmorpholine-4-carboximidamide (15)**

White solid. Mp: 30-31 °C. ^{1}H NMR (600 MHz, $CDCl_3$) δ 7.31 (t, J = 7.2 Hz, 2H), 7.27 (t, J = 6.6 Hz, 1H), 7.24-7.17 (m, 4H), 6.95 (t, J = 7.2 Hz, 1H), 6.73 (d, J = 7.2 Hz, 2H), 4.24 (s, 2H), 3.72 (t, J = 4.2 Hz, 4H), 3.26 (t, J = 4.2 Hz, 4H). ^{13}C NMR (150 MHz, $CDCl_3$) δ 156.2, 138.6, 129.3, 128.8, 127.6, 127.4, 122.5, 122.2, 66.6, 49.3, 48.3. HRMS (ESI), *m/z* calcd. for $C_{18}H_{22}N_3O$ ($[M+H]^+$) 296.1757, found: 296.1755.

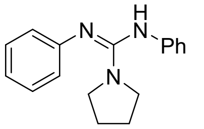

17

***N,N′*-Diphenylpyrrolidine-1-carboximidamide (17)[6]**

White solid. ^{1}H NMR (600 MHz, $CDCl_3$) δ 7.23 (t, J = 7.5 Hz, 4H), 6.95 (t, J = 7.2 Hz, 2H), 6.89 (d, J = 7.8 Hz, 4H), 5.59 (s, 1H), 3.40-3.35 (m, 4H), 1.89-1.79 (m, 4H). HRMS (ESI), *m/z* calcd. for $C_{17}H_{20}N_3$ ($[M+H]^+$) 266.1652, found: 266.1647.

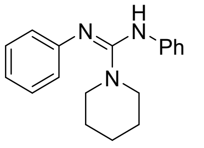

18

***N,N′*-Diphenylpiperidine-1-carboximidamide (18)**

White solid. Mp: 78-80 °C. ^{1}H NMR (600 MHz, $CDCl_3$) δ 7.23 (t, J = 7.8 Hz, 4H), 6.99-6.85 (m, 6H), 5.45 (s, 1H), 3.43-3.21 (m, 4H), 1.69-1.48 (m, 6H). ^{13}C NMR (150 MHz, $CDCl_3$) δ 151.2, 129.2, 122.0, 47.6, 25.4, 24.7. HRMS (ESI), *m/z* calcd. for $C_{13}H_9N_4O$ ($[M+H]^+$) 280.1808, found: 280.1806. HRMS (ESI), *m/z* calcd. for $C_{18}H_{22}N_3$ ($[M+H]^+$) 280.1808, found: 280.1806.

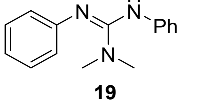

19

1,1-Dimethyl-2,3-diphenylguanidine (19)

White solid. Mp: 29-31 °C. ^{1}H NMR (400 MHz, $CDCl_3$) δ 7.30 (t, J = 7.8 Hz, 4H), 6.95 (t, J = 8.0 Hz, 2H), 6.86 (d, J = 7.8 Hz, 4H), 5.39 (d, J = 75.6 Hz, 1H), 2.90 (s, 6H). ^{13}C NMR (100 MHz, $CDCl_3$) δ 151.6, 129.3, 122.0, 38.2. HRMS (ESI), m/z calcd. for $C_{15}H_{18}N_3$ ($[M+H]^+$) 240.1495, found: 240.1496.

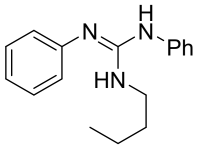

20

1-Butyl-2,3-diphenylguanidine (20)[6]

White solid. ^{1}H NMR (600 MHz, $CDCl_3$) δ 7.33-7.27 (m, 4H), 7.10-6.98 (m, 6H), 5.68 (s, 1H), 4.04 (s, 1H), 3.33 (t, J = 7.2 Hz, 2H), 1.59-1.47 (m, 2H), 1.41-1.32 (m, 2H), 0.94 (t, J = 7.5 Hz, 3H). HRMS (ESI), m/z calcd. for $C_{17}H_{22}N_3$ ($[M+H]^+$) 268.1808, found: 268.1808.

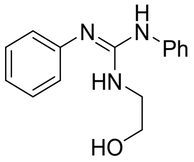

21

1-(2-Hydroxyethyl)-2,3-diphenylguanidine (21)[6]

White solid. ^{1}H NMR (600 MHz, DMSO) δ 8.33 (s, 1H), 7.35 (t, J = 7.8 Hz, 4H), 7.25 (d, J = 7.8 Hz, 4H), 7.15 (t, J = 7.2 Hz, 2H), 3.59 (t, J = 4.8 Hz, 2H), 3.45 (t, J = 4.8 Hz, 2H). HRMS (ESI), m/z calcd. for $C_{15}H_{18}N_3O$ ($[M+H]^+$) 256.1444, found: 256.1444.

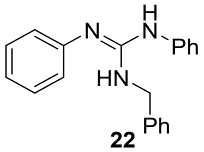

22

1-Benzyl-2,3-diphenylguanidine (22)[6]

White solid. ^{1}H NMR (400 MHz, $CDCl_3$) δ 7.38-7.24 (m, 9H), 7.11-7.98 (m, 6H), 4.54 (s, 2H). HRMS (ESI), m/z calcd. for $C_{20}H_{20}N_3$ ($[M+H]^+$) 302.1652, found: 302.1652.

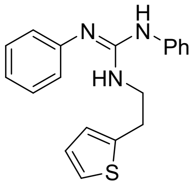

23

1,2-Diphenyl-3-(2-(thiophen-2-yl)ethyl)guanidine (23)

White solid. Mp: 62-63 °C. ^1H NMR (400 MHz, $CDCl_3$) δ 7.26 (t, J = 7.8 Hz, 4H), 7.14 (dd, J = 5.0, 1.2 Hz, 1H), 7.02 (t, J = 7.4 Hz, 2H), 6.99-6.89 (m, 5H), 6.80 (d, J = 2.8 Hz, 1H), 5.65 (s, 1H), 4.25 (s, 1H), 3.62 (t, J = 6.4 Hz, 2H), 3.13 (t, J = 6.4 Hz, 2H). ^{13}C NMR (150 MHz, $CDCl_3$) δ 147.9, 141.9, 129.5, 127.0, 125.5, 1239, 123.3, 42.9, 29.8. HRMS (ESI), m/z calcd. for $C_{19}H_{20}N_3S$ ($[M+H]^+$) 322.1372, found: 322.1372.

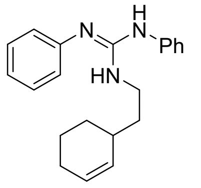

24

1-(2-(Cyclohex-2-en-1-yl)ethyl)-2,3-diphenylguanidine (24)

White solid. Mp: 70-72 °C. ^1H NMR (400 MHz, $CDCl_3$) δ 7.34-7.22 (m, 4H), 7.10-7.98(m, 6H), 5.67 (s, 1H), 5.36 (s, 1H), 4.17 (s, 1H), 3.39 (t, J = 6.4 Hz, 2H), 2.18 (t, J = 6.4 Hz, 2H), 1.96-1.84 (m, 4H), 1.69-1.55 (m, 2H), 1.54-1.42 (m, 2H). ^{13}C NMR (100 MHz, $CDCl_3$) δ 148.2, 134.8, 129.4, 124.2, 123.3, 38.9, 37.5, 27.5, 25.2, 22.8, 22.4. HRMS (ESI), m/z calcd. for $C_{21}H_{26}N_3$ ($[M+H]^+$) 320.2121, found: 320.2122.

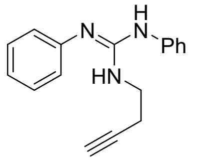

25

1-(But-3-yn-1-yl)-2,3-diphenylguanidine (25)

Yellow solid. Mp: 69-71 °C. ^1H NMR (600 MHz, $CDCl_3$) δ 7.27-7.24 (m, 4H), 7.12-7.07 (m, 6H), 3.51 (t, J = 6.3 Hz, 2H), 2.51 (td, J = 6.0, 2.4 Hz, 2H), 2.02 (t, J = 2.4 Hz, 1H). ^{13}C NMR (100 MHz, $CDCl_3$) δ 147.8, 129.5, 123.3, 82.4, 69.9, 40.1, 19.4. HRMS (ESI), m/z calcd. for $C_{17}H_{18}N_3$ ($[M+H]^+$) 264.1495, found: 264.1495.

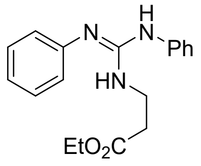

26

Ethyl-3-(2,3-diphenylguanidino)propanoate (26)[6]

White solid. ^1H NMR (600 MHz, $CDCl_3$) δ 7.29 (t, J = 7.5 Hz, 4H), 7.11-6.97 (m, 6H), 5.85 (s, 1H), 4.59 (s, 1H), 4.13 (q, J = 7.2 Hz, 2H), 3.63 (t, J = 5.7 Hz, 2H), 2.66 (t, J = 5.7

Hz, 2H), 1.23 (t, J = 7.2 Hz, 3H). HRMS (ESI), *m/z* calcd. for $C_{18}H_{22}N_3O_2$ ([M+H]$^+$) 312.1707, found: 312.1708.

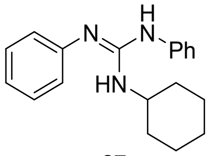

27

1-Cyclohexyl-2,3-diphenylguanidine (27)[7]

White solid. ^{1}H NMR (600 MHz, $CDCl_3$) δ 7.28 (t, J = 7.8 Hz, 4H), 7.10-6.96 (m, 6H), 5.64 (s, 1H), 3.96 (s, 1H), 3.75 (s, 1H), 2.08 (d, J = 9.6 Hz, 2H), 1.71-1.63 (m, 2H), 1.62-1.53 (m, 1H), 1.43-1.32 (m, 2H), 1.18-1.05 (m, 3H). HRMS (ESI), *m/z* calcd. for $C_{19}H_{24}N_3$ ([M+H]$^+$) 294.1965, found: 294.1965.

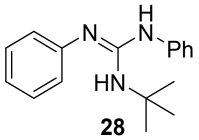

28

1-(Tert-butyl)-2,3-diphenylguanidine (28)

Yellow solid. Mp: 87-90 °C. ^{1}H NMR (400 MHz, $CDCl_3$) δ 7.30-7.23(m, 4H), 7.12-6.88 (m, 6H), 5.55 (s, 1H), 3.95 (s, 1H), 1.43 (s, 9H). ^{13}C NMR (100 MHz, $CDCl_3$) δ 129.4, 123.1, 121.9, 51.2, 29.6. HRMS (ESI), *m/z* calcd. for $C_{17}H_{22}N_3$ ([M+H]$^+$) 268.1808, found: 268.1807.

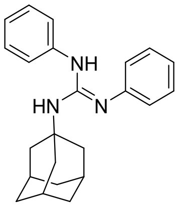

29

1-((3s,5s,7s)-Adamantan-1-yl)-2,3-diphenylguanidine (29)

Yellow solid. Mp: 75-77 °C. ^{1}H NMR (600 MHz, $CDCl_3$) δ 7.32-7.24 (m, 3H), 7.16-6.86 (m, 7H), 5.58 (s, 1H), 3.86 (s, 1H), 2.13-1.99 (m, 11H), 1.72-1.65 (m, 7H). ^{13}C NMR (150 MHz, $CDCl_3$) δ 129.3, 123.1, 121.7, 51.9, 42.7, 36.5, 29.7. HRMS (ESI), *m/z* calcd. for $C_{23}H_{28}N_3$ ([M+H]$^+$) 246.2278, found: 246.2278.

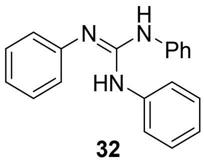

32

1,2,3-Triphenylguanidine (32)[8]

White solid. ^{1}H NMR (600 MHz, CDCl$_3$) δ 7.32 (t, J = 8.4 Hz, 6H), 7.28-7.15 (m, 6H), 7.11-7.02 (m, 3H), 5.93 (s, 2H). HRMS (ESI), m/z calcd. for $C_{19}H_{18}N_3$ ([M+H]$^+$) 288.1495, found: 288.1498.

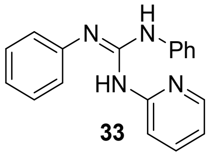

33

1,2-Diphenyl-3-(pyridin-2-yl)guanidine (33)

White solid. Mp: 95-98 °C. ^{1}H NMR (600 MHz, CDCl$_3$) δ 7.36-7.30 (m, 7H), 7.26-7.14 (m, 4H), 7.10-7.00 (m, 4H), 5.91 (s, 2H). ^{13}C NMR (150 MHz, CDCl$_3$) δ 146.2, 129.4, 123.7, 121.8. HRMS (ESI), m/z calcd. for $C_{18}H_{16}N_4$ ([M]$^+$) 288.1375, found: 288.1380.

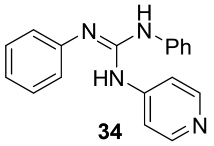

34

1,2-Diphenyl-3-(pyridin-4-yl)guanidine (34)

White solid. Mp: 82-85 °C. ^{1}H NMR (600 MHz, CDCl$_3$) δ 7.33-7.27 (m, 6H), 7.25-7.15 (m, 5H), 7.07-7.00 (m, 3H). ^{13}C NMR (150 MHz, CDCl$_3$) δ 145.0, 129.4, 123.2, 121.6. HRMS (ESI), m/z calcd. for $C_{18}H_{16}N_4$ ([M]$^+$) 288.1375, found: 288.1376.

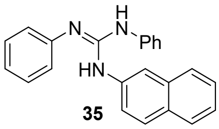

35

1-(Naphthalen-2-yl)-2,3-diphenylguanidine (35)

Yellow solid. Mp: 89-91 °C. ^{1}H NMR (600 MHz, CDCl$_3$) δ 7.78 (dd, J = 8.1, 3.9 Hz, 2H), 7.73 (d, J = 8.4 Hz, 1H), 7.67 (s, 1H), 7.48-7.29 (m, 8H), 7.27-7.20 (m, 3H), 7.07 (t, J = 7.2 Hz, 2H). ^{13}C NMR (150 MHz, CDCl$_3$) δ 145.1, 134.3, 130.2, 129.4, 129.2, 127.6, 127.1, 126.4, 124.5, 123.4, 122.4, 121.6. HRMS (ESI), m/z calcd. for $C_{23}H_{20}N_3$ ([M+H]$^+$) 338.1652, found: 338.1652.

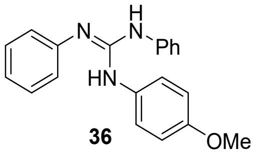

36

1-(4-Methoxyphenyl)-2,3-diphenylguanidine (36)

Brown solid. Mp: 109-110 °C. ^{1}H NMR (600 MHz, $CDCl_3$) δ 7.30 (t, J = 7.8 Hz, 4H), 7.25-7.16 (m, 4H), 7.14 (d, J = 7.8 Hz, 2H), 7.03 (t, J = 7.2 Hz, 2H), 6.89-6.85 (m, 2H), 3.79 (s, 3H). ^{13}C NMR (100 MHz, $CDCl_3$) δ 145.6, 129.3, 122.9, 121.5, 114.8, 55.6. HRMS (ESI), *m/z* calcd. for $C_{20}H_{20}N_3O$ ($[M+H]^+$) 318.1601, found: 318.1602.

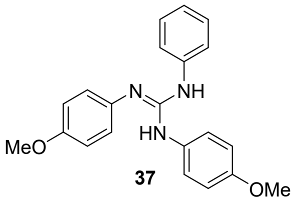

37

1,2-Bis(4-methoxyphenyl)-3-phenylguanidine (37)

Brown solid. Mp: 61-63 °C. ^{1}H NMR (400 MHz, $CDCl_3$) δ 7.29 (t, J = 7.8 Hz, 2H), 7.26-7.07 (m, 6H), 7.01 (t, J = 7.4 Hz, 1H), 6.89-6.83 (m, 4H), 5.85 (s, 1H), 3.79 (s, 6H). ^{13}C NMR (100 MHz, $CDCl_3$) δ 146.2, 129.3, 114.7, 55.6. HRMS (ESI), *m/z* calcd. for $C_{21}H_{22}N_3O_2$ ($[M+H]^+$) 348.1707, found: 348.1709.

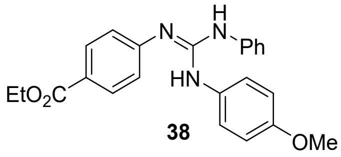

38

Ethyl-4-((((4-methoxyphenyl)amino)(phenylamino)methylene)amino)benzoate (38)

Black solid. Mp: 47-49 °C. ^{1}H NMR (400 MHz, $CDCl_3$) δ 7.91 (d, J = 8.8 Hz, 2H), 7.31-7.26 (m, 2H), 7.23-7.13 (m, 8.1 Hz, 4H), 7.11-7.03 (m, 3H), 6.85-6.81 (m, 2H), 4.32 (q, J = 7.2 Hz, 2H), 3.77 (s, 3H), 1.36 (t, J = 7.2 Hz, 3H). ^{13}C NMR (100 MHz, $CDCl_3$) δ 180.1, 166.4, 157.2, 146.8, 130.9, 129.4, 124.9, 124.7, 124.0, 121.8, 120.6, 114.9, 60.7, 55.5, 14.4. HRMS (ESI), *m/z* calcd. for $C_{23}H_{24}N_3O_3$ ($[M+H]^+$) 390.1812, found: 390.1811.

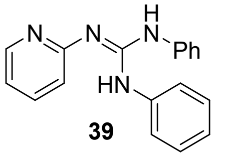

39

1,3-Diphenyl-2-(pyridin-2-yl)guanidine (39)

White solid. Mp: 144-145 °C. ^{1}H NMR (400 MHz, MeOD) δ 8.21 (dd, J = 5.2, 2 Hz, 1H), 7.65-7.57 (m, 1H), 7.34-7.23 (m, 8H), 7.06-6.98 (m, 2H), 6.94-6.87 (m, 2H). ^{13}C NMR (100 MHz, MeOD) δ 145.9, 138.0, 128.7, 122.9, 121.6, 116.7. HRMS (ESI), *m/z* calcd. for $C_{18}H_{17}N_4$([M+H]$^+$) 289.1448, found: 289.1447.

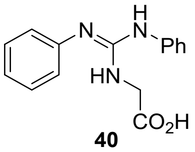

40

***N,N′*-Diphenylcarbamimidoyl)glycine (40)[9]**

White solid. ^{1}H NMR (400 MHz, MeOD) δ 7.44-7.31 (m, 8H), 7.29-7.22 (m, 2H), 3.88 (s, 2H). HRMS (ESI), *m/z* calcd. for $C_{15}H_{16}N_3O_2$ ([M+H]$^+$) 270.1237, found: 270.1237.

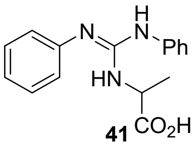

41

(*N,N′*-Diphenylcarbamimidoyl)alanine (41)

White solid. Mp: 194-196 °C. ^{1}H NMR (600 MHz, DMSO) δ 8.34 (s, 1H), 7.24 (t, J = 8.1 Hz, 4H), 7.11 (d, J = 7.2 Hz, 4H), 7.02 (t, J = 7.2 Hz, 2H), 4.05 (q, J = 7.2Hz, 1H), 1.32 (d, J = 7.2 Hz, 3H). ^{13}C NMR (150 MHz, DMSO) δ 174.5, 152.5, 129.6, 124.5, 122.0, 54.0, 17.6. HRMS (ESI), *m/z* calcd. for $C_{16}H_{18}N_3O_2$([M+H]$^+$) 284.1394, found: 284.1393.

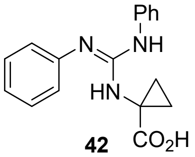

42

1-(2,3-Diphenylguanidino)cyclopropane-1-carboxylic acid (42)

White solid. Mp: 160-162 °C. ^{1}H NMR (600 MHz, TFA) δ 7.70-7.58 (m, 6H), 7.55-7.43 (m, 4H), 2.20-210 (m, 2H), 1.86-1.77 (m, 2H). ^{13}C NMR (150 MHz, TFA) δ 177.1, 156.1, 131.4, 130.5, 123.0, 126.6, 33.8, 18.8. HRMS (ESI), *m/z* calcd. for $C_{17}H_{18}N_3O_2$([M+H]$^+$) 296.1394, found: 296.1394.

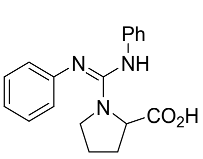

43

(*N,N′*-Diphenylcarbamimidoyl)proline (43)

White solid. Mp: 149-151 °C. ^{1}H NMR (400 MHz, MeOD) *δ* 7.26-6.98 (m, 10H), 4.61 (dd, *J* = 7.4, 5.8 Hz, 1H), 3.58 (t, *J* = 6.8 Hz, 2H), 2.37-2.16 (m, 2H), 2.04-1.87 (m, 2H). ^{13}C NMR (150 MHz, CD_3OD) *δ* 175.3, 1523, 137.1, 128.8, 127.8 125.0, 122.1, 121.8, 64.5, 49.7, 29.9, 24.2. HRMS (ESI), *m/z* calcd. for $C_{18}H_{20}N_3O_2$([M+H]$^+$) 310.1550, found: 310.1549.

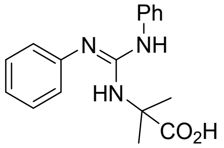

44

2-(2,3-Diphenylguanidino)-2-methylpropanoic acid (44)

White solid. Mp: 159-162 °C. ^{1}H NMR (400 MHz, MeOD) *δ* 7.30-7.23 (m, 4H), 7.17-7.05 (m, 6H), 1.60 (s, 6H). ^{13}C NMR (100 MHz, MeOD) *δ* 179.3, 152.7, 136.3 129.2, 125.3, 121.8, 61.3, 25.2. HRMS (ESI), *m/z* calcd. for $C_{17}H_{20}N_3O_2$([M+H]$^+$) 298.1550, found: 298.1538.

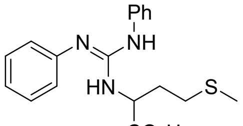

45

(*N*,*N'*-Diphenylcarbamimidoyl)methionine (45)

Orange solid. Mp: 101-103 °C. ^{1}H NMR (600 MHz, MeOD) *δ* 7.35 (t, *J* = 8.1 Hz, 4H), 7.26 (d, *J* = 7.8 Hz, 4H), 7.20 (t, *J* = 7.5 Hz, 2H), 4.29 (dd, *J* = 8.4, 4.8 Hz, 1H), 2.64-2.55 (m, 2H), 2.30-2.20 (m, 1H), 2.15-2.07 (m, 4H). ^{13}C NMR (150 MHz, MeOD) *δ* 175.2, 153.9, 135.9, 129.4, 126.2, 123.5, 57.2, 30.3, 29.8, 13.8. HRMS (ESI), *m/z* calcd. for $C_{18}H_{22}N_3O_2S$([M+H]$^+$) 344.1427, found: 344.1428.

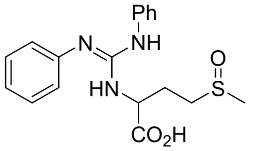

45'

2-(2,3-Diphenylguanidino)-4-(methylsulfinyl)butanoic acid (45')

Orange solid. Mp: 114-116 °C. ^{1}H NMR (600 MHz, MeOD) *δ* 7.36 (t, *J* = 7.5 Hz, 4H), 7.33-7.26 (m, 4H), 7.21 (t, *J* = 7.2 Hz, 2H), 4.38-4.24 (m, 1H), 3.03-2.79 (m, 2H), 2.64 (s, 3H), 2.45-2.38 (m, 1H), 2.32-2.21 (m, 1H). ^{13}C NMR (150 MHz, MeOD) *δ* 174.1, 153.6,

135.8, 129.4, 126.4, 123.8, 123.7, 56.7, 49.4, 37.0, 25.1. HRMS (ESI), *m/z* calcd. for $C_{18}H_{22}N_3O_3S$([M+H]$^+$) 360.1376, found: 360.1377.

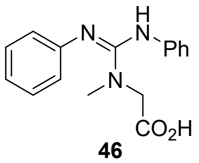

46

***N*-(*N*,*N′*-Diphenylcarbamimidoyl)-N-methylglycine (46)**

White solid. Mp: 161-163 °C. ^{1}H NMR (400 MHz, MeOD) δ 7.21 (t, J = 8.0 Hz, 4H), 7.12 (d, J = 7.6 Hz, 4H), 7.05 (t, J = 7.4 Hz, 2H), 4.08 (s, 2H), 3.11 (s, 3H). ^{13}C NMR (150 MHz, MeOD) δ 172.7, 155.0, 137.0, 129.0, 125.2, 121.7, 55.9, 38.2. HRMS (ESI), *m/z* calcd. for $C_{16}H_{18}N_3O_2$ ([M+H]$^+$) 284.1394, found: 284.1404.

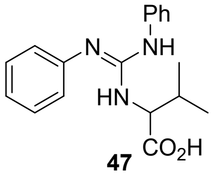

47

(*N*,*N′*-Diphenylcarbamimidoyl)valine (47)

White solid. Mp: 116-118 °C. ^{1}H NMR (400 MHz, TFA) δ 7.50-7.39 (m, 6H), 7.28 (d, J = 7.2 Hz, 4H), 4.42 (d, J = 4.4 Hz, 1H), 2.48-2.32 (m, 1H), 1.03 (d, J = 6.8 Hz, 3H), 0.89 (d, J = 6.8 Hz, 3H). ^{13}C NMR (150 MHz, TFA) δ 175.1, 154.9, 131.6, 130.5, 129.7, 125.8, 60.3, 30.8, 17.2, 15.4. HRMS (ESI), *m/z* calcd. for $C_{18}H_{22}N_3O_2$ ([M+H]$^+$) 312.1707, found: 312.1705.

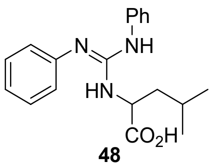

48

(*N*,*N′*-Diphenylcarbamimidoyl)leucine (48)

White solid. Mp: 113-116 °C. ^{1}H NMR (400 MHz, MeOD) δ 7.43-7.30 (m, 4H), 7.28-7.14 (m, 6H), 4.13 (t, J = 7.0 Hz, 1H), 1.83-1.72 (m, 3H), 1.02-0.92 (m 6H). ^{13}C NMR (150 MHz, MeOD) δ 176.6, 153.8, 136.1, 129.3, 125.9, 123.0, 57.5, 40.1, 24.8, 22.2, 20.7. HRMS (ESI), *m/z* calcd. for $C_{19}H_{24}N_3O_2$ ([M+H]$^+$) 326.1863, found: 326.1864.

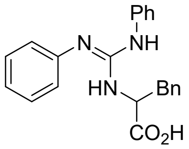

49

(N,N'-Diphenylcarbamimidoyl)phenylalanine (49)

White solid. Mp: 135-137 °C. ^{1}H NMR (600 MHz, TFA) δ 7.41-7.29 (m, 9H), 7.10 (d, J = 7.2 Hz, 2H), 7.02 (d, J = 3.0 Hz, 4H), 4.86 (dd, J = 8.4, 4.8 Hz, 1H), 3.38 (dd, J = 14.4, 4.8 Hz, 1H), 3.15 (dd, J = 14.4, 8.4 Hz, 1H). ^{13}C NMR (150 MHz, TFA) δ 174.8, 154.4, 133.4, 131.3, 130.3, 129.5, 129.5, 128.6, 128.3, 125.7, 56.0, 37.1. HRMS (ESI), *m/z* calcd. for $C_{22}H_{22}N_3O_2$ ([M+H]$^+$) 360.1707, found: 360.1707.

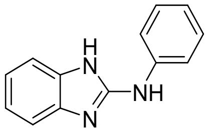

50

***N*-Phenyl-1*H*-benzo[*d*]imidazol-2-amine (50)**[10]

Yellow solid. ^{1}H NMR (400 MHz, DMSO) δ 10.88 (s, 1H), 9.39 (s, 1H), 7.75 (d, J = 7.6 Hz, 2H), 7.37-7.26 (m, 4H), 7.04-6.96 (m, 2H), 6.92 (t, J = 7.4 Hz, 1H). HRMS (ESI), *m/z* calcd. for $C_{13}H_{12}N_3$ ([M+H]$^+$) 210.1026, found: 210.1027.

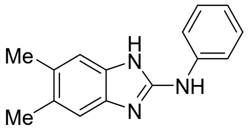

51

5,6-Dimethyl-*N*-phenyl-1*H*-benzo[*d*]imidazol-2-amine (51)[10]

Yellow solid. ^{1}H NMR (600 MHz, DMSO) δ 10.72 (s, 1H), 9.28 (s, 1H), 7.72 (d, J = 7.8 Hz, 2H), 7.29 (t, J = 8.1 Hz, 2H), 7.10 (s, 2H), 6.90 (t, J = 7.2 Hz, 1H), 2.25 (s, 6H). HRMS (ESI), *m/z* calcd. for $C_{15}H_{16}N_3$ ([M+H]$^+$) 238.1339, found: 238.1337.

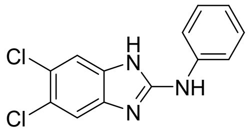

52

5,6-Dichloro-*N*-phenyl-1*H*-benzo[*d*]imidazol-2-amine (52)[11]

Yellow solid. ^{1}H NMR (600 MHz, DMSO) δ 11.11 (s, 1H), 9.68 (s, 1H), 7.74 (d, J = 7.8 Hz, 2H), 7.51 (s, 2H), 7.33 (t, J = 7.8 Hz, 2H), 6.96 (t, J = 7.2 Hz, 1H). HRMS (ESI), *m/z* calcd. for $C_{13}H_{10}Cl_2N_3$ ([M+H]$^+$) 278.0246, found: 278.0246.

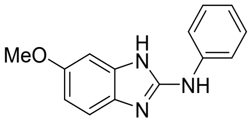

53

6-Methoxy-*N*-phenyl-1*H*-benzo[*d*]imidazol-2-amine (53)[10]

Yellow solid. ^{1}H NMR (400 MHz, $CDCl_3$) δ 8.83 (s, 2H), 7.31 (d, *J* = 7.6 Hz, 2H), 7.23 (t, *J* = 7.8 Hz, 2H), 7.15 (d, *J* = 8.4 Hz, 1H), 6.99 (t, *J* = 7.4 Hz, 1H), 6.82 (d, *J* = 1.6 Hz, 1H), 6.71 (dd, *J* = 8.6, 2.2 Hz, 1H), 3.75 (s, 3H). HRMS (ESI), *m/z* calcd. for $C_{14}H_{14}N_3O$ ($[M+H]^+$) 240.1131, found: 240.1130.

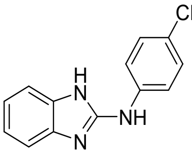

54

4-((1*H*-Benzo[*d*]imidazol-2-yl)amino)benzonitrile (54) [12]

Yellow solid. ^{1}H NMR (600 MHz, DMSO) δ 11.21 (s, 1H), 10.10 (s, 1H), 7.97 (d, *J* = 9.0 Hz, 2H), 7.76 (d, *J* = 9.0 Hz, 2H), 7.48-7.29 (m, 2H), 7.05 (dd, *J* = 5.4, 3.0 Hz, 2H). HRMS (ESI), *m/z* calcd. for $C_{14}H_{11}N_4$ ($[M+H]^+$) 235.0978, found: 235.0978.

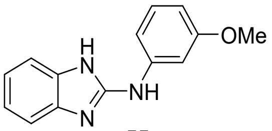

55

***N*-(3-Methoxyphenyl)-1*H*-benzo[*d*]imidazol-2-amine (55)**[13]

Yellow solid. ^{1}H NMR (600 MHz, $CDCl_3$) δ 7.32-7.27 (m, 2H), 7.19 (t, *J* = 8.1 Hz, 1H), 7.10-7.06 (m, 2H), 6.93 (d, *J* = 7.8, 1H), 6.90 (t, *J* = 2.2 Hz, 1H), 6.59 (dd, *J* = 8.4, 2.4 Hz, 1H), 3.70 (s, 3H). HRMS (ESI), *m/z* calcd. for $C_{14}H_{14}N_3O$ ($[M+H]^+$) 240.1131, found: 240.1131.

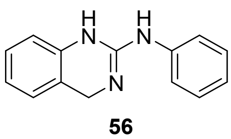

56

***N*-Phenyl-1,4-dihydroquinazolin-2-amine (56)**[14]

Yellow solid. ^{1}H NMR (600 MHz, $CDCl_3$) δ 7.34 (t, *J* = 7.8 Hz, 2H), 7.15-7.09 (m, 4H), 6.98 (d, *J* = 7.2 Hz, 1H), 6.92 (t, *J* = 7.2 Hz, 1H), 6.72 (d, *J* = 7.8 Hz, 1H), 4.39 (s, 2H). HRMS (ESI), *m/z* calcd. for $C_{14}H_{14}N_3$ ($[M+H]^+$) 224.1182, found: 224.1182.

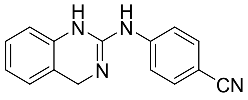

57

4-((1,4-Dihydroquinazolin-2-yl)amino)benzonitrile (57)

Yellow solid. Mp: 239-241 °C. ^{1}H NMR (600 MHz, $CDCl_3$) δ 7.60 (d, J = 8.4 Hz, 2H), 7.17 (t, J = 7.5z, 1H), 7.10 (d, J = 8.4 Hz, 2H), 7.05 (d, J = 7.8 Hz, 1H), 6.97 (t, J = 7.5 Hz, 1H), 6.66 (d, J = 7.8 Hz, 1H), 4.42 (s, 2H). ^{13}C NMR (100 MHz, $CDCl_3$) δ 152.7, 148.37, 136.47, 133.87, 128.47, 125.8, 124.1, 122.2, 119.6, 118.5, 114.4, 105.2, 43.0. HRMS (ESI), *m/z* calcd. for $C_{15}H_{13}N_4$ ([M+H]$^+$) 249.1135, found: 249.1130.

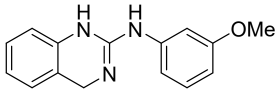

58

***N*-(3-Methoxyphenyl)-1,4-dihydroquinazolin-2-amine (58)**

Yellow solid. Mp: 171-173 °C. ^{1}H NMR (400 MHz, $CDCl_3$) δ 7.28-7.24 (m, 1H), 7.10 (t, J = 7.6 Hz, 1H), 6.99 (d, J = 7.6 Hz, 1H), 6.90 (t, J = 7.4 Hz, 1H), 6.76-6.61 (m, 4H), 6.13 (s, 2H), 4.38 (s, 2H), 3.78 (s, 3H). ^{13}C NMR (100 MHz, $CDCl_3$) δ 160.9, 149.6, 137.0, 130.4, 128.3, 125.6, 121.9, 118.5 116.2, 115., 109.8, 109.5, 55.3, 42.9. HRMS (ESI), *m/z* calcd. for $C_{15}H_{16}N_3O$ ([M+H]$^+$) 254.1288, found: 254.1289.

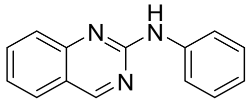

59

***N*-Phenylquinazolin-2-amine (59)**[15]

Yellow solid. ^{1}H NMR (600 MHz, $CDCl_3$) δ 9.09 (s, 1H), 7.85 (d, J = 7.8 Hz, 2H), 7.78-7.72 (m, 3H), 7.55 (s, 1H), 7.39 (t, J = 8.1 Hz, 2H), 7.36-7.31 (m, 1H), 7.09 (t, J = 7.5 Hz, 1H). HRMS (ESI), *m/z* calcd. for $C_{14}H_{12}N_3$ ([M+H]$^+$) 222.1026, found: 222.1025.

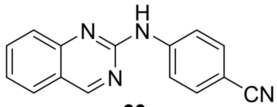

60

4-(Quinazolin-2-ylamino)benzonitrile (60)

Yellow solid. Mp: 224-225 °C. ^{1}H NMR (600 MHz, $CDCl_3$) δ 9.15 (s, 1H), 8.00 (d, J = 8.4 Hz, 2H), 7.85-7.79 (m, 3H), 7.66 (d, J = 9.0 Hz, 2H), 7.60 (s, 1H), 7.47-7.41(m, 1H). ^{13}C

NMR (150 MHz, $CDCl_3$) δ 162.1, 143.7, 134.8, 133.3, 127.5, 126.6, 124.9, 121.3, 119.5, 118.4, 104.7. HRMS (ESI), *m/z* calcd. for $C_{15}H_{11}N_4$ ($[M+H]^+$) 247.0978, found: 247.0976.

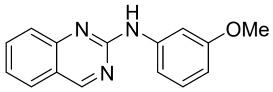

61

_N_-(3-Methoxyphenyl)quinazolin-2-amine (61)

Yellow solid. Mp: 124-127 ^oC. ^1H NMR (400 MHz, $CDCl_3$) δ 9.09 (s, 1H), 7.83-7.68 (m, 4H), 7.41-7.32 (m, 2H), 7.30-7.20 (m, 2H), 6.63 (d, J = 7.2 Hz, 1H), 3.88 (s, 3H). ^{13}C NMR (100 MHz, $CDCl_3$) δ 161.8, 160.3, 156.7, 151.5, 140.9, 134.4, 129.6, 127.4, 126.4, 123.9, 120.9, 111.4, 108.0, 105.0, 55.3. HRMS (ESI), *m/z* calcd. for $C_{15}H_{14}N_3O$ ($[M+H]^+$) 252.1131, found: 252.1130.

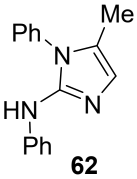

62

5-Methyl-_N_-1-diphenyl-1_H_-imidazol-2-amine (62)[16]

Yellow solid. ^1H NMR (600 MHz, $CDCl_3$) δ 7.54 (t, J = 7.5 Hz, 2H), 7.49 (t, J = 7.2 Hz, 1H), 7.30 (d, J = 8.4 Hz, 4H), 7.22 (t, J = 7.8 Hz, 2H), 6.88 (t, J = 7.5 Hz, 1H), 6.68 (s, 1H), 5.64 (s, 1H), 2.01 (s, 3H). HRMS (ESI), *m/z* calcd. for $C_{16}H_{16}N_3$ ($[M+H]^+$) 250.1339, found: 250.1339.

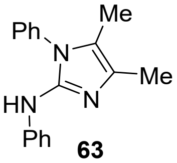

63

4,5-Dimethyl-_N_-1-diphenyl-1_H_-imidazol-2-amine (63)

Colorless oil. ^1H NMR (400 MHz, $CDCl_3$) δ 7.54-7.41 (m, 3H), 7.29-7.16 (m, 6H), 6.89-6.80 (m, 1H), 5.59 (s, 1H), 2.21 (s, 3H), 1.94 (s, 3H). ^{13}C NMR (100 MHz, $CDCl_3$) δ 142.2, 141.8, 135.5, 129.9, 129.7, 129.0, 128.8, 127.9, 120.4, 118.9, 116.1, 12.8, 9.4. HRMS (ESI), *m/z* calcd. for $C_{17}H_{18}N_3$ ($[M+H]^+$) 264.1495, found: 264.1491.

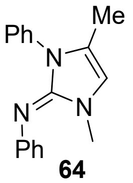

64

1,4-Dimethyl-*N*-3-diphenyl-1,3-dihydro-2*H*-imidazol-2-imine (64)

Yellow solid. Mp: 30-33 °C. ^{1}H NMR (400 MHz, $CDCl_3$) δ 7.20-7.14 (m, 2H), 7.13-7.04 (m, 3H), 6.89-6.82 (m, 2H), 6.59 (d, J = 7.6 Hz, 2H), 6.51 (t, J = 7.2 Hz, 1H), 6.08 (d, J = 1.2 Hz, 1H), 3.23 (s, 3H), 1.84 (d, J = 1.2 Hz, 3H). ^{13}C NMR (150 MHz, $CDCl_3$) δ 150.1, 147.6, 136.3, 128.6, 128.1, 127.8, 127.2, 121.8, 120.9, 118.4, 111.6, 33.0, 11.0. HRMS (ESI), *m/z* calcd. for $C_{17}H_{18}N_3$ ([M+H]$^+$) 264.1495, found: 264.1498.

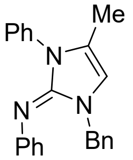

65

1-Benzyl-4-methyl-*N*-3-diphenyl-1,3-dihydro-2*H*-imidazol-2-imine (65)[16]

Yellow solid. ^{1}H NMR (600 MHz, $CDCl_3$) δ 7.40-7.28 (m, 5H), 7.15 (t, J = 7.5 Hz, 2H), 711-7.05 (m, 3H), 6.82 (t, J = 7.5 Hz, 2H), 6.57 (d, J = 7.8 Hz, 2H), 6.49 (t, J = 7.2 Hz, 1H), 6.00 (s, 1H), 4.87 (s, 2H), 1.79 (s, 3H). HRMS (ESI), *m/z* calcd. for $C_{23}H_{22}N_3$ ([M+H]$^+$) 340.1808, found: 340.1805.

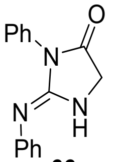

66

3-Phenyl-2-(phenylimino)imidazolidin-4-one (66)

Yellow solid. Mp: 200-203 °C. ^{1}H NMR (600 MHz, MeOD) δ 7.41 (t, J = 7.8 Hz, 4H), 7.35 (d, J = 7.8 Hz, 4H), 7.26 (t, J = 7.5 Hz, 2H), 3.88 (s, 2H). ^{13}C NMR (150 MHz, MeOD) δ 172.8, 154.4, 129.4, 126.5, 124.1, 45.8. HRMS (ESI), *m/z* calcd. for $C_{15}H_{14}N_3O$ ([M+H]$^+$) 252.1131, found: 252.1121.

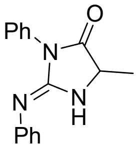

67

5-Methyl-3-phenyl-2-(phenylimino)imidazolidin-4-one (67)

Yellow solid. Mp: 204-207 ºC. ^{1}H NMR (600 MHz, MeOD) δ 7.37 (t, J = 7.8 Hz, 4H), 7.29 (d, J = 8.4 Hz, 4H), 7.21 (t, J = 7.2 Hz, 2H), 4.19 (q, J = 7.2 Hz, 1H), 1.48 (d, J = 7.2 Hz, 3H). ^{13}C NMR (150 MHz, MeOD) δ 176.2, 153.4, 136.0 129.4, 126.2, 123.4, 53.7, 16.8. HRMS (ESI), *m/z* calcd. for $C_{16}H_{16}N_3O$ ($[M+H]^+$) 266.1288, found: 266.1290.

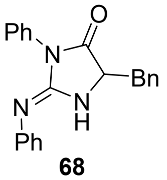

68

5-Benzyl-3-phenyl-2-(phenylimino)imidazolidin-4-one (68)

Yellow solid. Mp: 139-142 ºC. ^{1}H NMR (400 MHz, TFA) δ 7.56-7.43 (m, 9H), 7.28-7.12 (m, 6H), 5.10-4.94 (m, 1H), 3.54 (d, J = 12.4 Hz, 1H), 3.31 (dd, J = 14.0, 3.6 Hz, 1H). ^{13}C NMR (150 MHz, TFA) δ 174.8, 154.5, 133.4, 131.4, 130.4, 129.6, 129.5, 128.7, 128.3, 125.7, 56.1, 37.1. HRMS (ESI), *m/z* calcd. for $C_{22}H_{20}N_3O$ ($[M+H]^+$) 342.1601, found: 342.1589.

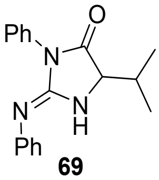

69

5-Isopropyl-3-phenyl-2-(phenylimino)imidazolidin-4-one (69)

Yellow solid. Mp: 119-121 ºC. ^{1}H NMR (600 MHz, MeOD) δ 7.36 (t, J = 7.8 Hz, 4H), 7.27 (d, J = 7.8 Hz, 4H), 7.23 (t, J = 7.2 Hz, 2H), 3.91 (d, J = 6.0 Hz, 1H), 2.37-2.28 (m, 1H), 1.05 (d, J = 6.6 Hz, 3H), 1.00 (d, J = 7.2 Hz, 3H). ^{13}C NMR (150 MHz, $CDCl_3$) δ 172.4, 150.1, 147.8, 132.3, 130.4, 129.5, 129.1, 128.3, 127.8, 127.3, 123.2, 122.4, 118.7, 62.5, 31.1, 18.7, 16.0. HRMS (ESI), *m/z* calcd. for $C_{18}H_{20}N_3O$ ($[M+H]^+$) 294.1601, found: 294.1601.

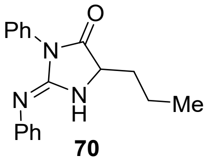

70

3-Phenyl-2-(phenylimino)-5-propylimidazolidin-4-one (70)

Yellow solid. Mp: 126-129 °C. ^{1}H NMR (400 MHz, MeOD) δ 7.35 (t, J = 7.8 Hz, 4H), 7.26 (d, J = 7.6 Hz, 4H), 7.20 (t, J = 7.4 Hz, 2H), 4.15-4.08 (m, 1H), 1.95-1.77 (m, 2H), 1.49-1.40 (m, 2H), 0.96 (t, J = 7.4 Hz, 3H). ^{13}C NMR (150 MHz, MeOD) δ 175.5, 153.6, 135.9, 129.4, 126.2, 123.5, 58.3, 33.3, 18.7, 12.8. HRMS (ESI), *m/z* calcd. for $C_{18}H_{20}N_3O$ ([M+H]$^+$) 294.1601, found: 294.1599.

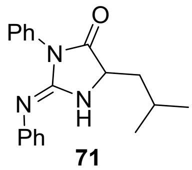

71

5-Isobutyl-3-phenyl-2-(phenylimino)imidazolidin-4-one (71)

Yellow solid. Mp: 121-123 °C. ^{1}H NMR (600 MHz, MeOD) δ 7.32 (t, J = 7.5 Hz, 4H), 7.23 (d, J = 7.8 Hz, 4H), 7.17 (t, J = 7.2 Hz, 2H), 4.18-4.10 (m, 1H), 1.86-1.73 (m, 3H), 0.97 (s, 6H). ^{13}C NMR (150 MHz, MeOD) δ 176.5, 153.9, 136.1, 129.3, 126.0, 123.1, 57.4, 40.1, 24.8, 22.1, 20.7. HRMS (ESI), *m/z* calcd. for $C_{19}H_{22}N_3O$ ([M+H]$^+$) 308.1757, found: 308.1755.

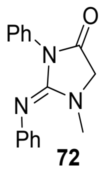

72

1-Methyl-3-phenyl-2-(phenylimino)imidazolidin-4-one (72)

Yellow solid. Mp: 148-149 °C. ^{1}H NMR (400 MHz, MeOD) δ 7.21 (t, J = 7.8 Hz, 4H), 7.12 (d, J = 8.0 Hz, 4H), 7.04 (t, J = 7.4 Hz, 2H), 4.08 (s, 2H), 3.12 (s, 3H). ^{13}C NMR (100 MHz, MeOD) δ 172.8, 154.9, 137.1, 129.0, 125.1, 121.7, 56.0, 38.3. HRMS (ESI), *m/z* calcd. for $C_{16}H_{16}N_3O$ ([M+H]$^+$) 266.1288, found: 266.1286.

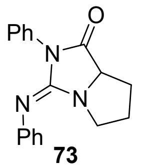

73

2-Phenyl-3-(phenylimino)hexahydro-1*H*-pyrrolo[1,2-*c*]imidazol-1-one (73)

Yellow solid. Mp: 154-156 °C. ^{1}H NMR (400 MHz, MeOD) δ 7.35 (t, J = 7.8 Hz, 4H), 7.26 (d, J = 7.6 Hz, 4H), 7.20 (t, J = 7.4 Hz, 2H), 4.15-4.08 (m, 1H), 1.95-1.77 (m, 2H), 1.52-1.36 (m, 2H), 0.96 (t, J = 7.4 Hz, 2H). ^{13}C NMR (150 MHz, MeOD) δ 175.5, 153.6, 135.9,

129.4, 126.2, 123.5, 58.3, 33.3, 18.7, 12.8. HRMS (ESI), *m/z* calcd. for $C_{18}H_{18}N_3O$ ([M+H]$^+$) 292.1444, found: 292.1444.

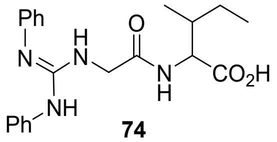

74

2-(2-(2,3-Diphenylguanidino)acetamido)-3-methylpentanoic acid (74)

White solid. Mp: 147-145 °C. ^{1}H NMR (400 MHz, DMSO) δ 8.13 (d, J = 7.2 Hz, 1H), 7.18 (t, J = 7.8 Hz, 4H), 7.07 (d, J = 8.0 Hz, 4H), 6.92 (t, J = 7.4 Hz, 2H), 4.19-4.13 (m, 1H), 4.05-3.83 (m, 2H), 1.80-1.69 (m, 1H), 1.50-1.32(m, 1H), 1.15-1.04 (m, 1H), 0.85-0.70 (m, 6H). ^{13}C NMR (150 MHz, MeOD) δ 176.4, 153.9, 136.09, 129.39, 126.09, 123.2, 57.5, 40.0, 24.8, 22.1, 20.7.

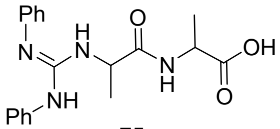

75

(*N*,*N'*-Diphenylcarbamimidoyl)alanylalanine (75)

White solid. Mp: 176-180°C. ^{1}H NMR (400 MHz, MeOD) δ 7.45-7.29 (m, 8H), 7.23 (t, J = 7.2 Hz, 2H), 4.45 (q, J = 7.0 Hz, 1H), 4.23 (q, J = 7.2 Hz, 1H), 1.53 (d, J = 7.2 Hz, 3H), 1.30 (d, J = 7.2 Hz, 3H). ^{13}C NMR (100 MHz, MeOD) δ 170.7, 154.1, 136.0, 129.4, 126.3, 123.4, 52.8, 50.7, 17.6, 16.7. HRMS (ESI), *m/z* calcd. for $C_{19}H_{23}N_4O_3$ ([M+H]$^+$) 355.1765, found: 355.1765.

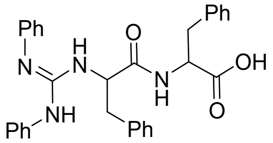

76

(*N*,*N'*-Diphenylcarbamimidoyl)phenylalanylphenylalanine (76)

White solid. Mp: 124-126 °C. ^{1}H NMR (600 MHz, MeOD) δ 7.39-7.30 (m, 3H), 7.29-7.21 (m, 8H), 7.20-7.10 (m, 5H), 6.92-6.77 (m, 4H), 4.65 (dd, J = 10.8, 4.8 Hz, 1H), 4.58 (dd, J = 8.4, 4.8 Hz, 1H), 3.30-3.21 (m, 2H), 3.05-2.97 (m, 2H). ^{13}C NMR (150 MHz, MeOD) δ 176.4, 169.9, 153.7, 138.2, 136.38 (s), 135.4, 129.3, 129.3, 128.7, 127.8, 127.1, 126.2, 126.0, 123.4, 58.3, 56.5, 38.0, 37.1. HRMS (ESI), *m/z* calcd. for $C_{31}H_{31}N_4O_3$ ([M+H]$^+$) 507.2391, found: 507.2391.

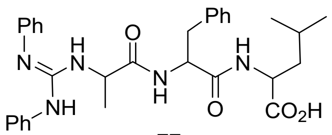

77

(*N*,*N'*-Diphenylcarbamimidoyl)alanylphenylalanylleucine (77)

White solid. Mp: 91-93 °C. ^{1}H NMR (400 MHz, MeOD) δ 7.34 (t, J = 7.8 Hz, 4H), 7.27-7.15 (m, 8H), 7.14-7.01 (m, 3H), 4.74 (dd, J = 10.0, 4.8 Hz, 1H), 4.42 (q, J = 6.8 Hz, 1H), 4.34-4.25 (m, 1H), 3.19 (d, J = 7.5 Hz, 1H), 2.87 (dd, J = 14.2, 9.9 Hz, 1H), 1.76-1.54 (m, 3H), 1.45 (d, J = 6.8 Hz, 3H), 1.37-1.27 (m, 2H), 0.91 (dd, J = 8.5, 6.2 Hz, 6H). ^{13}C NMR (100 MHz, MeOD) δ 171.8, 171.1, 153.8, 137.1, 136.1, 129.3, 128.8, 128.0, 126.3, 126.1, 123.2, 54.9, 52.3, 41.9, 37.2, 24.7, 22.4, 21.1, 17.1. HRMS (ESI), *m/z* calcd. for $C_{31}H_{38}N_5O_4$ ([M+H]$^+$) 544.2918, found: 544.2916.

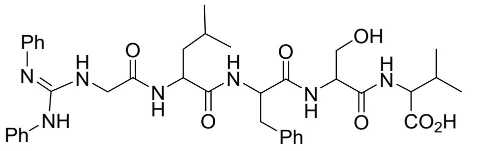

78

9-Benzyl-15-ethyl-12-(hydroxymethyl)-6-isobutyl-4,7,10,13-tetraoxo-1-(phenylamino)-1-(phenylimino)-2,5,8,11,14-pentaazahexadec-an-16-oic acid (78)

White solid. Mp: 202-204 °C. ^{1}H NMR (600 MHz, TFA) δ 7.60-7.51 (m, 6H), 7.42-7.24 (m, 9H), 5.12-4.96 (m, 2H), 4.85-4.79 (m, 1H), 4.69 (d, J = 4.2 Hz, 1H), 4.48-4.35 (m, 2H), 4.23 -4.10 (m, 2H), 3.27-3.14 (m, 2H), 2.52-2.42 (m, 1H), 1.75-1.62 (m, 3H), 1.19-1.07 (m, 7H), 1.02-0.91 (m, 7H). ^{13}C NMR (150 MHz, TFA) δ 176.9, 174.4, 173.4, 170.8, 169.9, 155.2, 133.9, 131.7, 130.4, 129.6, 128.7, 128.7, 128.6, 127.5, 125.8, 62.1, 58.5, 55.7, 54.6, 52.9, 44.1, 40.3, 37.6, 30.4, 24.4, 21, 19.6, 17.5, 16.0. HRMS (ESI), *m/z* calcd. for $C_{38}H_{50}N_7O_7$ ([M+H]$^+$) 716.3766, found: 716.3767.

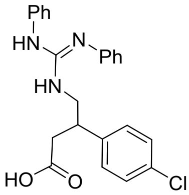

79

3-(4-Chlorophenyl)-4-(2,3-diphenylguanidino)butanoic acid (79)

White solid. Mp: 140-142 °C. ^{1}H NMR (400 MHz, TFA) δ 7.53-7.29 (m, 8H), 7.21-6.82

(m, 6H), 3.85 (dd, J = 13.4, 4.2 Hz, 1H), 3.65-3.54 (m, 1H), 3.50-3.36 (m, 1H), 2.83 (d, J = 7.2 Hz, 2H). ^{13}C NMR (150 MHz, TFA) δ 178.2, 154.2, 136.5, 134.9, 130.3, 129.6, 129.5, 128.4, 125.9, 46.0, 40.5, 36.8. HRMS (ESI), m/z calcd. for $C_{23}H_{22}ClN_3O_2$ ($[M+H]^+$) 408.1473, found: 408.1474.

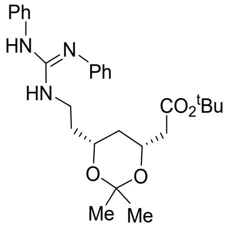

80

Tert-butyl-2-((4R,6R)-6-(2-(-2,3-diphenylguanidino)ethyl)-2,2-dimethyl-1,3-dioxan-4-yl)acetate (80)

Yellow solid. Mp: 138-140 °C. ^{1}H NMR (600 MHz, $CDCl_3$) δ 7.45-7.33 (m, 2H), 7.30-7.26 (m, 3H), 7.06-6.95 (m, 5H), 4.28-4.14 (m, 1H), 4.00-3.90 (m, 1H), 3.60-3.51 (m, 1H), 3.42-3.30 (m, 1H), 2.33-2.23 (m, 1H), 2.30-2.22 (m, 1H), 1.82-1.74 (m, 1H), 1.72-1.62 (m, 1H), 1.58-1.46 (m, 1H), 1.46-1.38 (m, 9H), 1.34-1.30 (m, 3H), 1.29-1.17 (m, 2H), 1.01 (s, 3H). ^{13}C NMR (150 MHz, $CDCl_3$) δ 180.1, 170.2, 148.5, 137.5, 129.4, 126.7, 125.1, 123.2, 98.7, 80.7, 68.8, 66.14 (s), 42.6, 39.4, 36.2, 35.3, 29.7, 28.1, 19.6. HRMS (ESI), m/z calcd. for $C_{27}H_{38}N_3O_4$ ($[M+H]^+$) 468.2857, found: 468.2857.

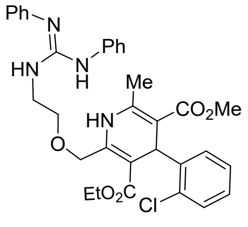

81

3-Ethyl-5-methyl-4-(2-chlorophenyl)-2-((2-(2,3-diphenylguanidino)ethoxy)methyl)-6-methyl-1,4-dihydropyridine-3,5-dicarboxylate (81)

Yellow solid. Mp: 150-151 °C. ^{1}H NMR (400 MHz, $CDCl_3$) δ 7.82 (s, 1H), 7.39 (d, J = 7.6 Hz, 1H), 7.25-7.18 (m, 5H), 7.12-7.00 (m, 8H), 5.40 (s, 1H), 4.81 (d, J = 14.0 Hz, 1H), 4.66 (d, J = 14.0 Hz, 1H), 4.10-3.94 (m, 2H), 3.68 (s, 2H), 3.63-3.49 (m, 5H), 2.35 (s, 3H), 1.17 (t, J = 7.0 Hz, 3H). ^{13}C NMR (100 MHz, $CDCl_3$) δ 168.1, 167.2, 145.9, 145.1, 144.4, 132.2, 131.4, 129.5, 129.2, 127.4, 127.0, 125.1, 122.9, 103.4, 103.2, 69.9, 67.9, 60.0, 50.78

(s), 42.9, 37.1, 19.3, 14.3. HRMS (ESI), *m/z* calcd. for $C_{33}H_{36}ClN_4O_5$ ([M+H]$^+$) 603.2369, found: 603.2371.

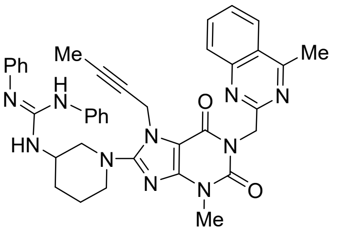

82

1-(1-(7-(But-2-yn-1-yl)-3-methyl-1-((4-methylquinazolin-2-yl)methyl)-2,6-dioxo-2,3,6,7-tetrahydro-1H-purin-8-yl)piperidin-3-yl)-2,3-diphenylguanidine (82)

Yellow solid. Mp: 132-134 °C. ^{1}H NMR (600 MHz, $CDCl_3$) δ 8.01 (d, J = 8.4, 1H), 7.85 (d, J = 8.4 Hz, 1H), 7.76-7.71 (m, 1H), 7.51 (t, J = 7.5, 1H), 7.29-7.22 (m, 5H), 7.07-7.00 (m, 5H), 5.56 (d, J = 3.0 Hz, 2H), 4.83-4.80 (m, 2H), 4.26 (s, 1H), 3.64 (dd, J = 13.2, 2.4 Hz, 1H), 3.55-3.48 (m, 2H), 3.42-3.32 (m, 4H), 2.88 (s, 3H), 1.97-1.87 (m, 3H), 1.79-1.73 (m, 4H). ^{13}C NMR (150 MHz, $CDCl_3$) δ 168.5, 161.2, 155.9, 154.4, 151.8, 150.0, 147.8, 133.2, 129.5, 128.9, 126.7, 124.9, 123.2, 123.0, 104.5, 81.4, 73.2, 54.4, 50.6, 47.0, 46.4, 35.8, 29.6, 29.1, 22.3, 21.8. HRMS (ESI), *m/z* calcd. for $C_{27}H_{38}N_3O_4$ ([M+H]$^+$) 468.2857, found: 468.2857.

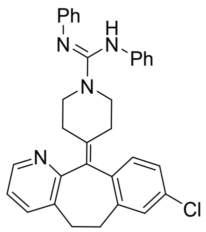

83

4-(8-Chloro-5,6-dihydro-11*H*-benzo[5,6]cyclohepta[1,2-*b*]pyridin-11-ylidene)-N,N'-diphenylpiperidine-1-carboximidamide (83)

Yellow solid. Mp: 200-202 °C. ^{1}H NMR (400 MHz, $CDCl_3$) δ 8.52 (d, J = 3.6 Hz, 1H), 7.60-7.52 (m, 2H), 7.45-7.32 (m, 5H), 7.30 (s, 1H), 7.21 (dd, J = 7.6, 4.8 Hz, 1H), 7.16-7.00 (m, 6H), 5.65 (s, 1H), 3.91-3.78 (m, 2H), 3.65-3.46 (m, 2H), 3.21-3.09 (m, 2H), 3.04-2.89 (m, 2H), 2.76-2.66 (m, 1H), 2.61-2.40 (m, 3H). ^{13}C NMR (100 MHz, $CDCl_3$) δ 168.1, 167.2, 145.9, 145.1, 144.4, 132.2, 131.4, 129.5, 129.2, 127.4, 127.0, 125.1, 122.9, 103.4,

103.2, 69.9, 67.9, 60.0, 50.8, 42.9, 37.1, 19.3, 14.3. HRMS (ESI), *m/z* calcd. for $C_{32}H_{30}ClN_4$ ([M+H]$^+$) 505.2154, found: 505.2151.

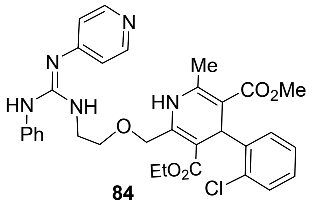

84

3-Ethyl-5-methyl-4-(2-chlorophenyl)-6-methyl-2-((2-(3-phenyl-2-(pyridin-4-yl)guanidino)ethoxy)methyl)-1,4-dihydropyridine-3,5-dicarboxylate (84)

Yellow solid. Mp: 71-73 ^oC. ^1H NMR (600 MHz, $CDCl_3$) δ 8.23 (s, 2H), 7.35 (dd, J = 7.8, 1.2 Hz, 1H), 7.29 (t, J = 7.5 Hz, 2H), 7.26 (s, 1H), 7.23 (d, J = 7.2 Hz, 1H), 7.17-7.07 (m, 5H), 7.06-7.01 (m, 1H), 6.88 (d, J = 4.2 Hz, 2H), 5.40 (s, 1H), 4.81 (d, J = 15.6 Hz, 1H), 4.71 (d, J = 15.6 Hz, 1H), 4.09-4.01 (m, 2H), 3.77-3.69 (m, 2H), 3.63-3.54 (m, 5H), 2.28 (s, 3H), 1.18 (t, J = 7.2 Hz, 3H). ^{13}C NMR (150 MHz, $CDCl_3$) δ 168.0, 167.2, 145.6, 144.7, 144.2, 132.4, 131.5, 129.6, 129.3, 127.5, 126.9, 124.8, 122.7, 117.0, 103.8, 102.2, 70.3, 68.0, 59.9, 50.8, 41.8, 37.3, 19.4, 14.3. HRMS (ESI), *m/z* calcd. for $C_{32}H_{35}ClN_5O_5$ ([M+H]$^+$) 604.2321, found: 604.2320.

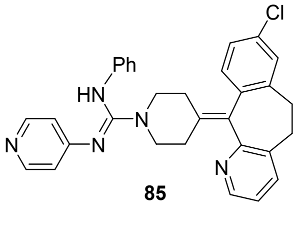

85

4-(8-Chloro-5,6-dihydro-11*H*-benzo[5,6]cyclohepta[1,2-*b*]pyridin-11-ylidene)-*N*-phenyl-*N'*-(pyridin-4-yl)piperidine-1-carboximidamide (85)

Yellow solid. Mp: 140-142 ^oC. ^1H NMR (600 MHz, $CDCl_3$) δ 8.35 (dd, J = 4.8, 1.2 Hz, 1H), 8.18 (d, J = 6.0 Hz, 2H), 7.41 (dd, J = 7.5, 1.5 Hz, 1H), 7.20 (t, J = 7.8 Hz, 2H), 7.14 (s, 1H), 7.11-7.08 (m, 2H), 7.06 (dd, J = 7.8, 4.8 Hz, 1H), 6.95 (t, J = 7.5 Hz, 1H), 6.90 (d, J = 7.2 Hz, 2H), 6.72 (d, J = 6.0 Hz, 2H), 5.97 (s, 1H), 3.76-3.64 (m, 2H), 3.45-3.26 (m, 2H), 3.12-3.02 (m, 2H), 2.88-2.75 (m, 2H), 2.63-2.51 (m, 1H), 2.50-2.41 (m, 1H), 2.40-2.28 (m, 2H). ^{13}C NMR (150 MHz, $CDCl_3$) δ 157.0, 149.8, 146.7, 139.6, 137.6, 137.6, 137.5, 134.2, 133.4, 133.0, 130.5, 129.4, 129.0, 126.2, 122.8, 122.3, 47.7, 47.6, 31.7, 31.5, 30.3, 30.1. HRMS (ESI), *m/z* calcd. for $C_{31}H_{29}ClN_5$ ([M+H]$^+$) 506.2106, found: 506.2110.

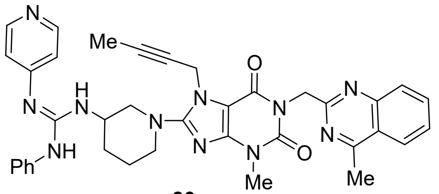

86

1-(1-(7-(But-2-yn-1-yl)-3-methyl-1-((4-methylquinazolin-2-yl)methyl)-2,6-dioxo-2,3,6,7-tetrahydro-1*H*-purin-8-yl)piperidin-3-yl)-3-phenyl-2-(pyridin-4-yl)guanidine (86)

Yellow solid. Mp: 136-137 °C. ^{1}H NMR (600 MHz, MeOD) δ 8.22 (d, J = 8.4 Hz, 1H), 8.07-8.02 (m, 2H), 7.88 (t, J = 7.5 Hz, 1H), 7.83 (d, J = 8.4 Hz, 1H), 7.66 (t, J = 7.5 Hz, 1H), 7.20 (t, J = 7.5 Hz, 2H), 7.05 (d, J = 7.8 Hz, 2H), 7.01-6.89 (m, 3H), 5.46 (s, 2H), 4.16-4.10 (m, 1H), 3.82 (d, J = 10.8 Hz, 1H), 3.63-3.55 (m, 1H), 3.46-3.35 (m, 5H), 2.93 (s, 3H), 2.10-2.02 (m, 1H), 1.98-1.91 (m, 1H), 1.84-1.76 (m, 2H), 1.74 (s, 3H). ^{13}C NMR (150 MHz, $CDCl_3$) δ 168.8, 161.3, 155.9, 154.4, 151.8, 149.9, 149.6, 147.9, 133.5, 129.7, 128.9, 128.5, 126.8, 125.0, 123.2, 123.1, 122.8, 119.3, 104.5, 73.0, 54.3, 50.6, 47.2, 46.5, 35.8, 29.6, 29.2, 22.3, 21.8, 3.7. HRMS (ESI), *m/z* calcd. for $C_{37}H_{38}N_{11}O_2$ ([M+H]$^+$) 668.3204, found: 668.3190.

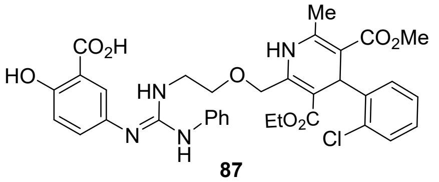

87

5-((((2-((4-(2-Chlorophenyl)-3-(ethoxycarbonyl)-5-(methoxycarbonyl)-6-methyl-1,4-dihydropyridin-2-yl)methoxy)ethyl)amino)(phenylamino)methylene)amino)-2-hydroxybenzoic acid (87)

Yellow solid. Mp: 153-155 °C. ^{1}H NMR (600 MHz, MeOD) δ 7.79 (d, J = 2.4 Hz, 1H), 7.41-7.35 (m, 3H), 7.31-7.21 (m, 4H), 7.17 (dd, J = 8.7, 2.7 Hz, 1H), 7.12 (t, J = 7.5 Hz, 1H), 7.09-7.05 (m, 1H), 6.81 (d, J = 9.0 Hz, 1H), 5.43 (s, 1H), 4.82 (d, J = 113.2 Hz, 1H), 4.63 (d, J = 12.6 Hz, 1H), 4.01-3.92 (m, 2H), 3.79-3.71 (m, 2H), 3.66-3.53 (m, 5H), 2.24 (s, 3H), 1.12 (t, J = 6.9 Hz, 3H). ^{13}C NMR (150 MHz, MeOD) δ 173.5, 168.6, 167.4, 161.0, 155.3, 145.8, 145.7, 144.4, 135.8, 132.1, 131.3, 129.8, 129.7, 129.1, 127.6, 127.6, 127.0,

126.9, 124.9, 124.5, 120.1, 117.5, 102.6, 69.6, 67.4, 59.9, 50.0, 42.4, 37.5, 17.5, 13.4. HRMS (ESI), *m/z* calcd. for $C_{34}H_{36}ClN_4O_8$ ($[M+H]^+$) 663.2216, found: 663.2208.

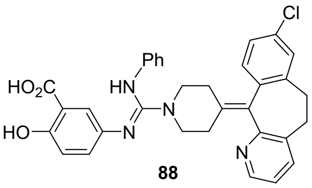

88

5-(((4-(8-Chloro-5,6-dihydro-11*H*-benzo[5,6]cyclohepta[1,2-*b*]pyridin-11-ylidene)piperidin-1-yl)(phenylamino)methylene)amino)-2-hydroxybenzoic acid (88)

Brown solid. Mp: 225-228 °C. ^1H NMR (600 MHz, TFA) δ 8.60 (br, 1H), 8.41 (br, 1H), 7.91 (br, 1H), 7.82 (br, 2H), 7.47-7.24 (m, 6H), 7.23-6.5 (m, 4H), 4.18-3.93 (m, 2H), 3.97-3.35 (m, 4H), 3.27-3.17 (m, 1H), 3.08-2.90 (m, 2H), 2.85-2.45 (m, 3H). HRMS (ESI), *m/z* calcd. for $C_{33}H_{30}ClN_4O_3$ ($[M+H]^+$) 565.2001, found: 565.1990. ^{13}C NMR spectrum was not detected due to its poor solubility. The purity is > 98.8% by HPLC (Please see pages S50-S51).

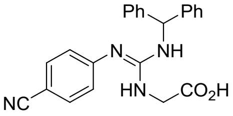

89

(*N*-Benzhydryl-*N′*-(4-cyanophenyl)carbamimidoyl)glycine (NC-174) (89)[17]

White solid. ^1H NMR (600 MHz, MeOD) δ 7.62 (d, *J* = 9 Hz, 2H), 7.41-7.36 (m, 4H), 7.35-7.26 (m, 7H), 6.06 (s, 1H), 3.84 (s, 2H). HRMS (ESI), *m/z* calcd. for $C_{23}H_{21}N_4O_2$ ($[M+H]^+$) 385.1659, found: 385.1657.

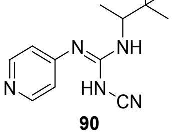

90

Pinacidil (90)[18]

Yellow solid. ^1H NMR (600 MHz, $CDCl_3$) δ 8.54 (d, *J* = 5.4 Hz, 2H), 7.17 (d, *J* = 4.8 Hz, 2H), 5.24 (d, *J* = 8.4 Hz, 1H), 3.87 (s, 1H), 1.14 (d, *J* = 6.6 Hz, 3H), 0.93 (s, 9H). HRMS (ESI), *m/z* calcd. for $C_{13}H_{20}N_5$ ($[M+H]^+$) 246.1713, found: 246.1714.

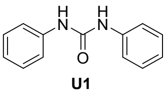

U1

1,3-Diphenylurea (U1)[19]

White solid. ^{1}H NMR (400 MHz, DMSO) δ 8.67 (s, 2H), 7.46 (d, J = 7.6 Hz, 4H), 7.28 (t, J = 7.8 Hz, 4H), 6.96 (t, J = 7.4 Hz, 2H).

References

[1] a) Bhagat, S. B. & Telvekar, V. N. NBS mediated protocol for the synthesis of *N*-bridged fused heterocycles in water *Tetrahedron Lett.* ***58***, 3662–3666 (2017); b) Chiacchio, A. D. et al. One-pot synthesis of 2-phenylimidazo[1,2-α] pyridines from acetophenone, [Bmim]Br(3) and 2-aminopyridine under solvent-free conditions. *Arch. Pharm. Pharm. Med. Chem.* **331**, 273–278 (1998).

[2] a) Viana, G. M. et al. Antileishmanial Thioureas: Synthesis, Biological Activity and in Silico Evaluations of New Promising Derivatives. *Chem. Pharm. Bull.* **65**, 911–919 (2017); b) Zhang, G. Clicking thiourea into a salen scaffold: Structures and cytotoxicity of cobalt(II) and nickel(II) complexes. *Inorg. Chem. Comm.* **48**, 127–130 (2014).

[3] Marenich, A. V., Cramer, C. J. & Truhlar, D. G. Universal solvation model based on solute electron density and on a continuum model of the solvent defined by the bulk dielectric constant and atomic surface tensions. *J. Phys. Chem. B* **113**, 6378−6396 (2009).

[4] Fu, Y. et al. Quantum-chemical predictions of absolute standard redox potentials of diverse organic molecules and free radicals in acetonitrile. *J. Am. Chem. Soc.* **127**, 7227-7234 (2005).

[5] Zhao,Y. et al. Dicyanopyrazine-derived push–pull chromophores for highly efficient photoredox catalysis. *RSC Adv.* **4**, 30062-30067 (2014).

[6] Saetan, T., Sukwattanasinitt, M. & Wacharasindhu, S. A mild photocatalytic synthesis of guanidine from thiourea under visible light. *Org. Lett.* **22**, 7864-7869 (2020).

[7] Zhang, W. X., Li, D., Wang, Z. & Xi, Z. Alkyl aluminum-catalyzed addition of amines to carbodiimides: a highly efficient route to substituted guanidines. *Organometallics* **28**, 882-887 (2009).

[8] Dangate, P. S. & Akamanchi, K. G. o-Iodoxybenzoic acid mediated oxidative condensation: synthesis of guanidines using 1,-3-disubstituted thiourea precursors. *Tetrahedron Lett.* **53**, 6765-6767 (2012).

[9] Miller, A. E. & Bischoff, J. J. A facile conversion of amino acids to guanidine acids. *Synthesis* **9**, 777-779 (1986).

[10] Kondraganti, L., Manabolu, S. & Dittakavi, R. Synthesis of benzimidazoles via domino intra and intermolecular C-N cross-coupling reaction. *ChemistrySelect* **3**, 11744-11748 (2018).

[11] Kumar, K. A., Kannaboina, P., Rao, D. N. & Das, P. Nickel-catalyzed chan–lam cross-coupling: chemoselective *N*-arylation of 2-aminobenzimidazole. *Org. Biomol. Chem.* **14**, 8989-8997 (2016).

[12] Rao, D. N. et al. Copper-catalyzed C-NH_2 arylation of 2-aminobenzimidazoles and related C-amino-NH-azoles. *Adv. Synth. Catal.* **358**, 2126-2133 (2016).

[13] Lan, P. et al. An efficient method to access 2-substituted benzimidazoles under solvent-free conditions. *Tetrahedron Lett.* **49**, 1910-1914 (2008).

[14] Kelly, B. et al. α^2-Adrenoceptor antagonists: synthesis, pharmacological evaluation, and molecular modeling investigation of pyridinoguanidine, pyridino-2-aminoimidazoline and their derivatives. *J. Med. Chem.* **58**, 963-977 (2015).

[15] Saari, R., Torma, J.-C. & Nevalainen, T. Microwave-assisted synthesis of quinoline, isoquinoline, quinoxaline and quinazoline derivatives as CB2 receptor agonists. *Bioorg. Med. Chem.* **19**, 939-950 (2011).

[16] Jia, J. H. et al. Synthesis of imidazole derivatives by cascade reaction: base-mediated addition/alkyne hydroamination of propargylamines and carbodiimides. *Synthesis* **47**, 3473-3478 (2015).

[17] Chen, J., Pattarawarapan, M., Zhang, A. J. & Burgess, K. Solution- and solid-phase syntheses of substituted guanidinocarboxylic acids. *J. Comb. Chem.* **2**, 276-281 (2000).

[18] Baeten, M. & Maes, B. U. W. Guanidine synthesis: use of amidines as guanylating agents. *Adv. Synth. Catal.* **358**, 826-833 (2016).

[19] Lin, B. & Waymouth, R. M. Urea anions: simple, fast, and selective catalysts for ring-opening polymerizations. *J. Am. Chem. Soc.* **139**, 1645-1652 (2017).

HPLC analysis data of 88

5-(((4-(8-Chloro-5,6-dihydro-11*H*-benzo[5,6]cyclohepta[1,2-*b*]pyridin-11-ylidene)piperidin-1-yl)(phenylamino)methylene)amino)-2-hydroxybenzoic acid

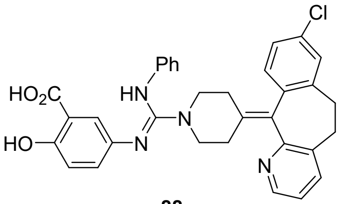

88

HPLC-1: Eclipse XDB-C18, mobile phases: A = H_2O; B = MeOH, runtime = 10 min, gradient: 10-95% B, flow rate = 1 mL/min, wavelength: λ = 254 nm.

	t_R	Area	Rel. Area (%)
1	2.41 min	397.8	1.0
2	2.72 min	39308.1	99.0

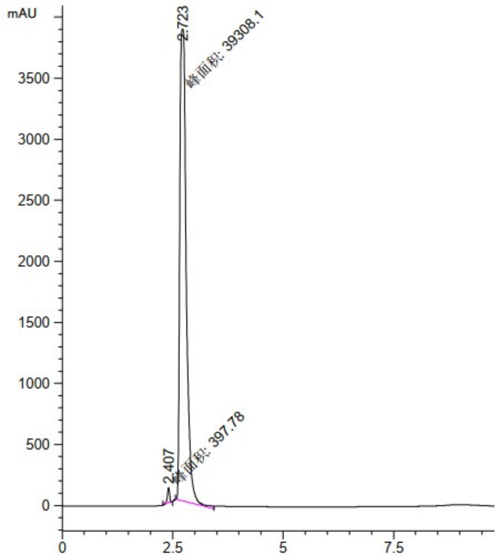

HPLC-2: Eclipse XDB-C18, mobile phases: A = H_2O; B = MeCN, runtime = 10 min, gradient: 8-95% B, flow rate = 1 mL/min, wavelength: λ = 254 nm

	t_R	Area	Rel. Area (%)
1	7.47 min	25468.4	98.8
2	8.10 min	311.3	1.2

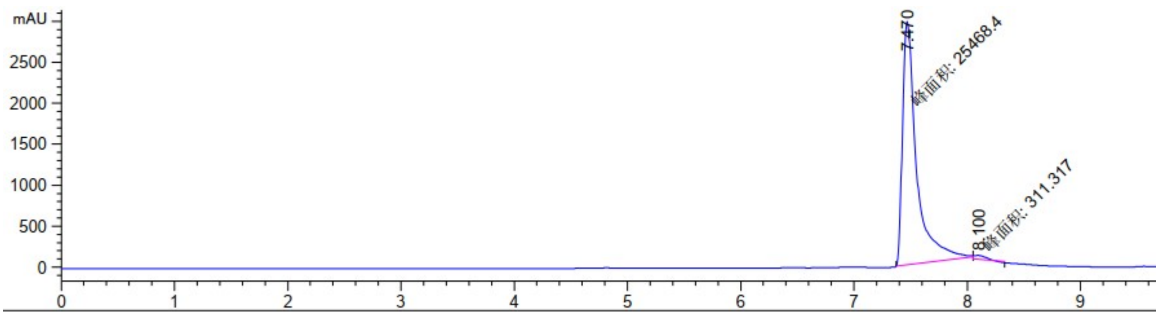

Bioactivities for Selected Products

Cell antiproliferative activity assay:

Cell antiproliferative activity was evaluated by the CellTiter-Glo (Promega, USA) assay. Make 1000 × compounds solution in DMSO, add 1 μl 1000 × compounds to 49 μl growth medium to make 20 × compounds. Dilute cell suspensions in growth medium to desired density and 95 μl were taken to 96-well plate. Add 5μl 20 × compounds into 96-well plate according to the plate map. Final DMSO concentration in each well was 0.1%. Then the cell was incubated at 37℃, 5% CO_2 for 72 h. Equilibrate the assay plate to room temperature before measurement. Add 20 μl of CellTiterGlo® Reagent into each well. Mix contents for 2 minutes on an orbital shaker to induce cell lysis. Incubate at room temperature for 10 minutes to stabilize luminescent signal. Record luminescence using EnVision Multilabel Reader (PerkinElmer). Cell viability (CV%) was calculated relative to vehicle (DMSO) treated control wells using following fromula: Cell viability(%) =(RLU compound -RLU blank)/(RLU control-RLU blank)*100%. The IC_{50} values were calculated using GraphPad Prism 6.0 software, fitting to a 4-parameter equation to generate concentration response curves. All assays were conducted with three parallel samples and three repetitions.

Bioactivities Data for Selected Products：

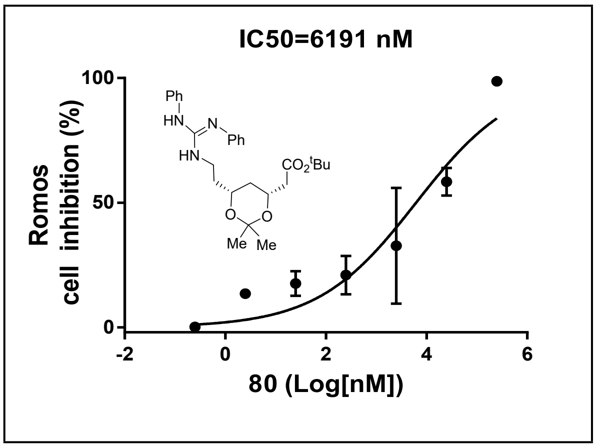
IC50=6191 nM
Romos cell inhibition (%)
80 (Log[nM])
100
50
0
-2
0
2
4
6

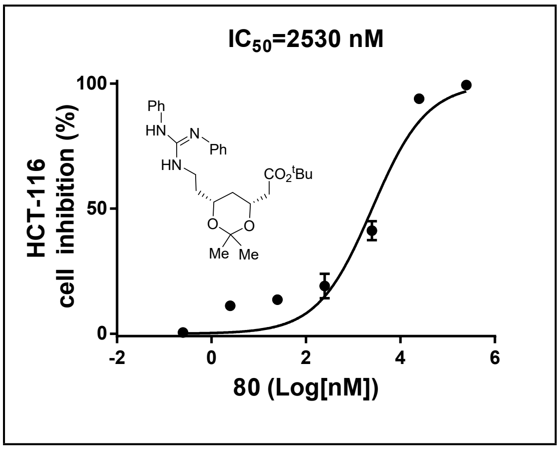
IC50=2530 nM
HCT-116 cell inhibition (%)
80 (Log[nM])
100
50
0
-2
0
2
4
6

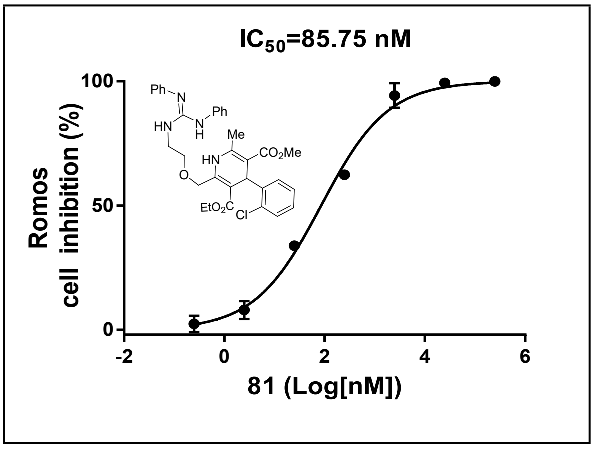
IC50=85.75 nM
Romos cell inhibition (%)
81 (Log[nM])
100
50
0
-2
0
2
4
6

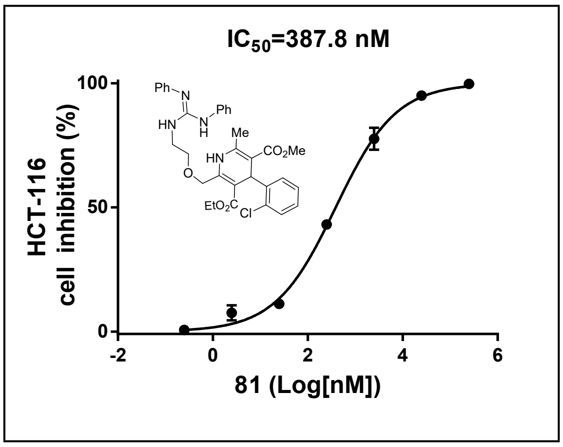
IC50=387.8 nM
HCT-116 cell inhibition (%)
81 (Log[nM])
100
50
0
-2
0
2
4
6

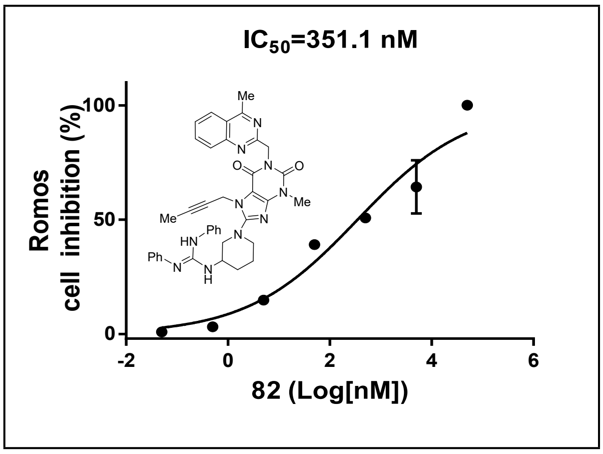
IC50=351.1 nM
Romos cell inhibition (%)
82 (Log[nM])
100
50
0
-2
0
2
4
6

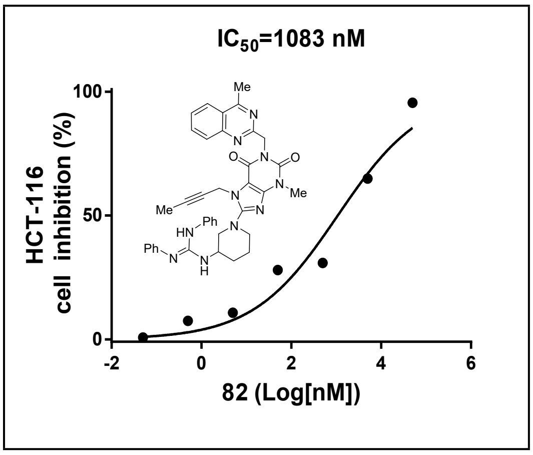
IC50=1083 nM
HCT-116 cell inhibition (%)
82 (Log[nM])
100
50
0
-2
0
2
4
6

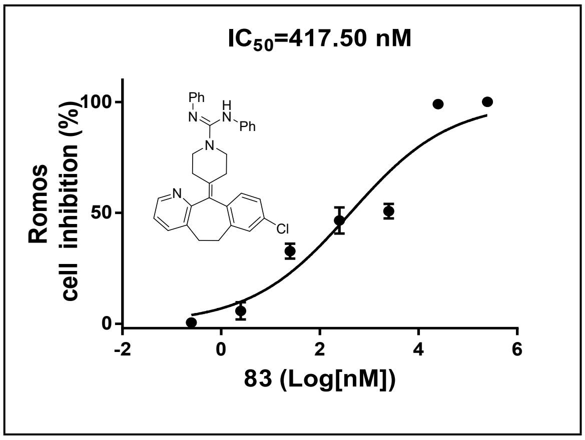
IC50=417.50 nM
Romos cell inhibition (%)
83 (Log[nM])
Cl

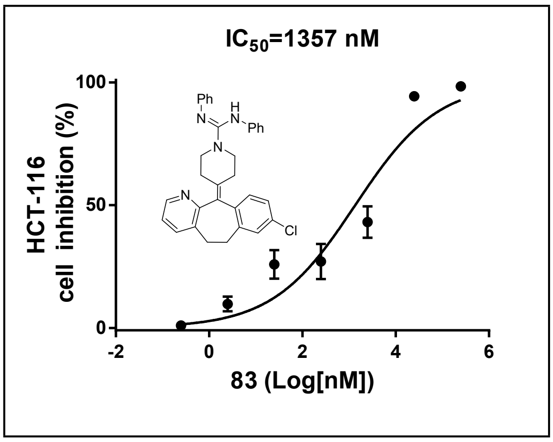
IC50=1357 nM
HCT-116 cell inhibition (%)
83 (Log[nM])
Cl

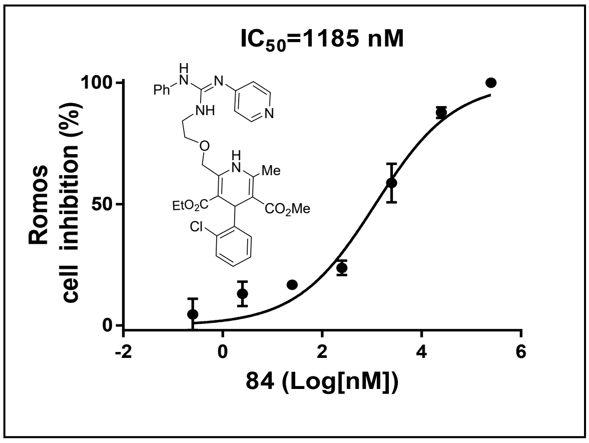
IC50=1185 nM
Romos cell inhibition (%)
84 (Log[nM])
EtO2C
CO2Me
Me
Cl

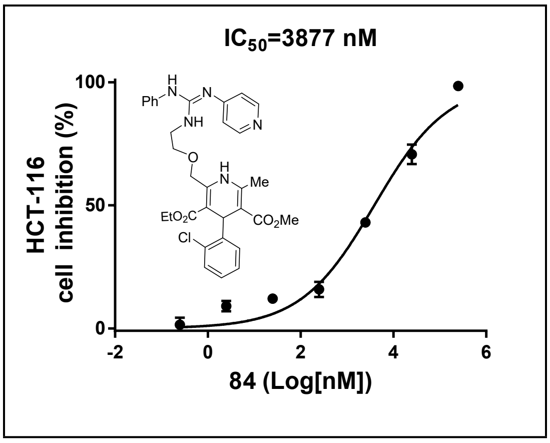
IC50=3877 nM
HCT-116 cell inhibition (%)
84 (Log[nM])
EtO2C
CO2Me
Me
Cl

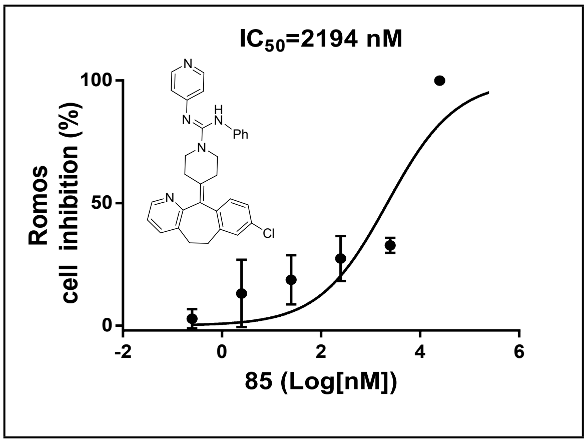
IC50=2194 nM
Romos cell inhibition (%)
85 (Log[nM])
Cl

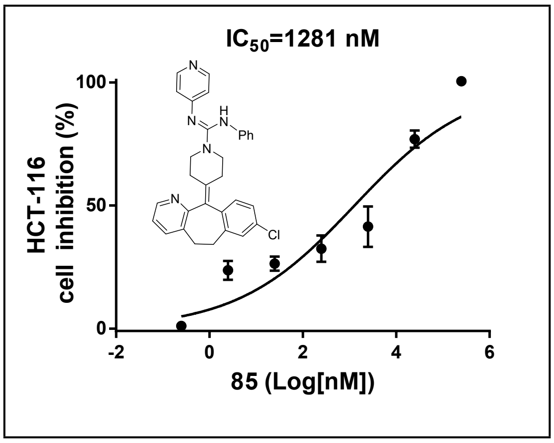
IC50=1281 nM
HCT-116 cell inhibition (%)
85 (Log[nM])
Cl

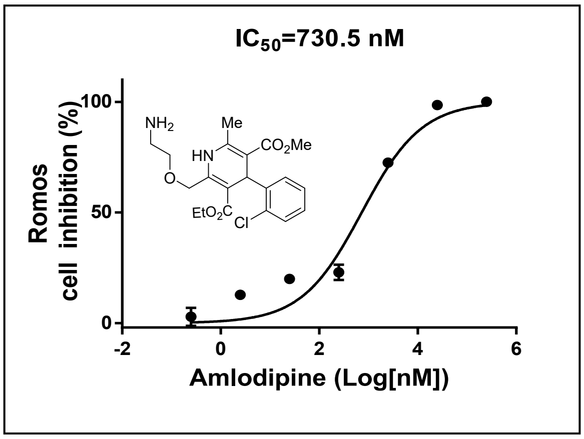
IC50=730.5 nM
Romos cell inhibition (%)
Amlodipine (Log[nM])
NH2
Me
CO2Me
HN
EtO2C
Cl

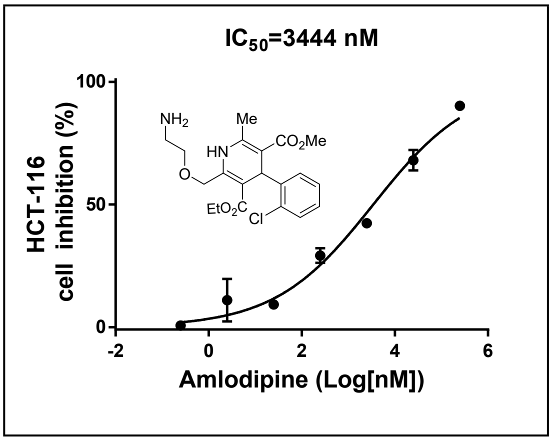
IC50=3444 nM
HCT-116 cell inhibition (%)
Amlodipine (Log[nM])
NH2
Me
CO2Me
HN
EtO2C
Cl

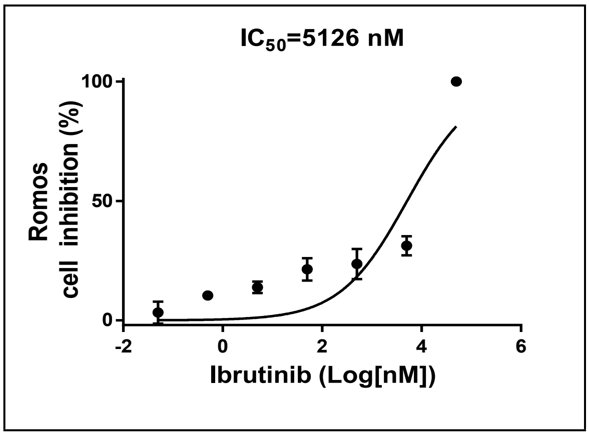
IC50=5126 nM
Romos
cell inhibition (%)
100
50
0
-2
0
2
4
6
Ibrutinib (Log[nM])

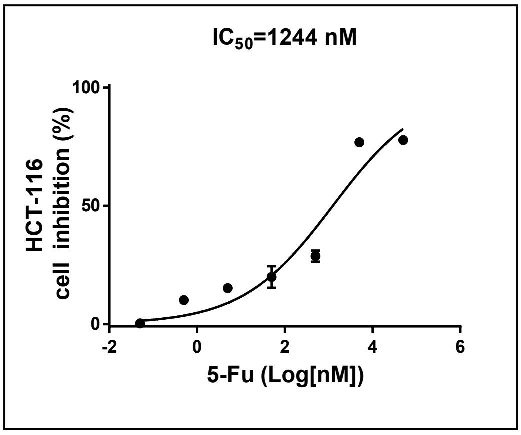
IC50=1244 nM
HCT-116
cell inhibition (%)
100
50
0
-2
0
2
4
6
5-Fu (Log[nM])

NMR spectra of all new compounds

2-(Pyridin-2-yl)-2,3-dihydroimidazo[1,2-*a*]pyridine-3-carboxamide (amide1)

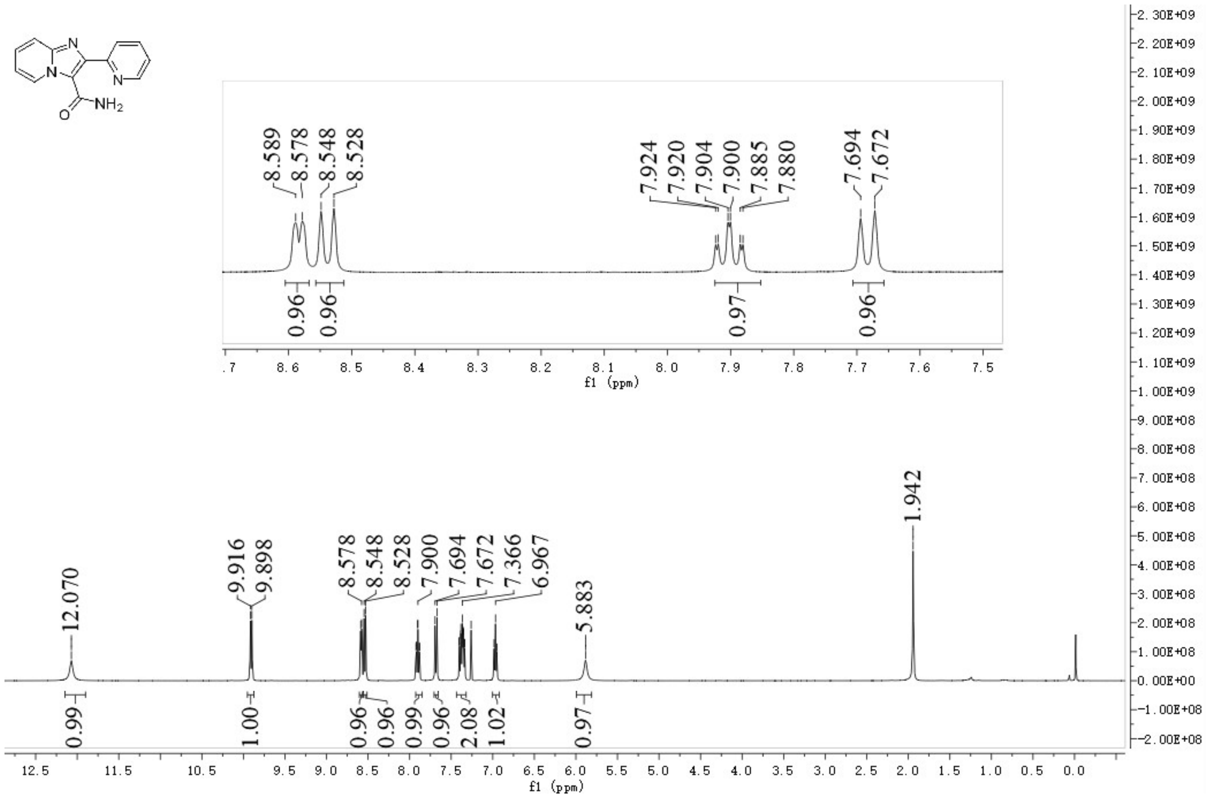

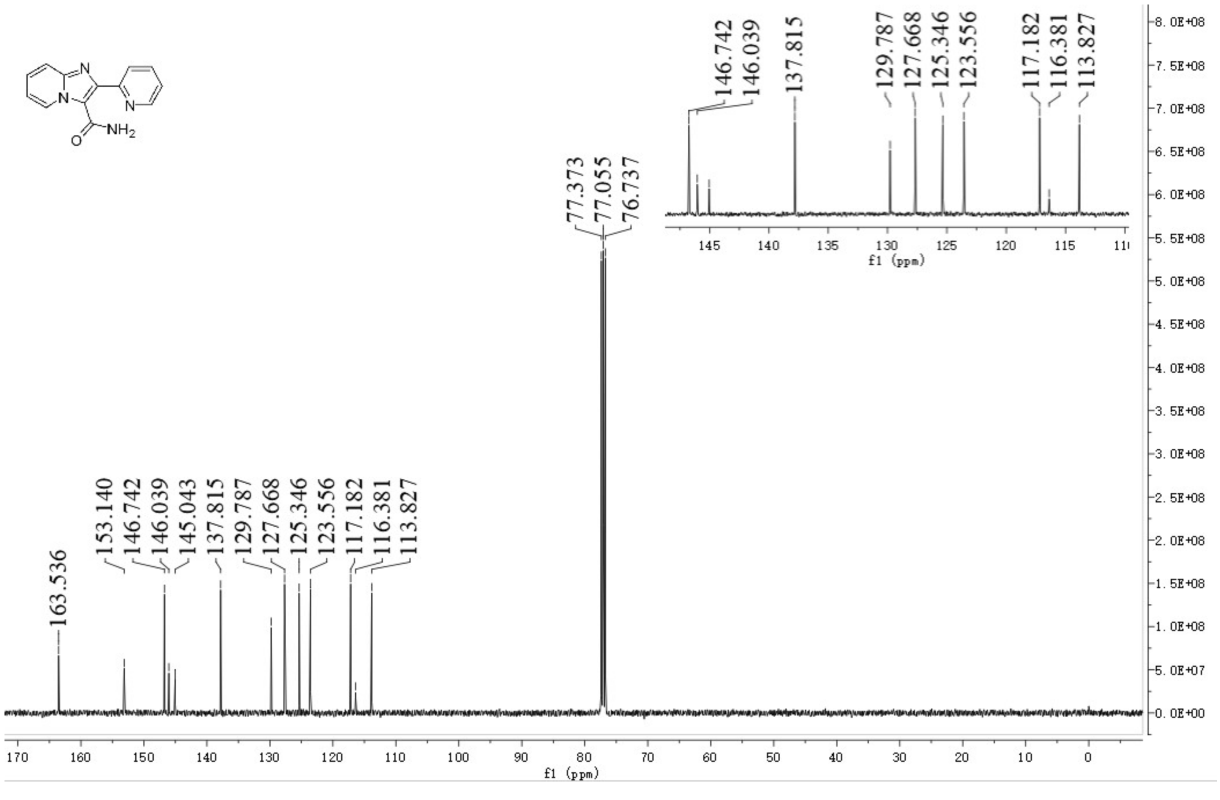

2-(4-Methylpyridin-2-yl)-2,3-dihydroimidazo[1,2-*a*]pyridine-3-carboxamide (amide2)

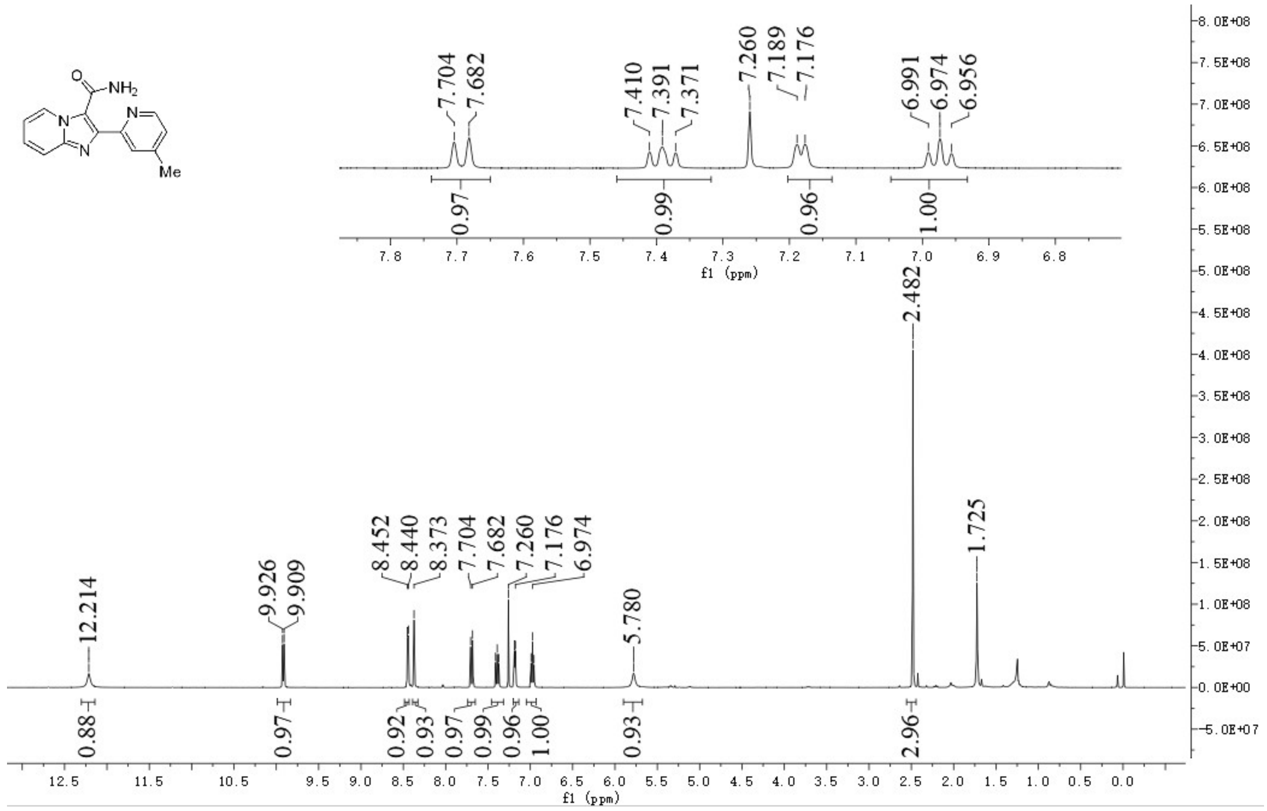

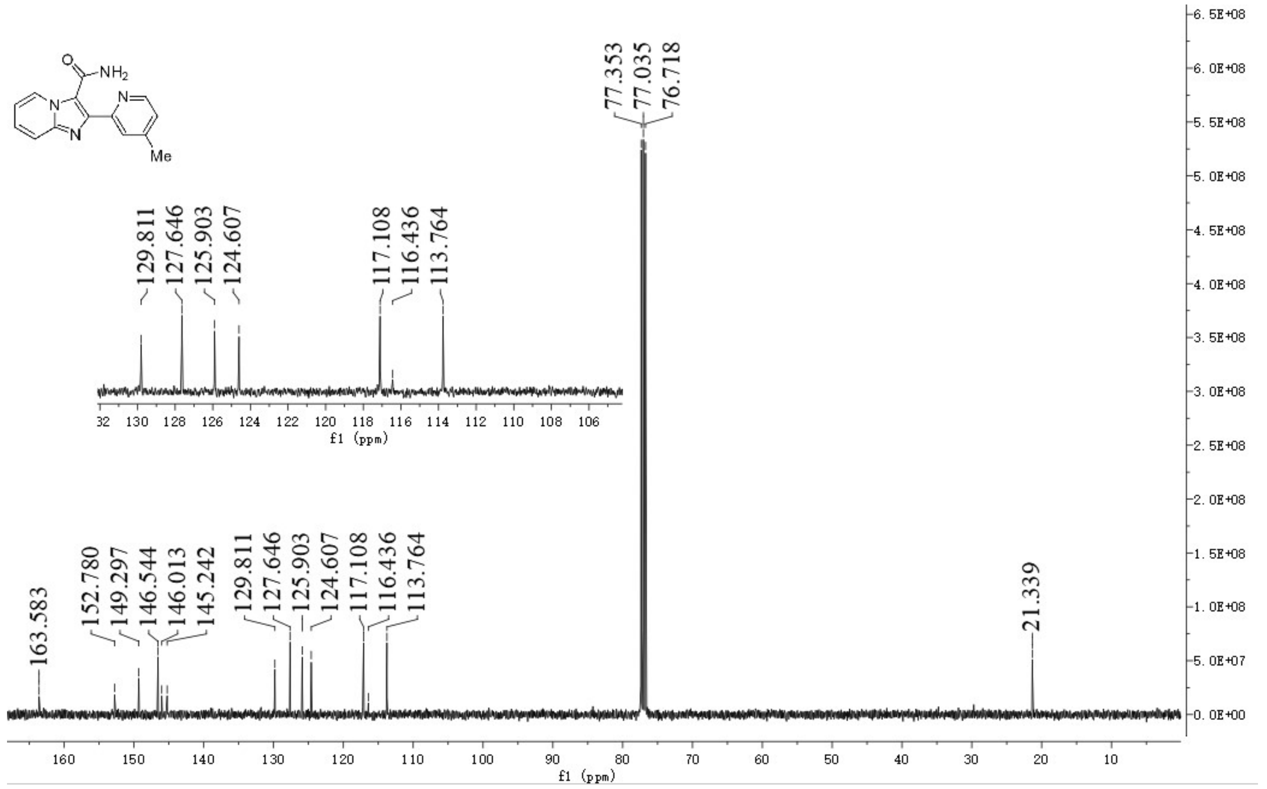

2-(4-Methoxypyridin-2-yl)-2,3-dihydroimidazo[1,2-*a*]pyridine-3-carboxamide (amide3)

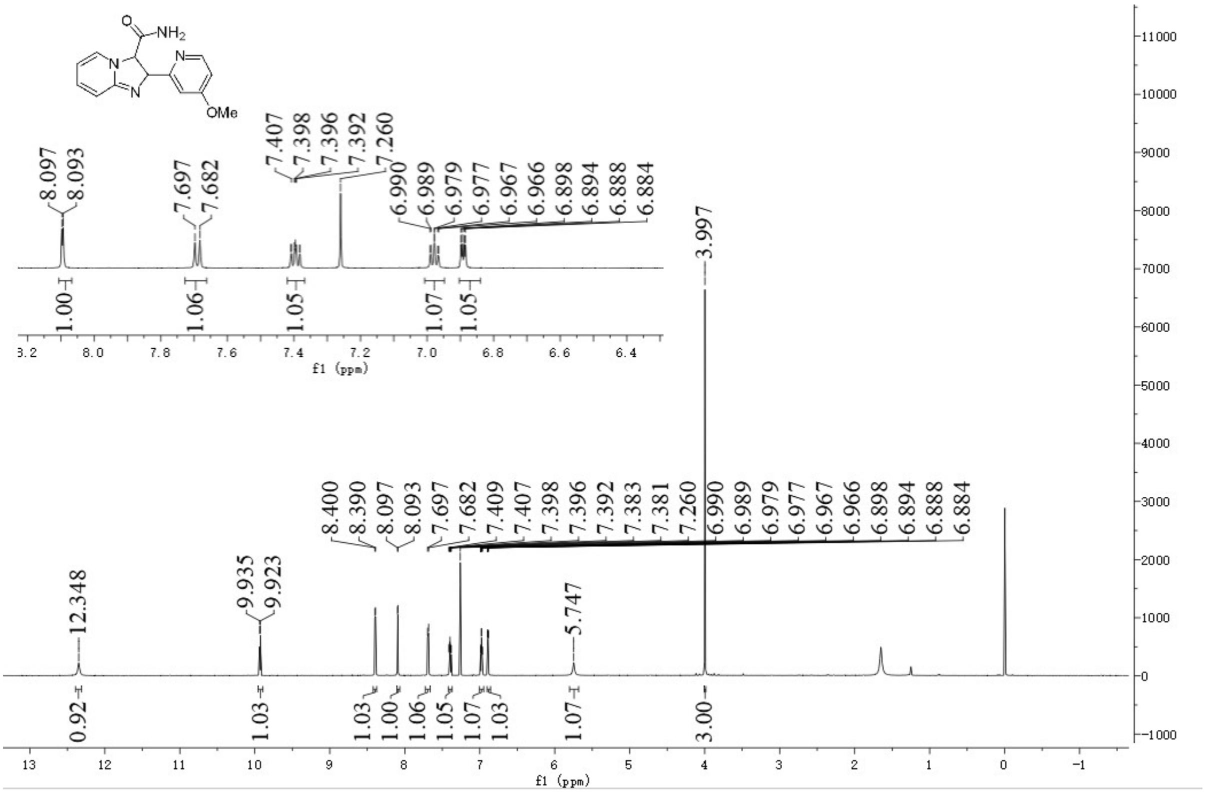

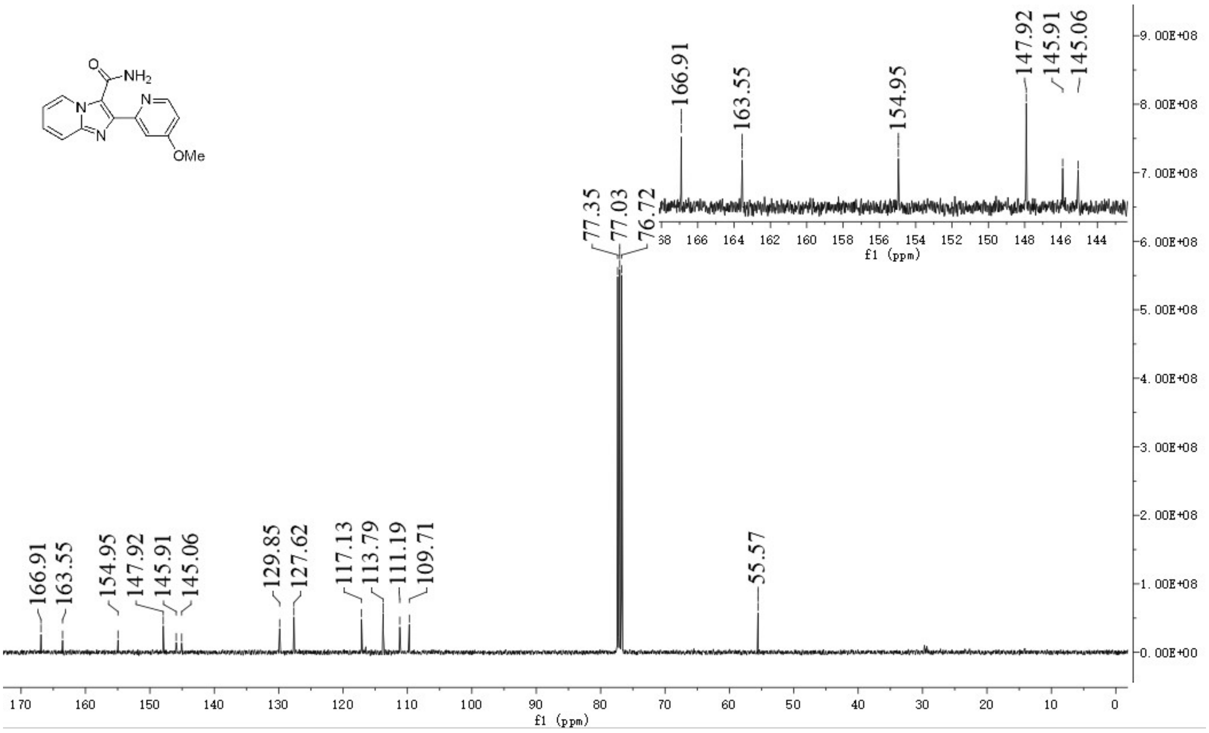

7-Methoxy-2-(4-methoxypyridin-2-yl)-2,3-dihydroimidazo[1,2-*a*]pyridine-3-carboxamide (amide4)

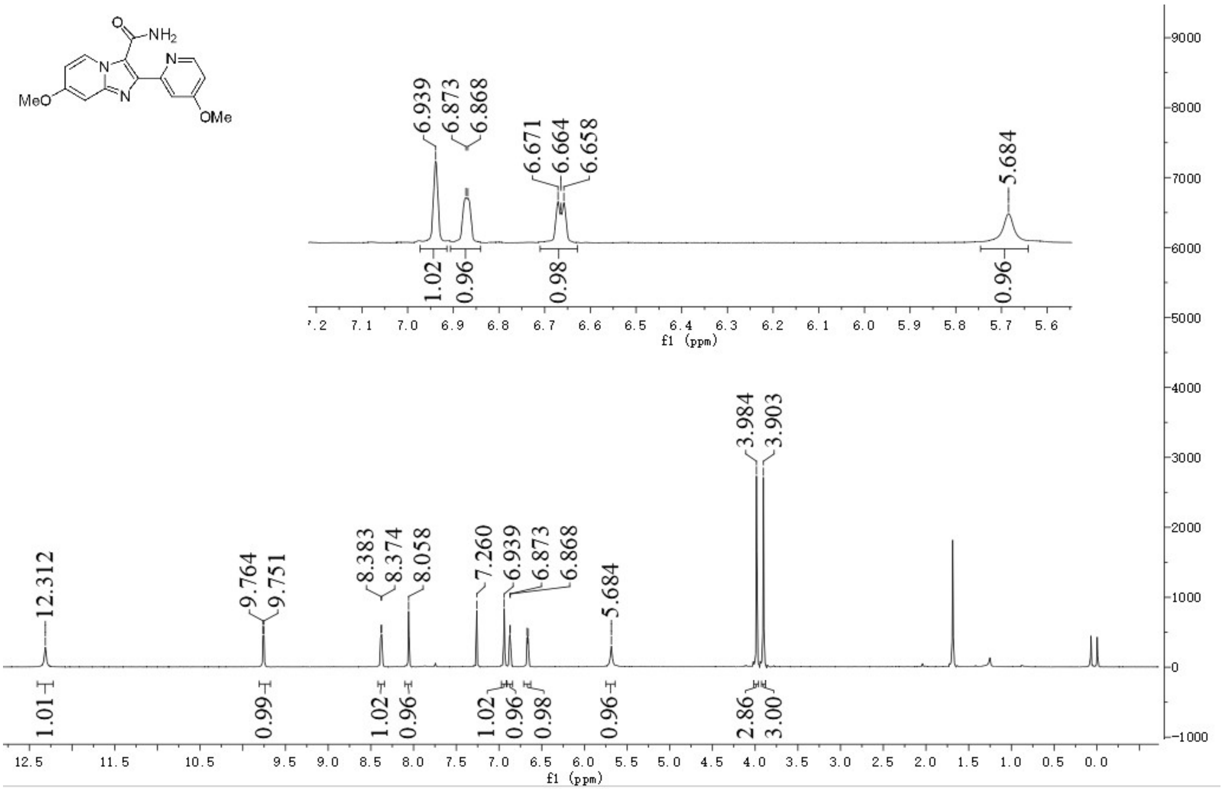

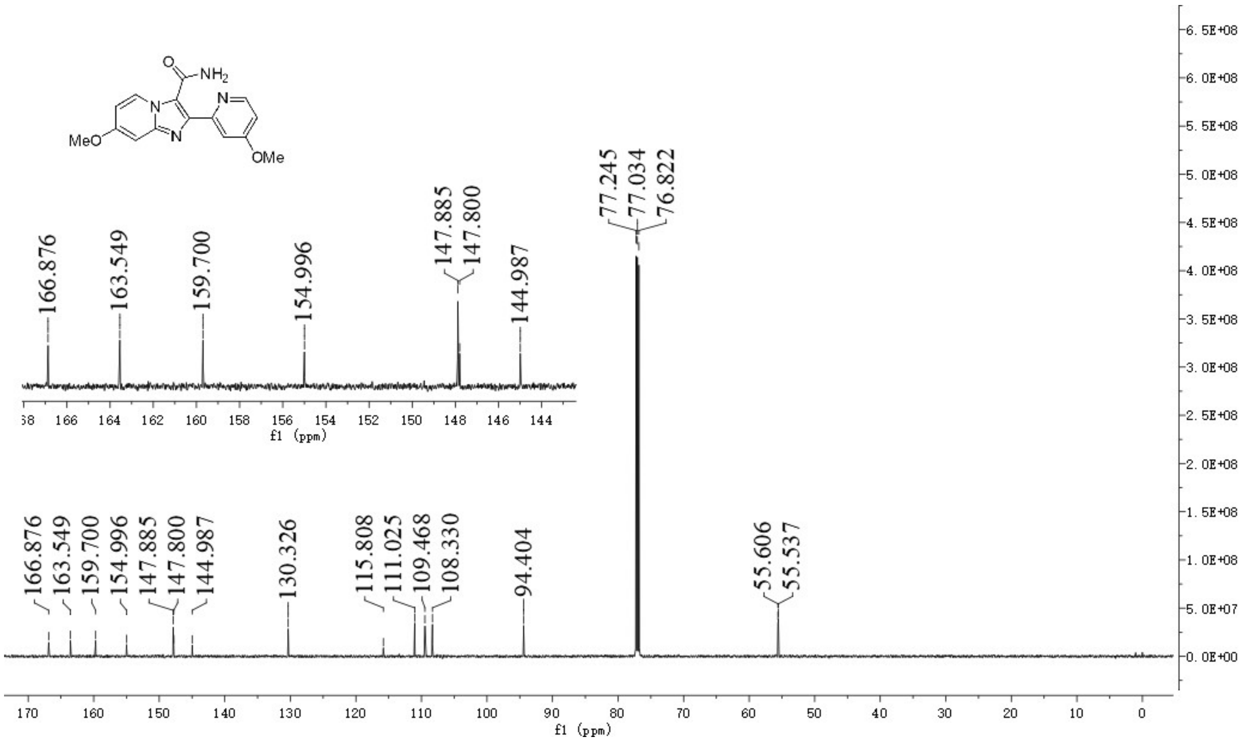

7-Cyano-2-(pyridin-2-yl)imidazo[1,2-a]pyridine-3-carboxamide (amide5)

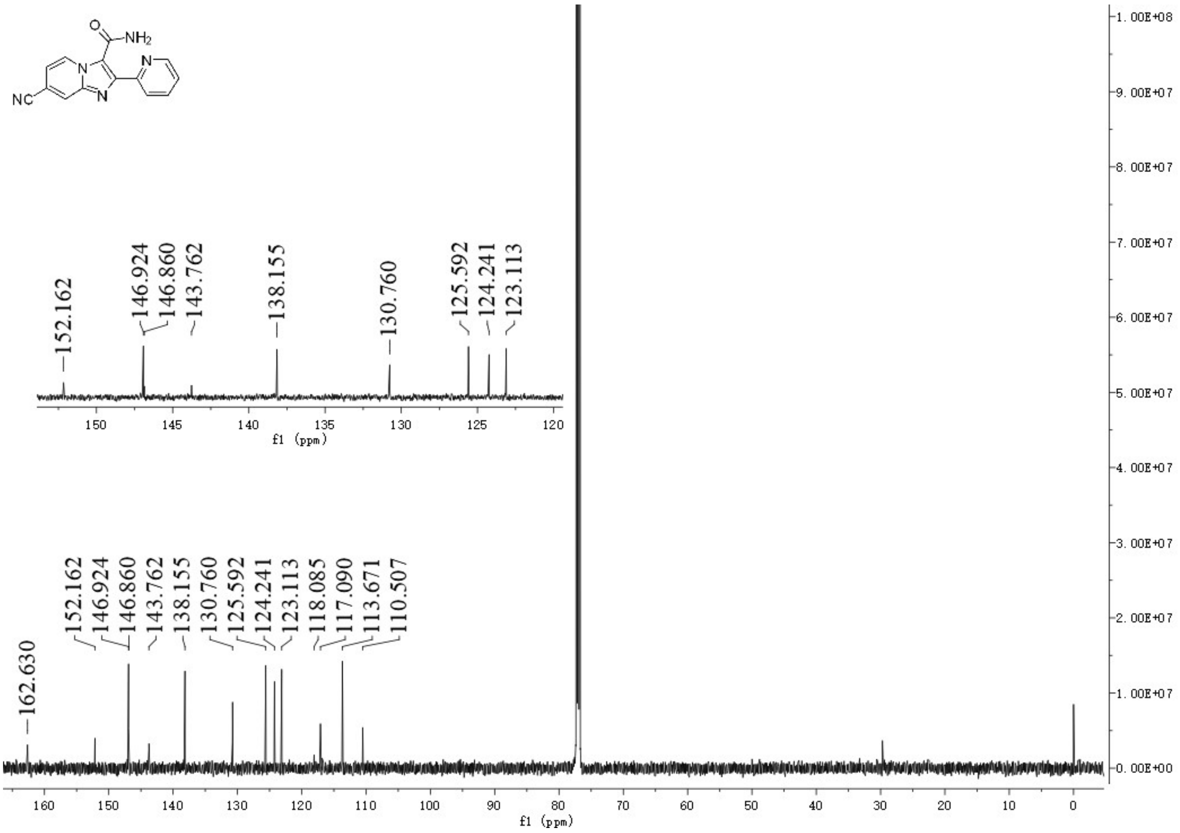

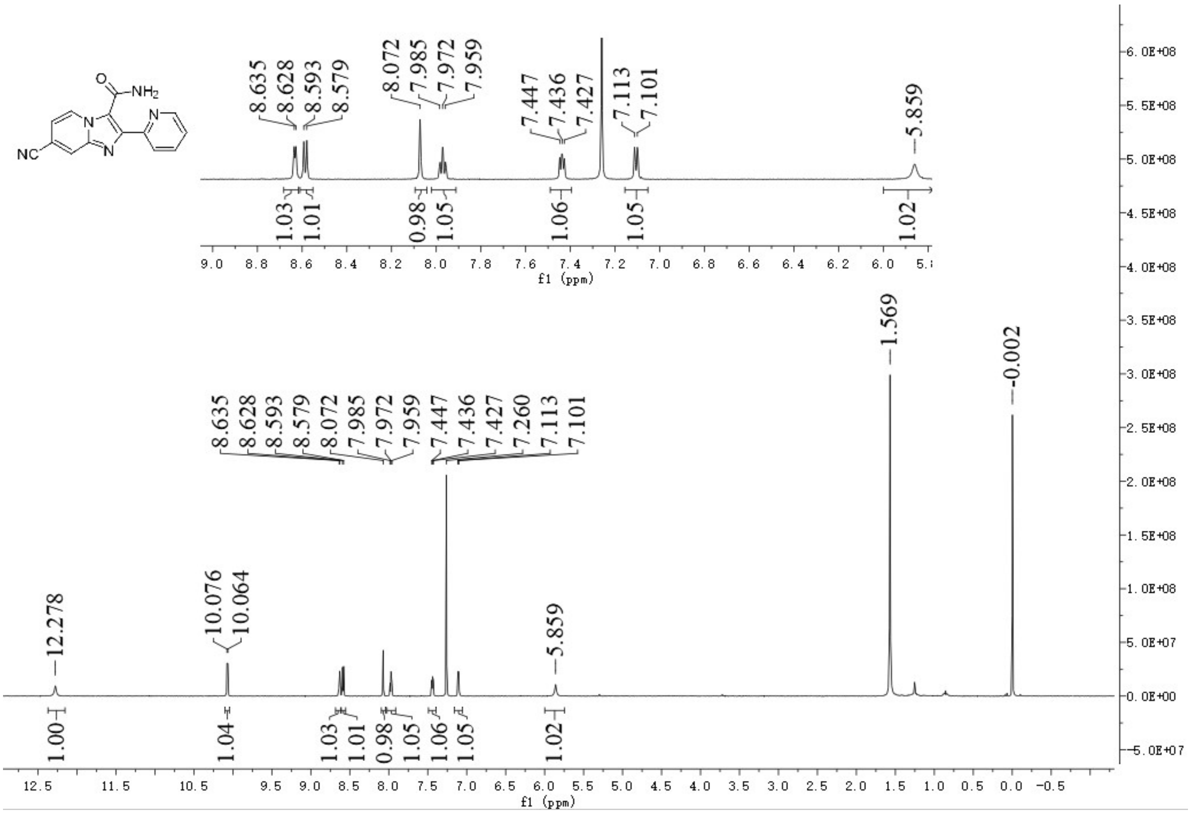

6*H*-Dipyrido[1,2-*e*:2',1'-*i*]purin-6-one (DP1)

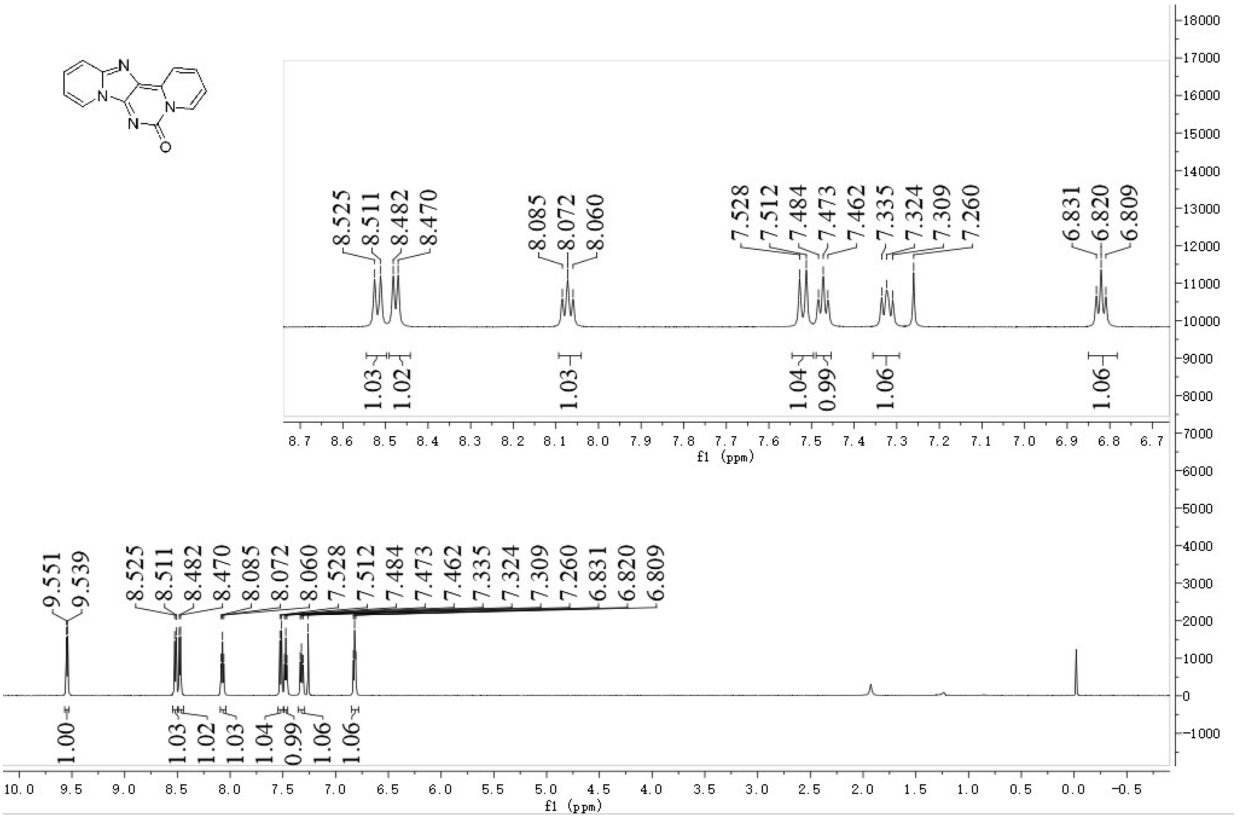

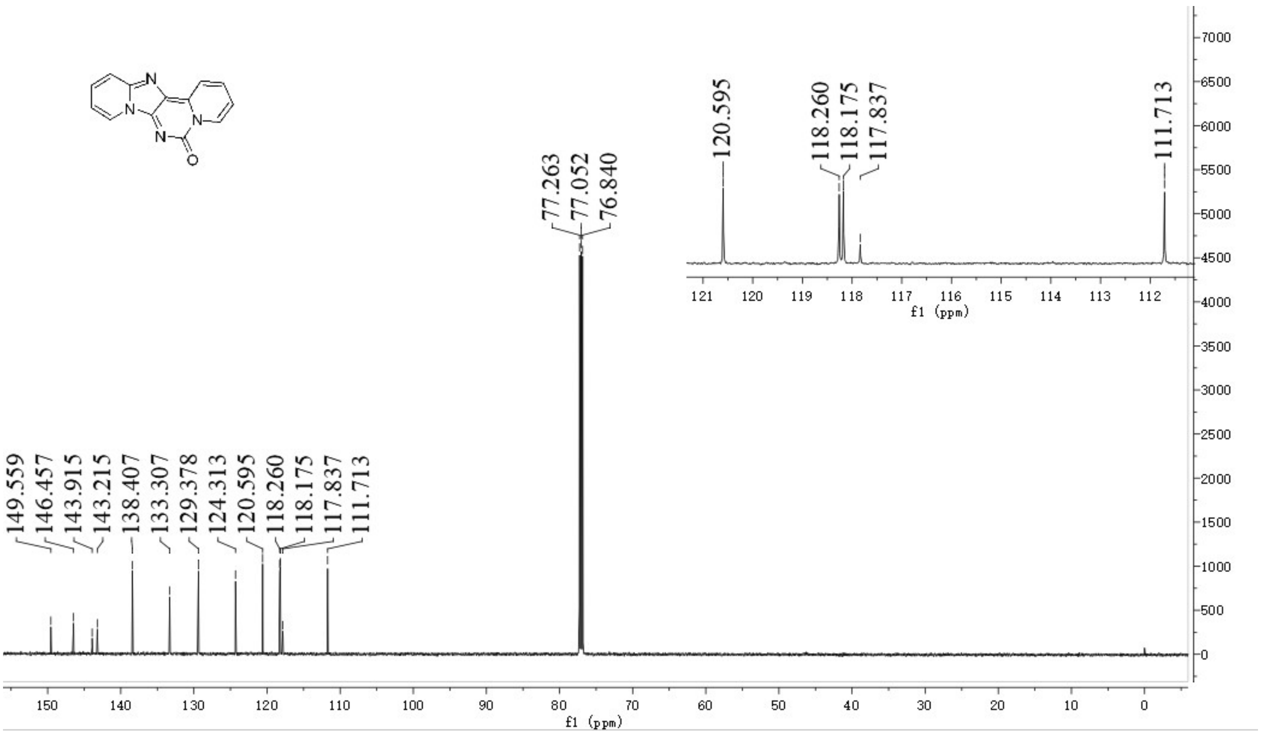

2-Methyl-6*H*-dipyrido[1,2-*e*:2',1'-*i*]purin-6-one (DP2)

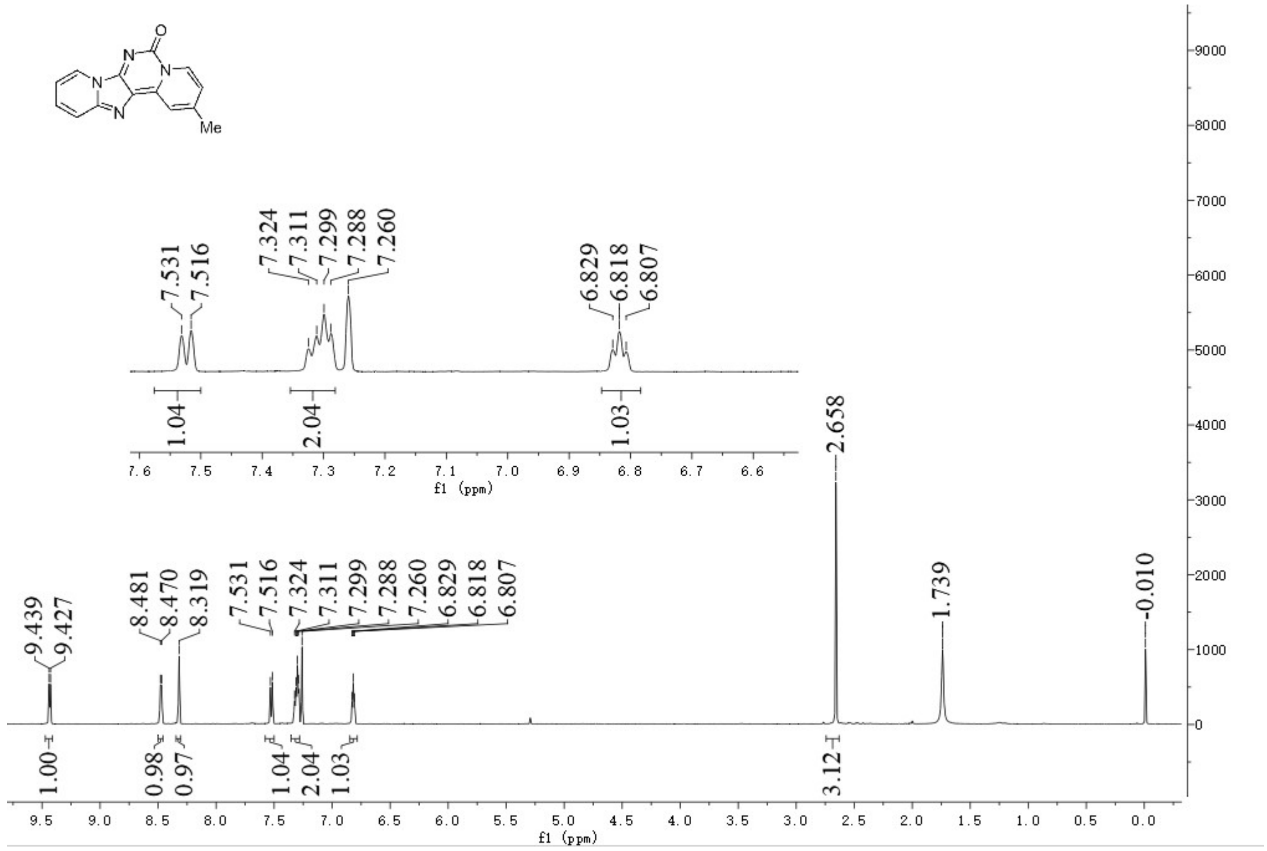

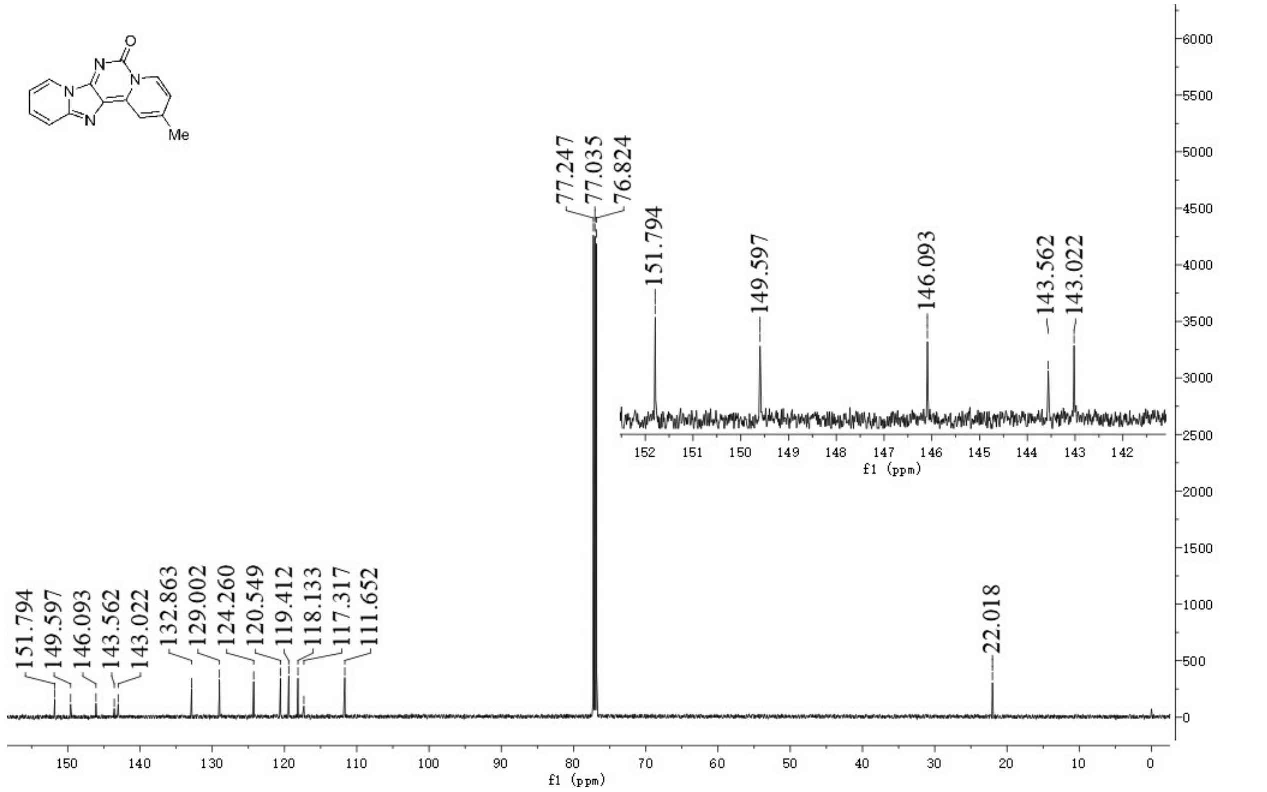

2-Methoxy-6*H*-dipyrido[1,2-*e*:2',1'-*i*]purin-6-one (DP3)

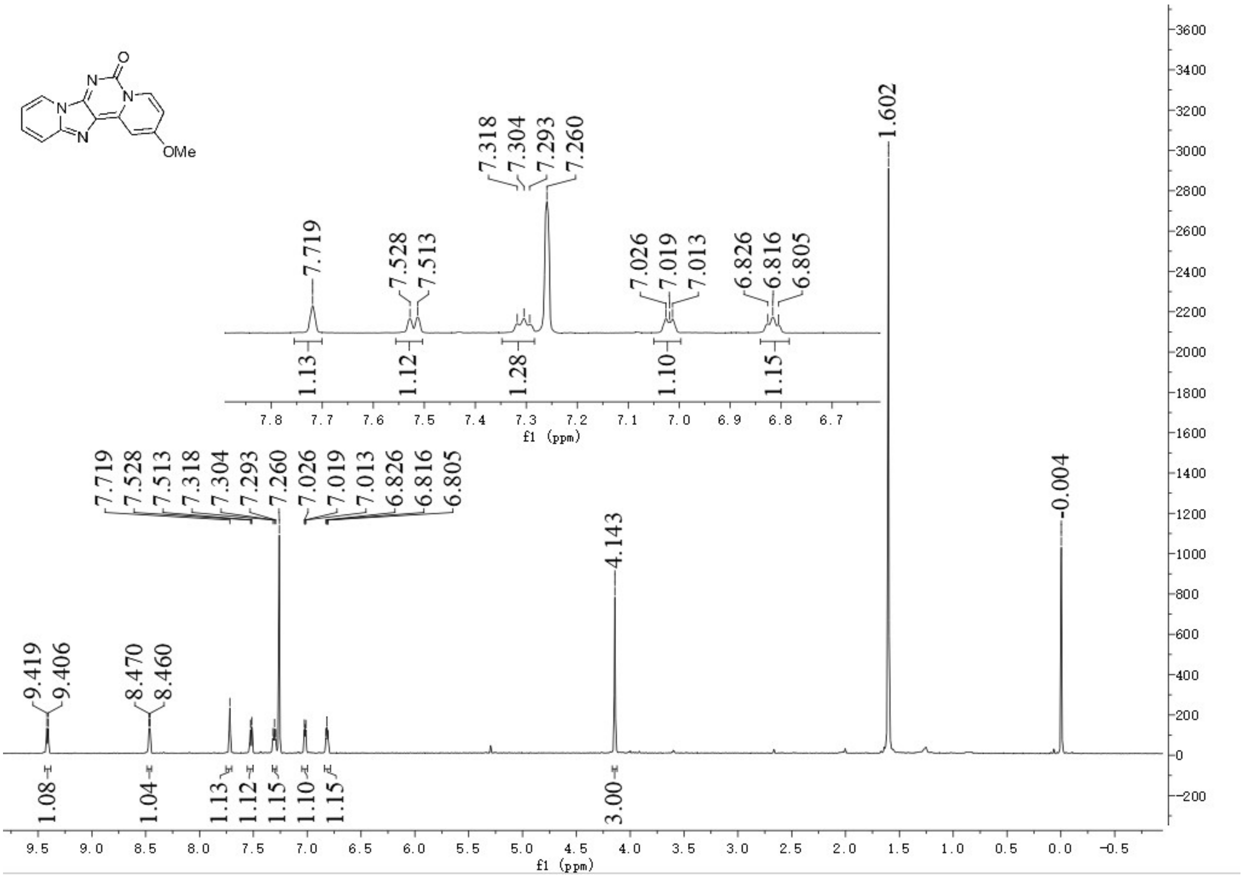

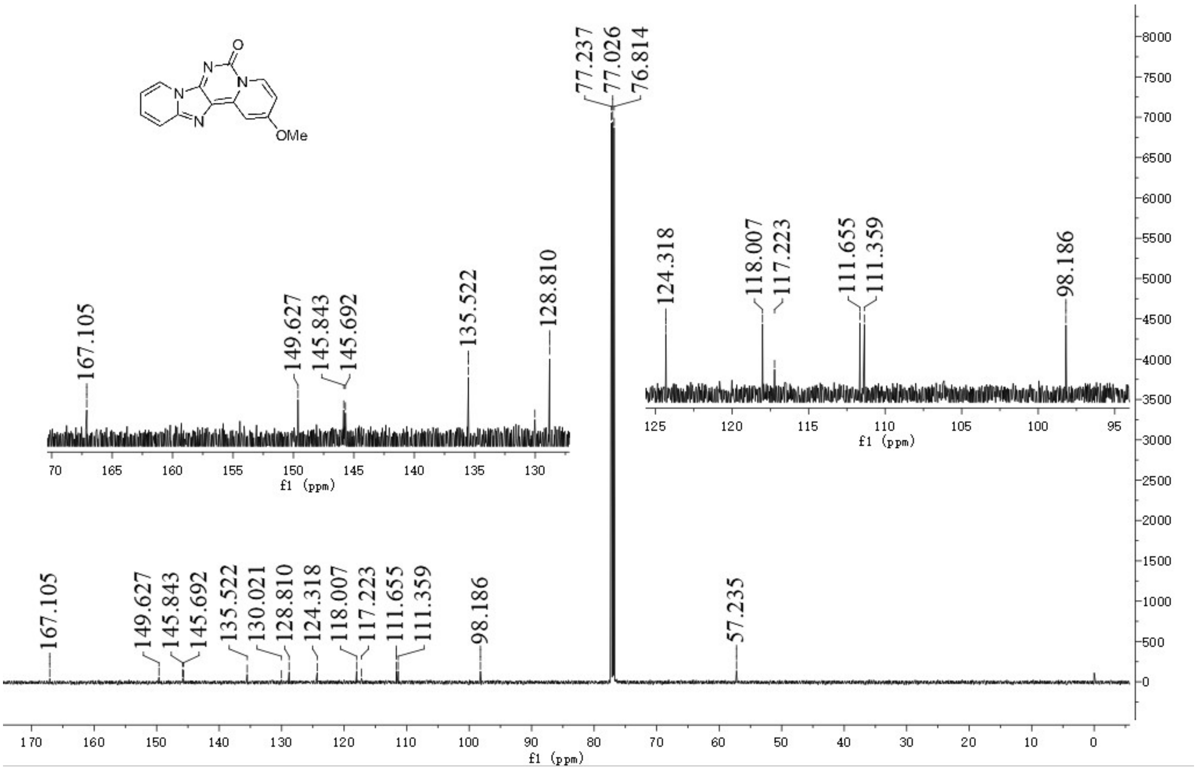

2,11-Dimethoxy-6*H*-dipyrido[1,2-*e*:2',1'-*i*]purin-6-one (DP4)

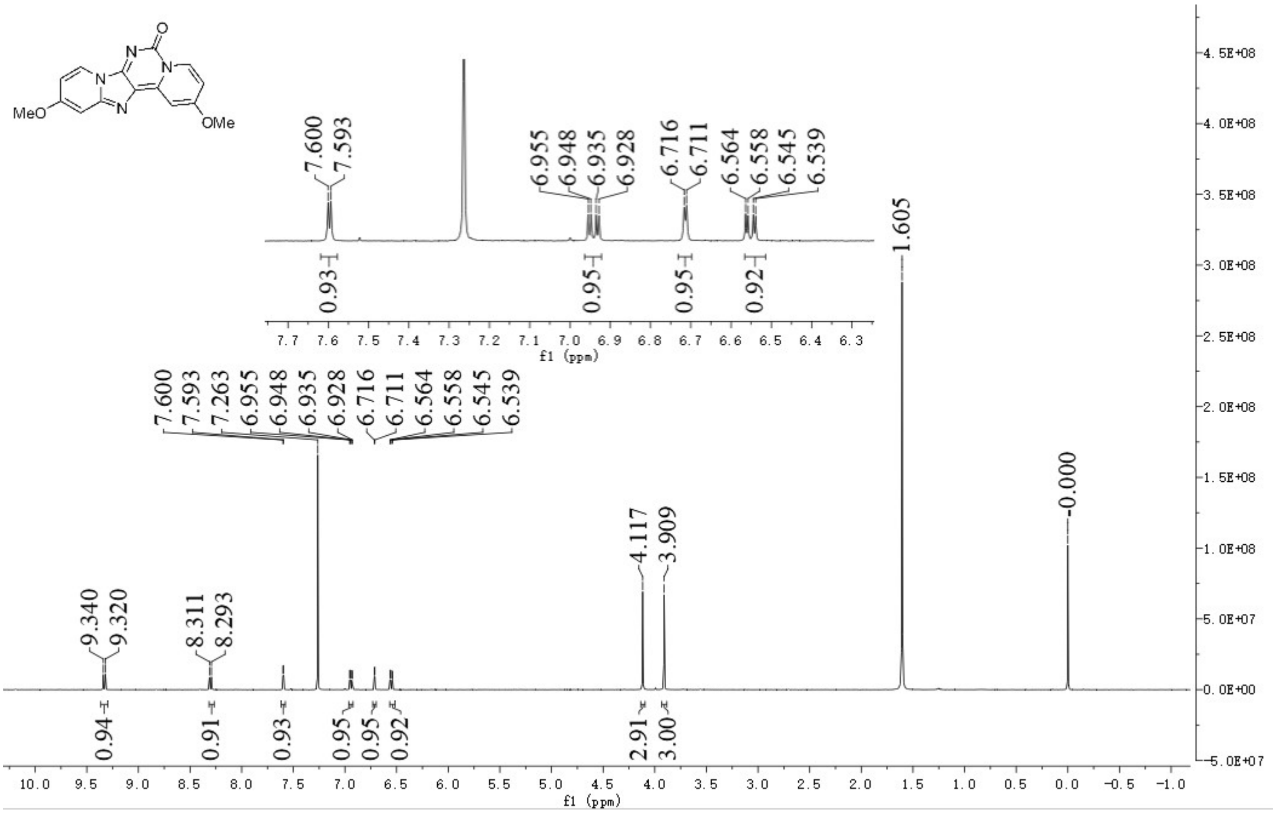

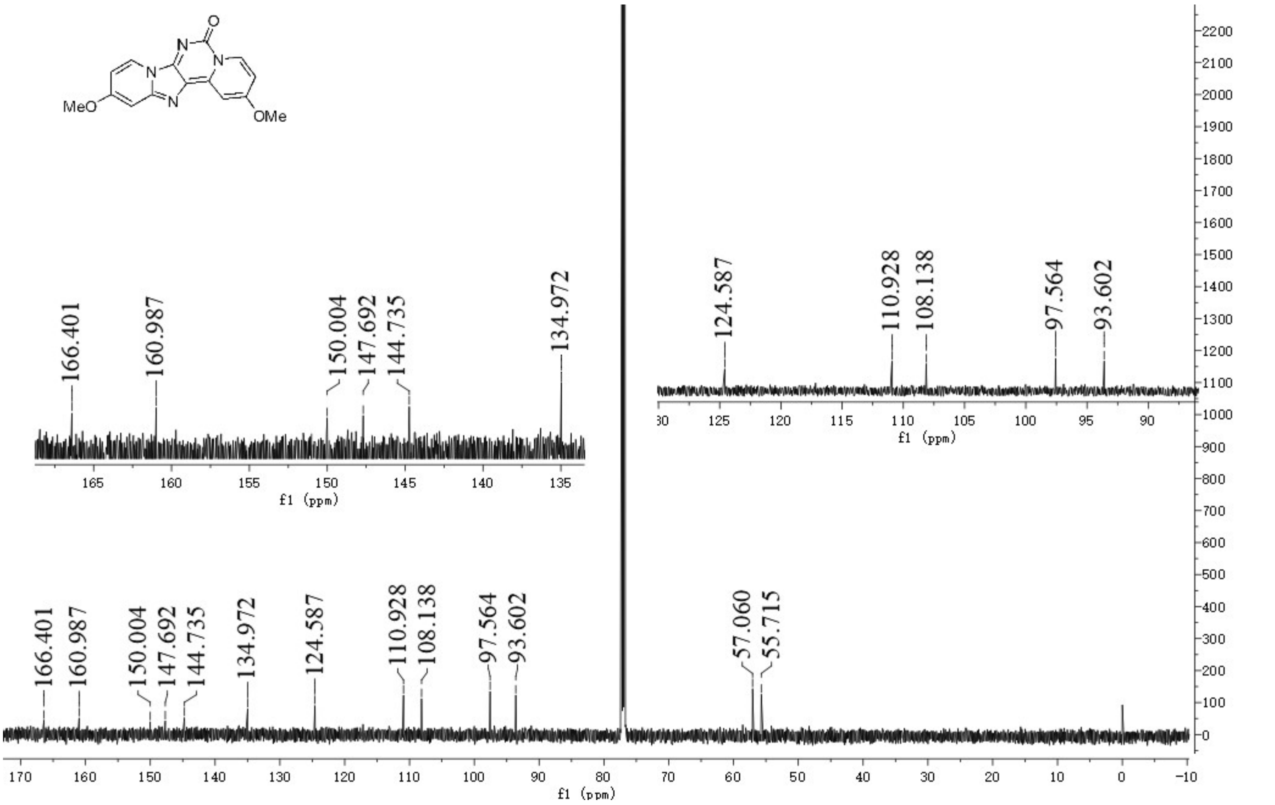

6-Oxo-6*H*-dipyrido[1,2-e:2',1'-i]purine-11-carbonitrile (DP5)

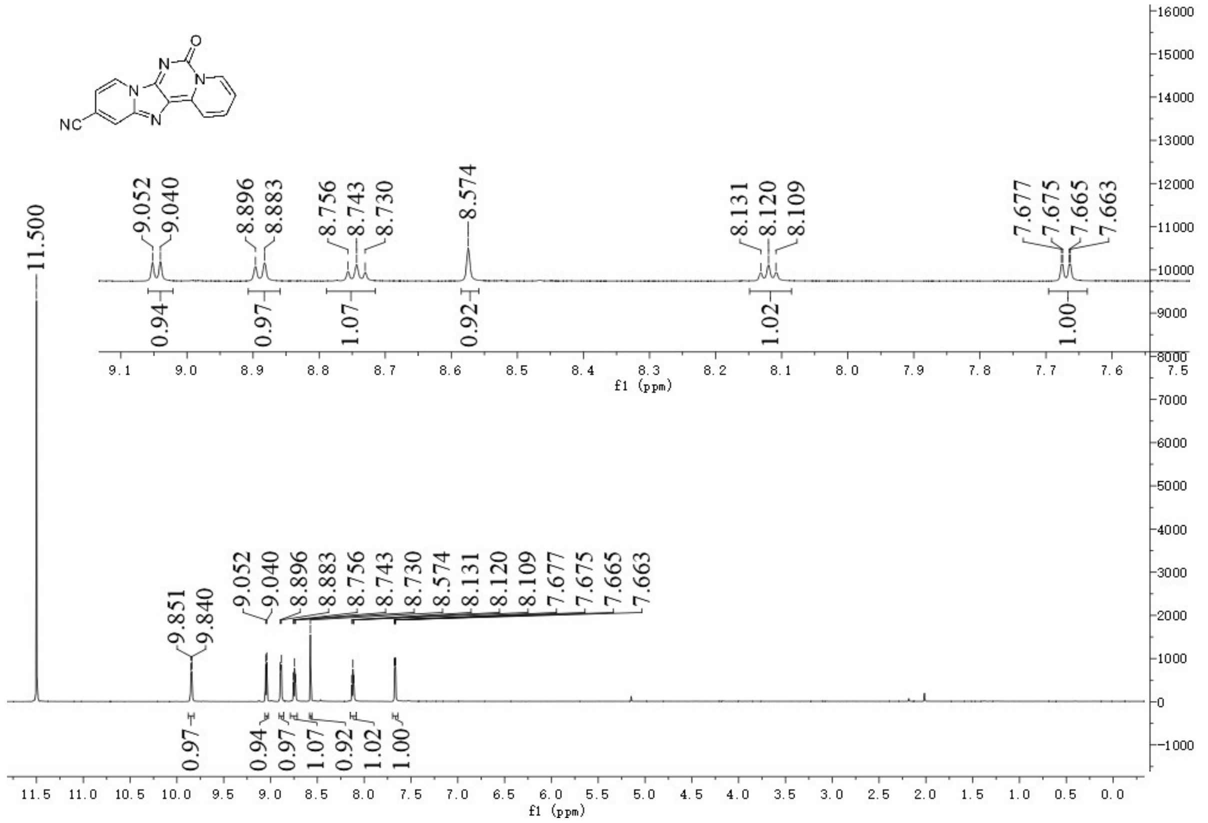

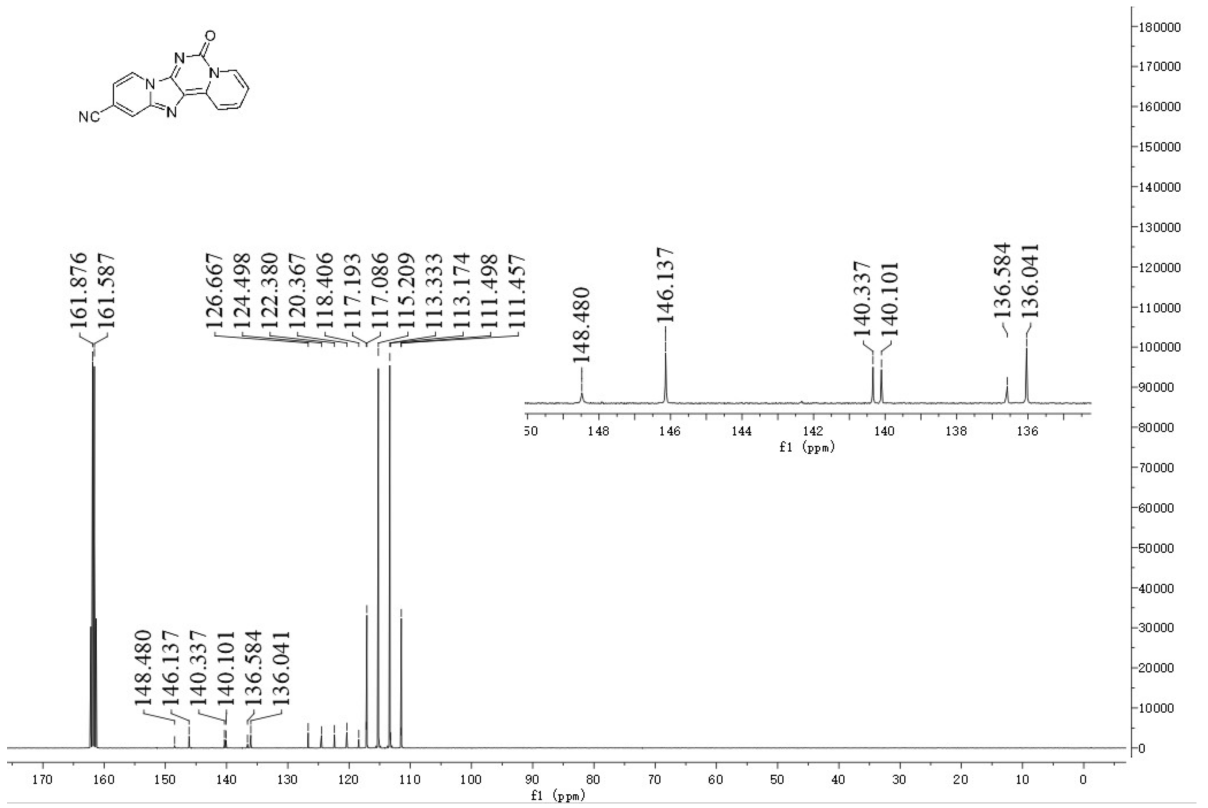

***N*,*N′*-Diphenylmorpholine-4-carboxamidamide (1)**

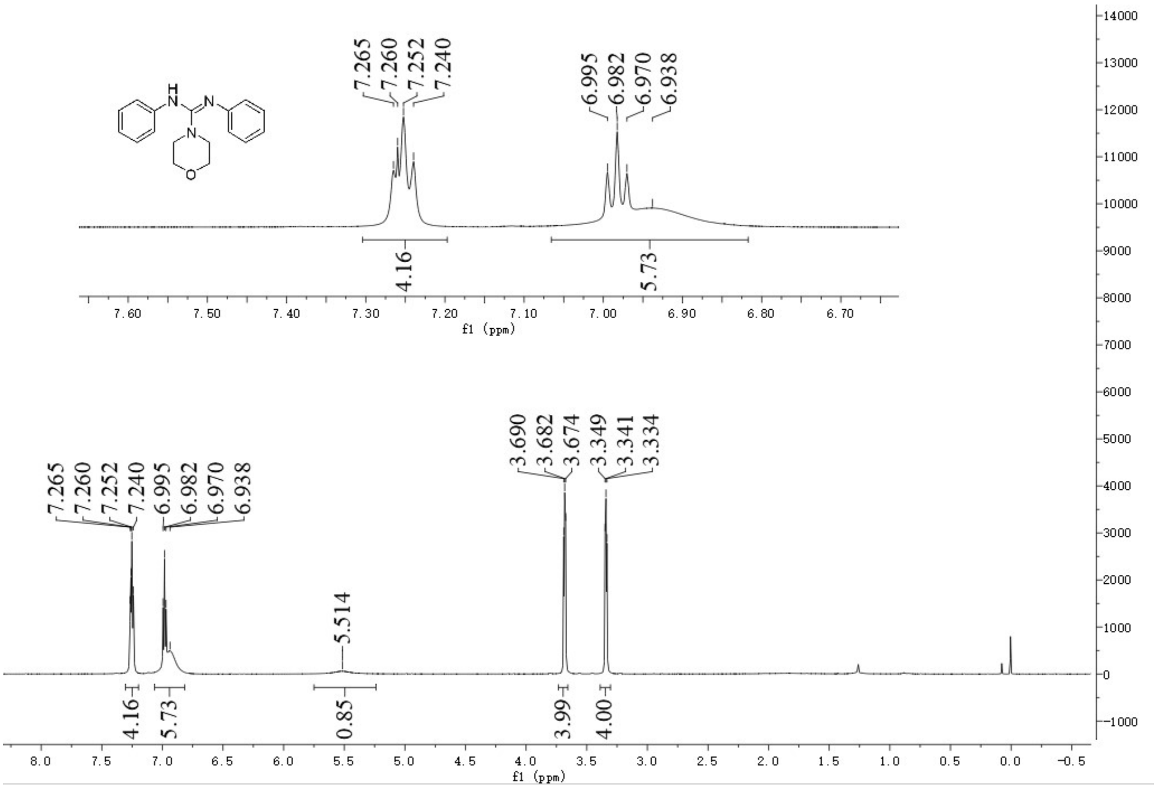

***N*-Phenyl-*N′*-(*o*-tolyl)morpholine-4-carboxamidamide (2)**

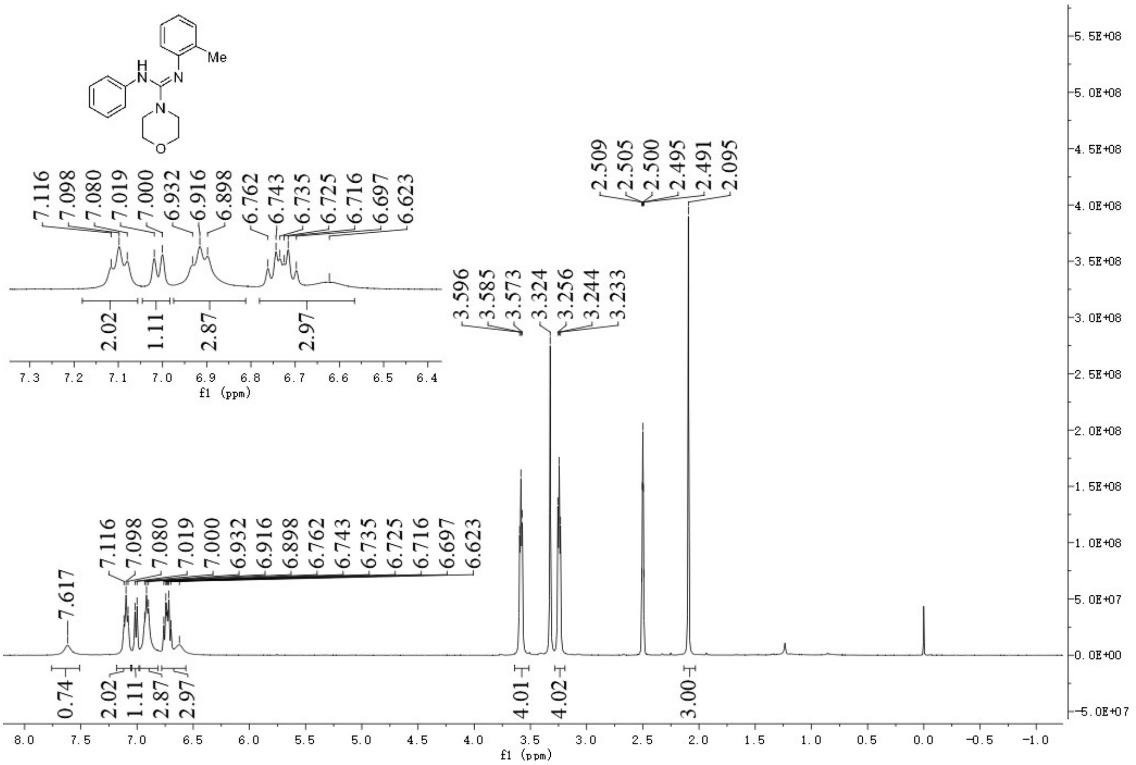

N-Phenyl-N′-(m-tolyl)morpholine-4-carboxamidamide (3)

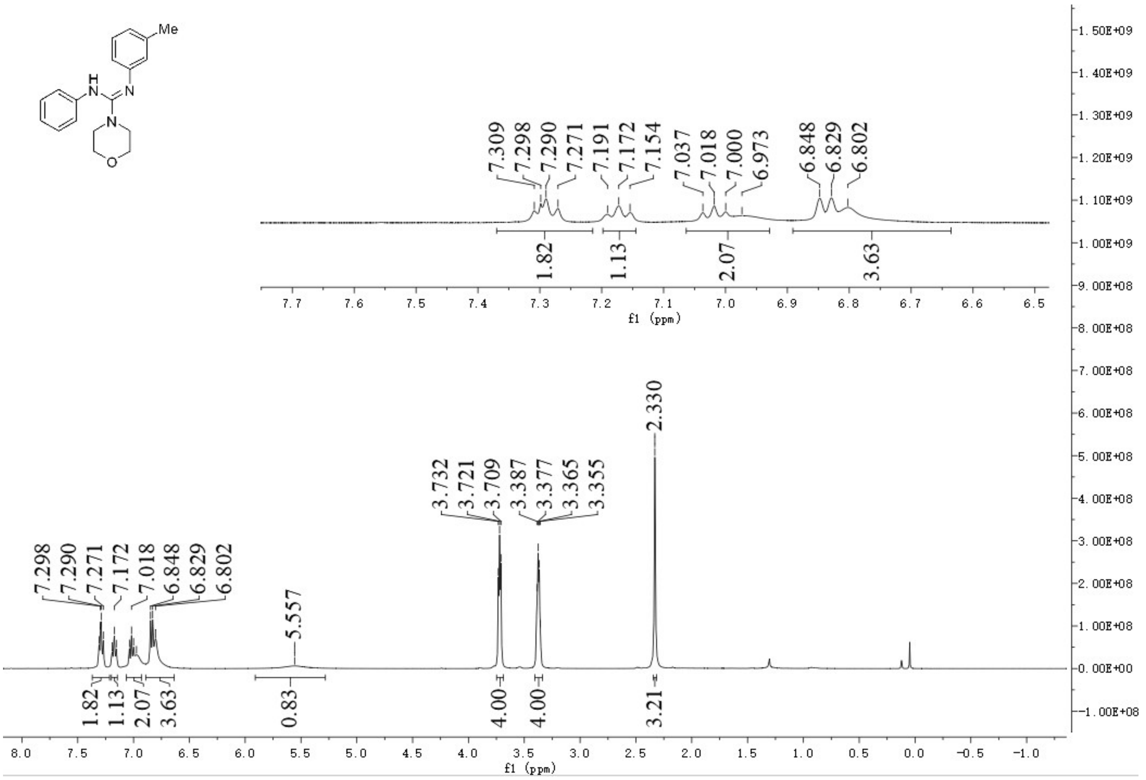

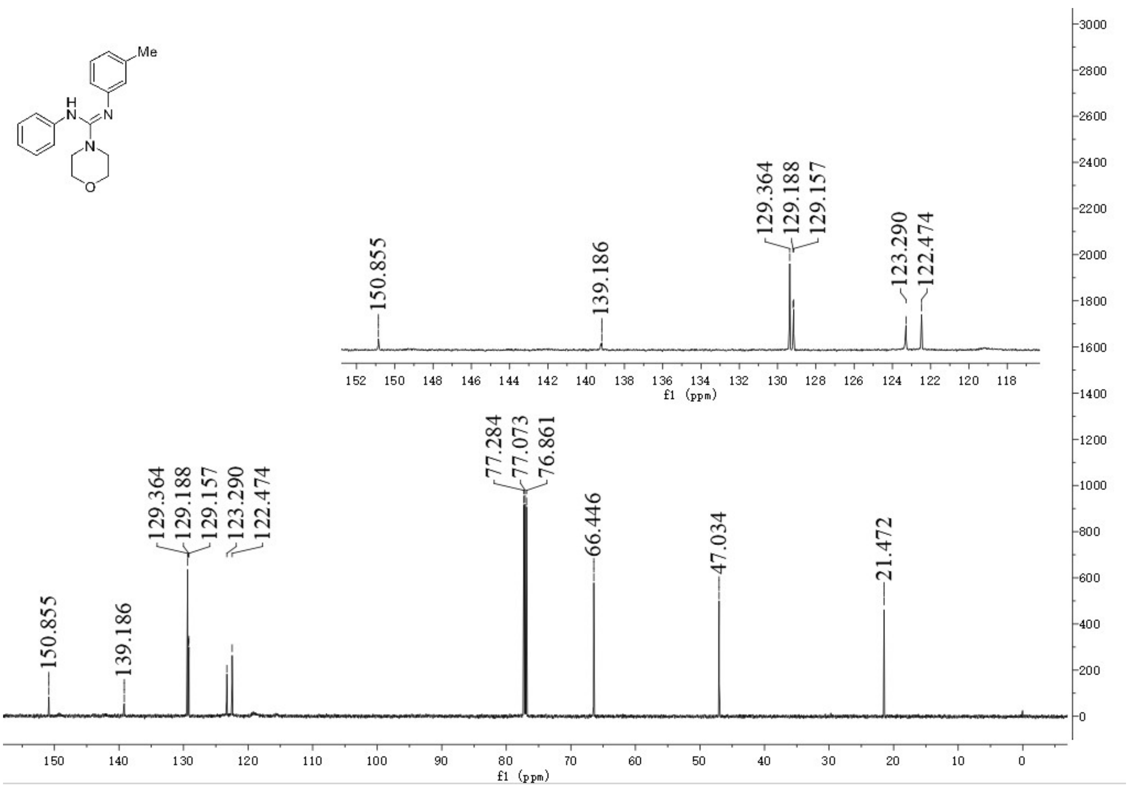

N-Phenyl-*N′*-(*p*-tolyl)morpholine-4-carboximidamide (4)

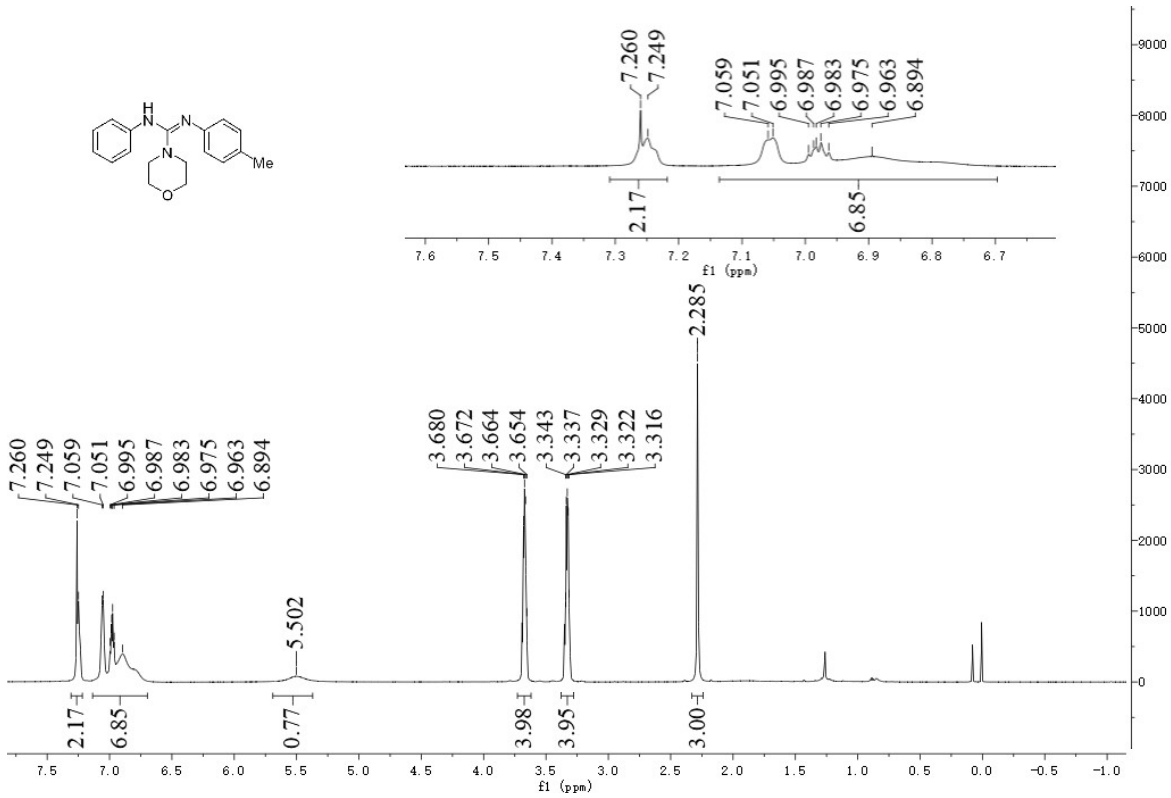

N′-(4-Methoxyphenyl)-*N*-phenylmorpholine-4-carboximidamide (5)

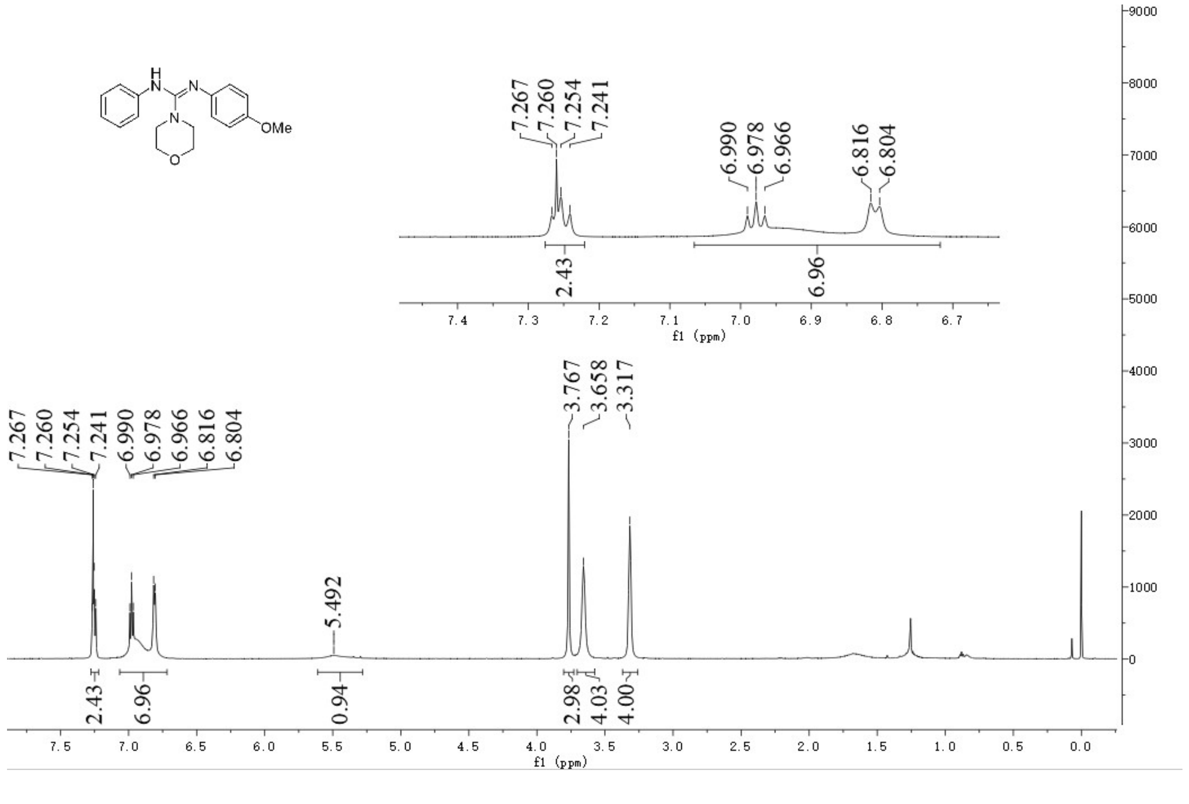

N′-(4-Chlorophenyl)-*N*-phenylmorpholine-4-carboximidamide (6)

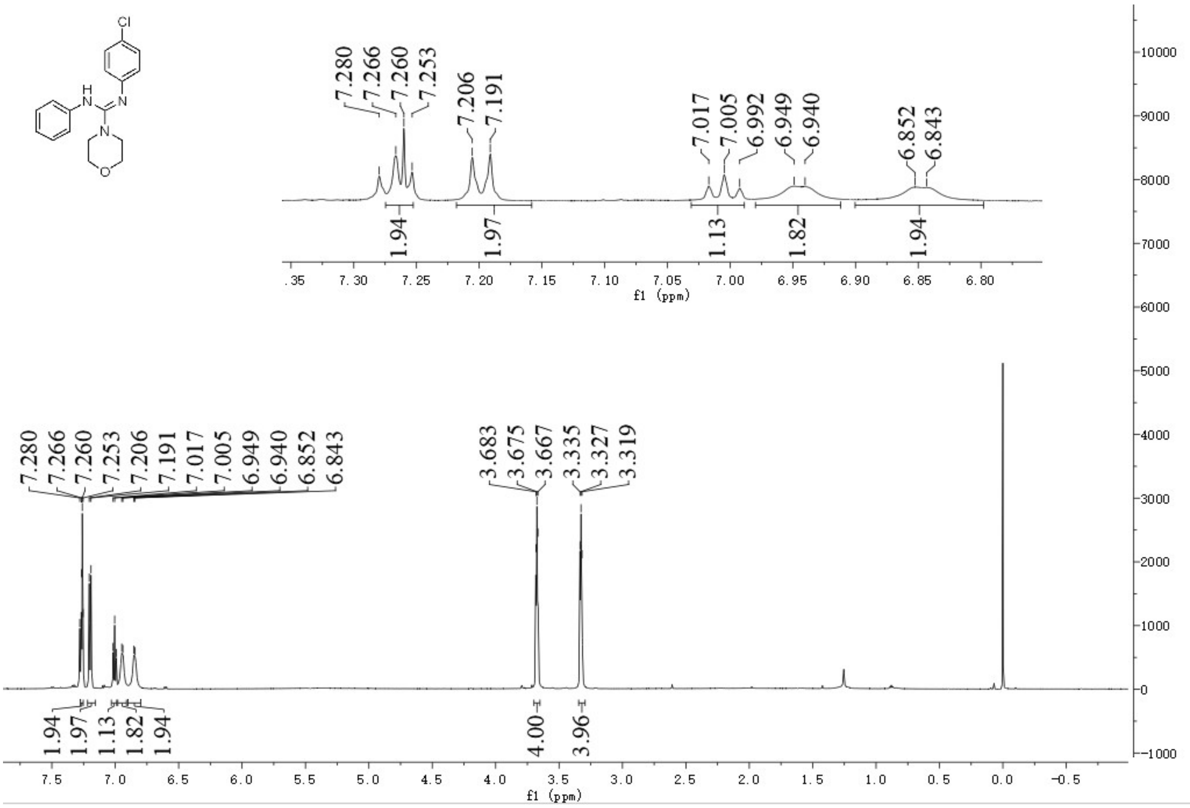

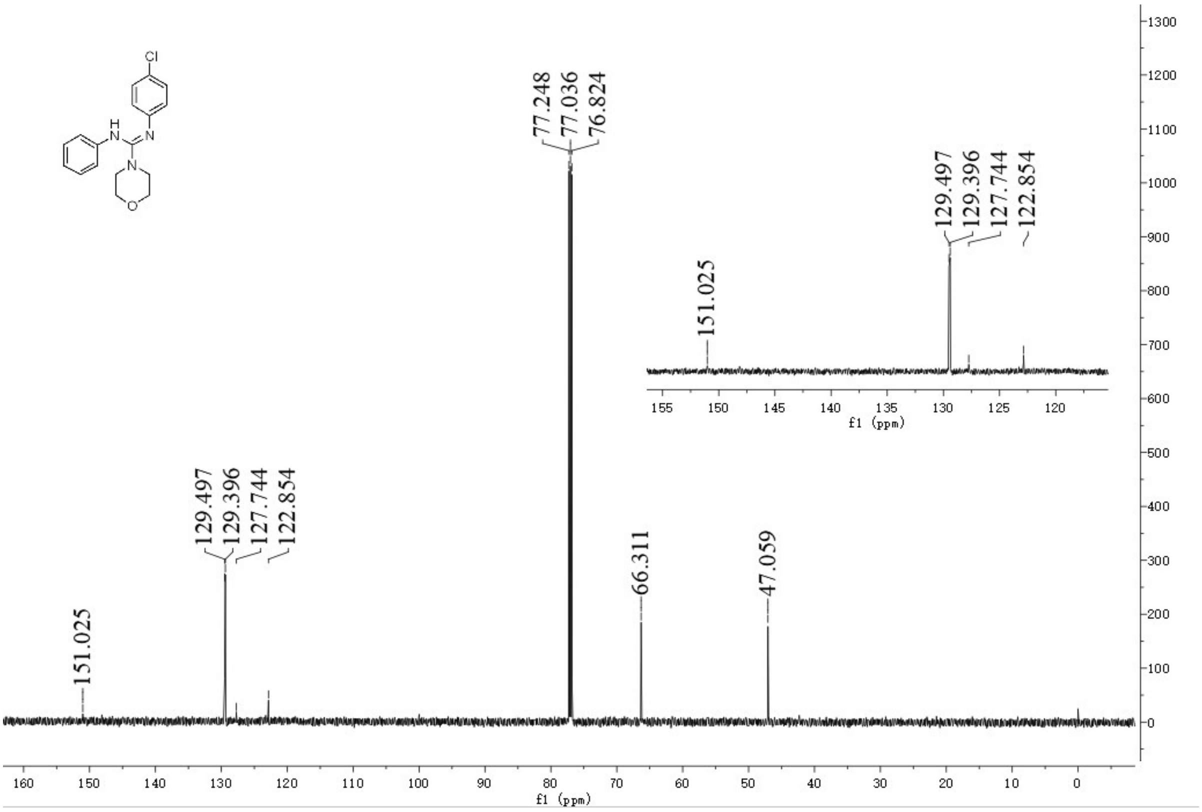

_N′_-(4-Bromophenyl)-_N_-phenylmorpholine-4-carboximidamide (7)

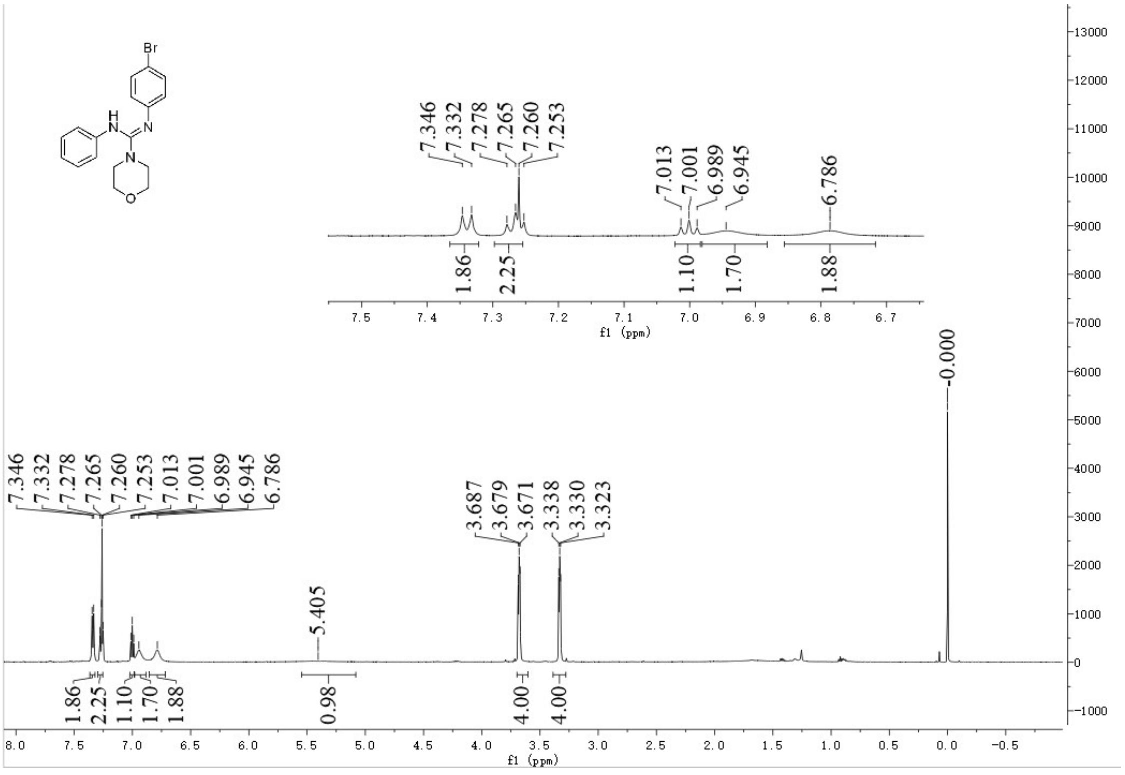

Ethyl -4-((morpholino(phenylamino)methylene)amino)benzoate (8)

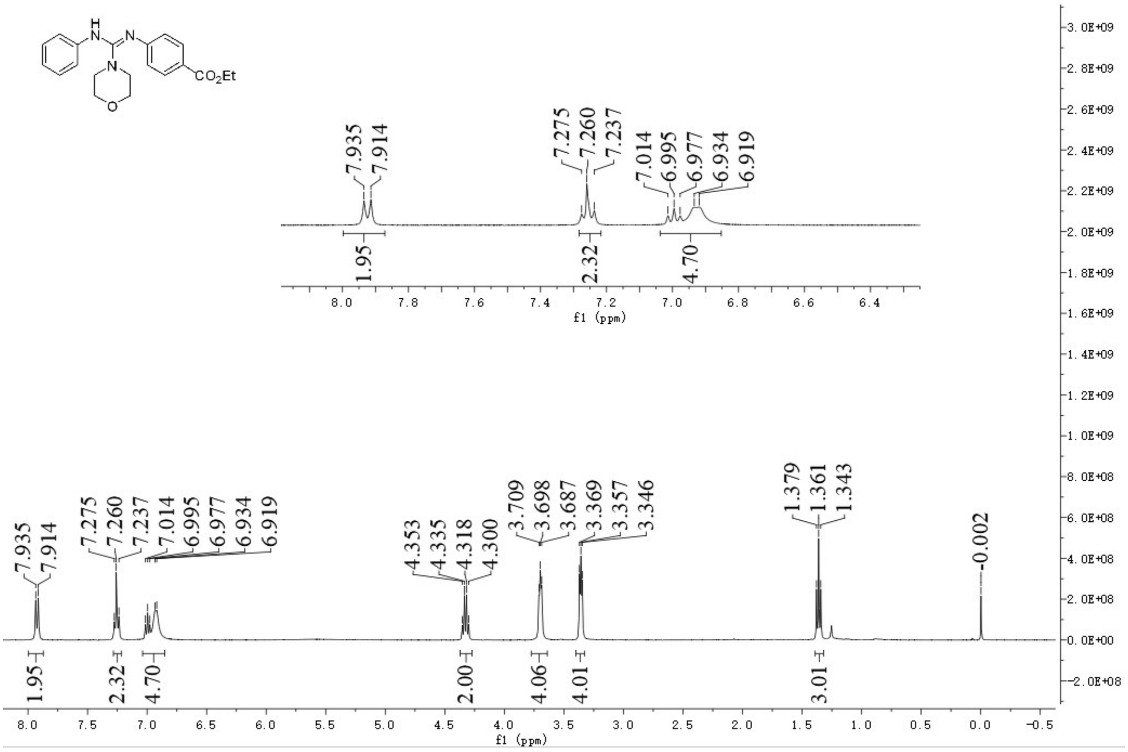

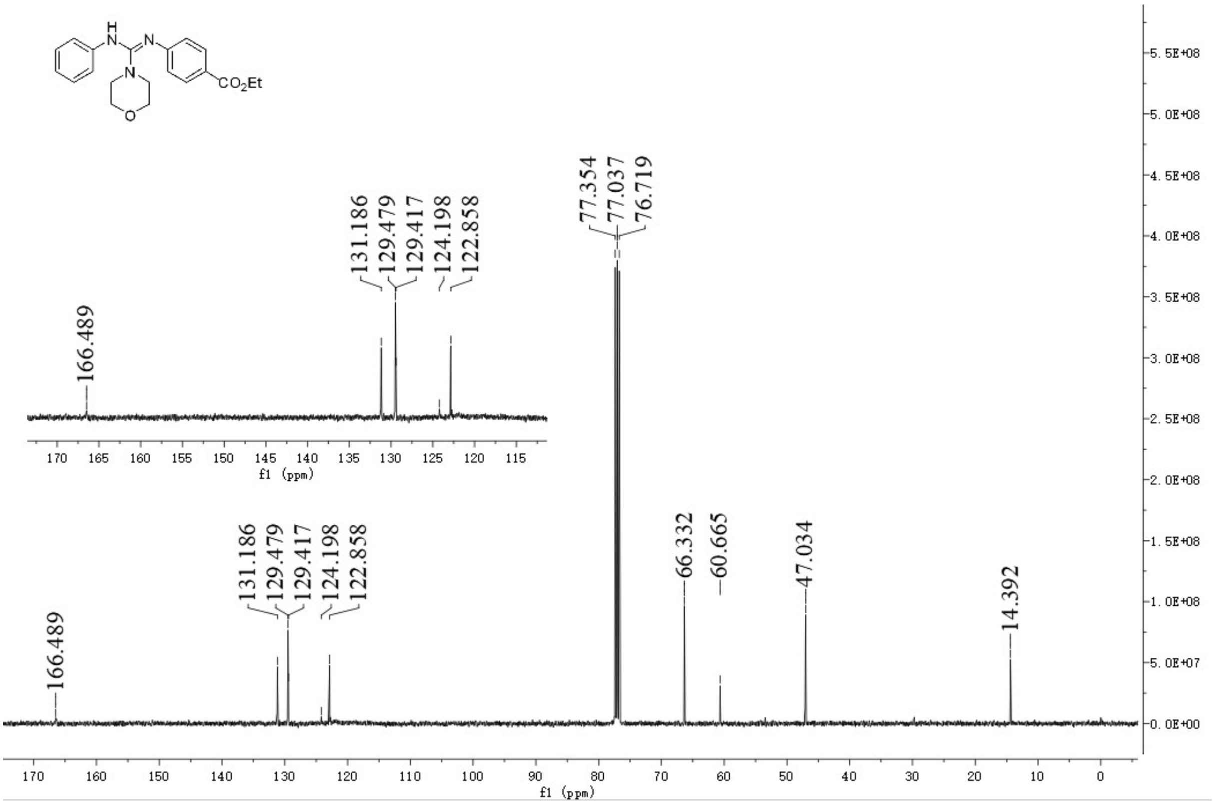

N-Phenyl-N′-(pyridin-2-yl)morpholine-4-carboximidamide (9)

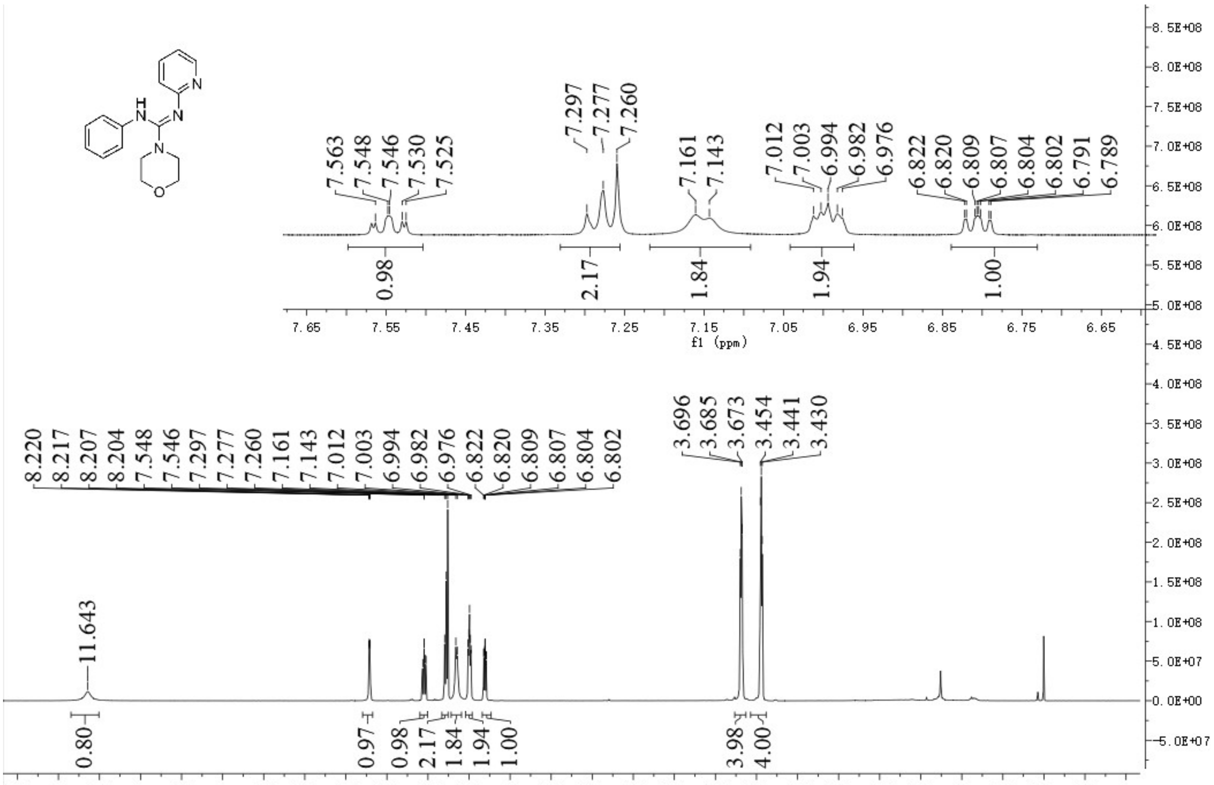

2-Hydroxy-5-((morpholino(phenylamino)methylene)amino)benzoic acid (10)

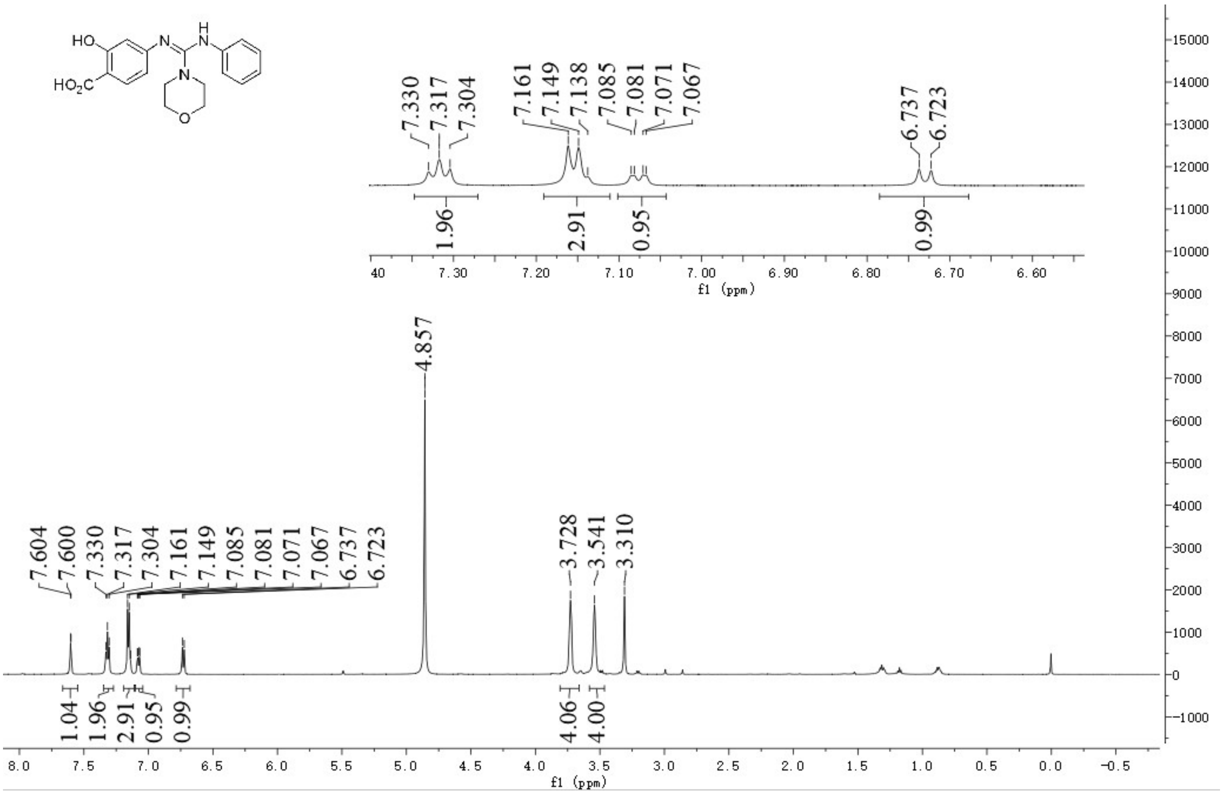

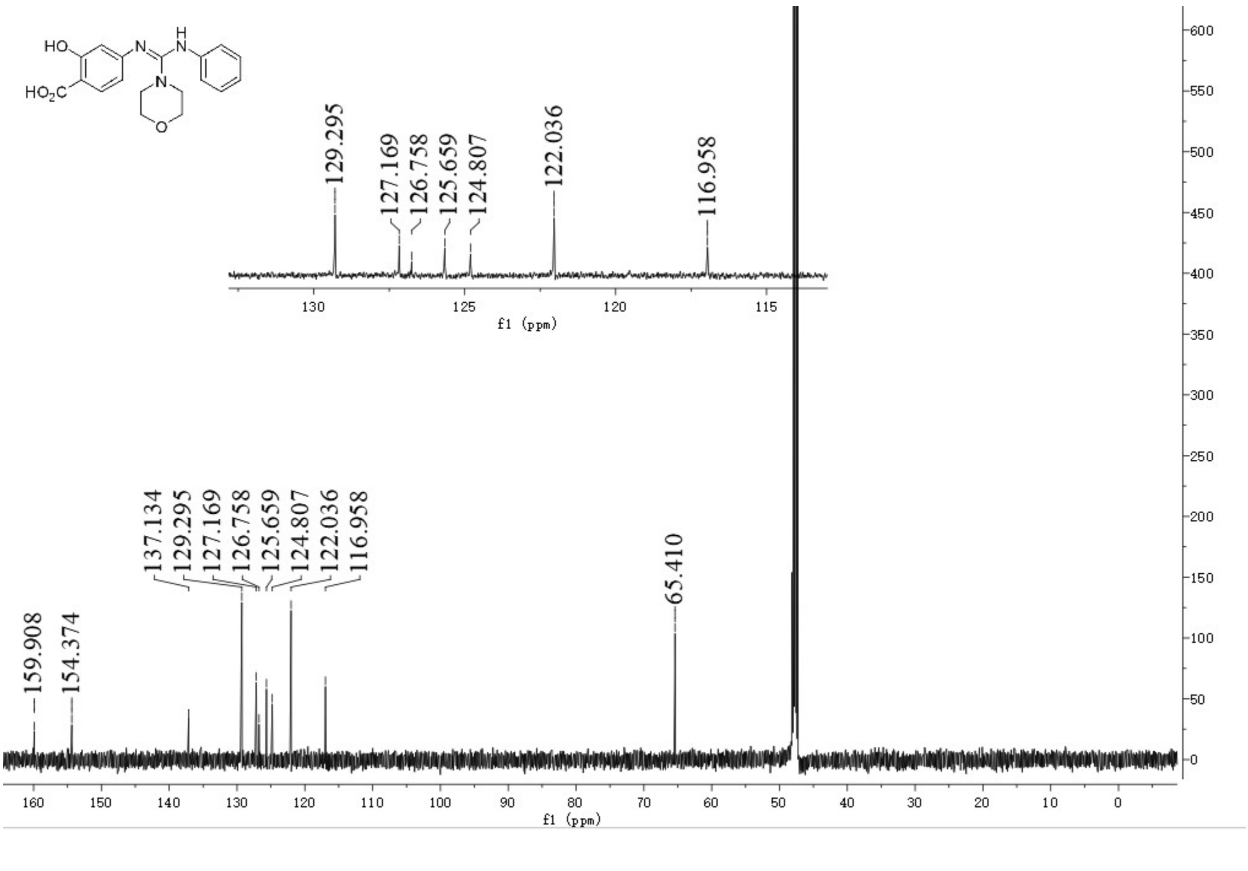

***N*-(Tert-butyl)-*N'*-phenylmorpholine-4-carboximidamide (11)**

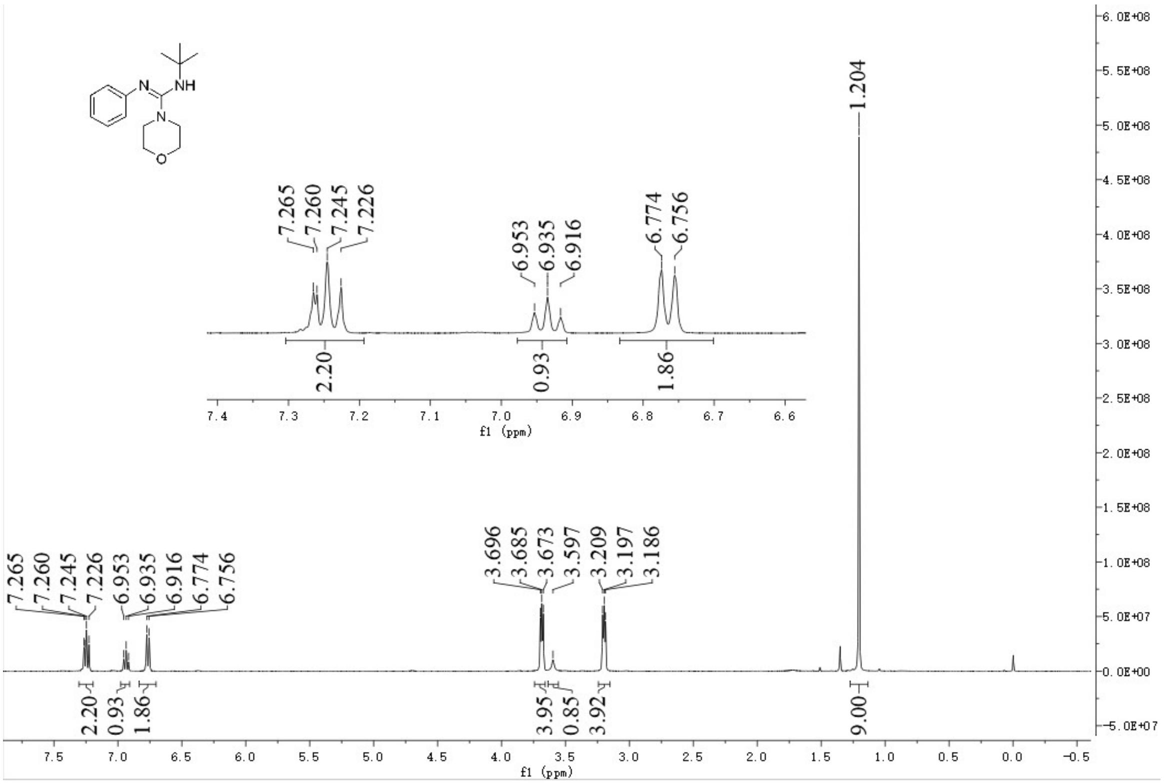

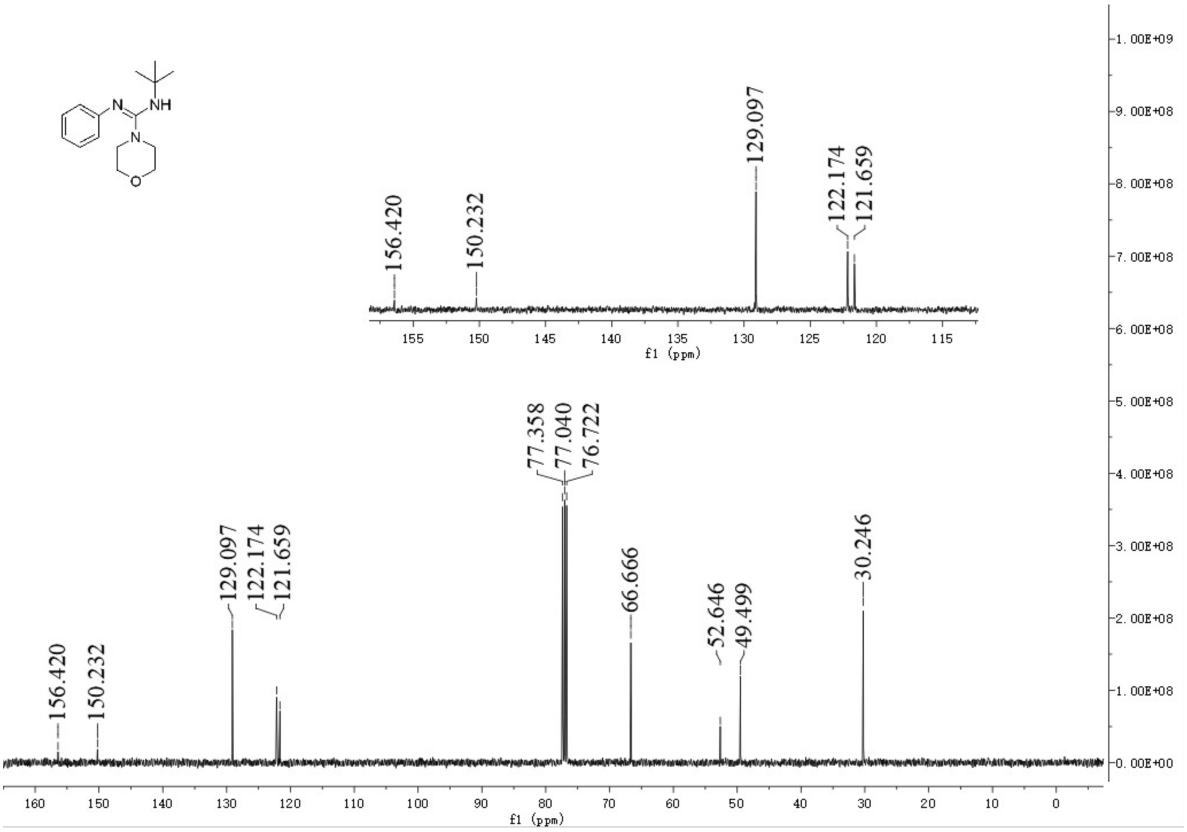

2-Cyclohexyl-1-morpholino-*N*-phenylethan-1-imine (12)

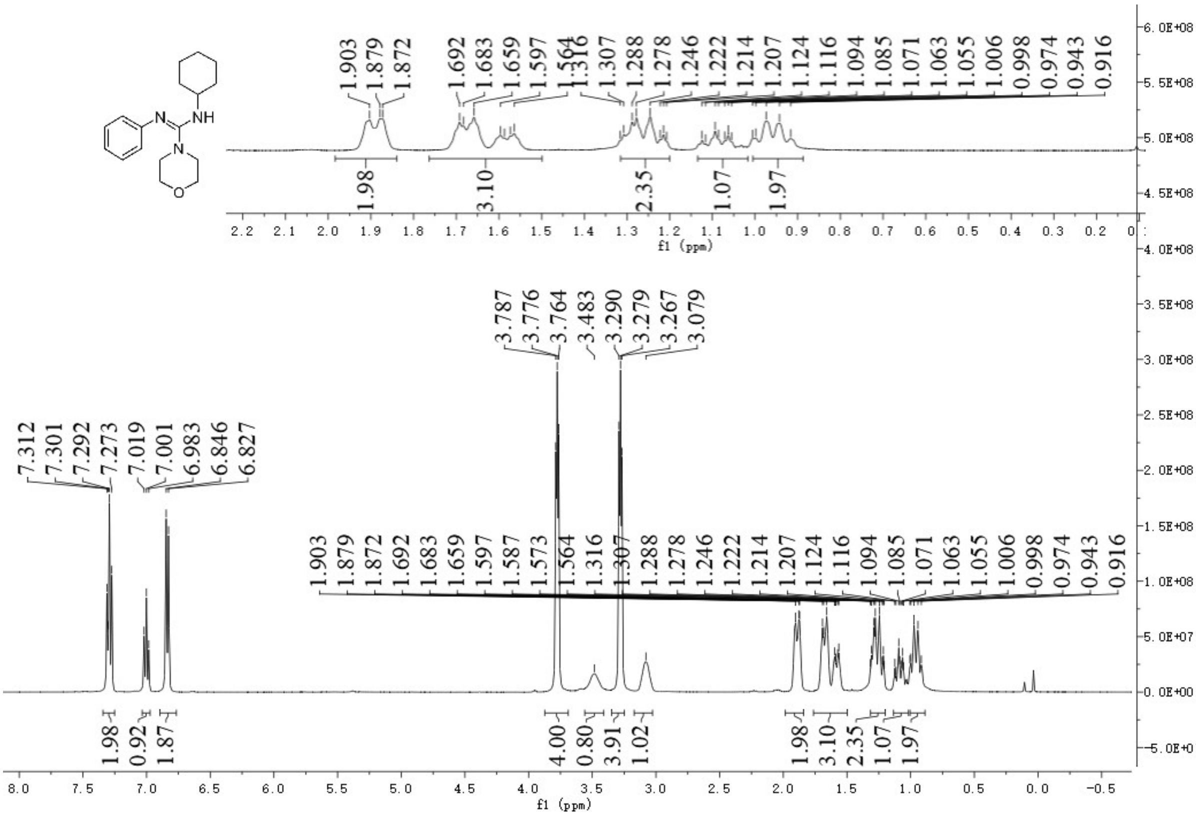

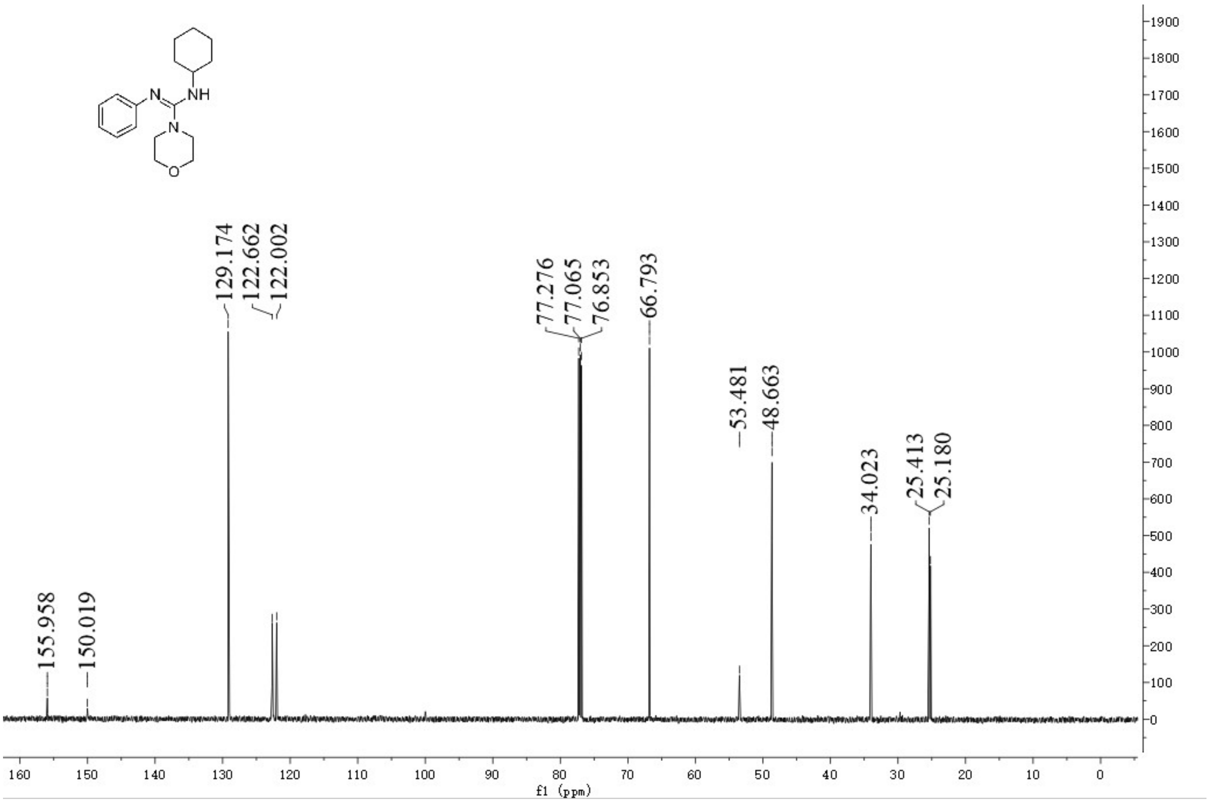

***N*-(3,3-Dimethylbutan-2-yl)-*N'*-(pyridin-4-yl)morpholine-4-carboximidamide (13)**

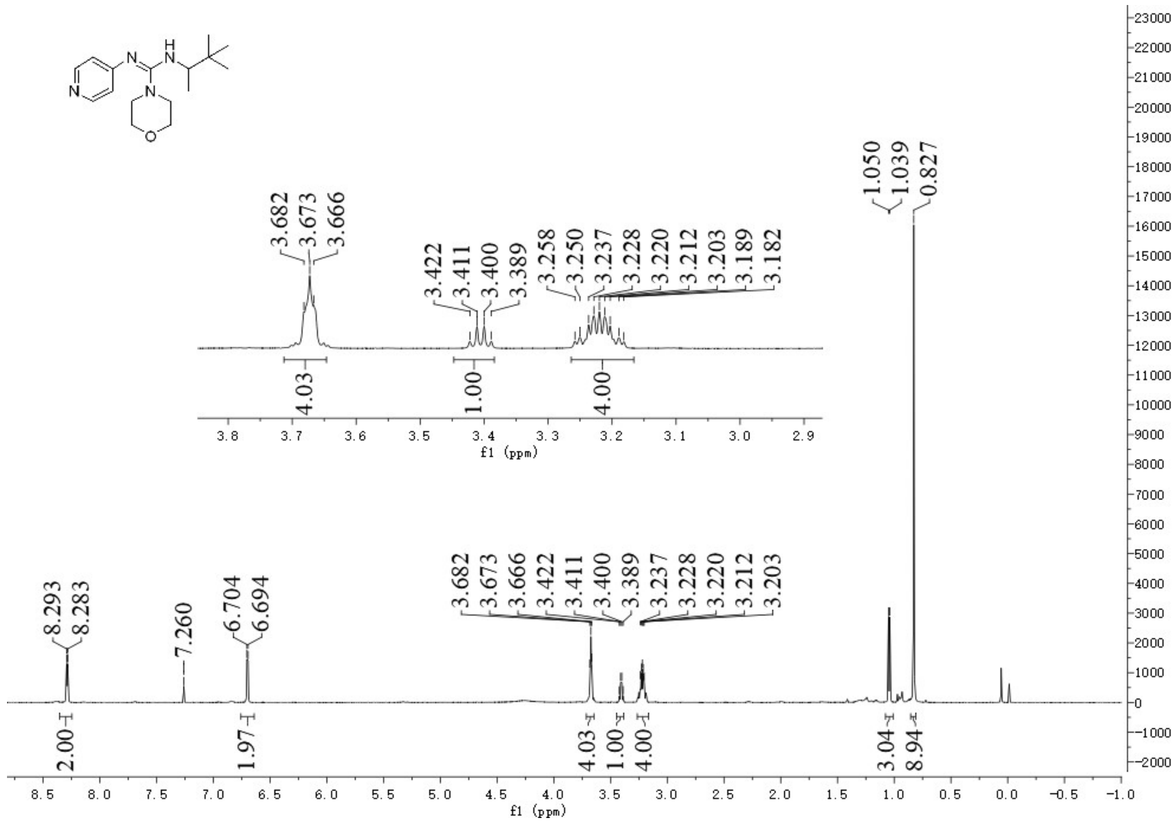

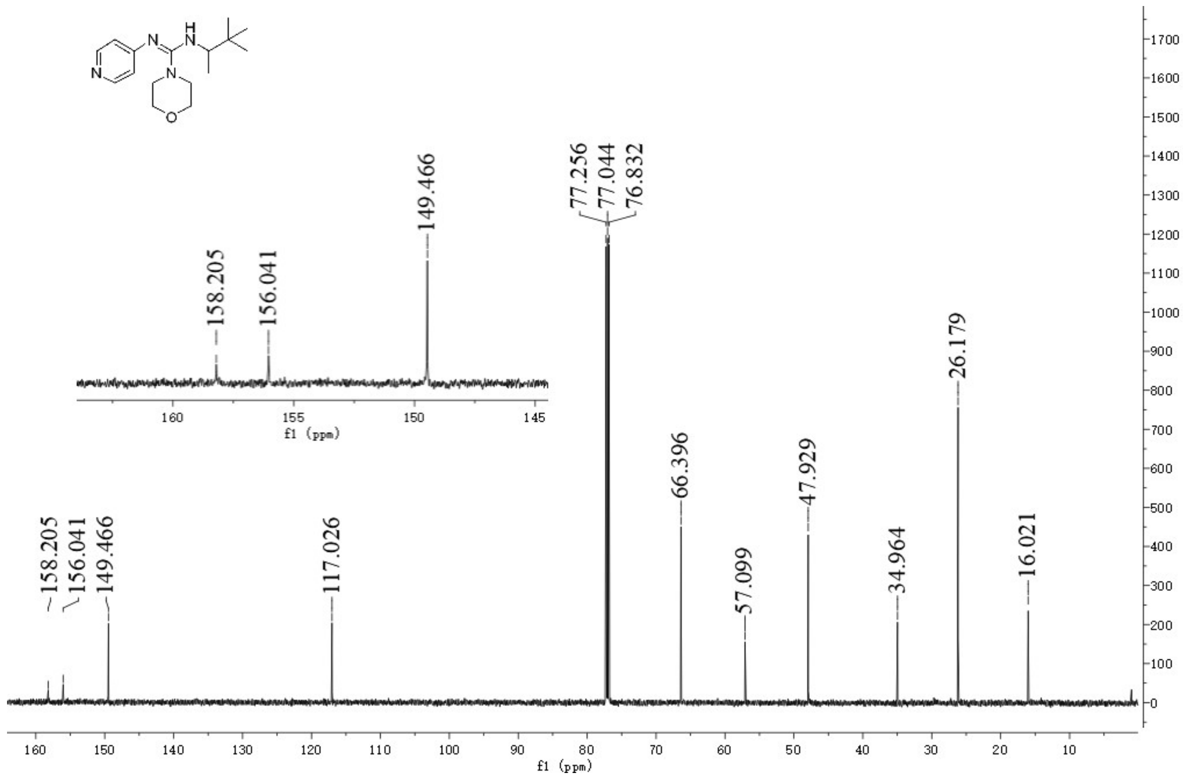

N-Benzhydryl-*N′*-(4-cyanophenyl)morpholine-4-carboximidamide (14)

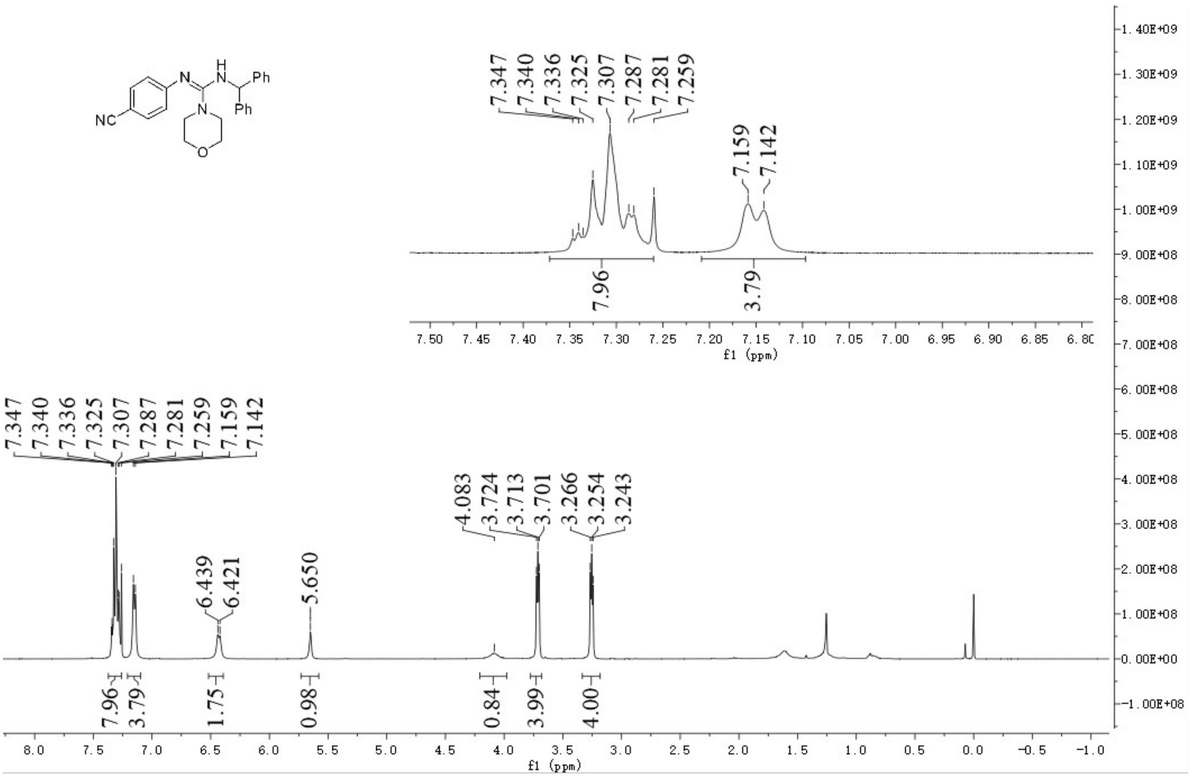

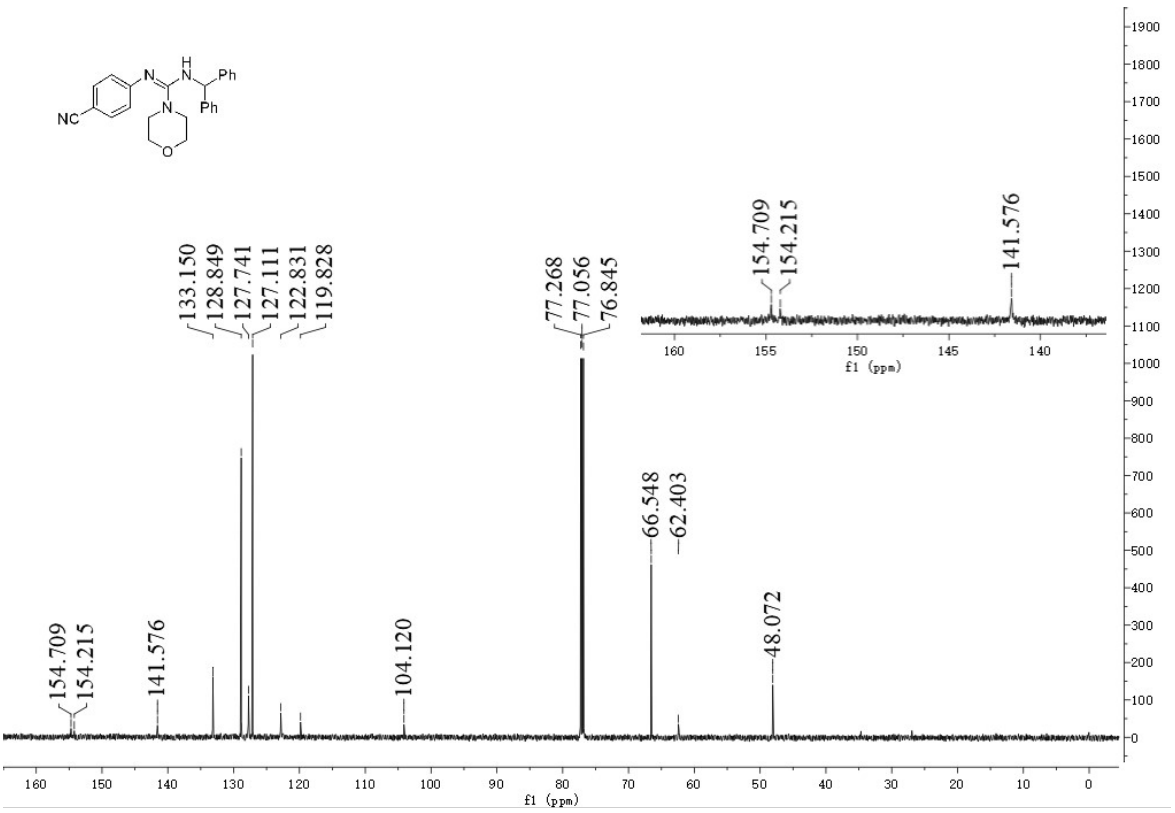

N-Benzyl-N′-phenylmorpholine-4-carboxamidamide (15)

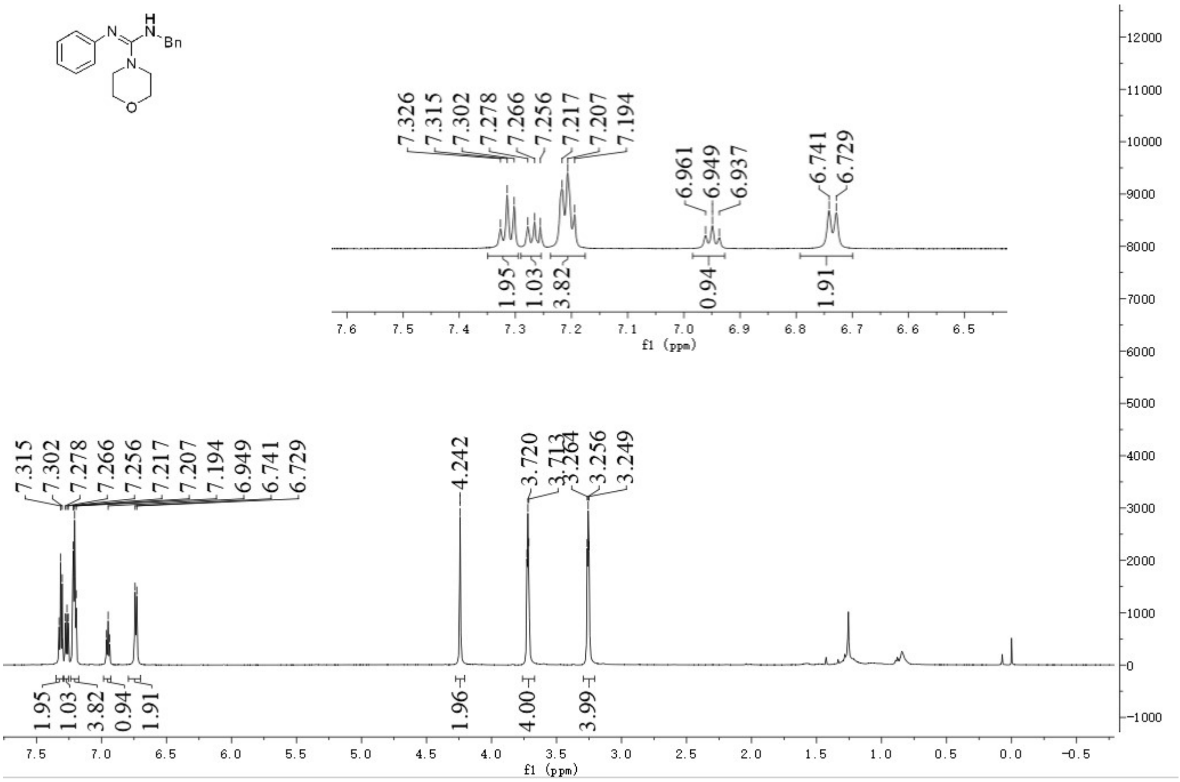

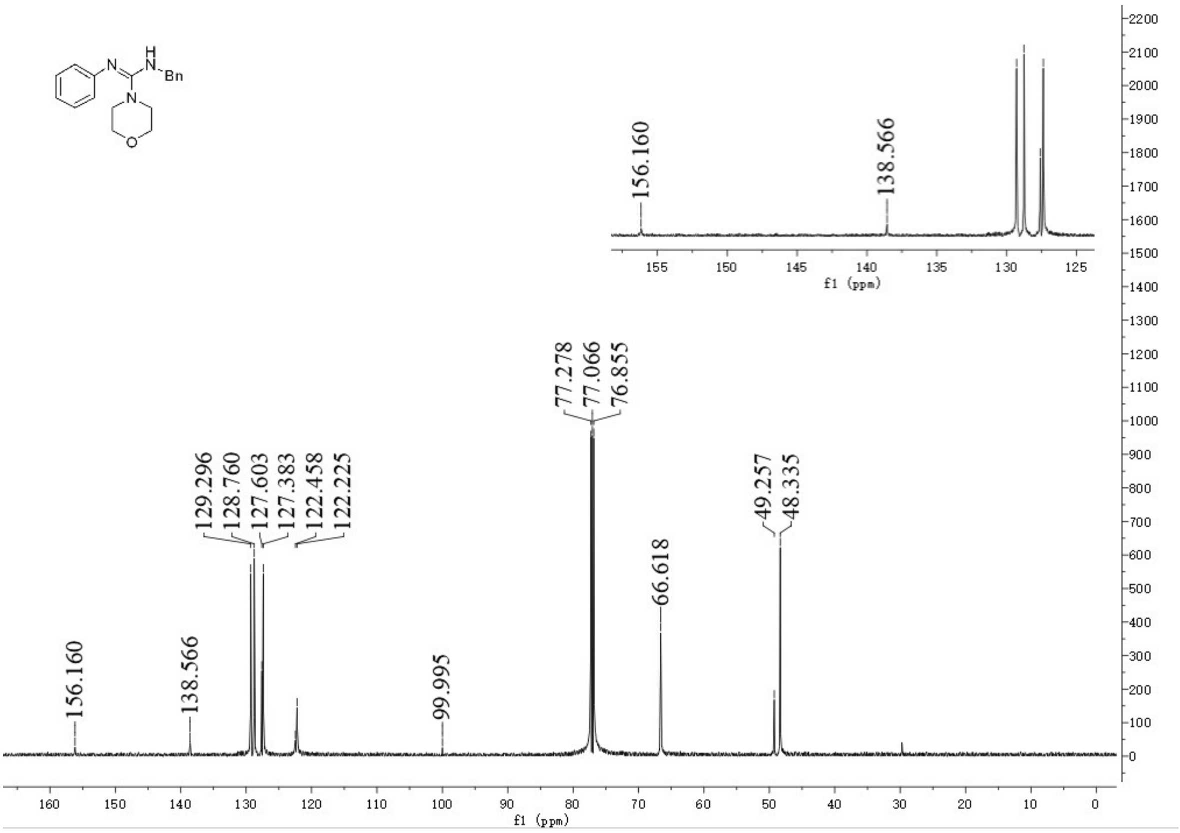

_N,N′_-Diphenylpyrrolidine-1-carboximidamide (17)

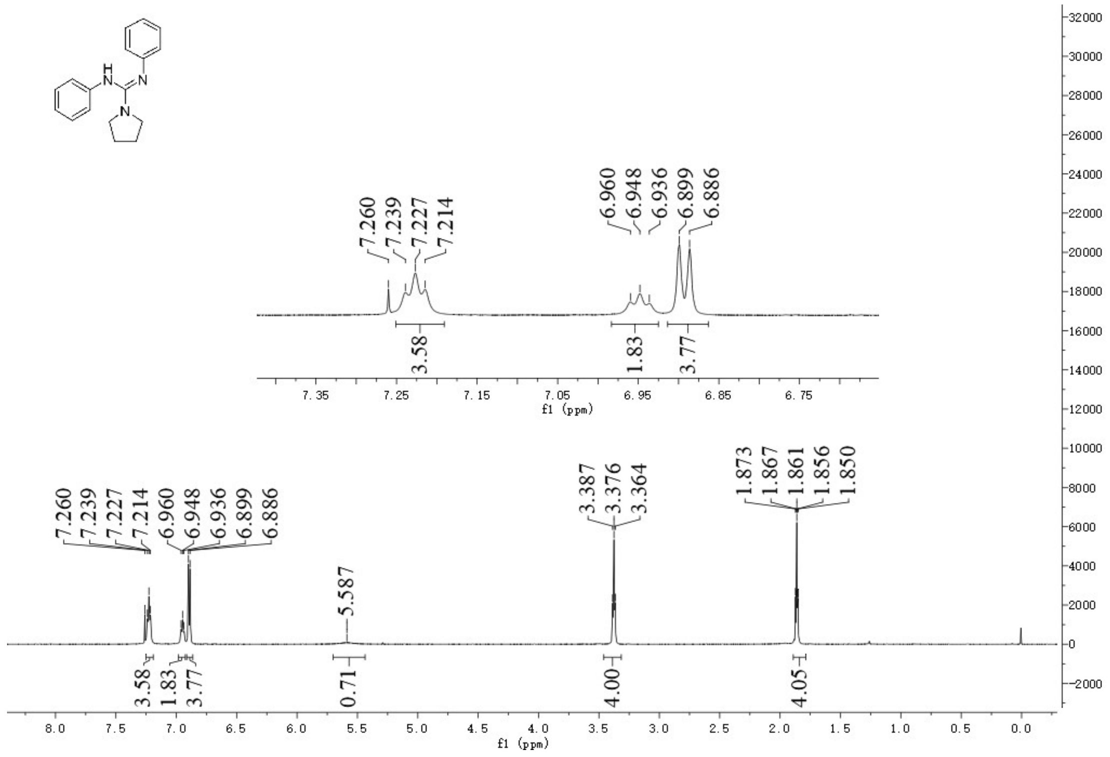

_N,N′_-Diphenylpiperidine-1-carboximidamide (18)

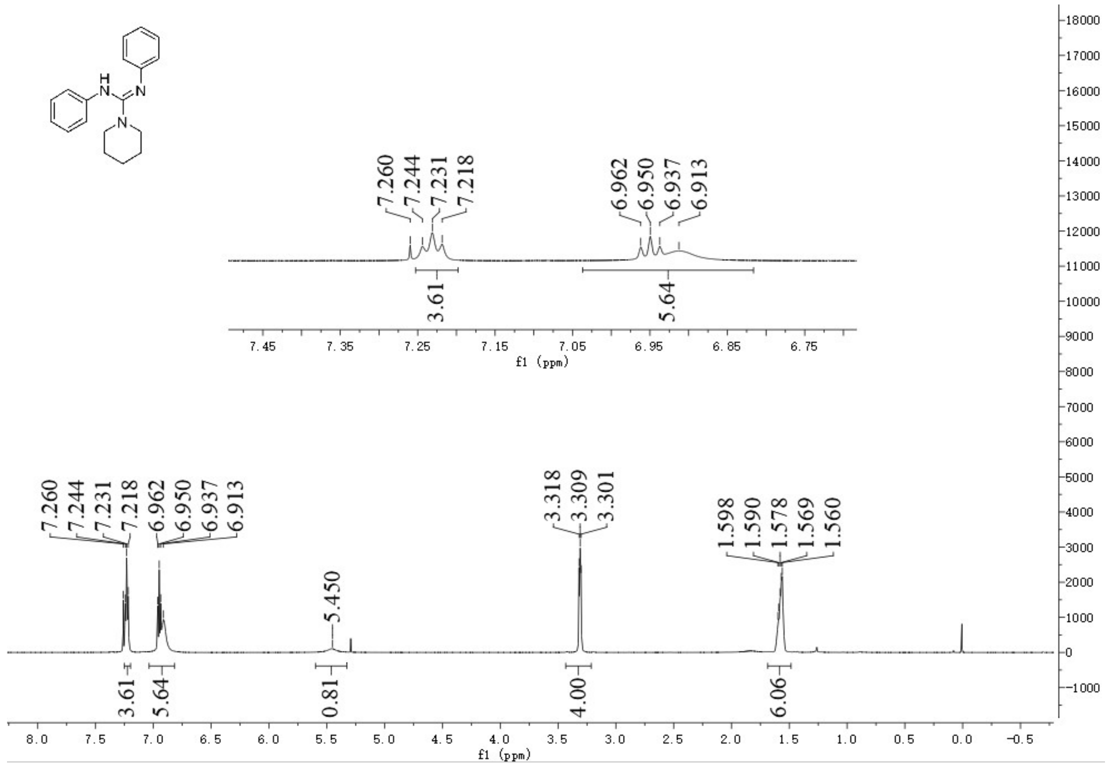

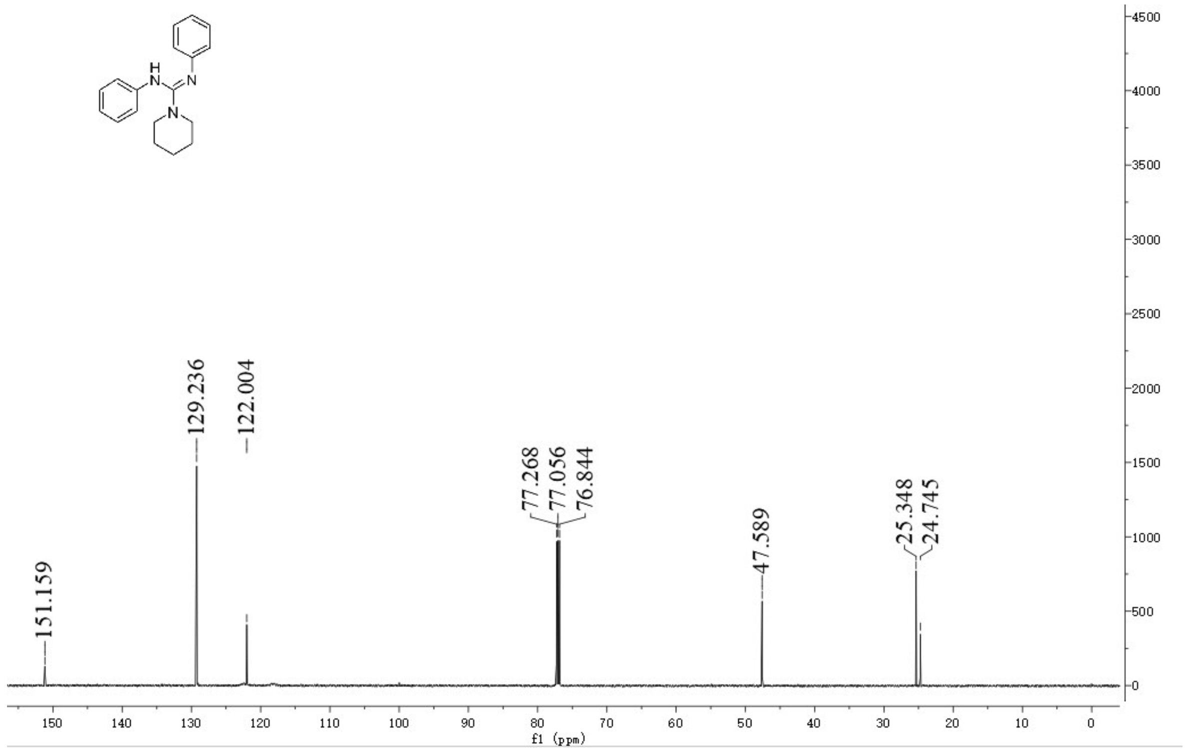

1,1-Dimethyl-2,3-diphenylguanidine (19)

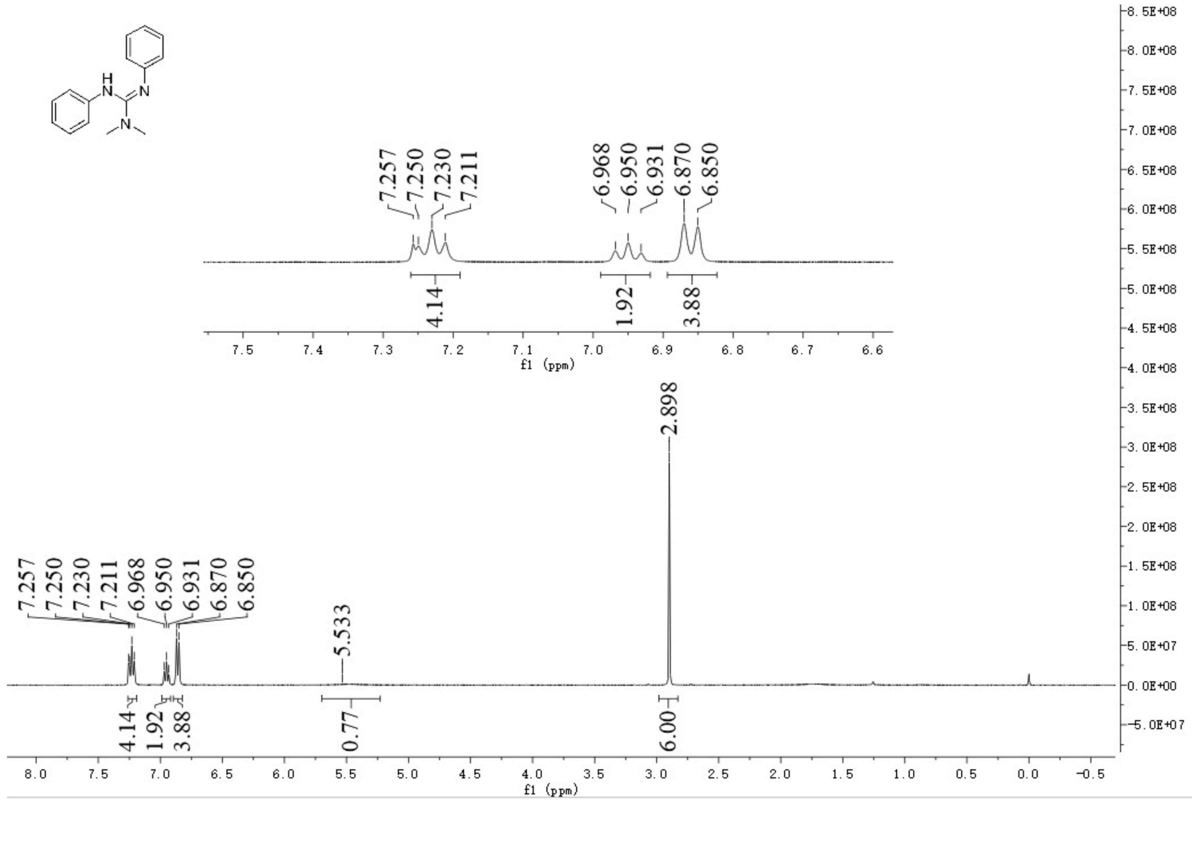

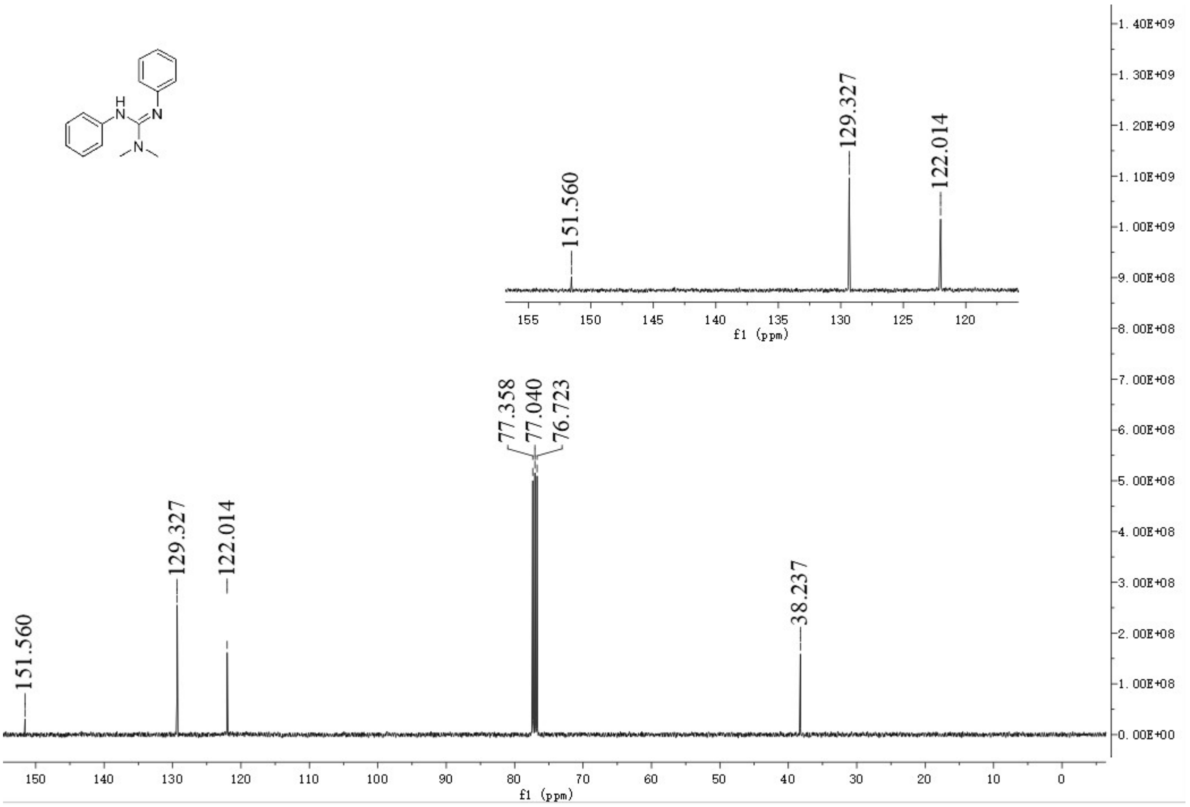

1-Butyl-2,3-diphenylguanidine (20)

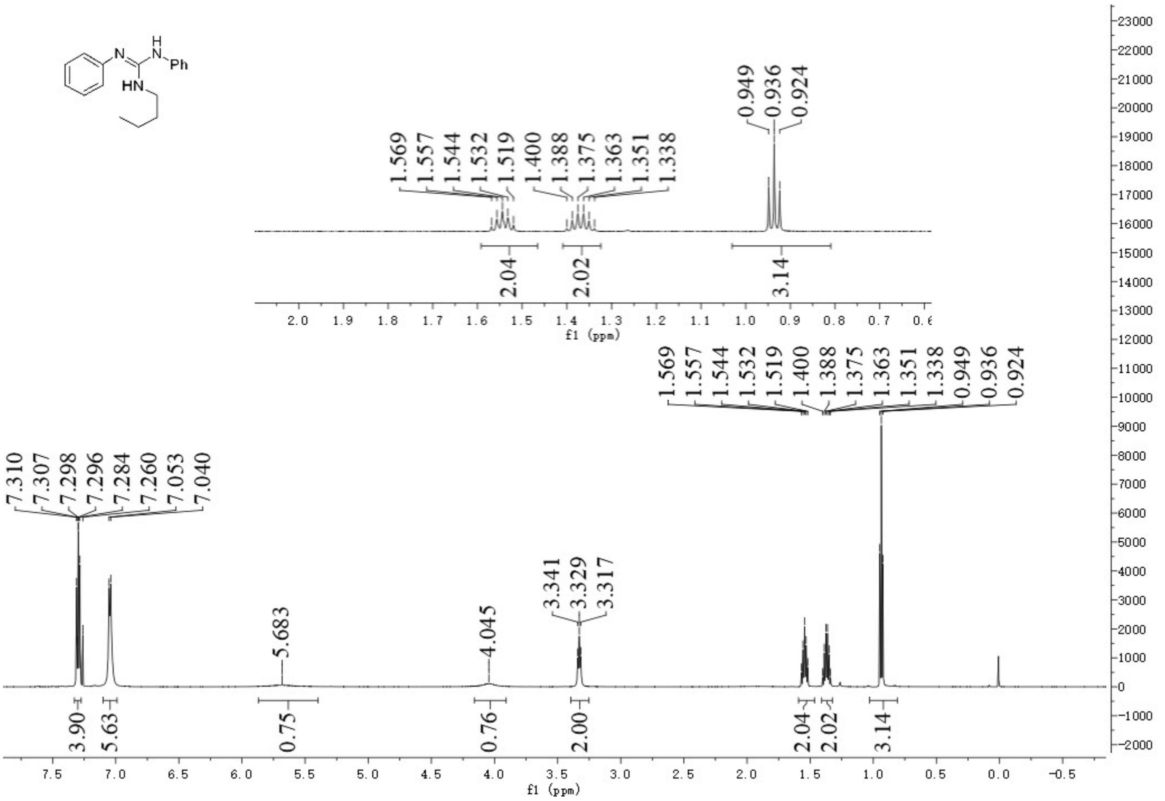

1-(2-Hydroxyethyl)-2,3-diphenylguanidine (21)

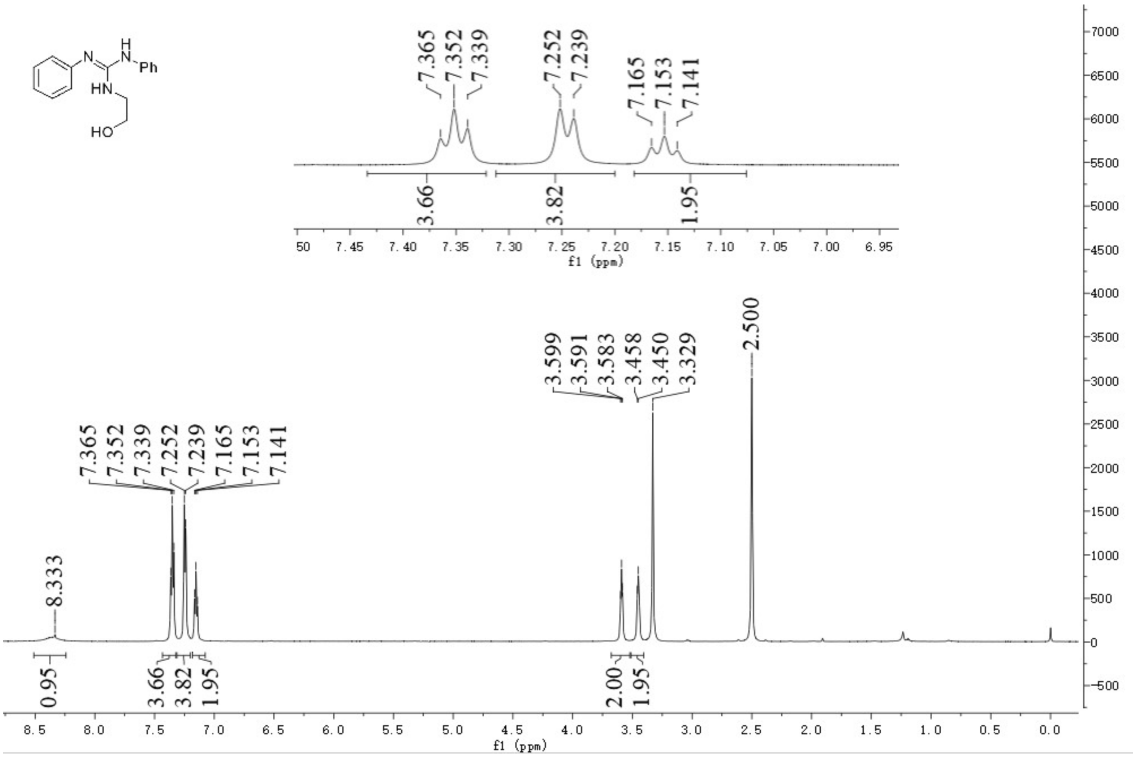

1-Benzyl-2,3-diphenylguanidine (22)

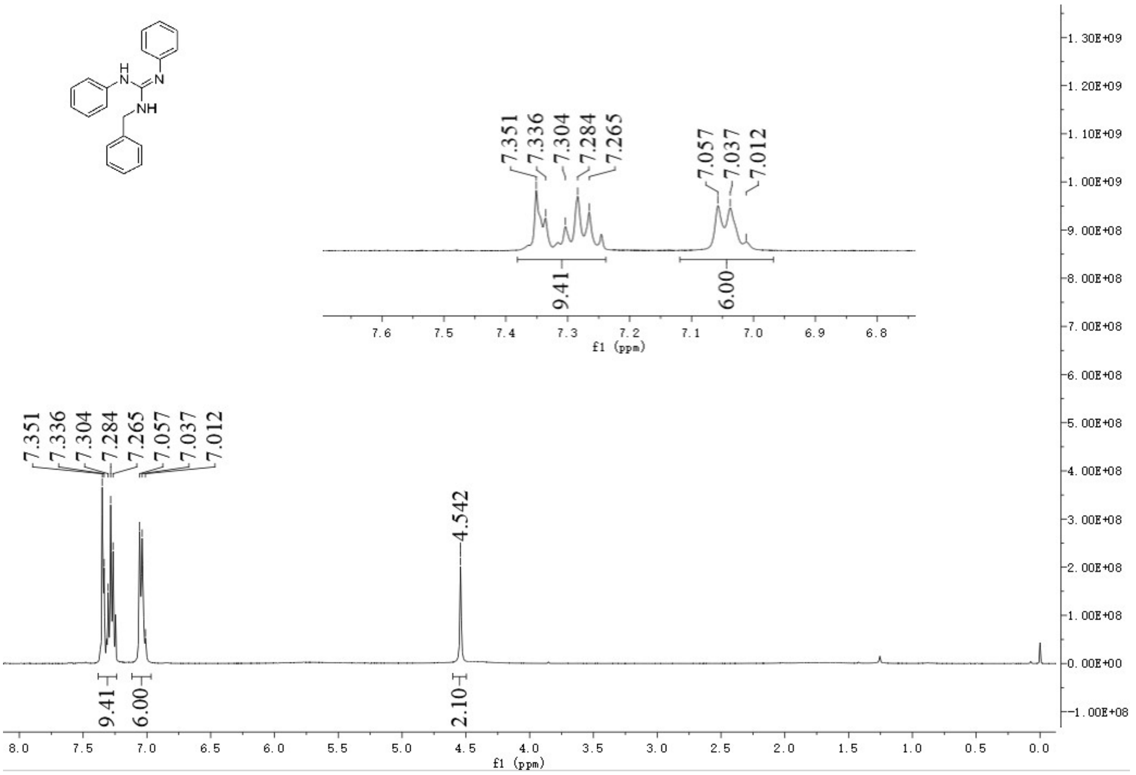

1,2-Diphenyl-3-(2-(thiophen-2-yl)ethyl)guanidine (23)

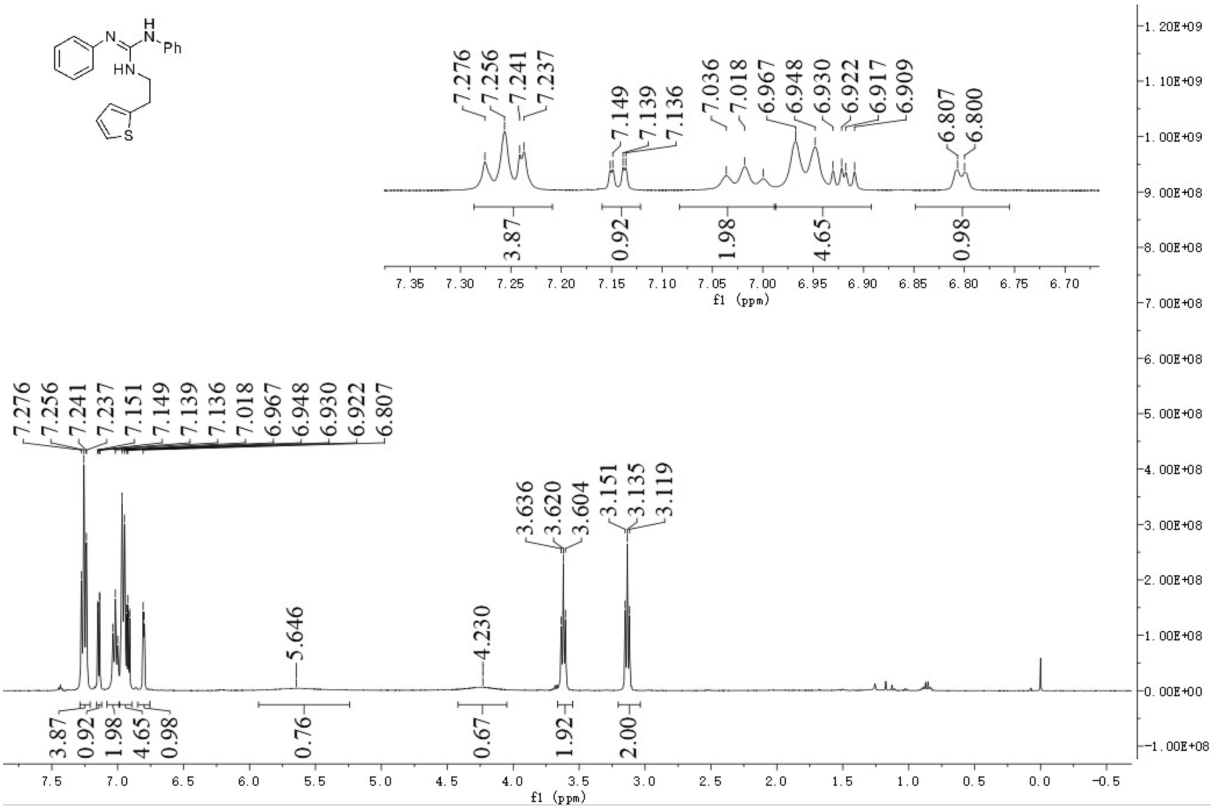

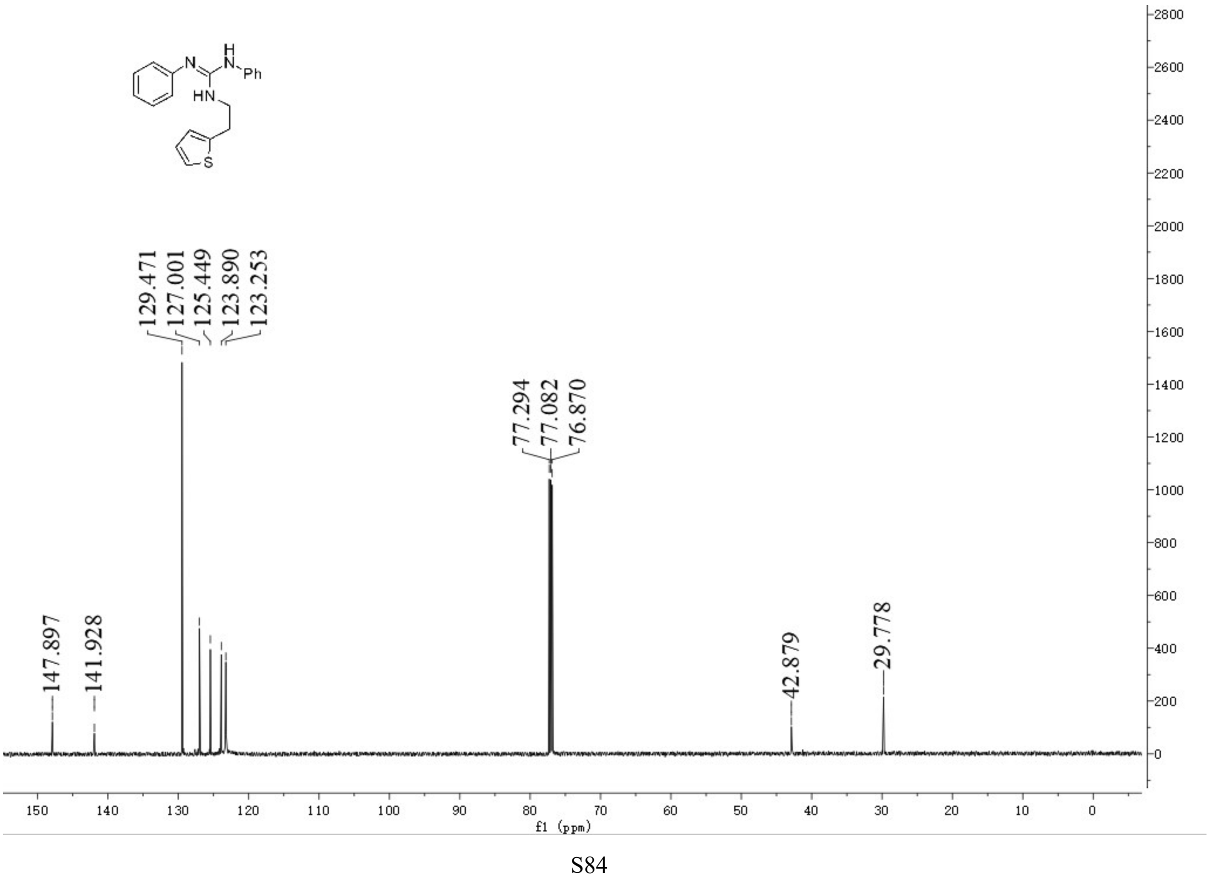

1-(2-(Cyclohex-2-en-1-yl)ethyl)-2,3-diphenylguanidine (24)

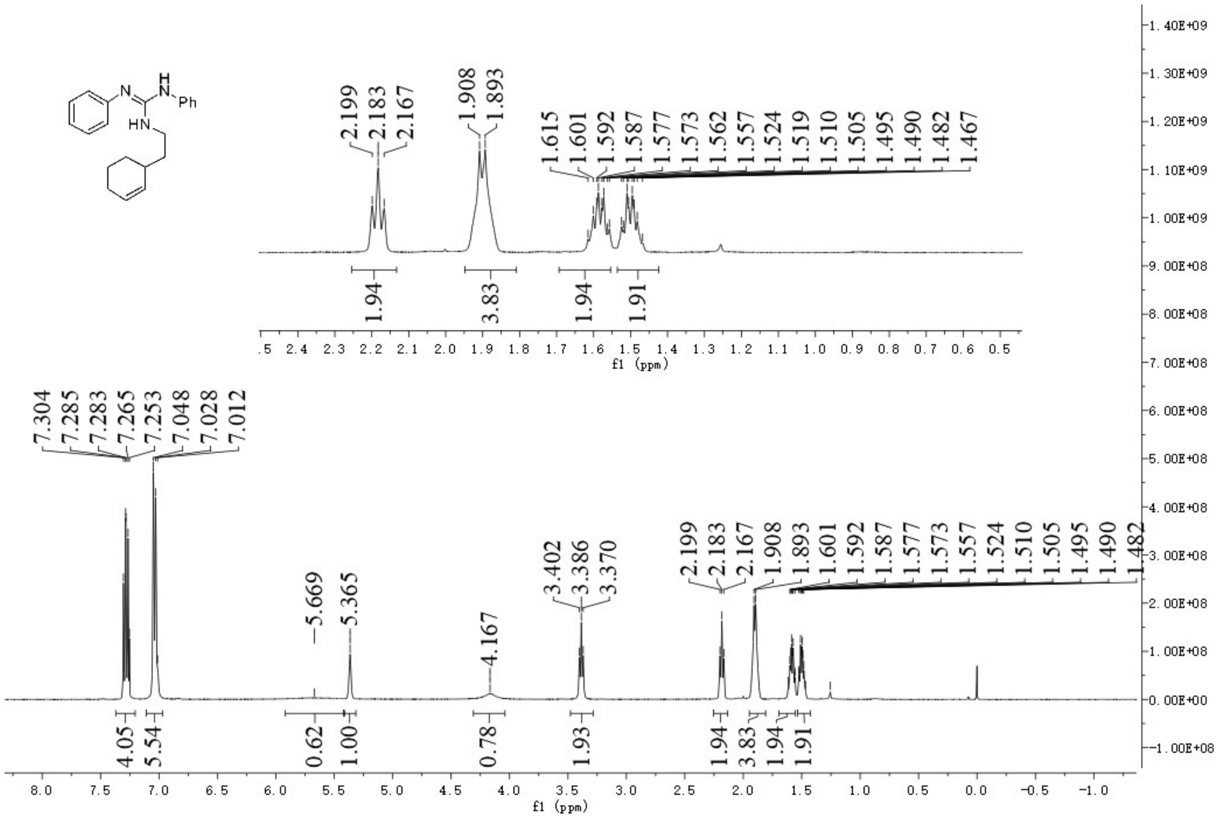

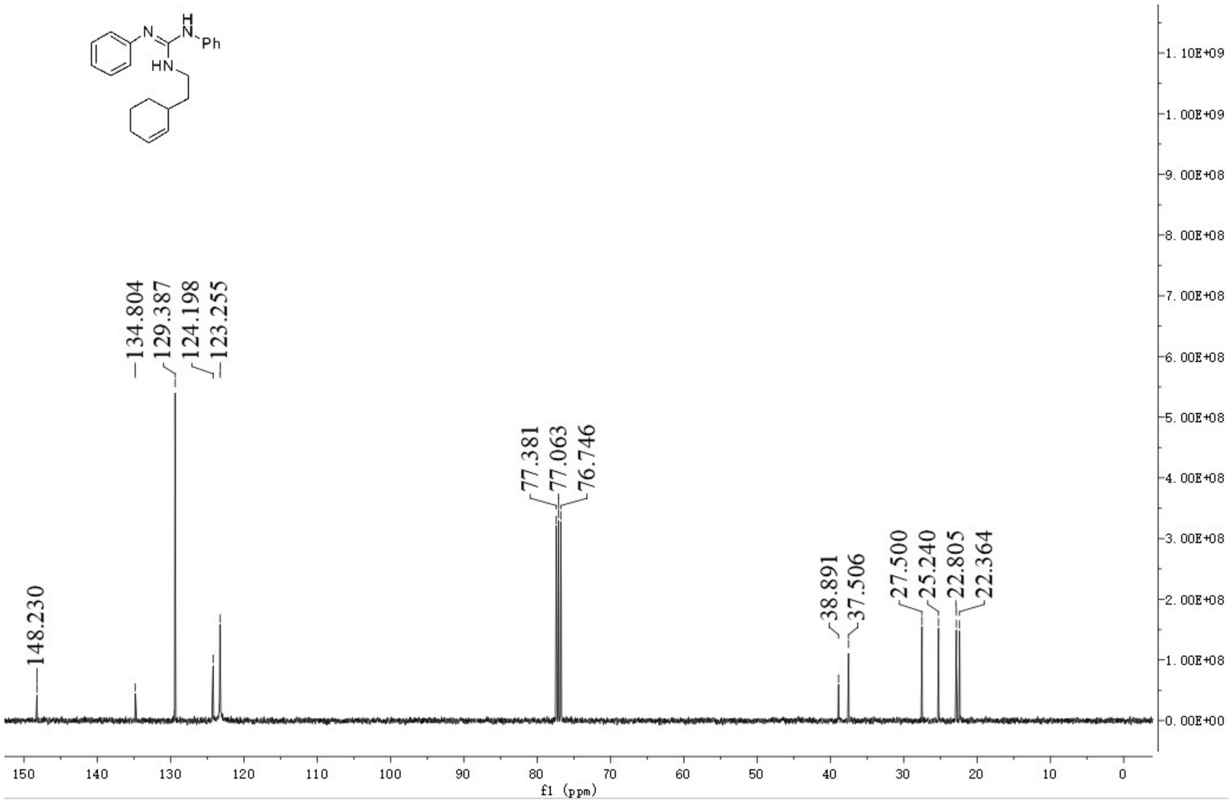

1-(But-3-yn-1-yl)-2,3-diphenylguanidine (25)

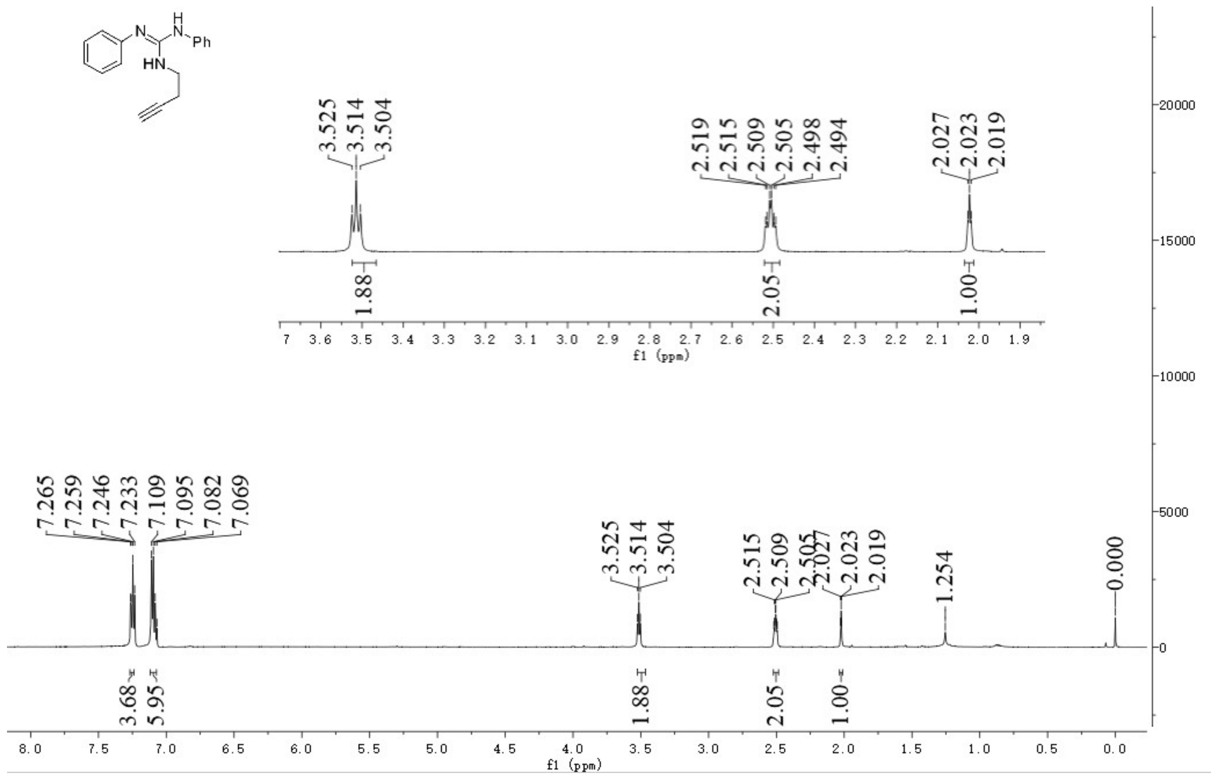

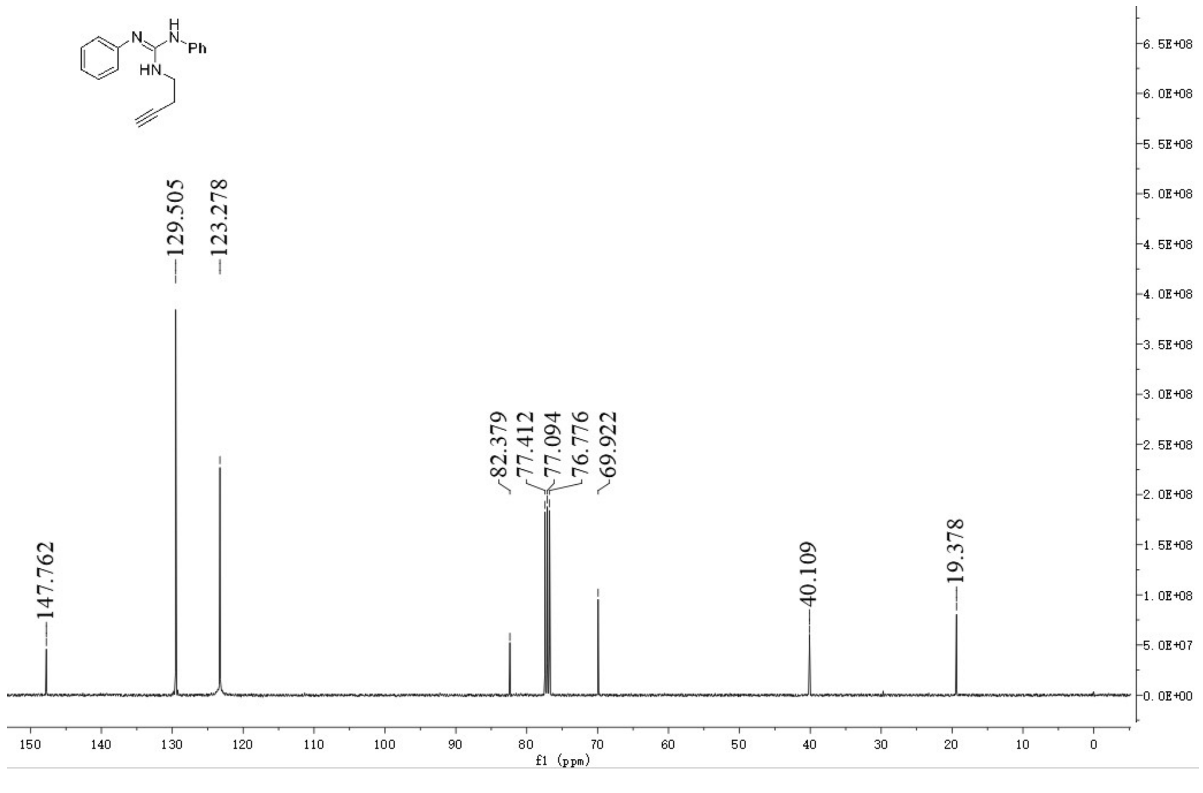

Ethyl-3-(2,3-diphenylguanidino)propanoate (26)

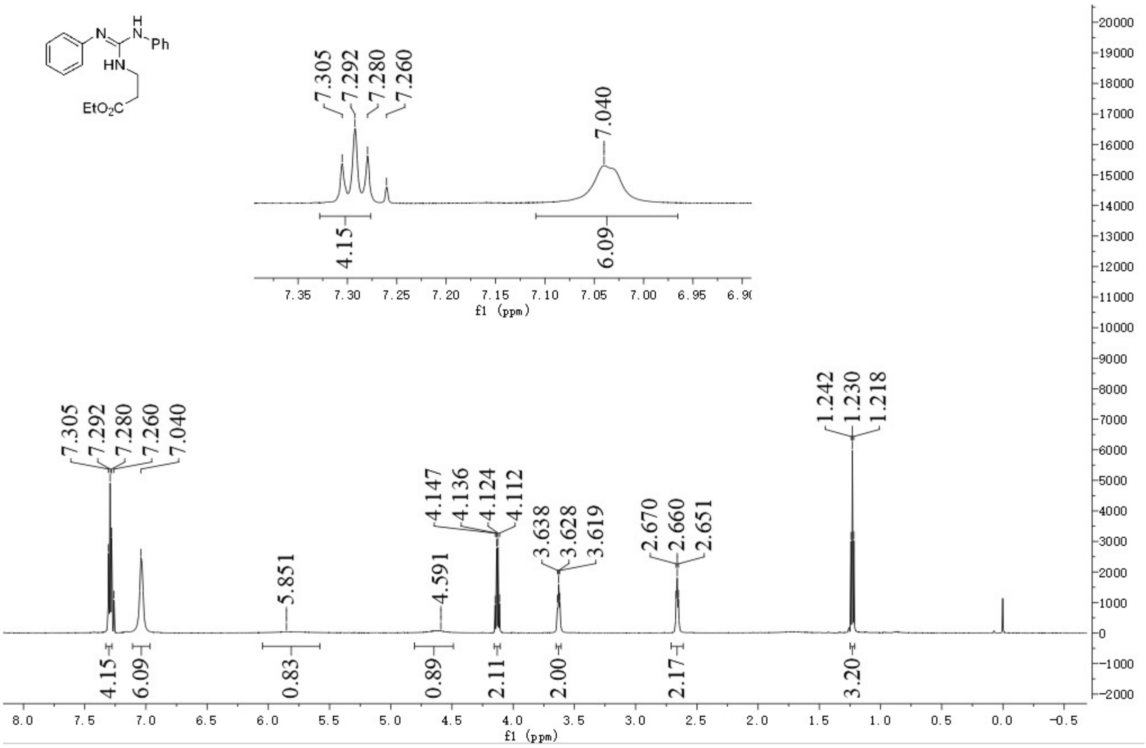

1-Cyclohexyl-2,3-diphenylguanidine (27)

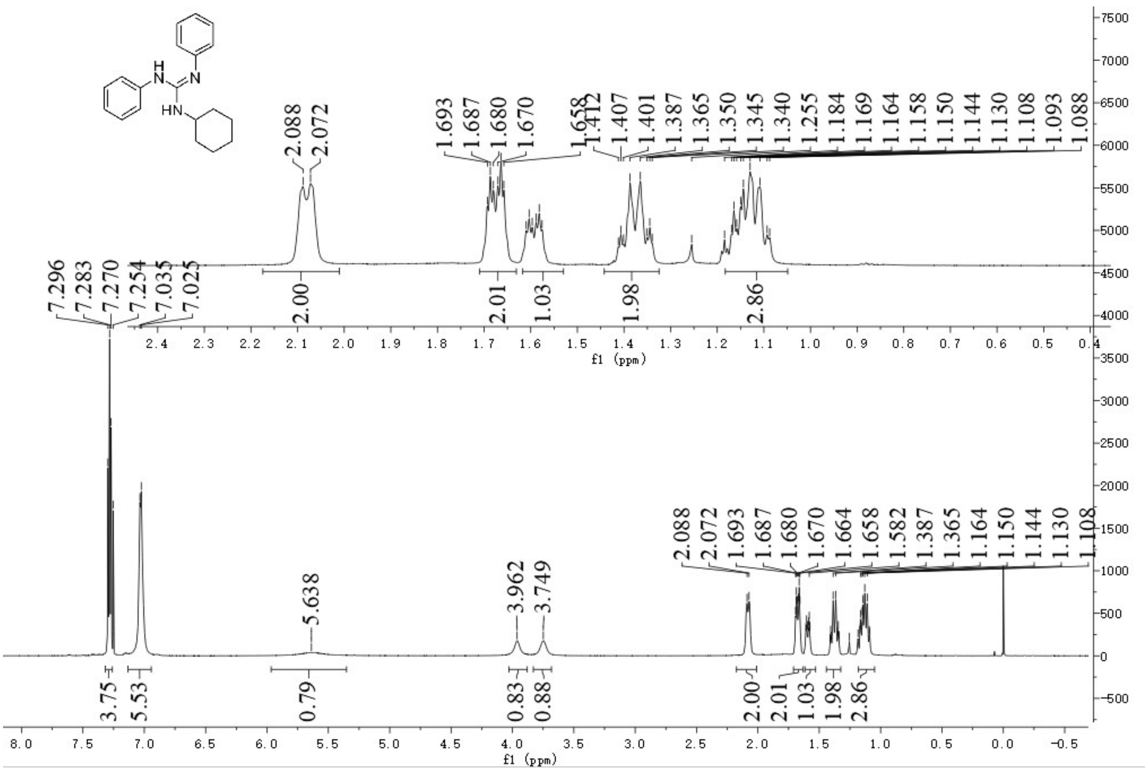

1-(Tert-butyl)-2,3-diphenylguanidine (28)

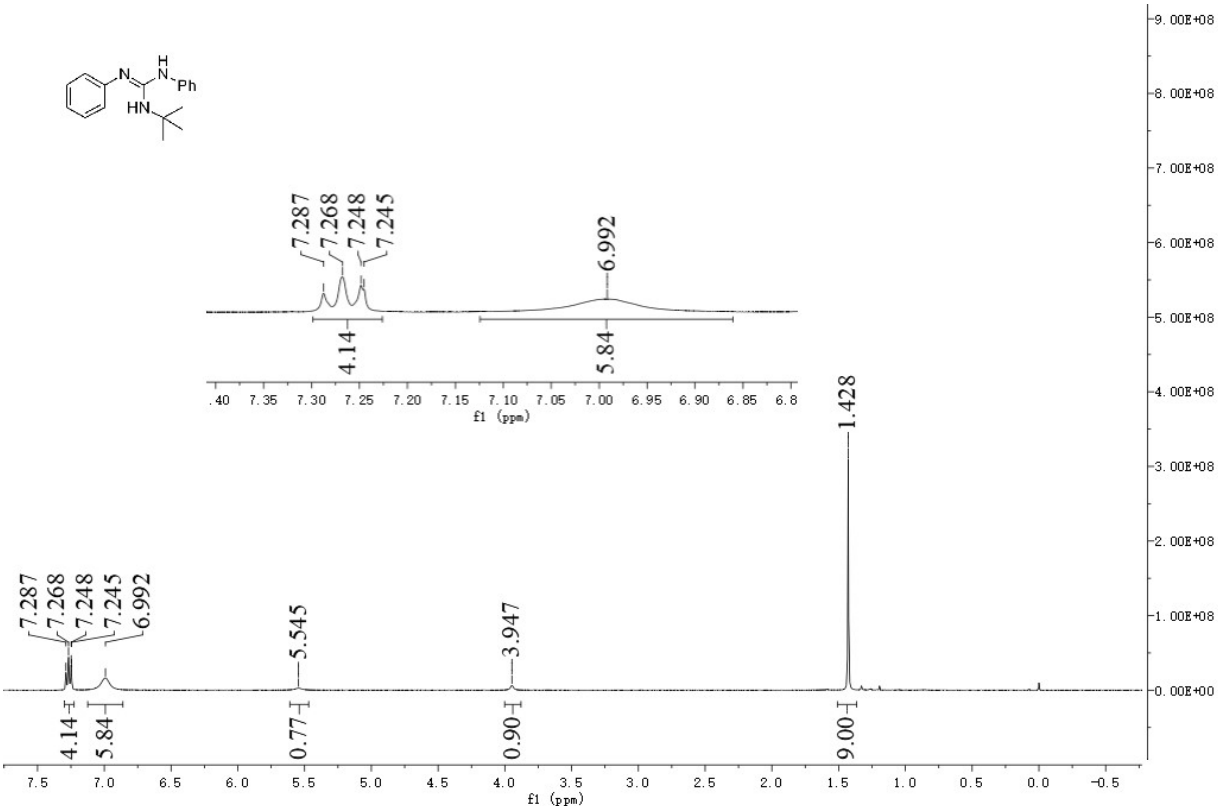

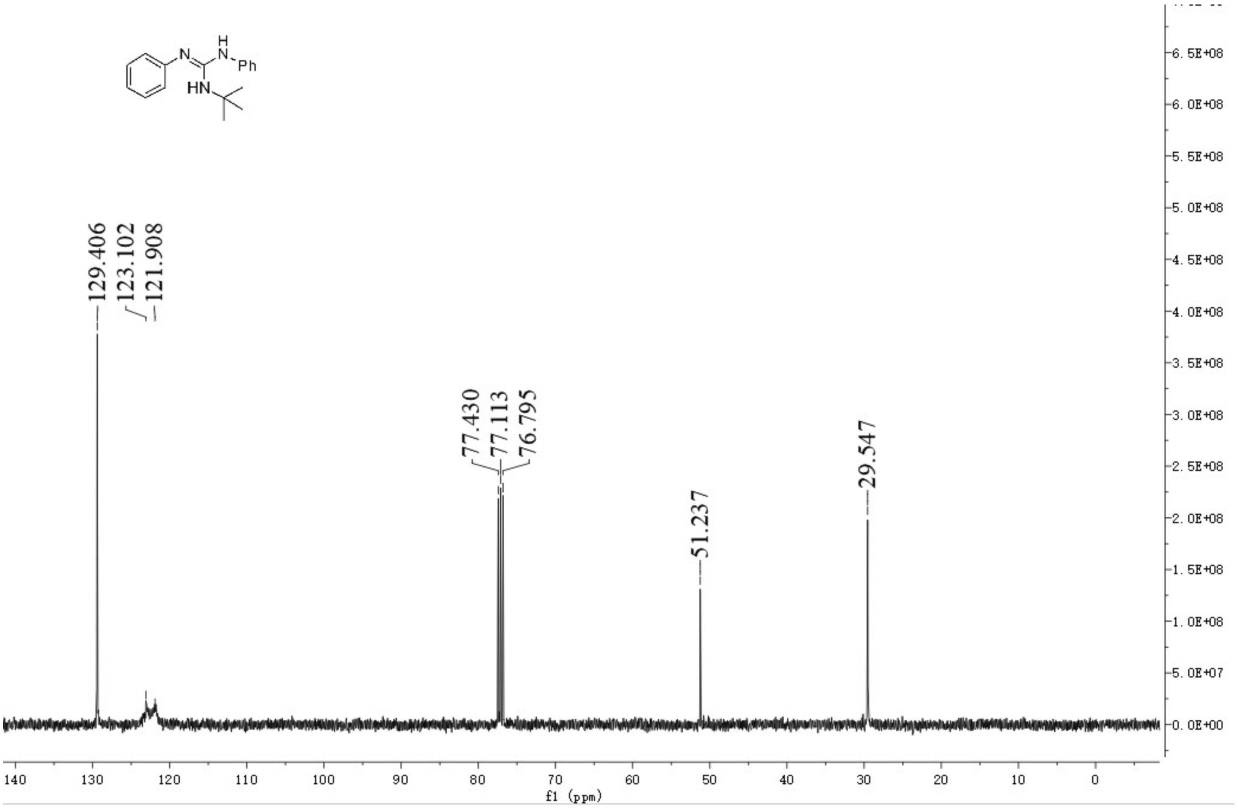

1-((3s,5s,7s)-Adamantan-1-yl)-2,3-diphenylguanidine (29)

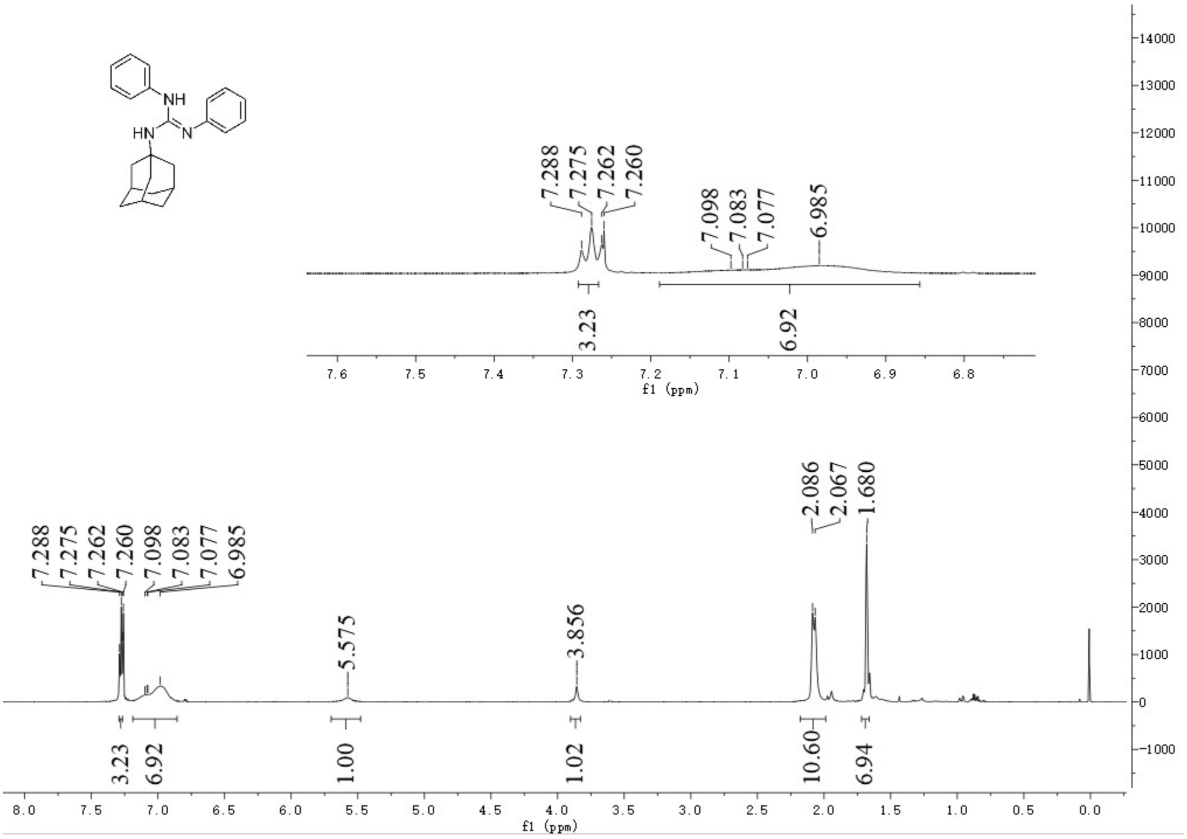

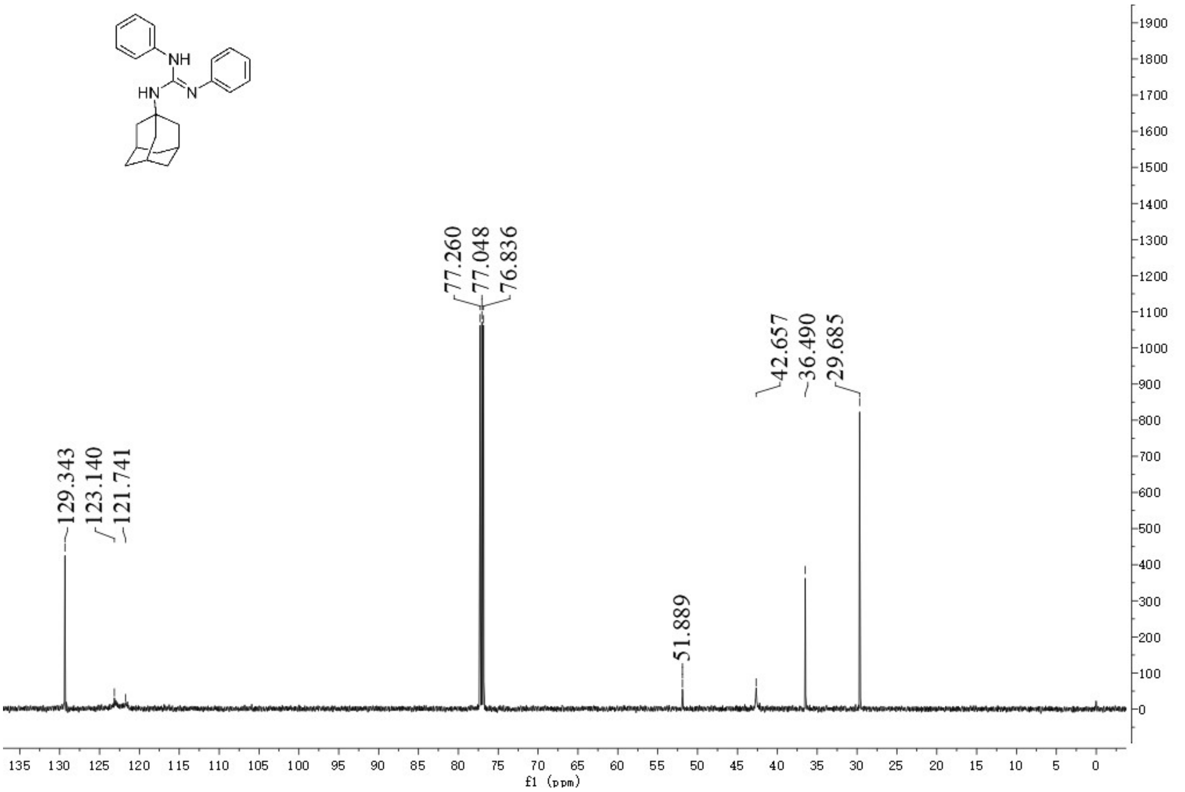

1,2,3-Triphenylguanidine (32)

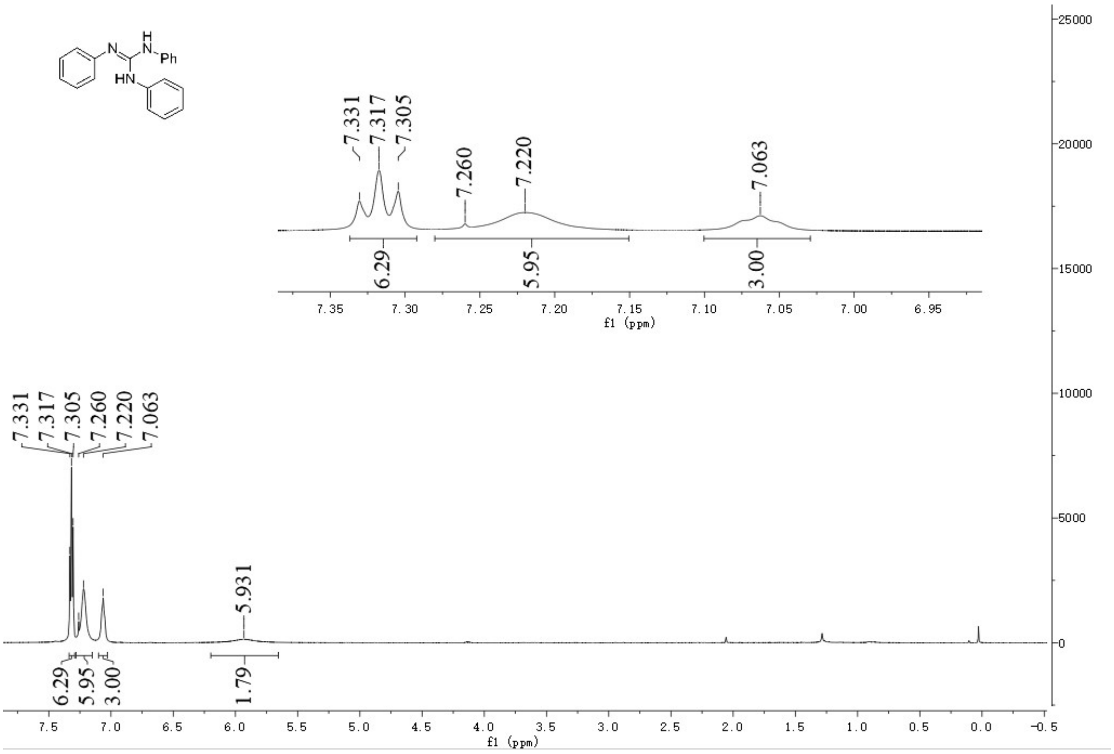

1,2-Diphenyl-3-(pyridin-2-yl)guanidine (33)

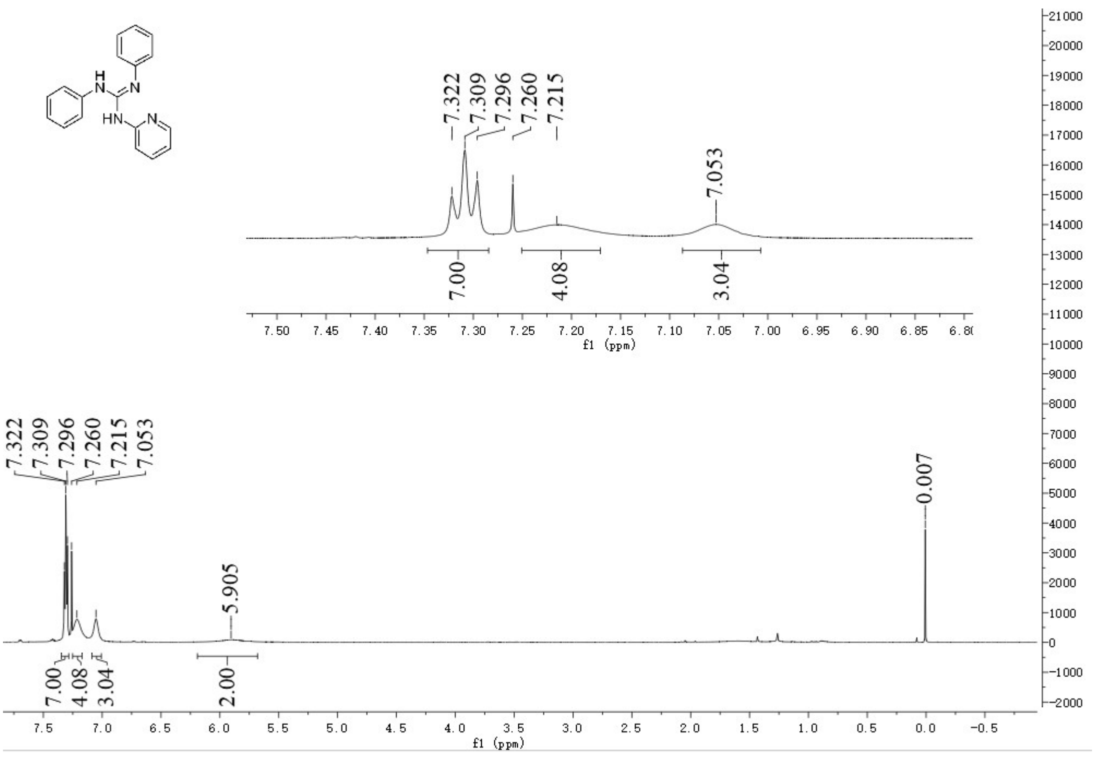

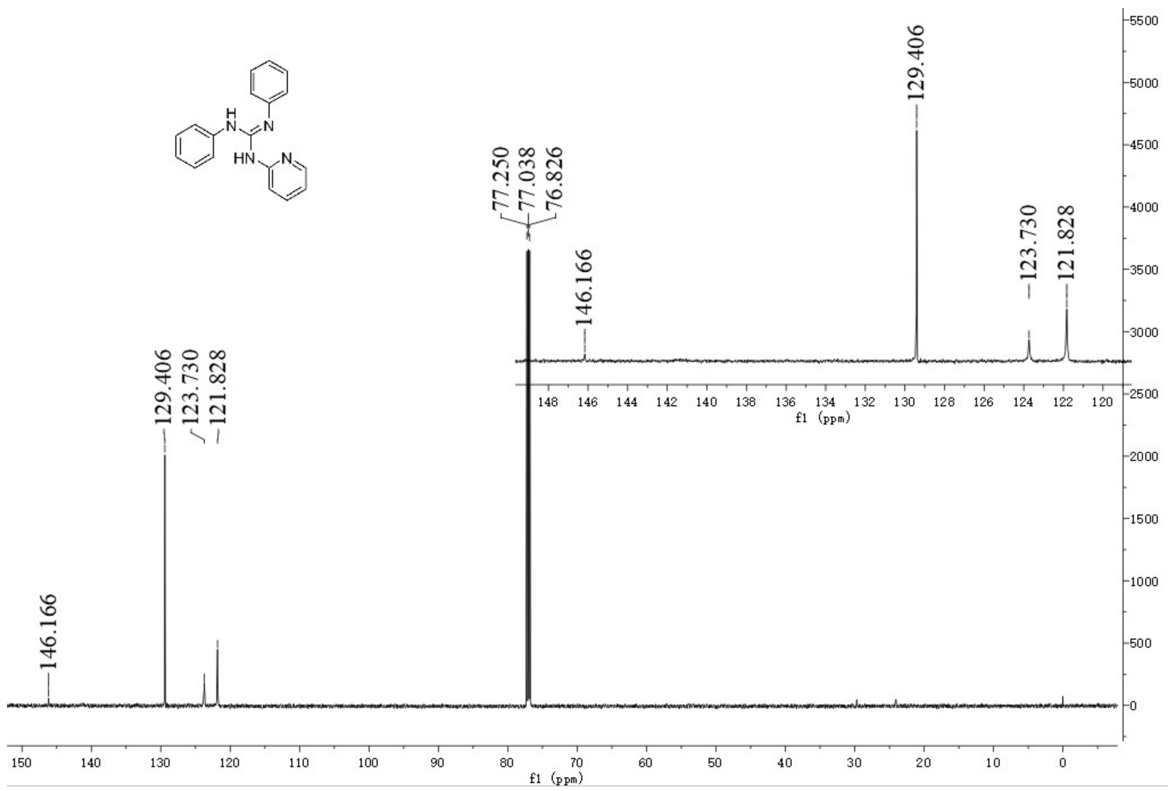

1,2-Diphenyl-3-(pyridin-4-yl)guanidine (34)

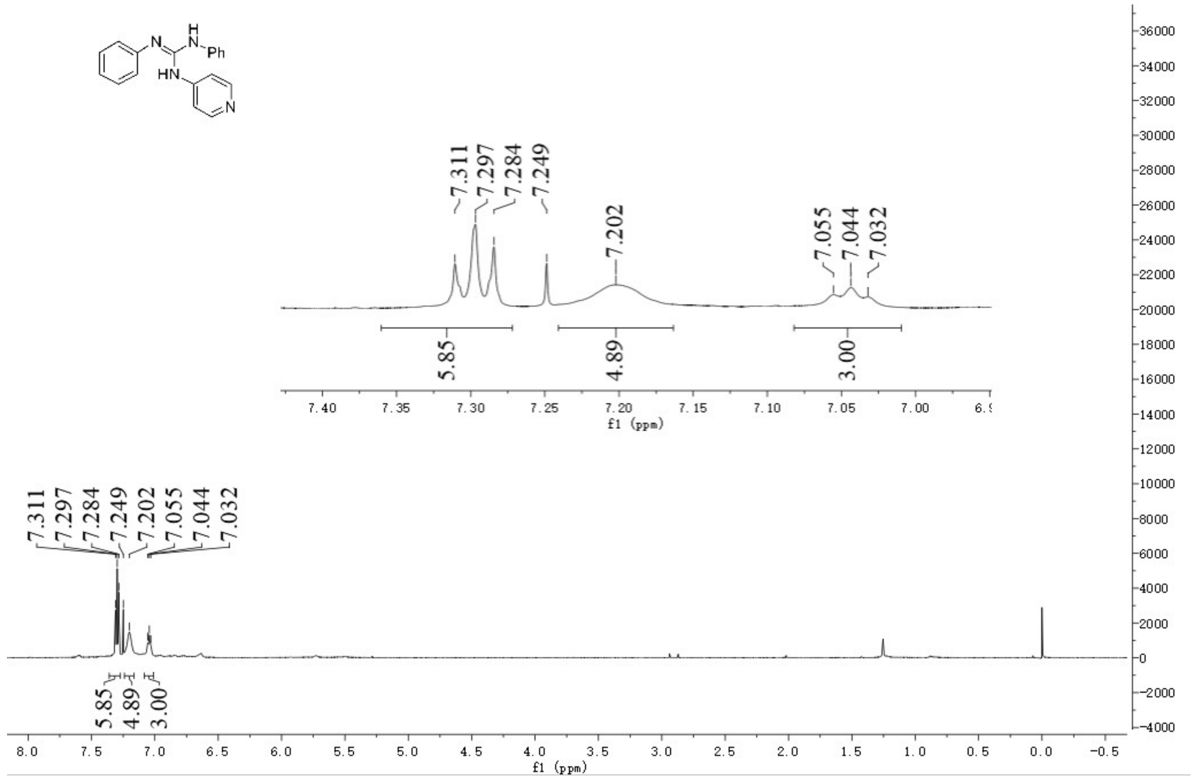

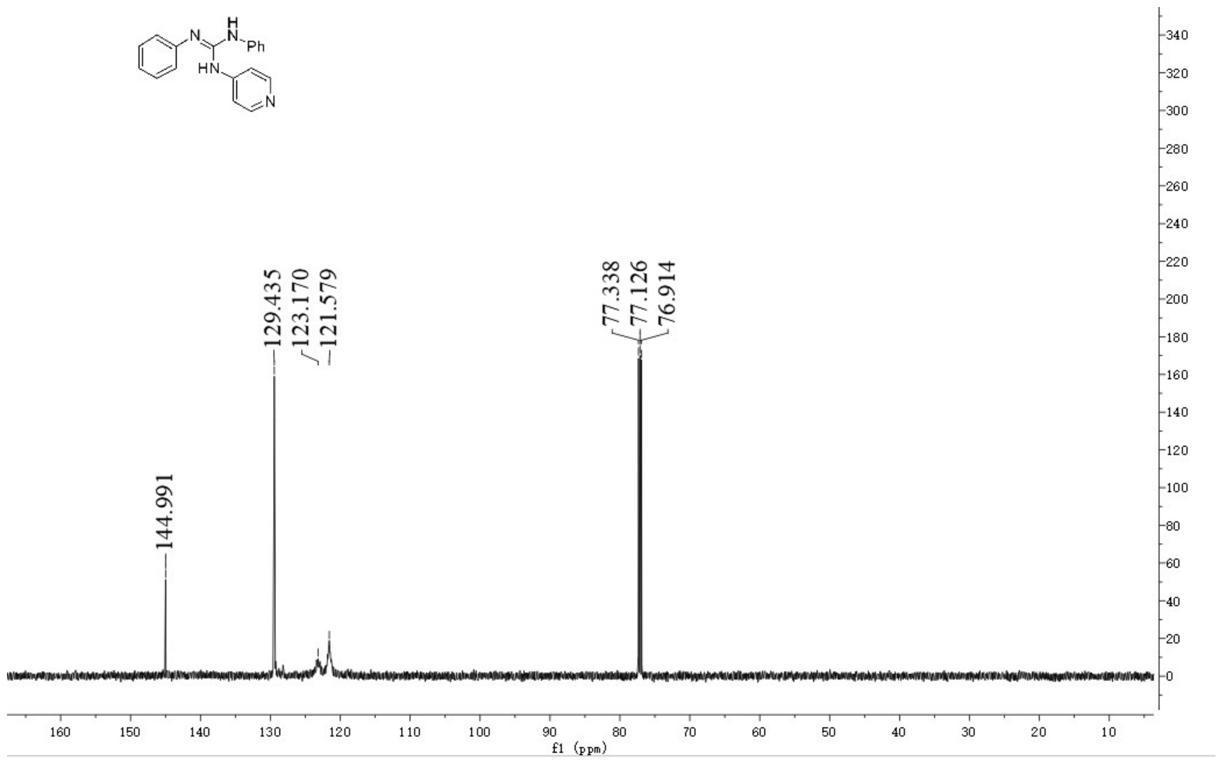

1-(Naphthalen-2-yl)-2,3-diphenylguanidine (35)

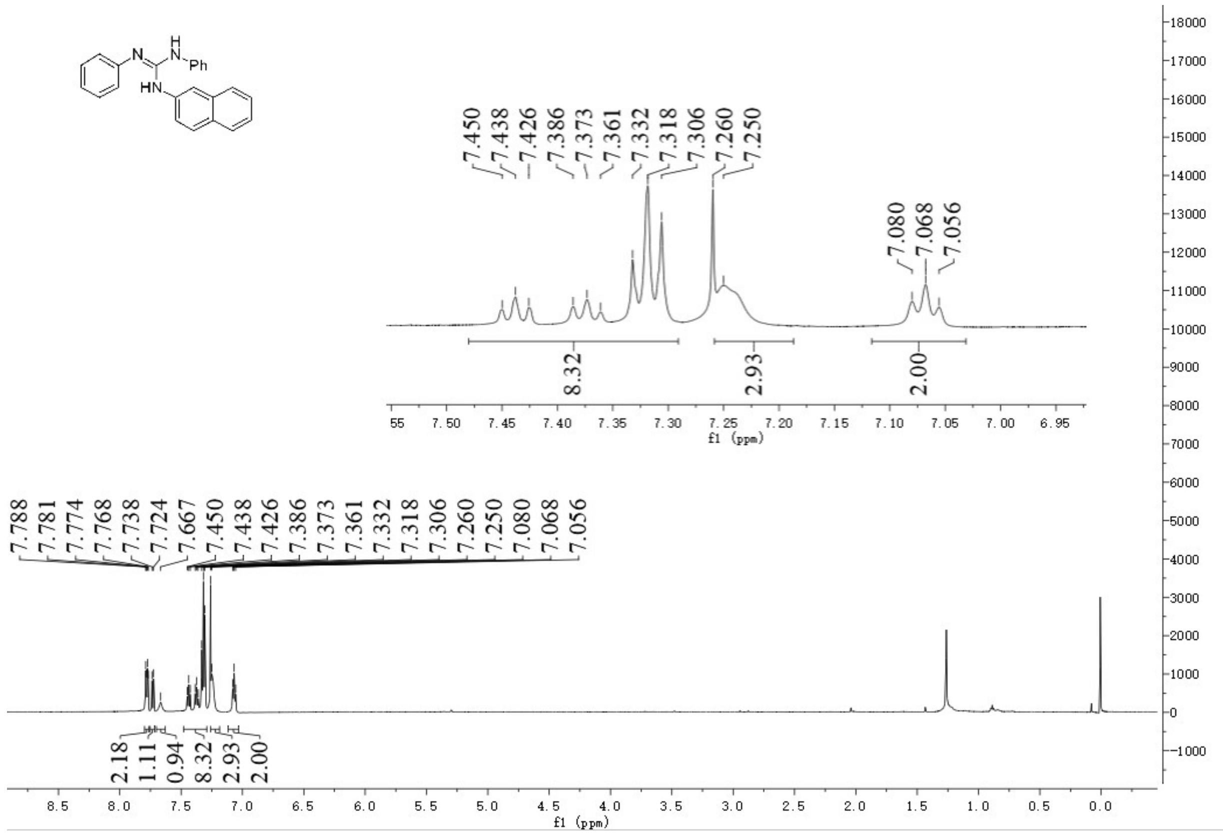

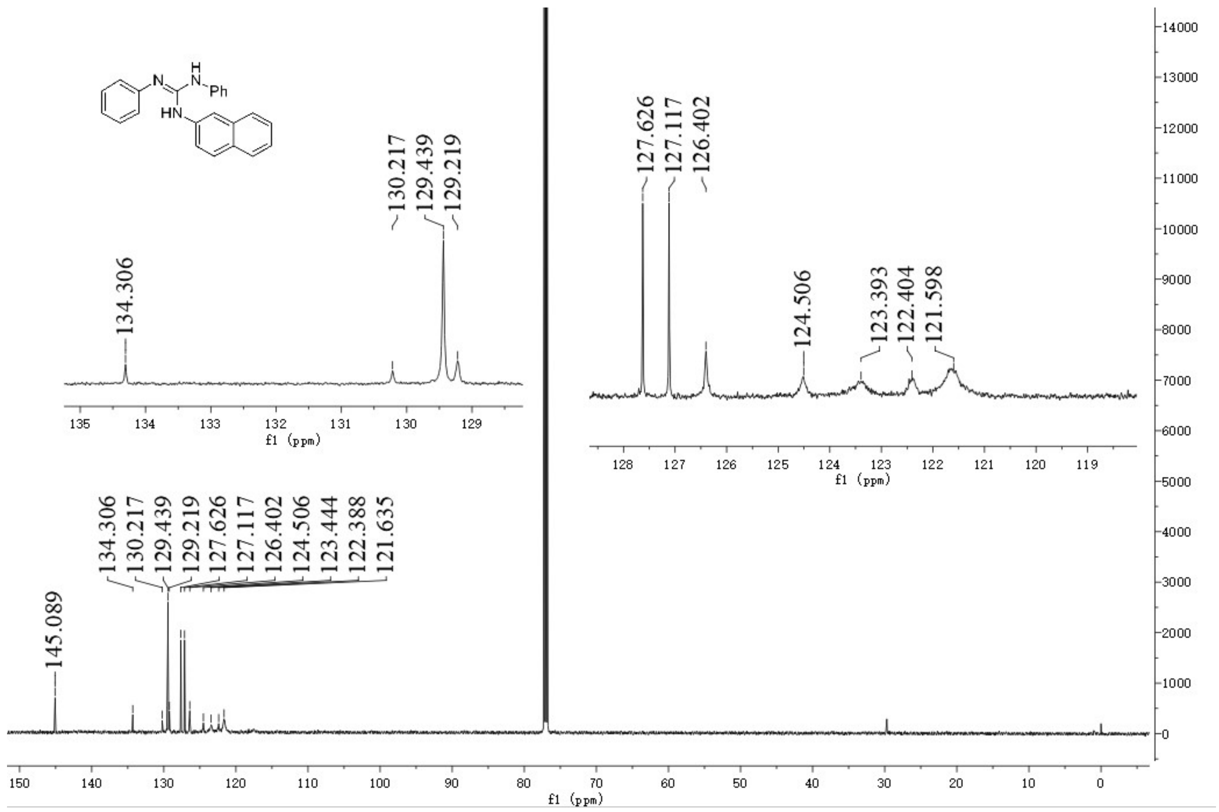

1-(4-Methoxyphenyl)-2,3-diphenylguanidine (36)

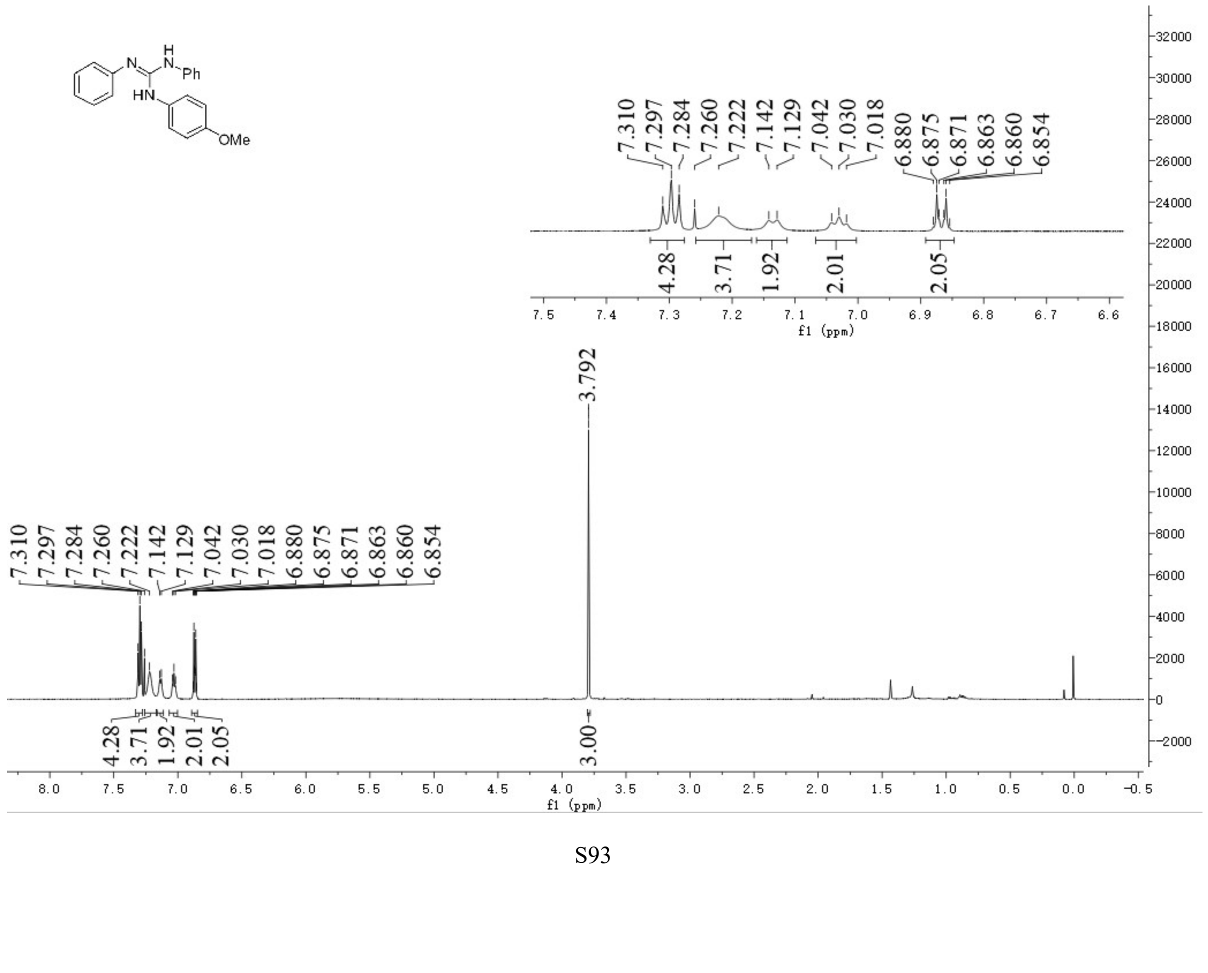

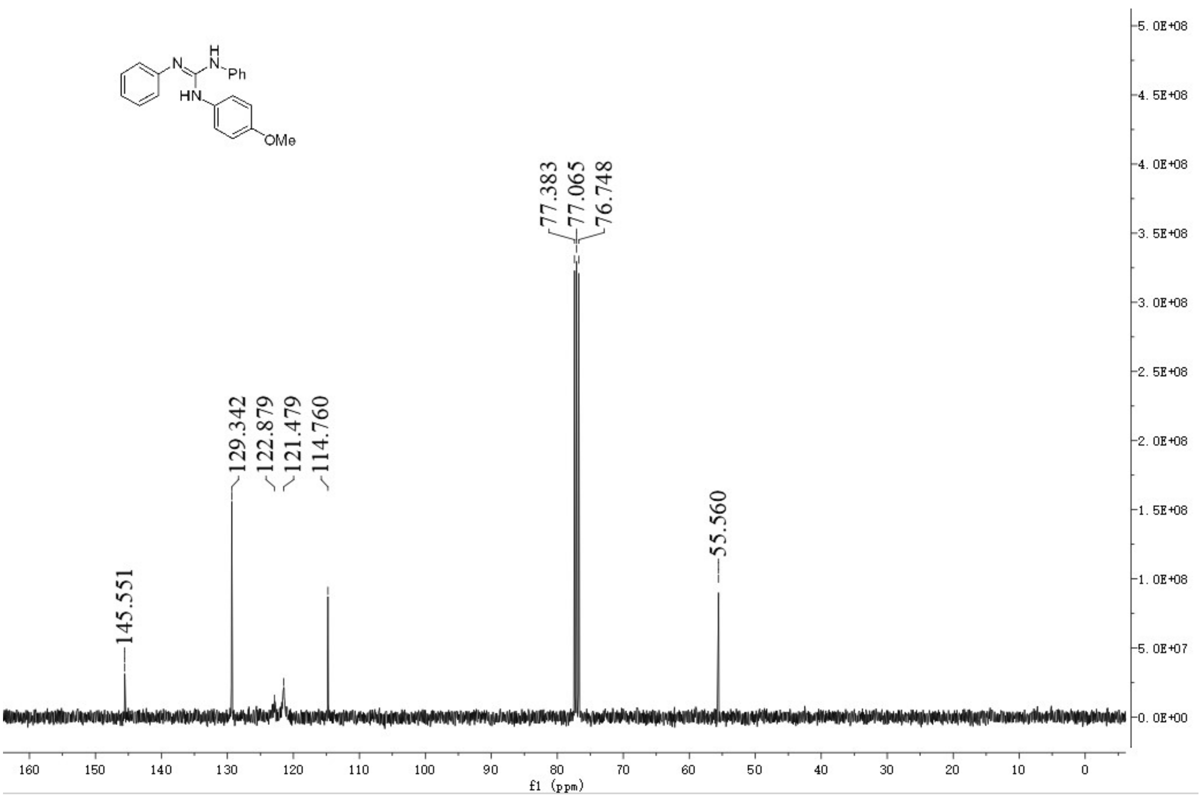

1,3-Bis(4-methoxyphenyl)-2-phenylguanidine (37)

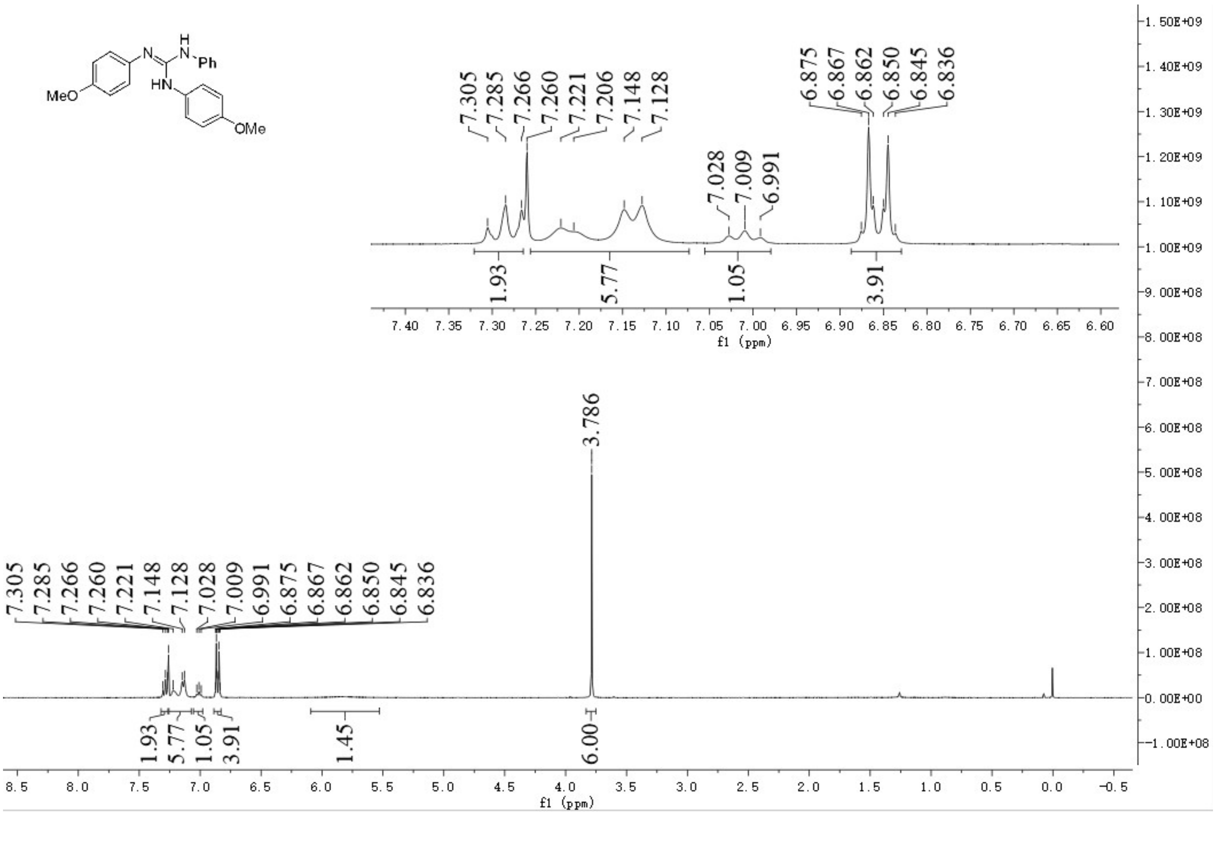

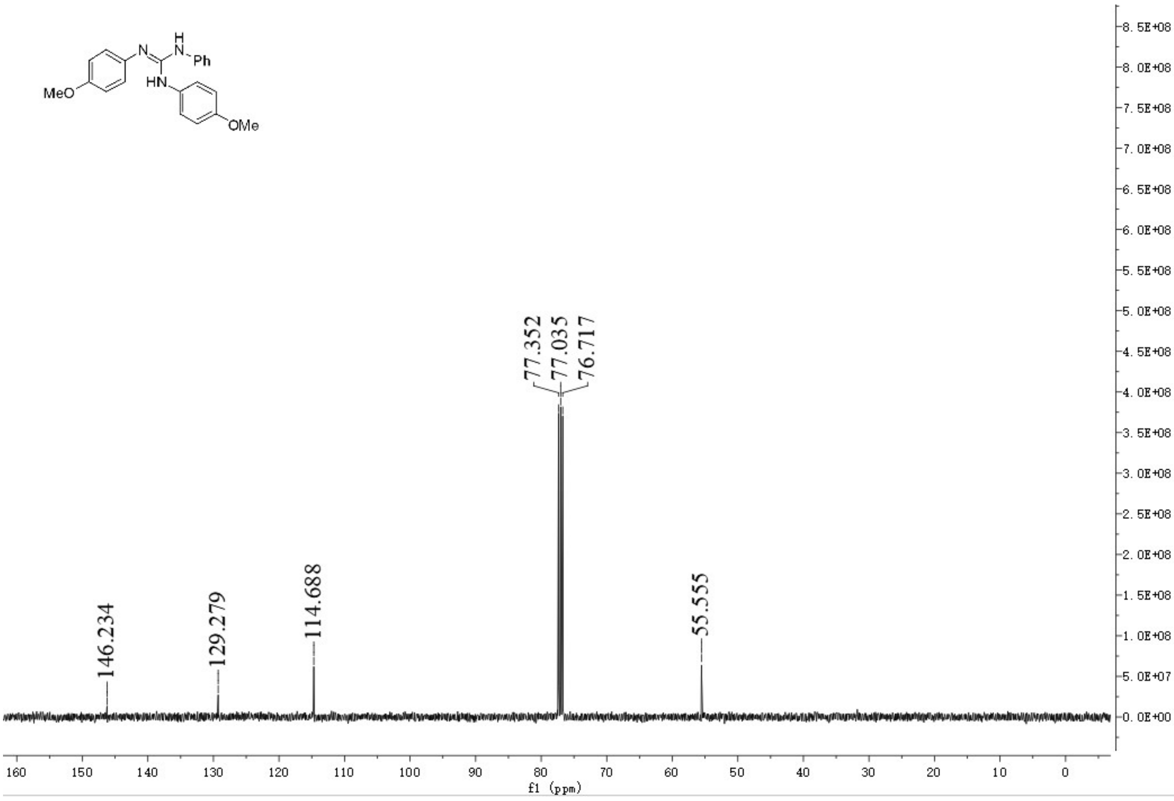

Ethyl-4-((((4-methoxyphenyl)amino)(phenylamino)methylene)amino)benzoate (38)

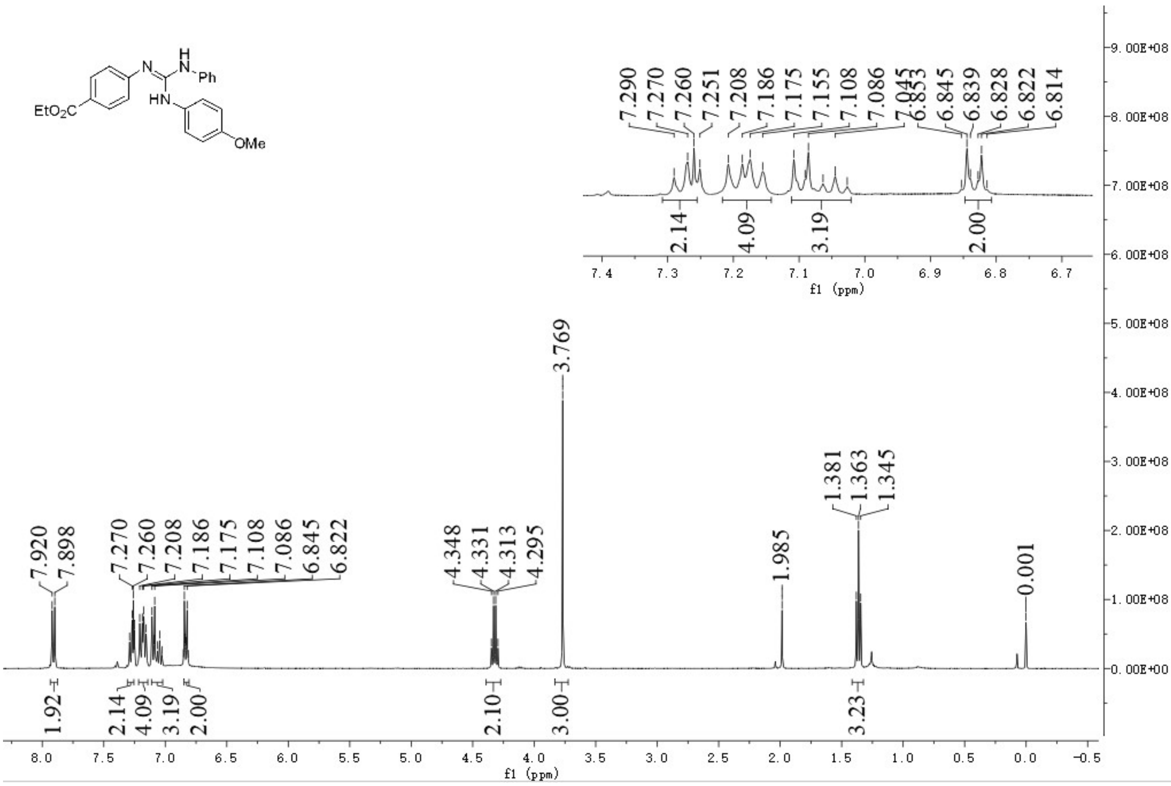

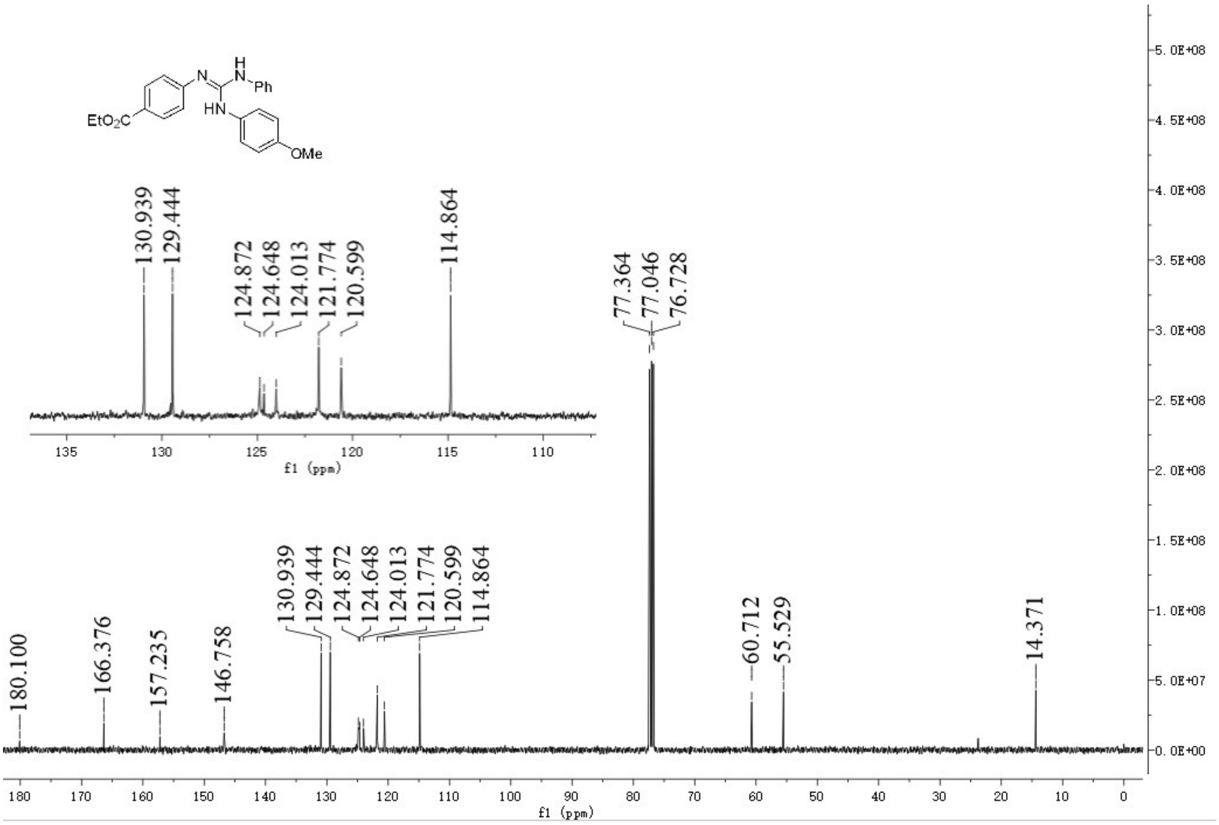

1,3-Diphenyl-2-(pyridin-2-yl)guanidine (39)

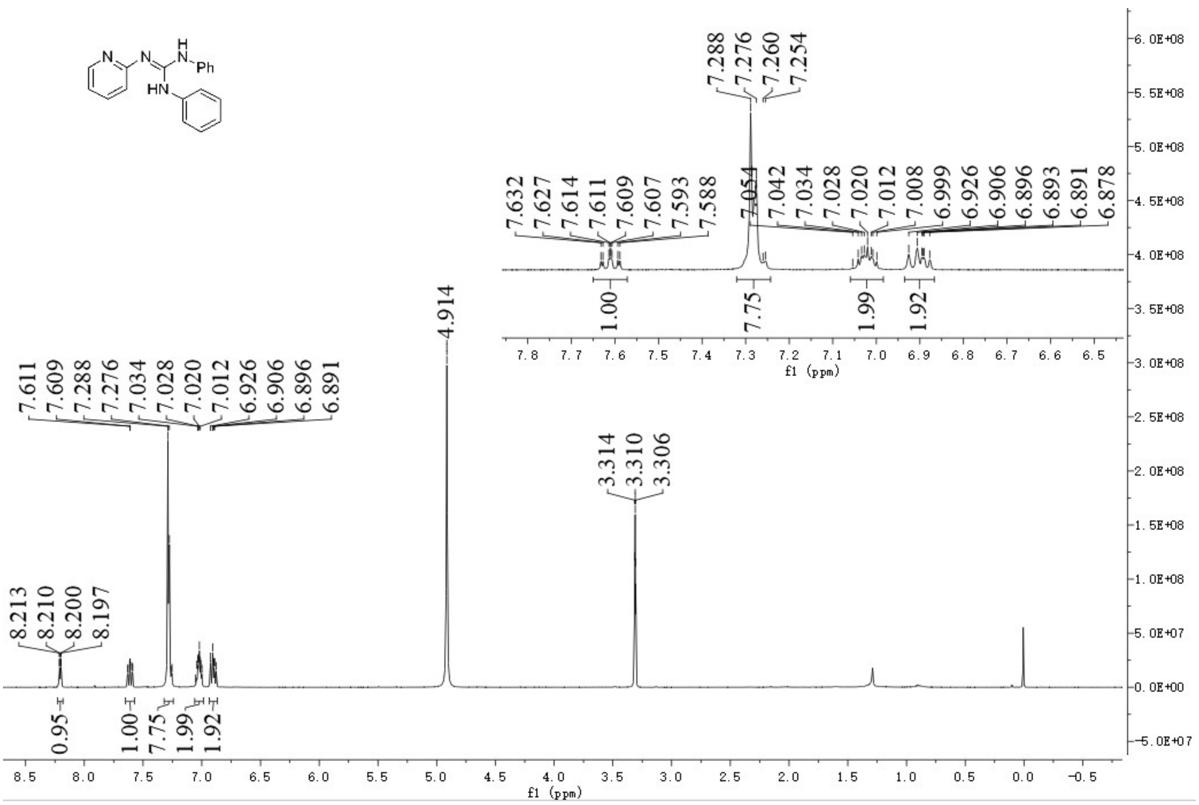

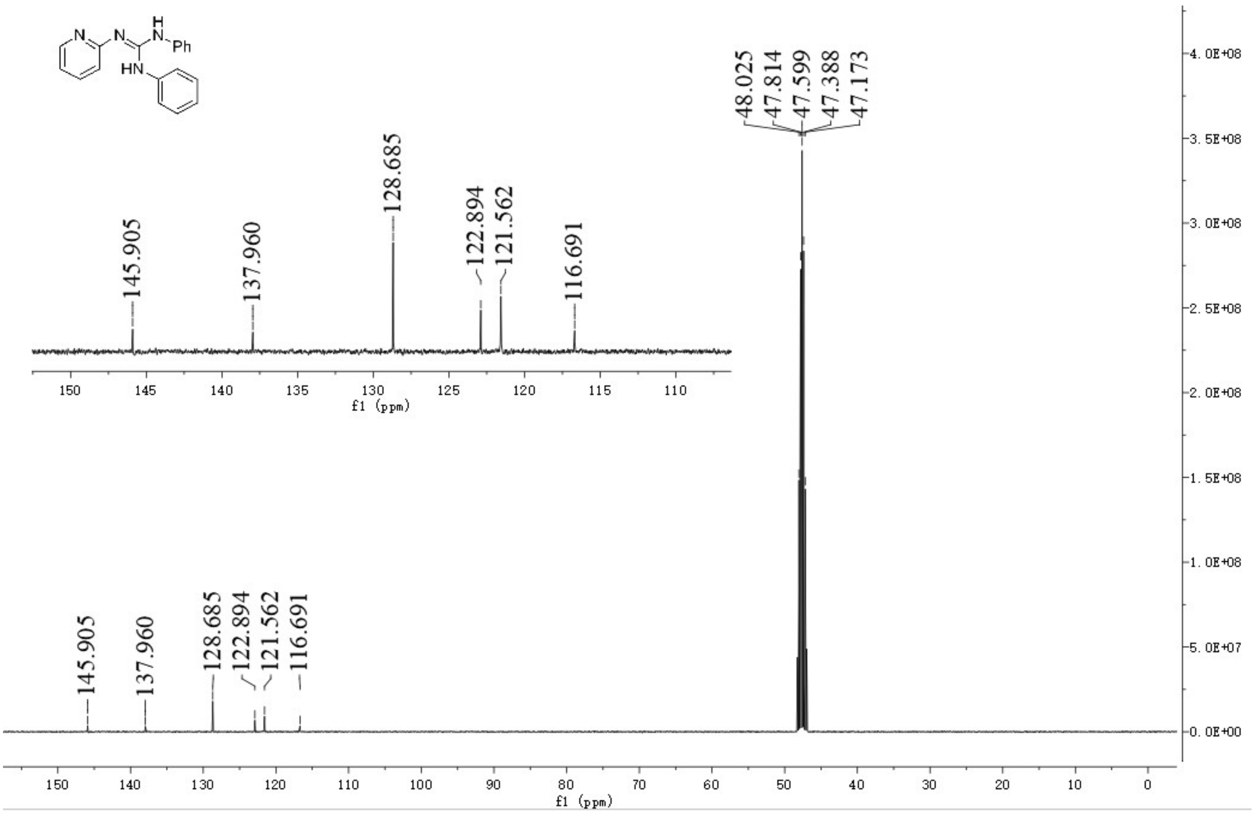

N,*N′*-Diphenylcarbamimidoyl)glycine (40)

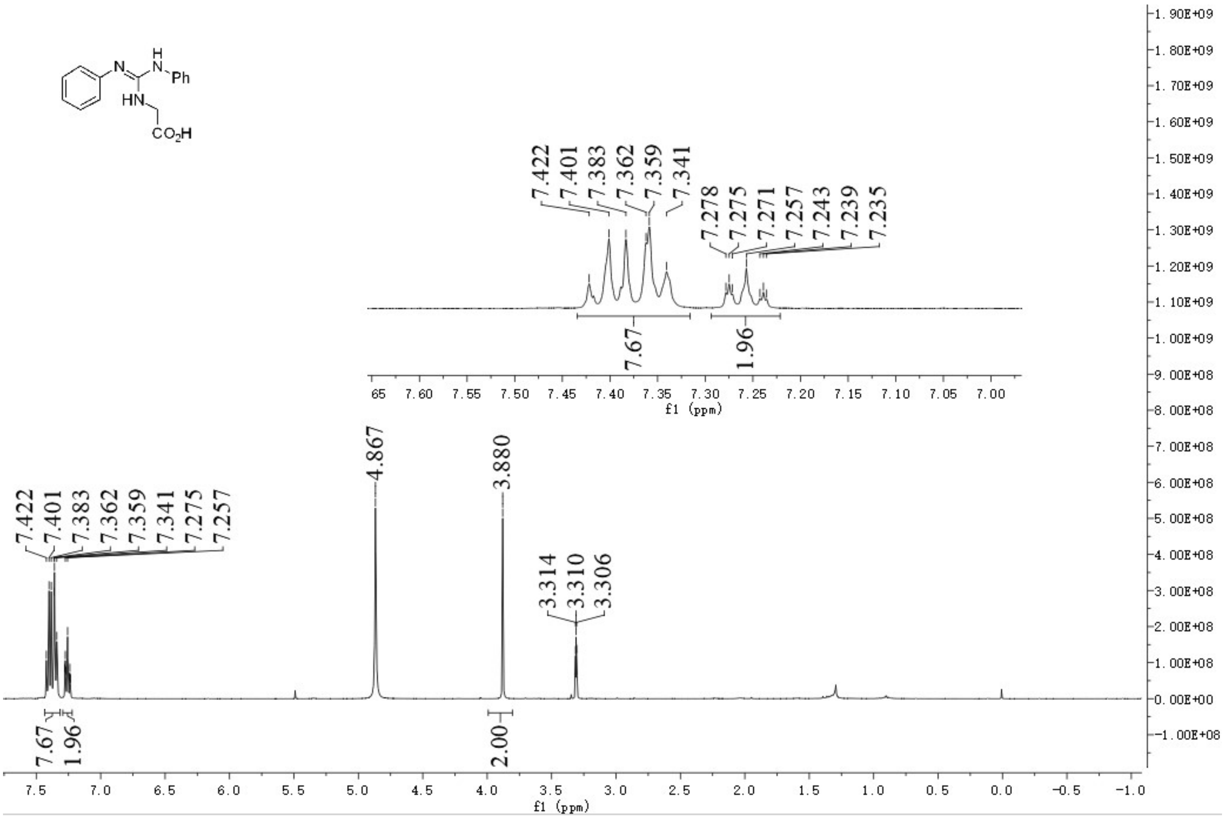

N,N′-Diphenylcarbamimidoyl)alanine (41)

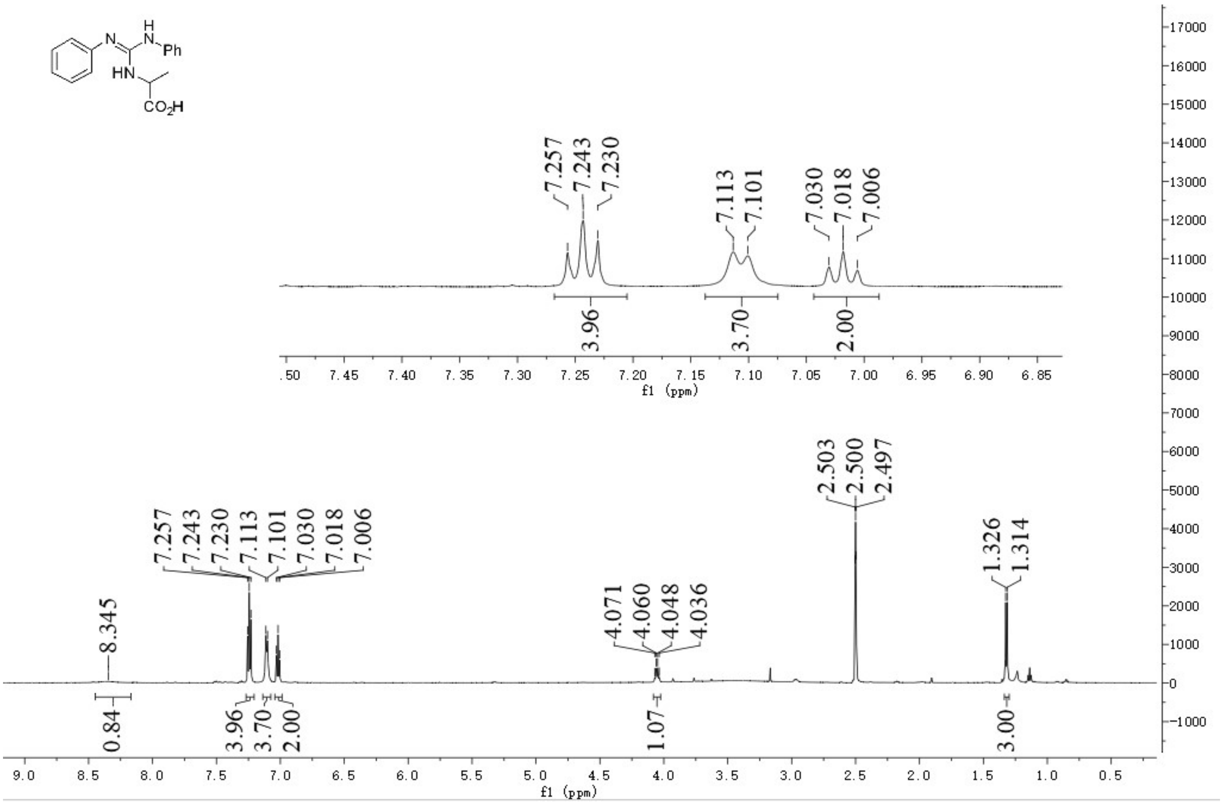

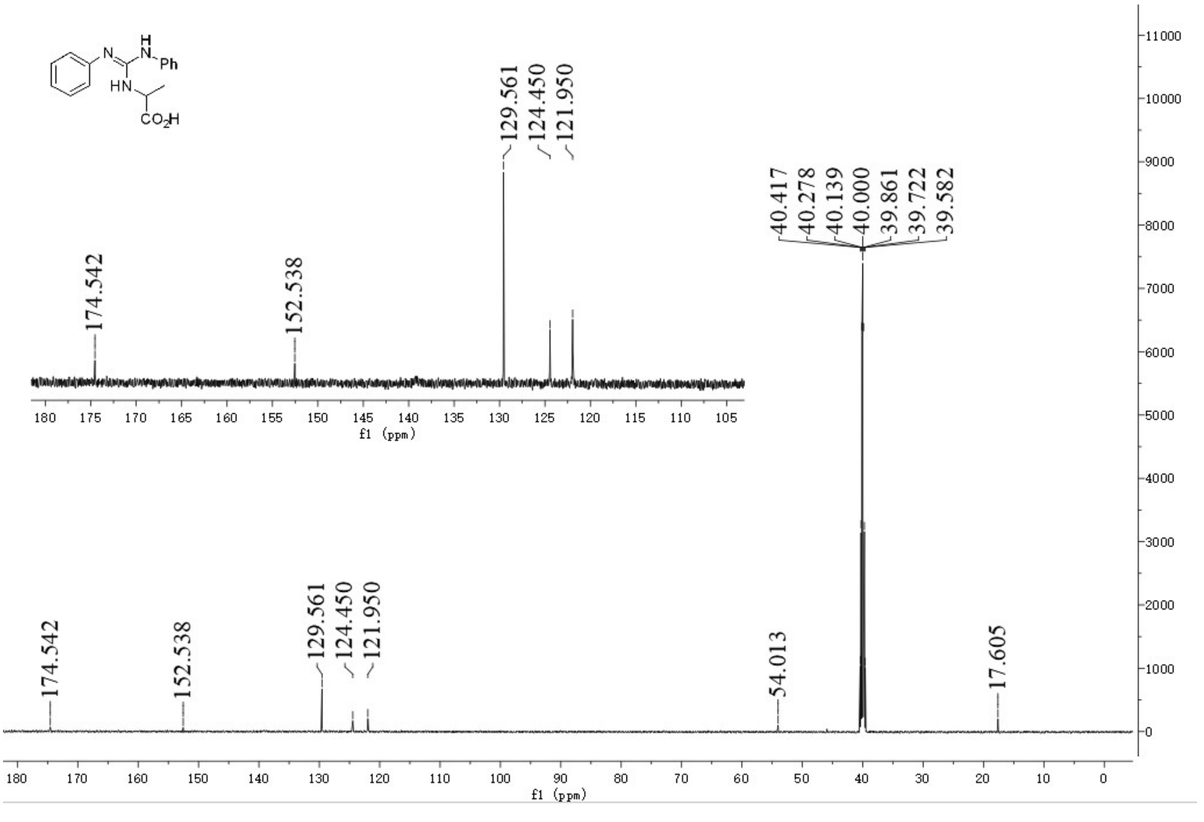

1-(2,3-Diphenylguanidino)cyclopropane-1-carboxylic acid (42)

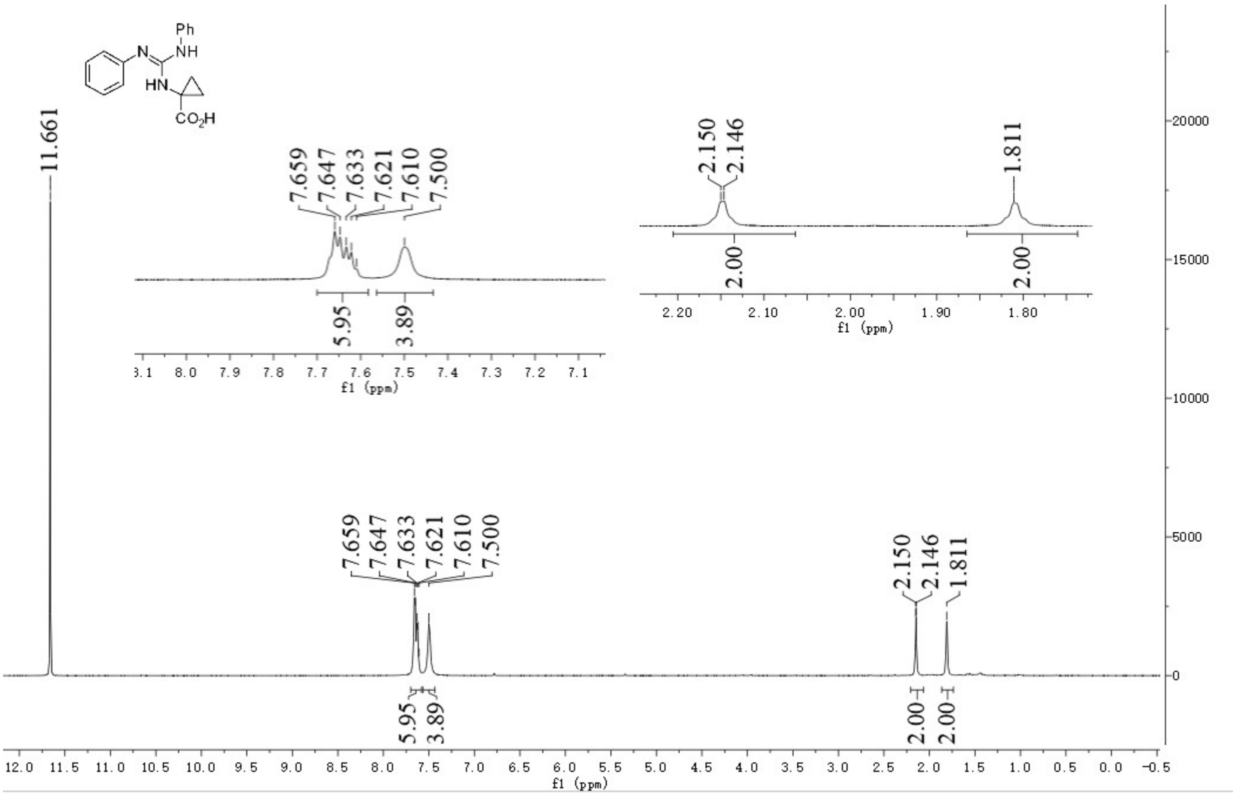

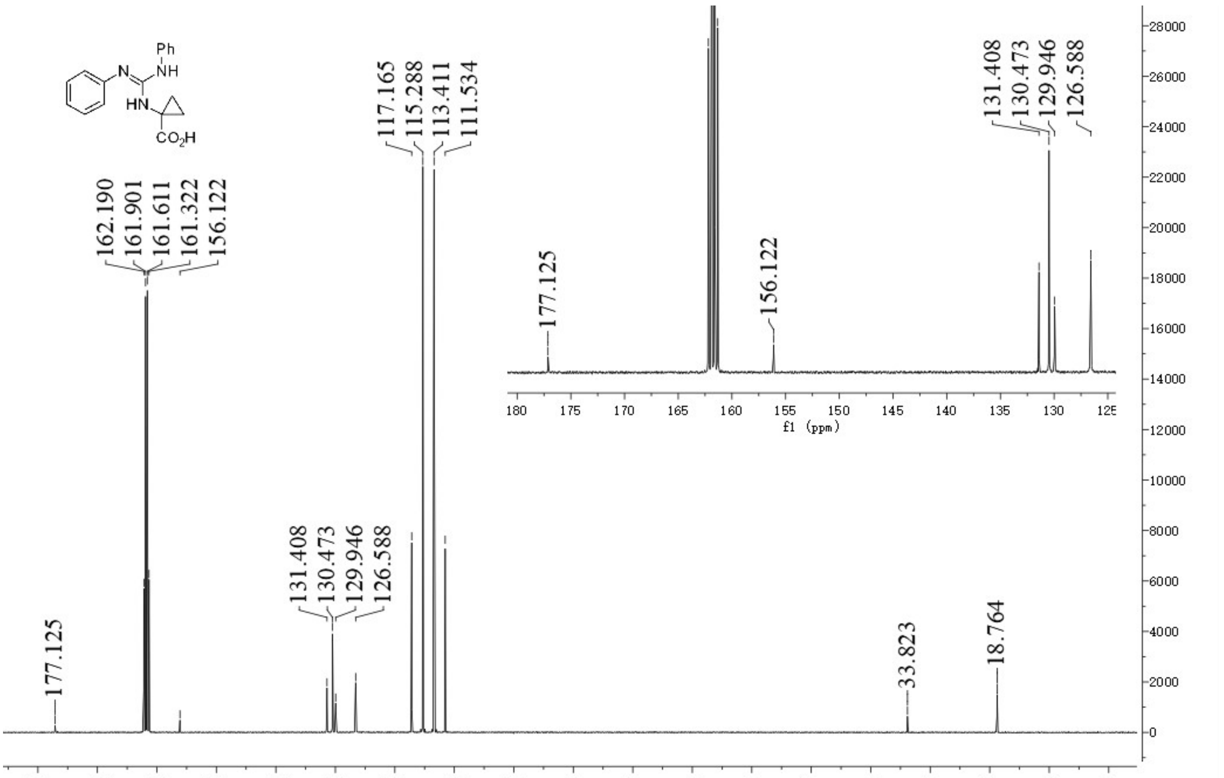

(*N*,*N′*-Diphenylcarbamimidoyl)proline (43)

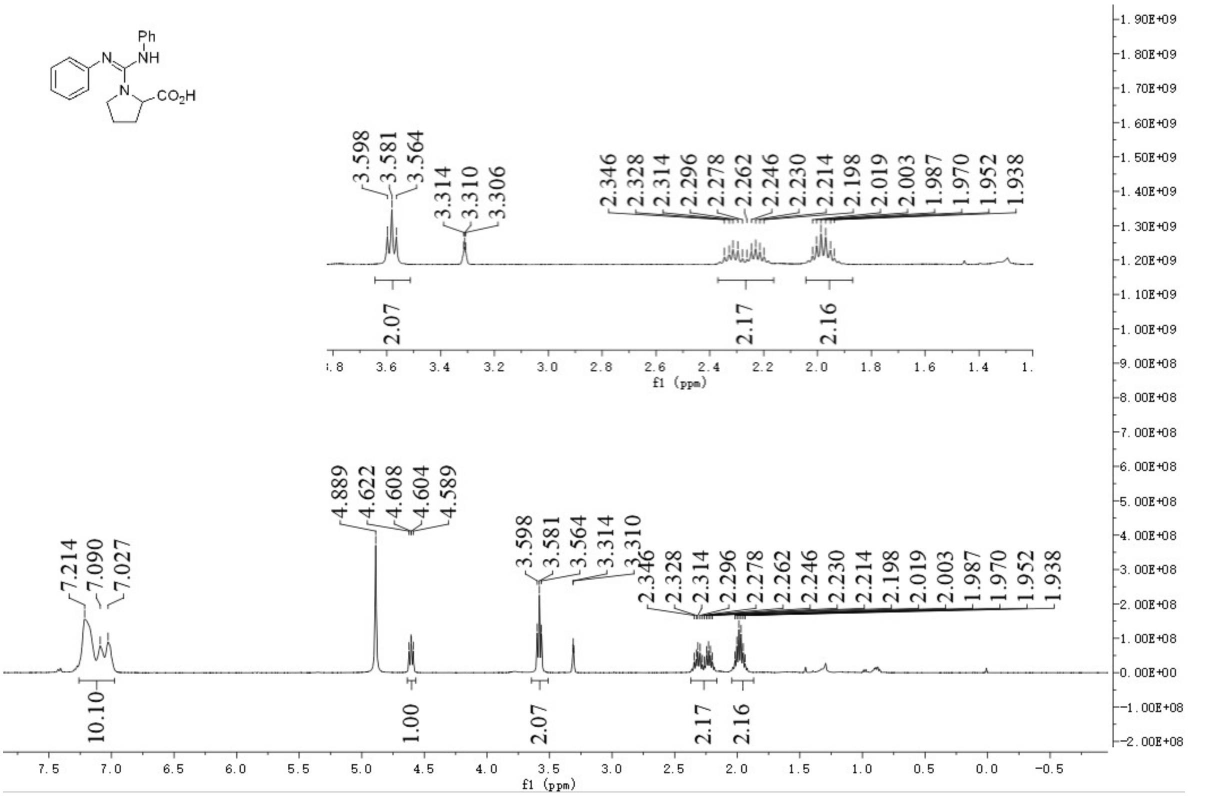

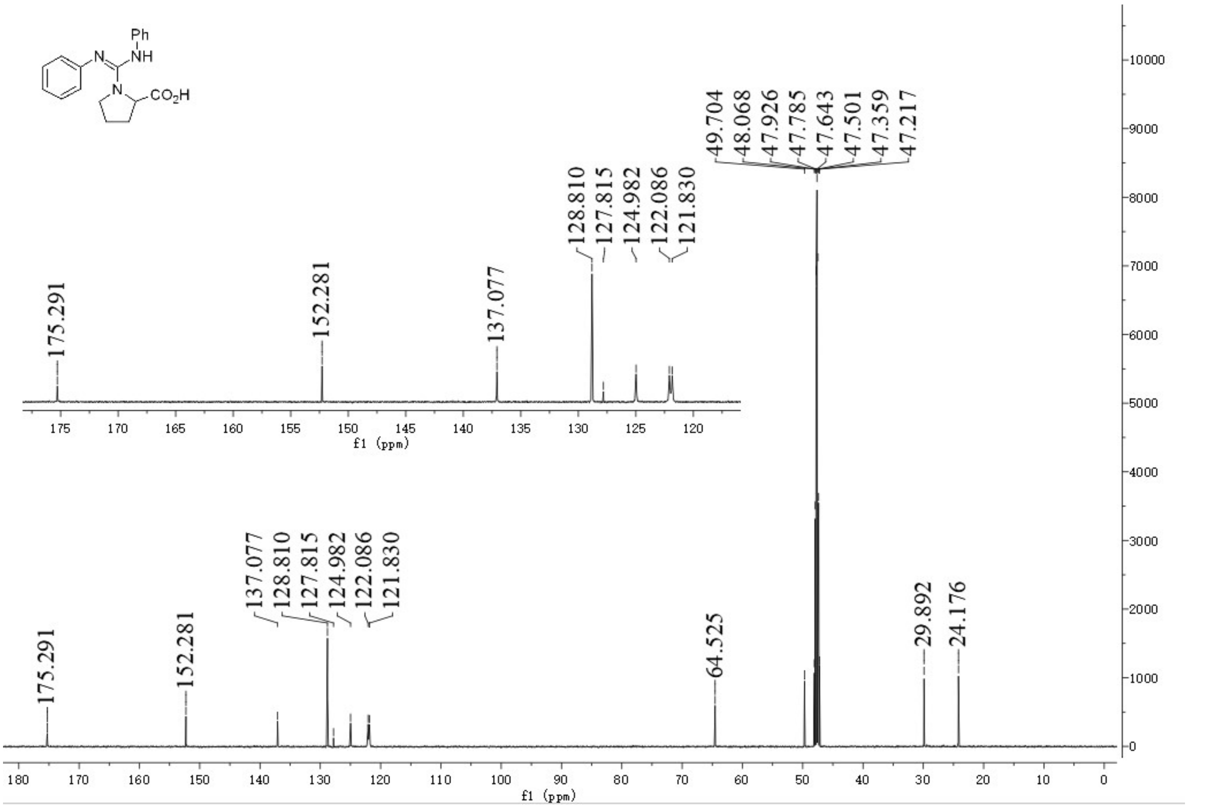

2-(2,3-Diphenylguanidino)-2-methylpropanoic acid (44)

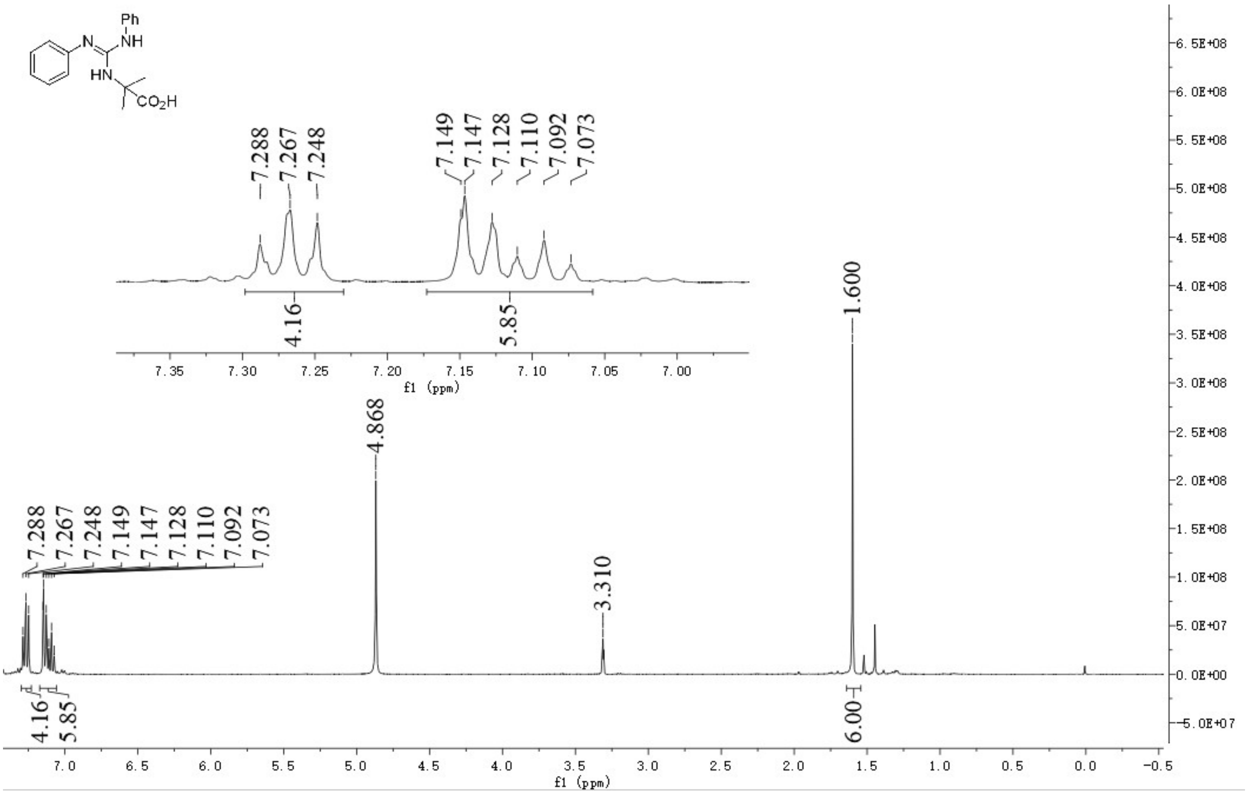

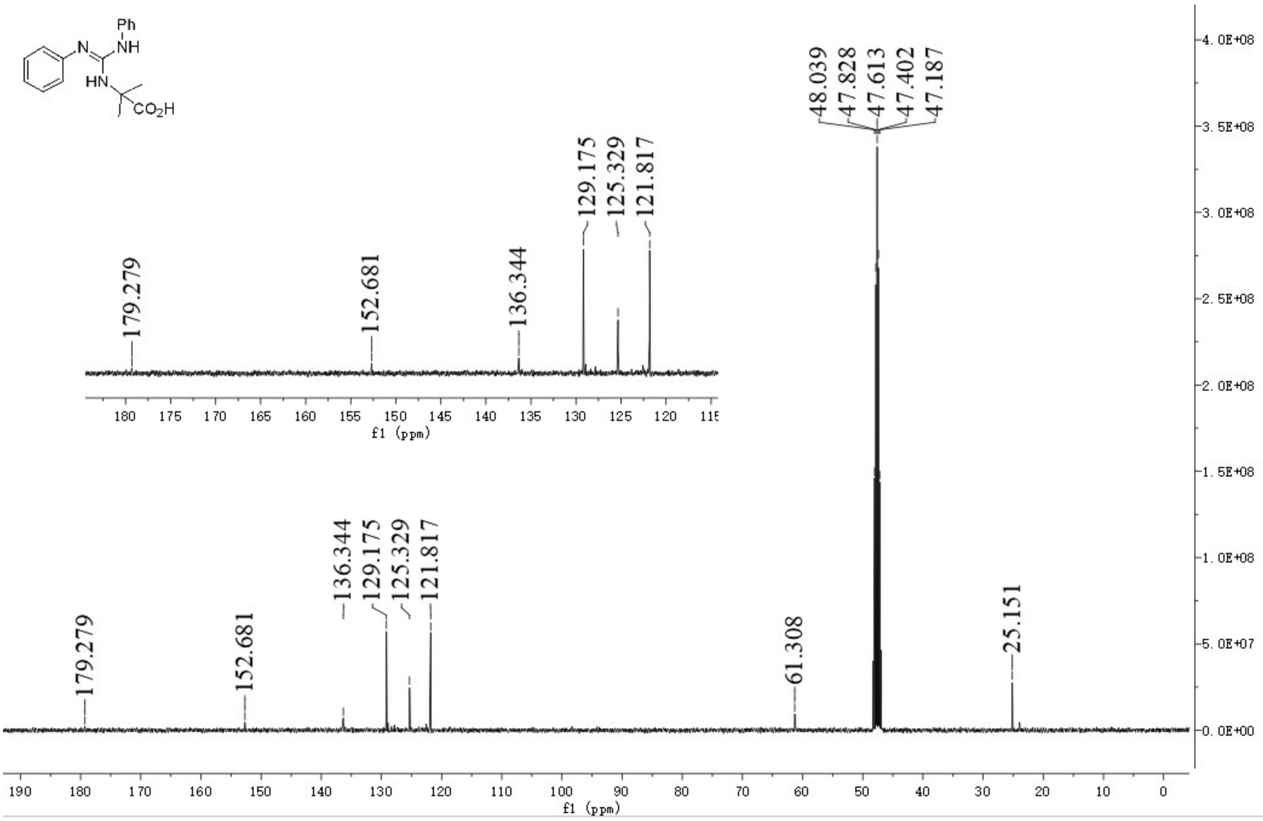

(*N*,*N'*-Diphenylcarbamimidoyl)methionine (45)

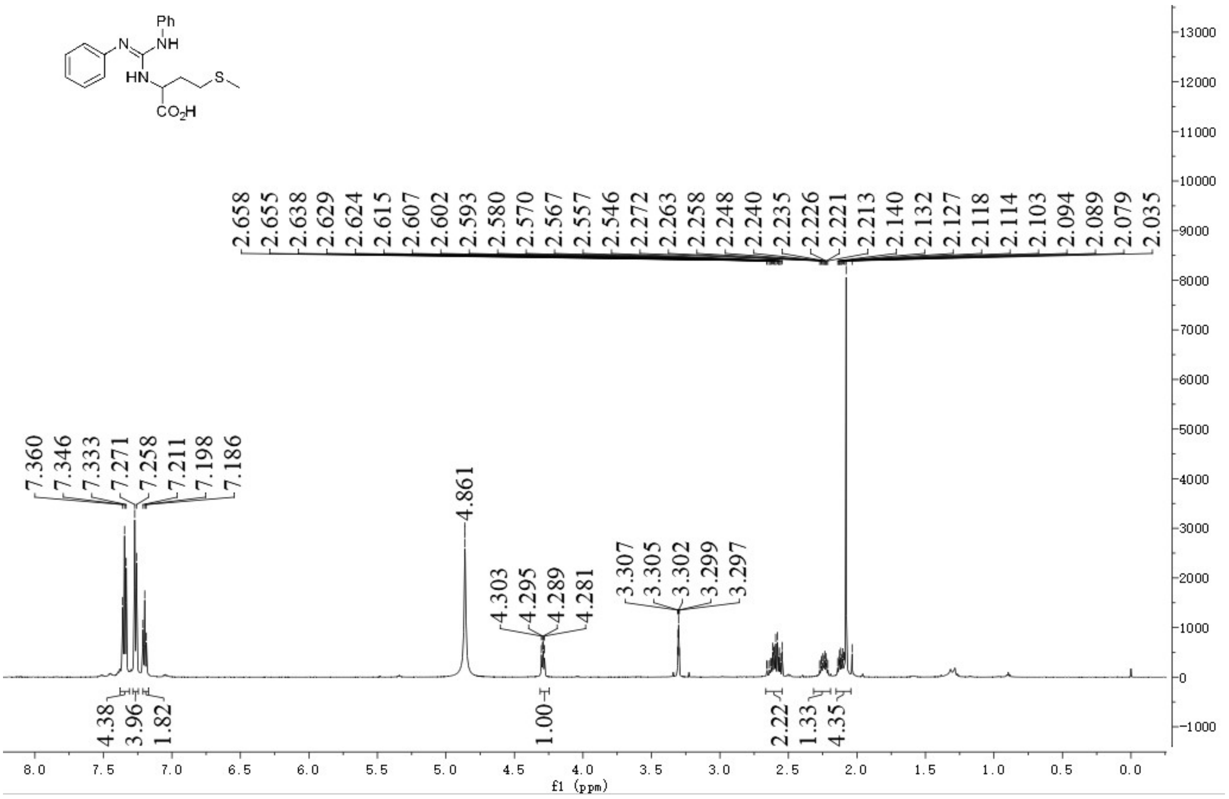

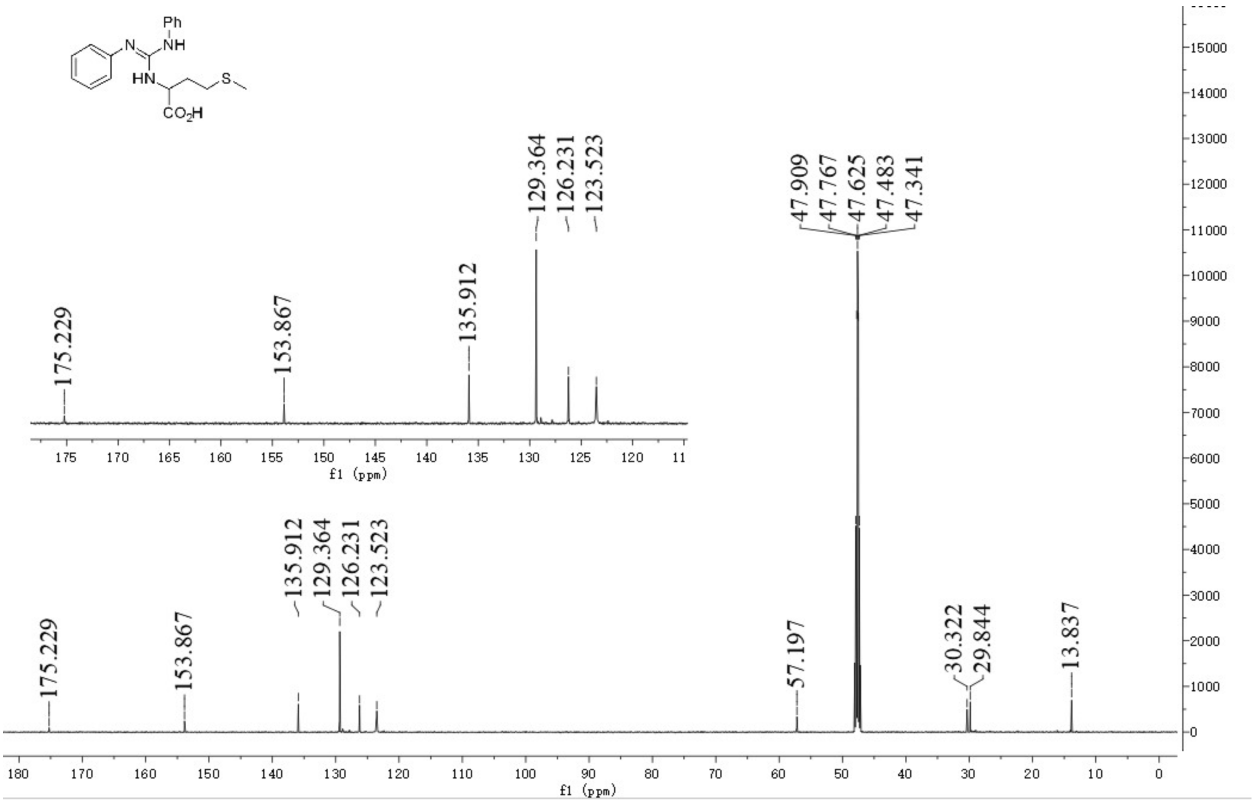

2-(2,3-Diphenylguanidino)-4-(methylsulfinyl)butanoic acid (45')

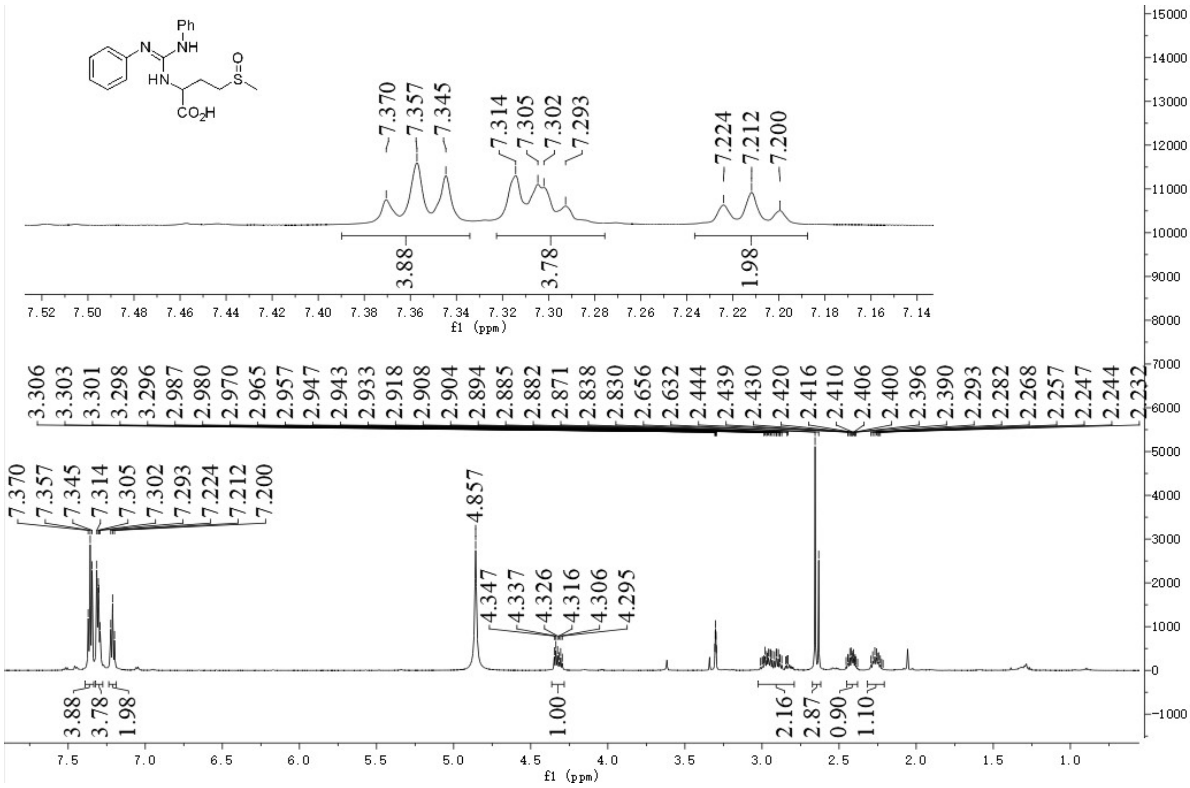

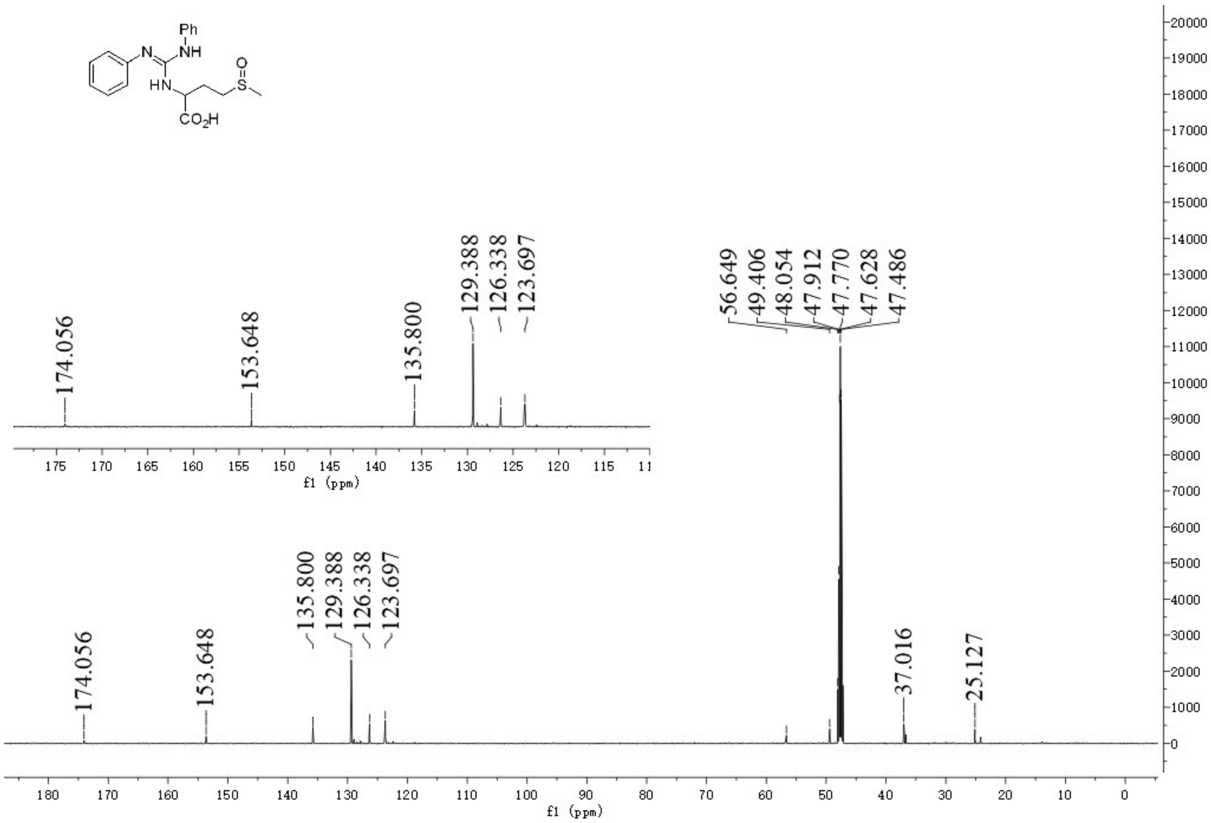

***N*-(*N*,*N′*-Diphenylcarbamimidoyl)-N-methylglycine (46)**

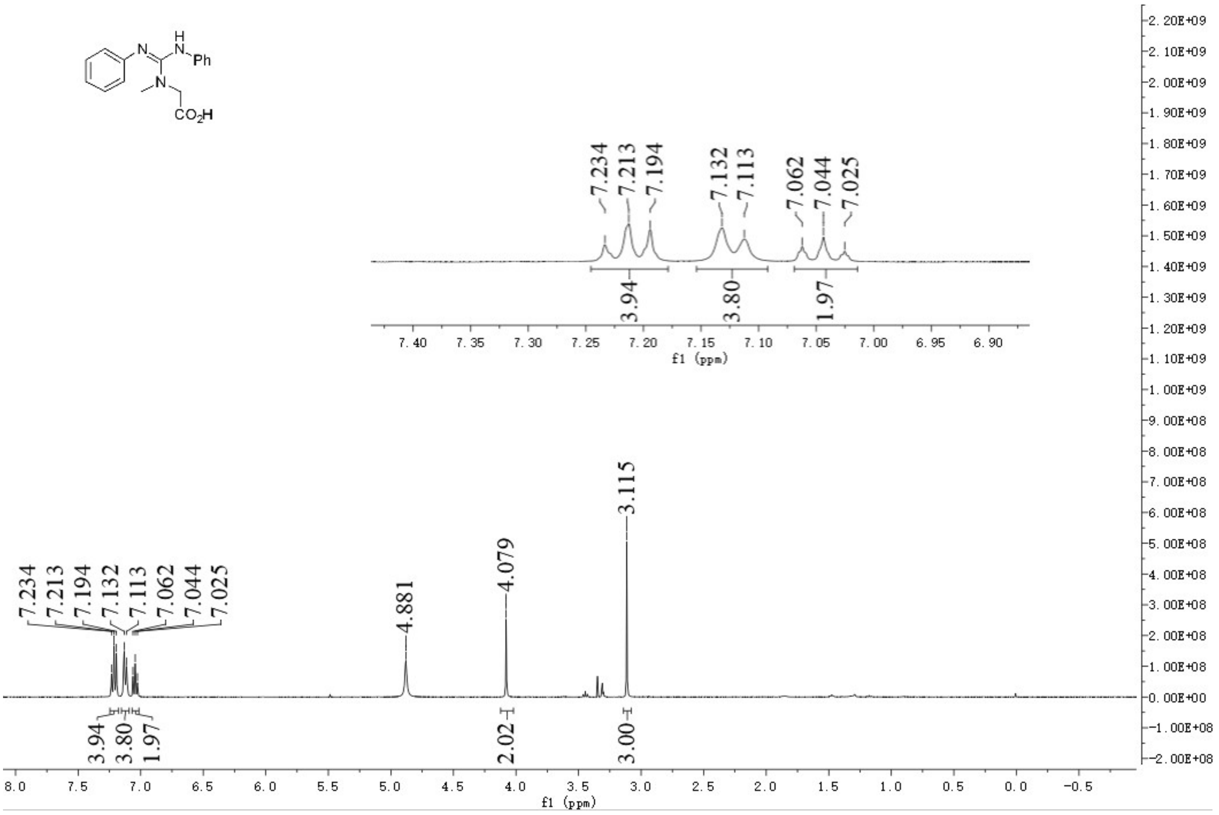

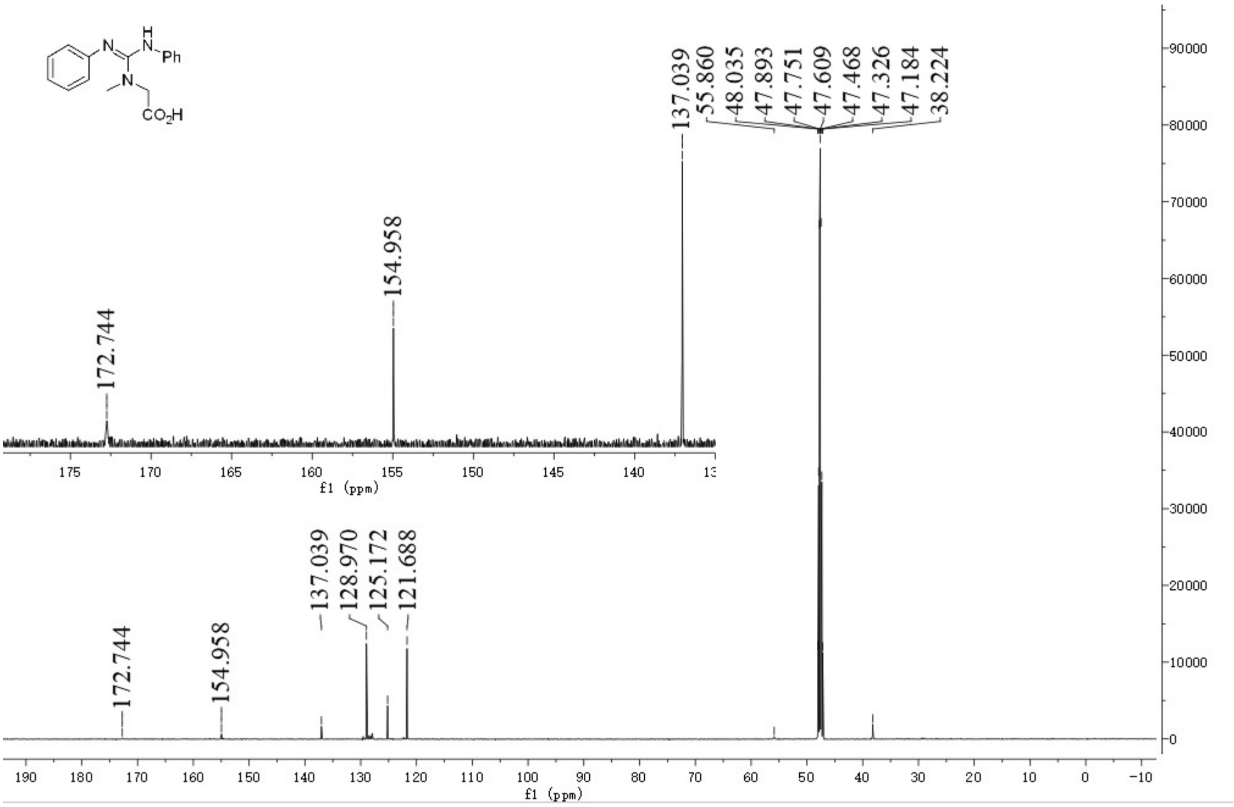

(*N*,*N′*-Diphenylcarbamimidoyl)valine (47)

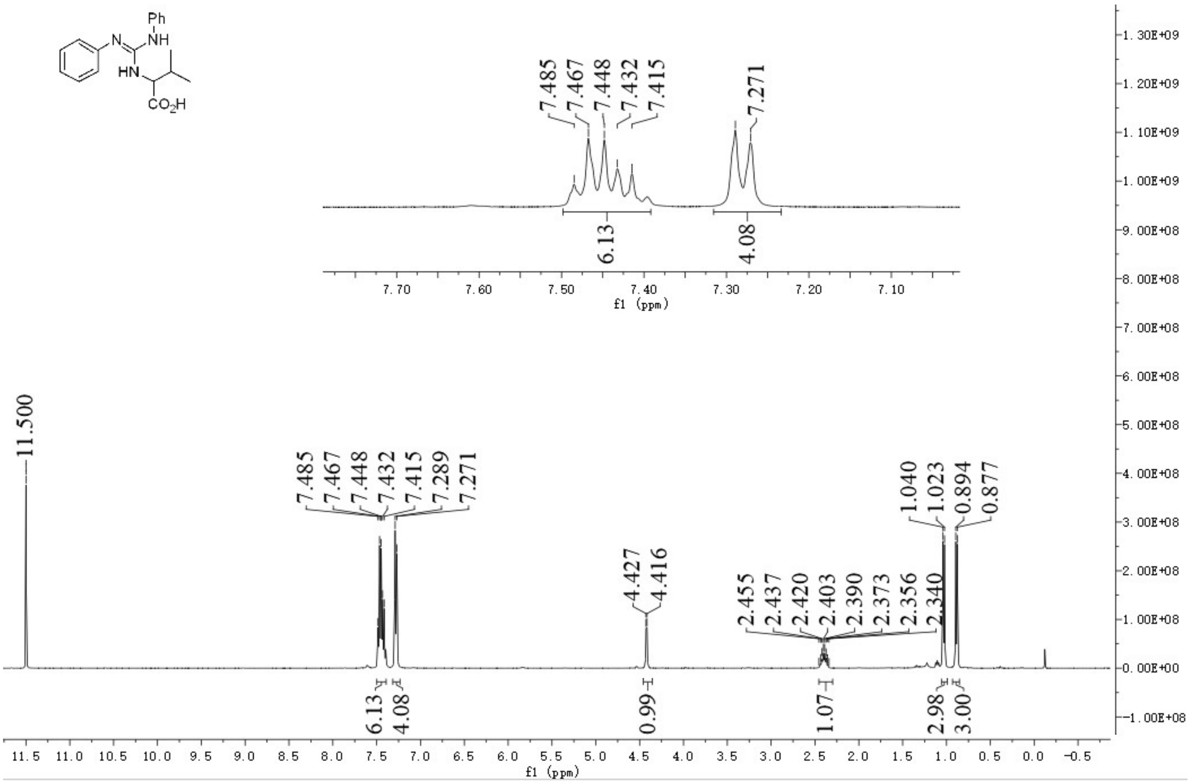

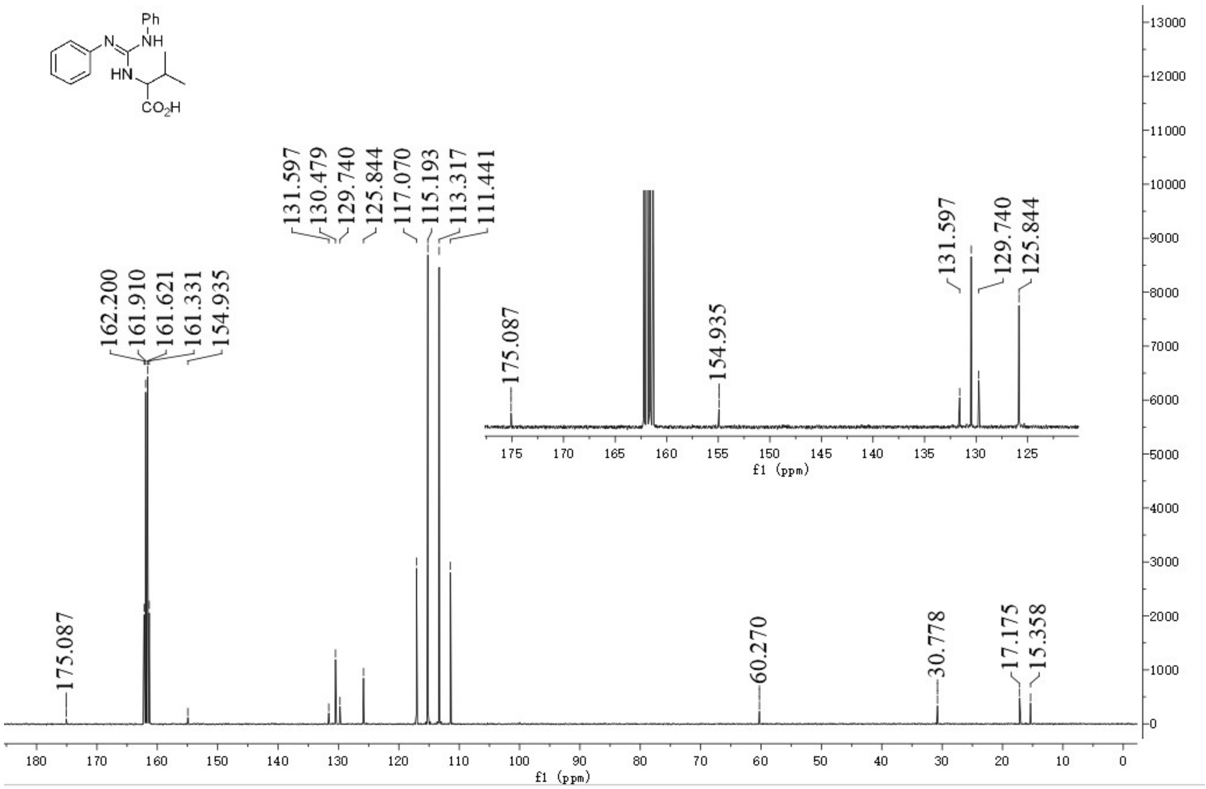

(*N*,*N′*-Diphenylcarbamimidoyl)leucine (48)

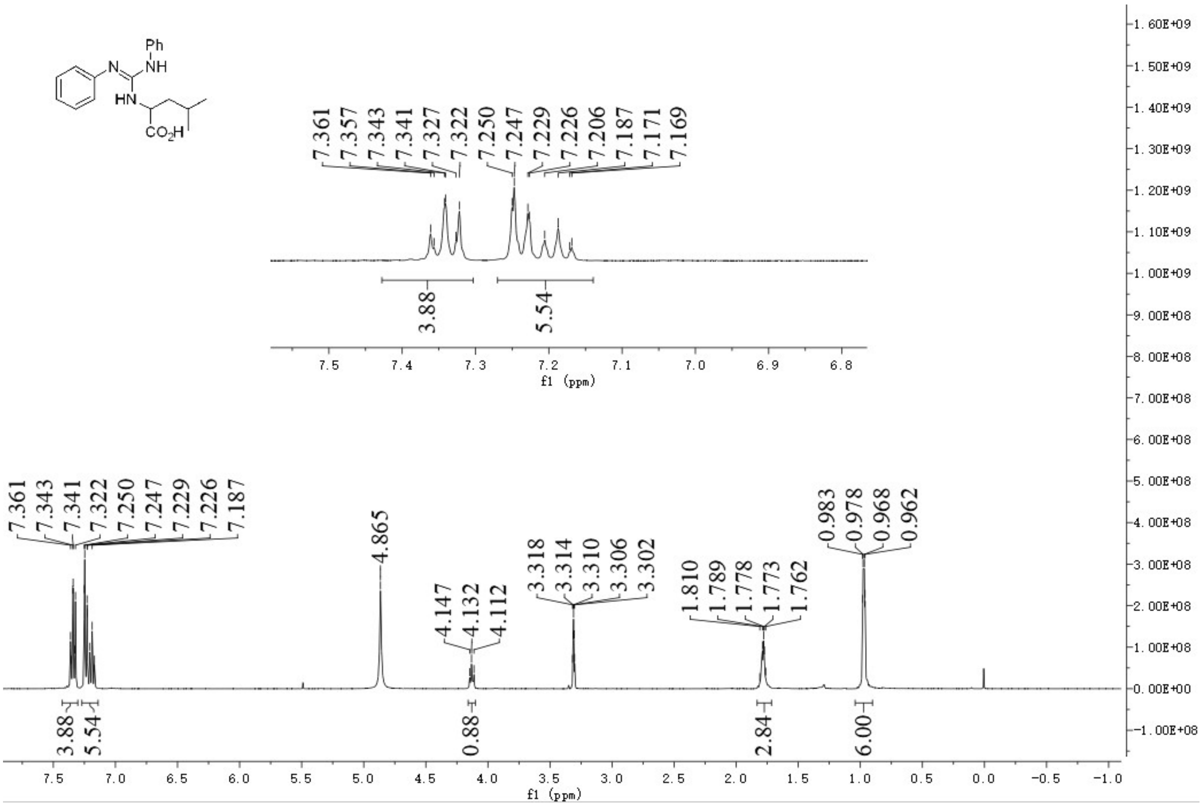

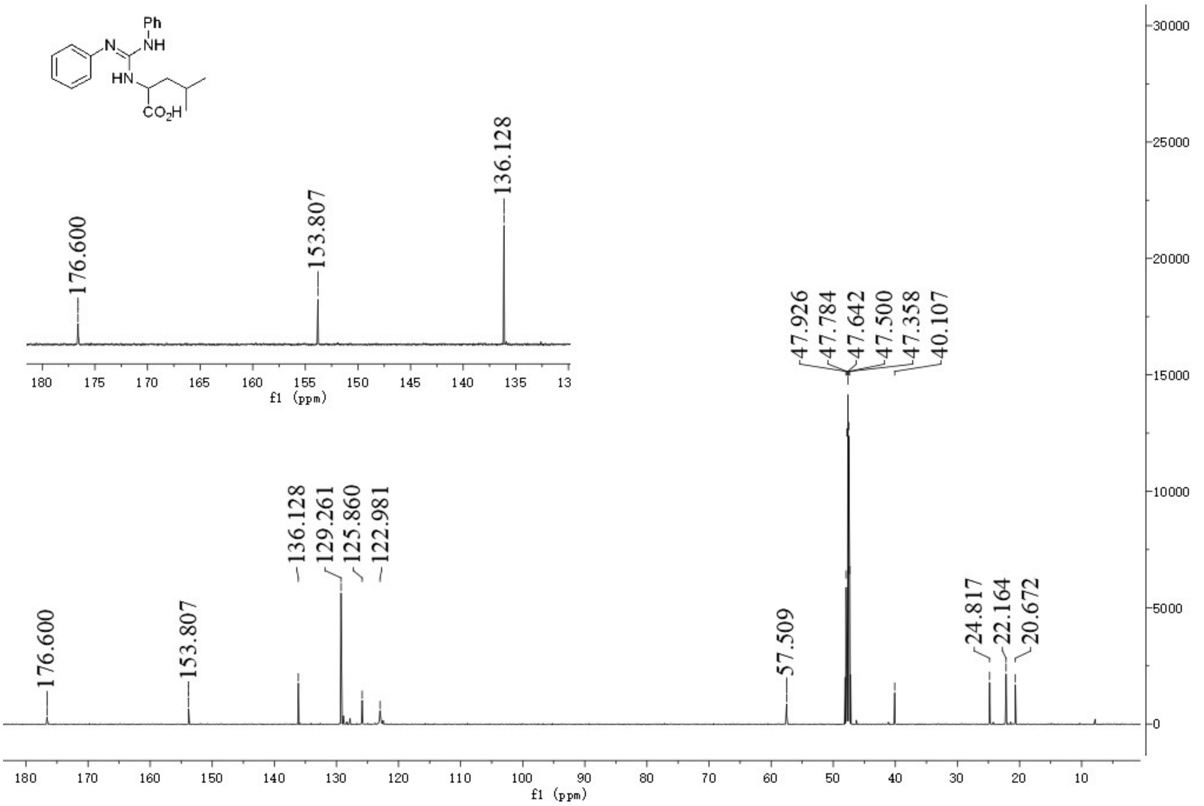

(*N*,*N′*-Diphenylcarbamimidoyl)phenylalanine (49)

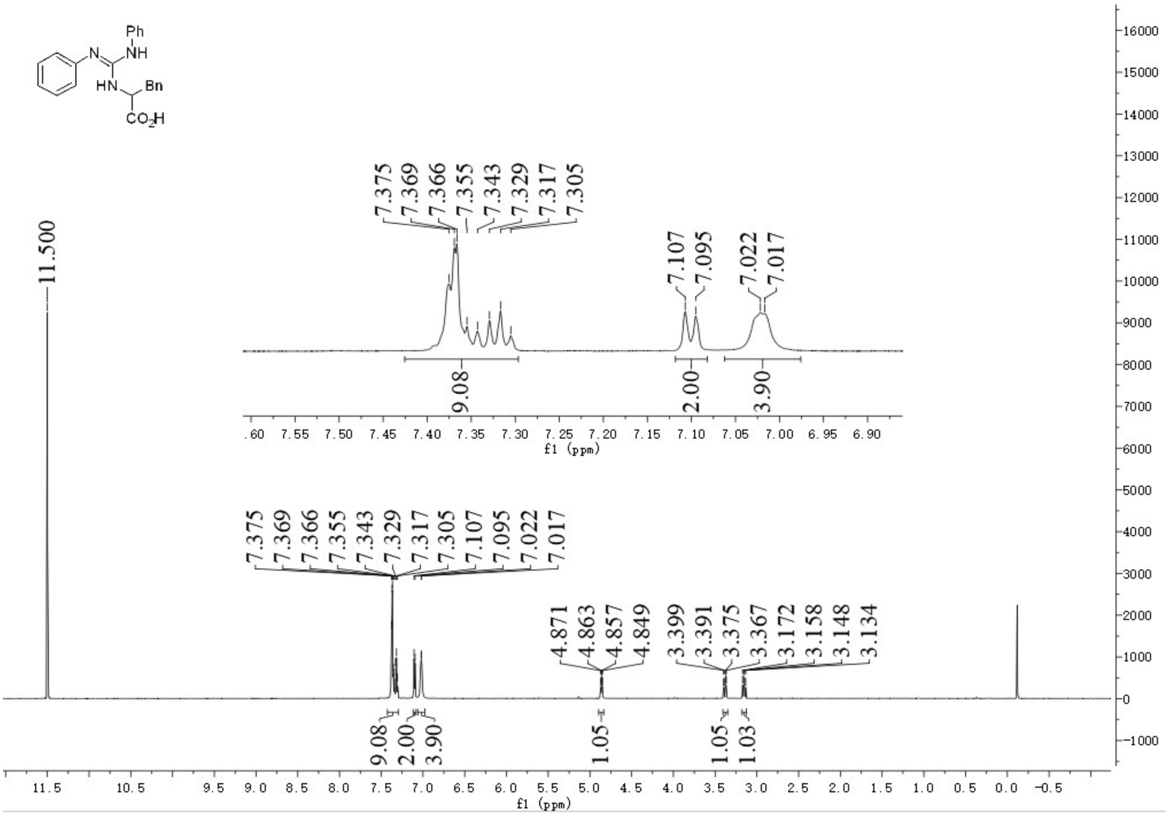

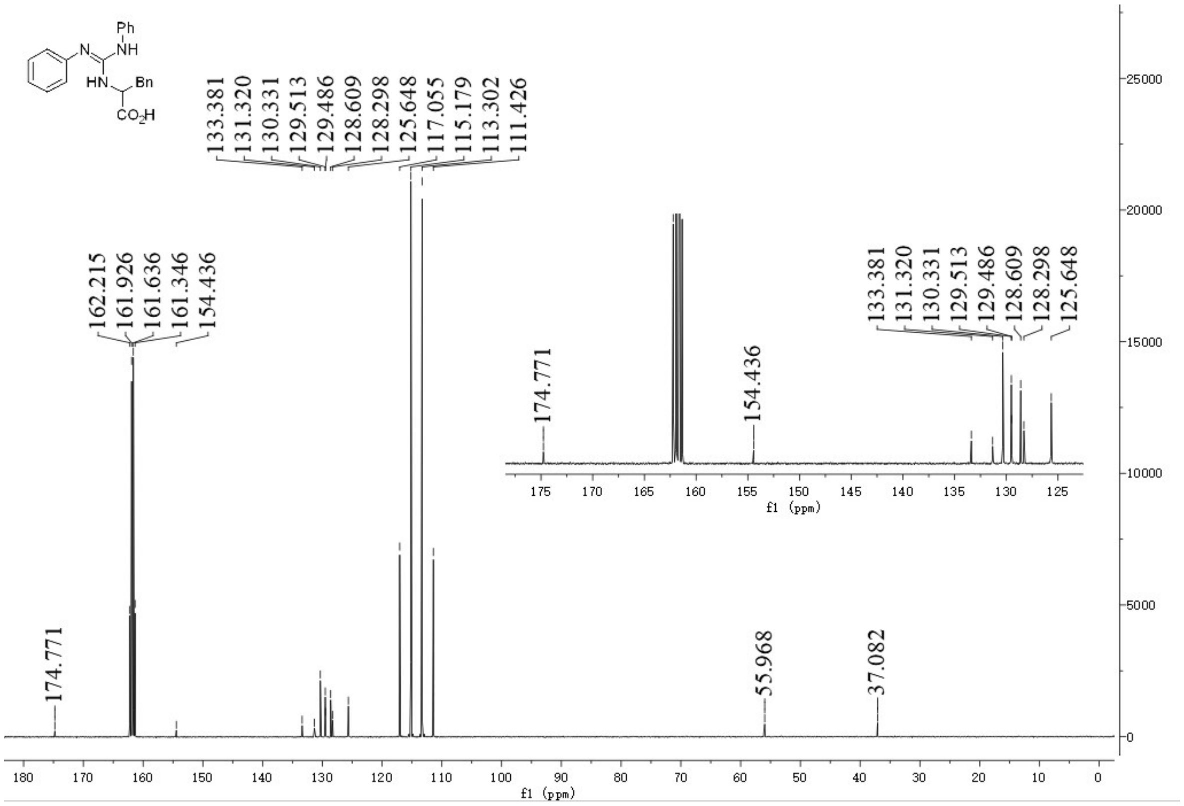

N-Phenyl-1*H*-benzo[*d*]imidazol-2-amine (50)

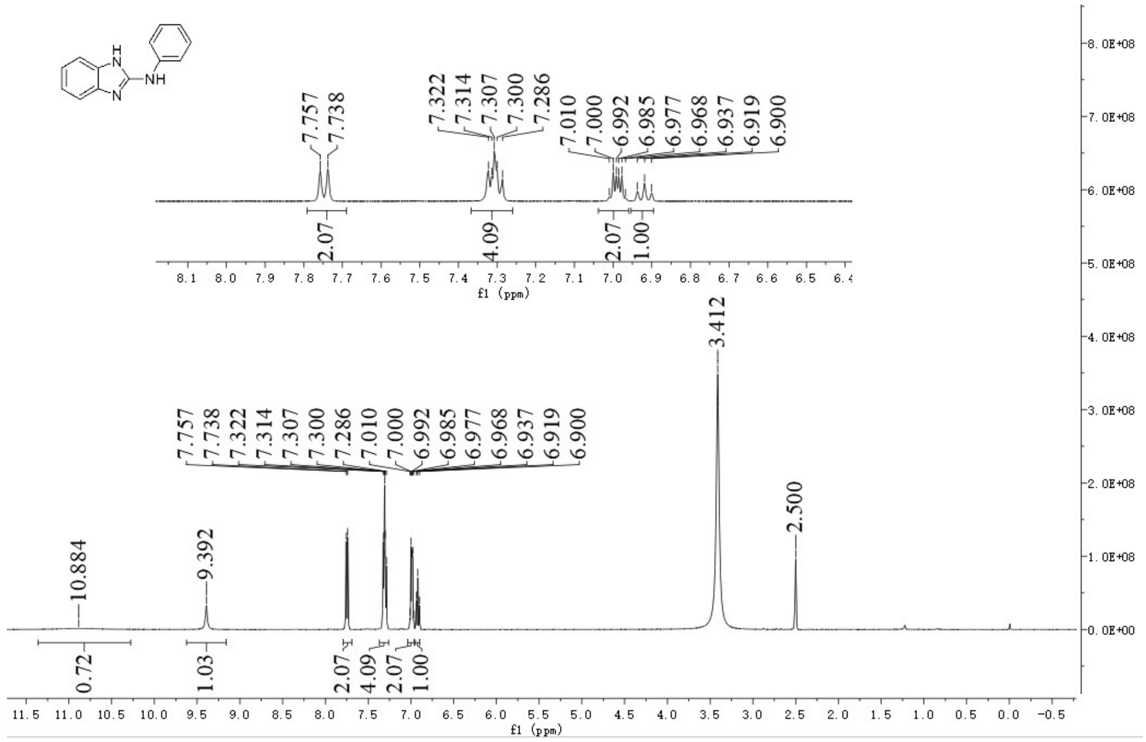

5,6-Dimethyl-*N*-phenyl-1*H*-benzo[*d*]imidazol-2-amine (51)

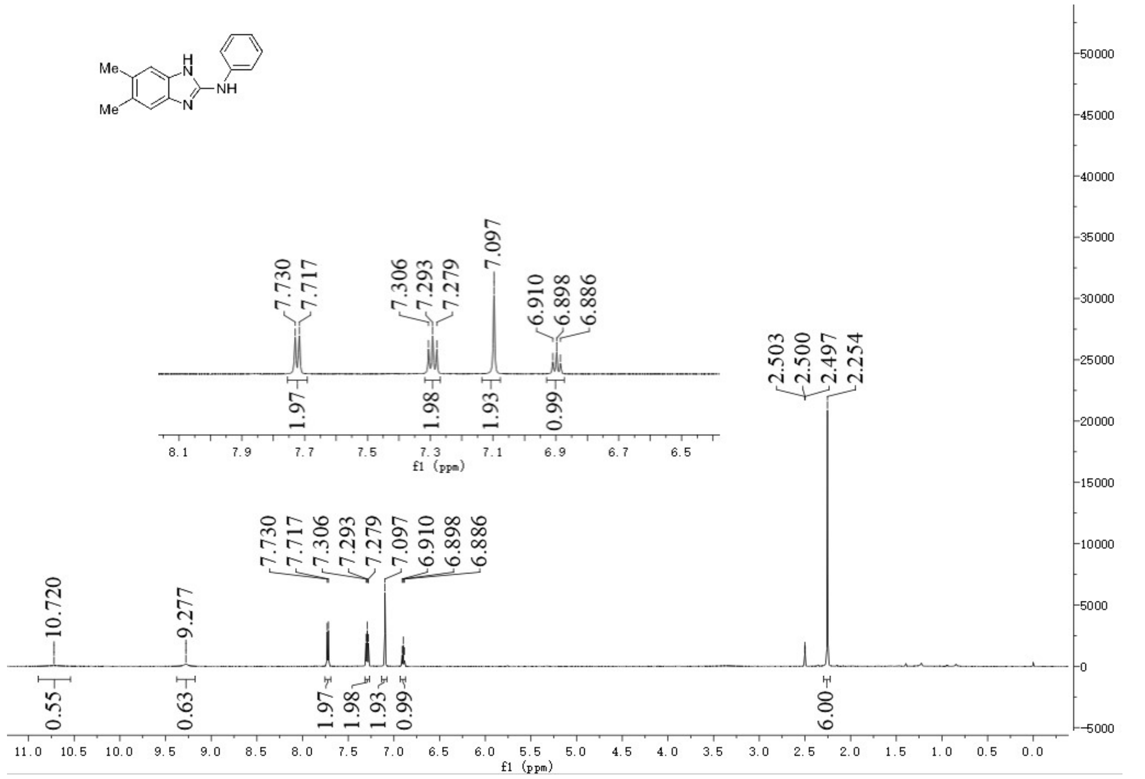

5,6-Dichloro-*N*-phenyl-1*H*-benzo[*d*]imidazol-2-amine (52)

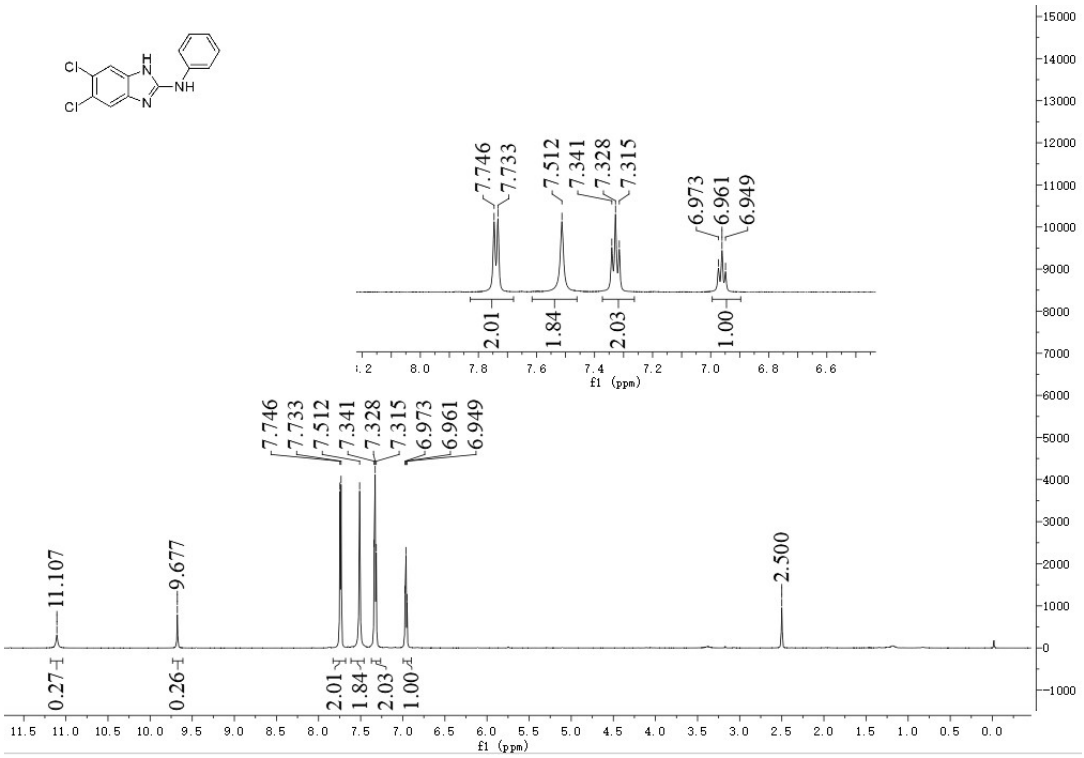

6-Methoxy-*N*-phenyl-1*H*-benzo[*d*]imidazol-2-amine (53)

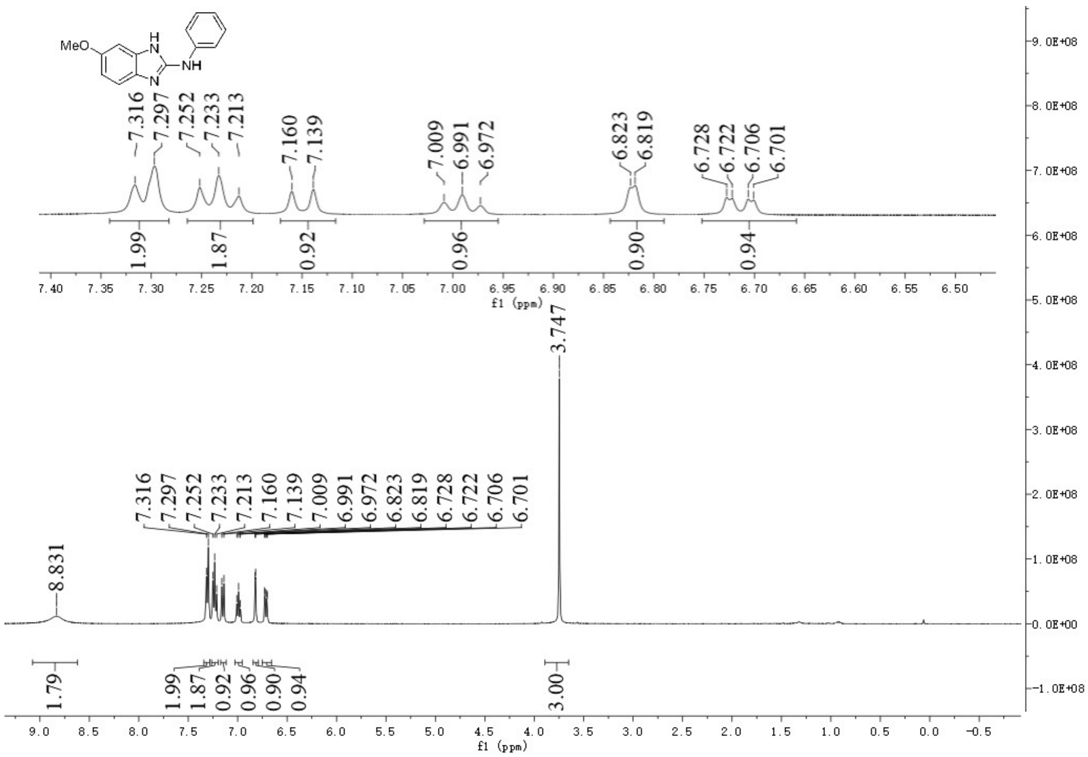

4-((1*H*-Benzo[*d*]imidazol-2-yl)amino)benzonitrile (54)

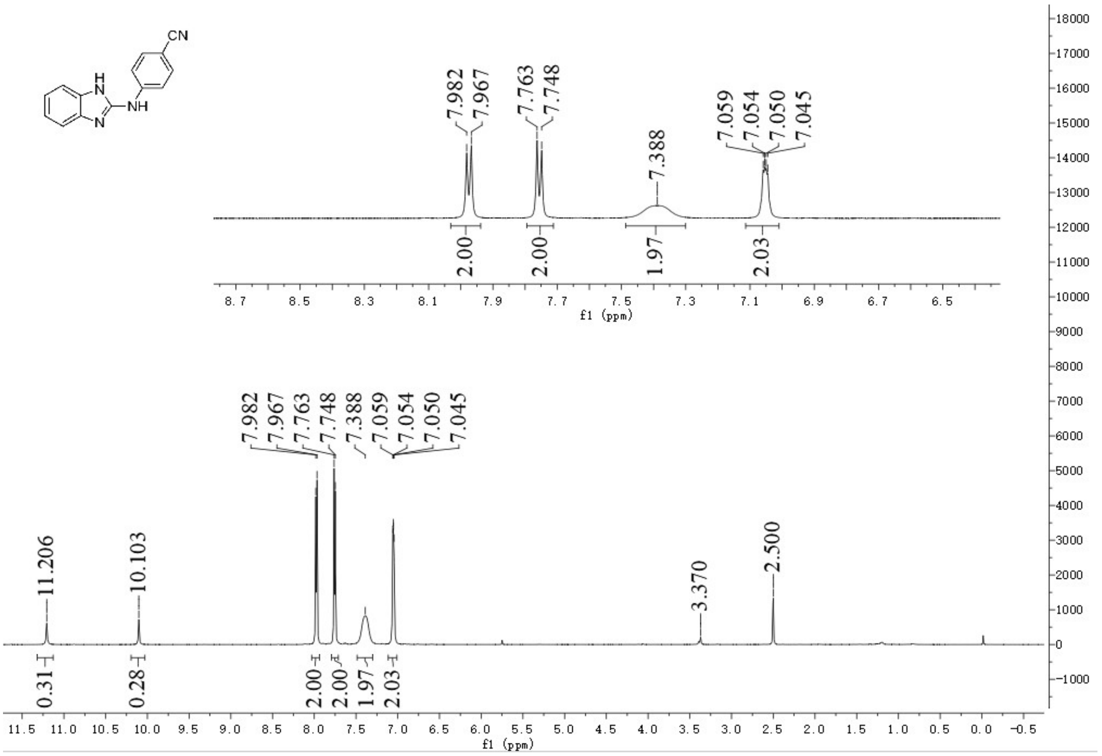

***N*-(3-Methoxyphenyl)-1*H*-benzo[*d*]imidazol-2-amine (55)**

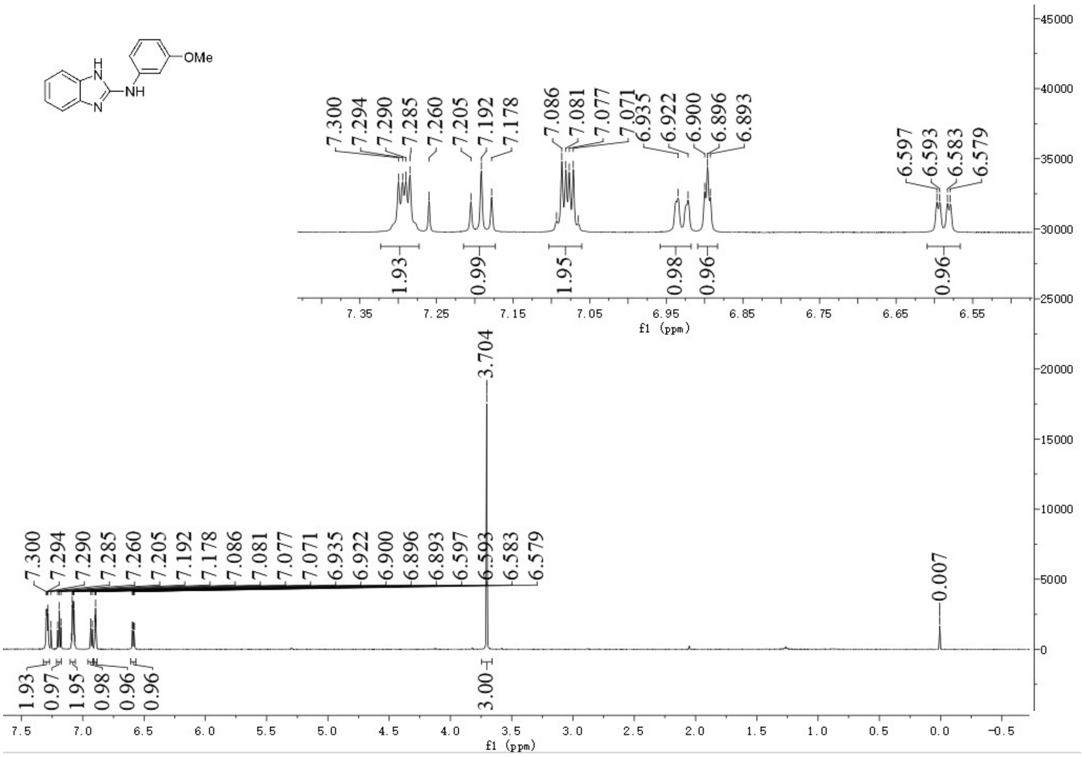

***N*-Phenyl-1,4-dihydroquinazolin-2-amine (56)**

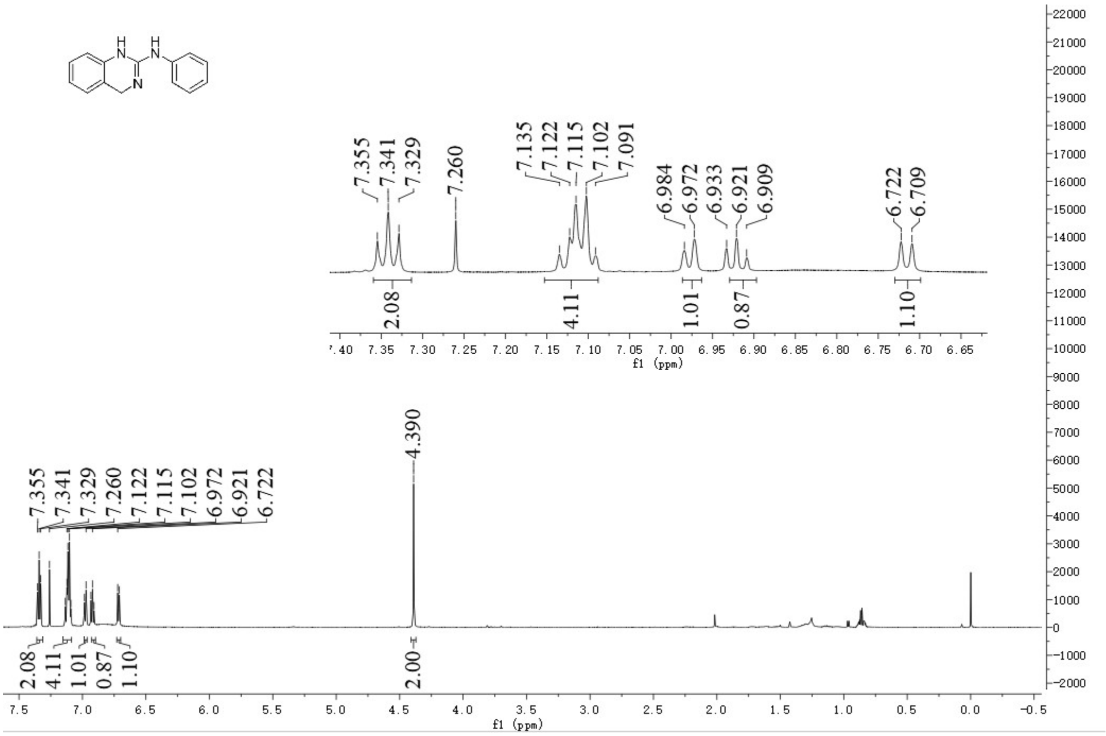

4-((1,4-Dihydroquinazolin-2-yl)amino)benzonitrile (57)

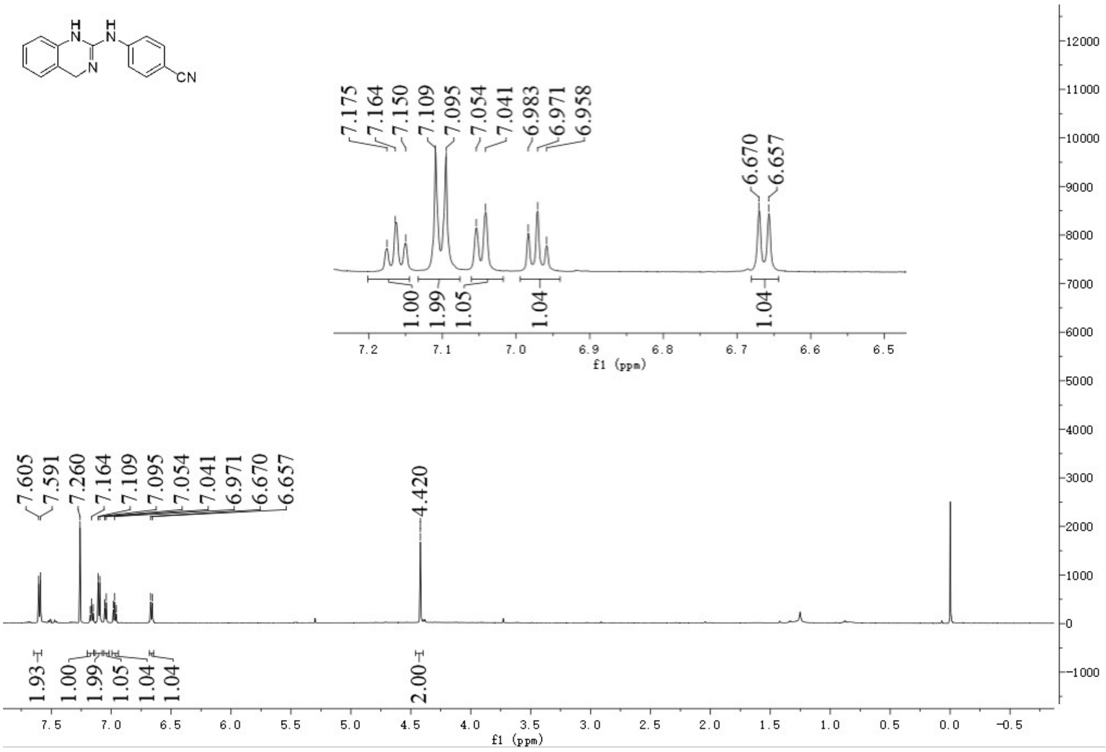

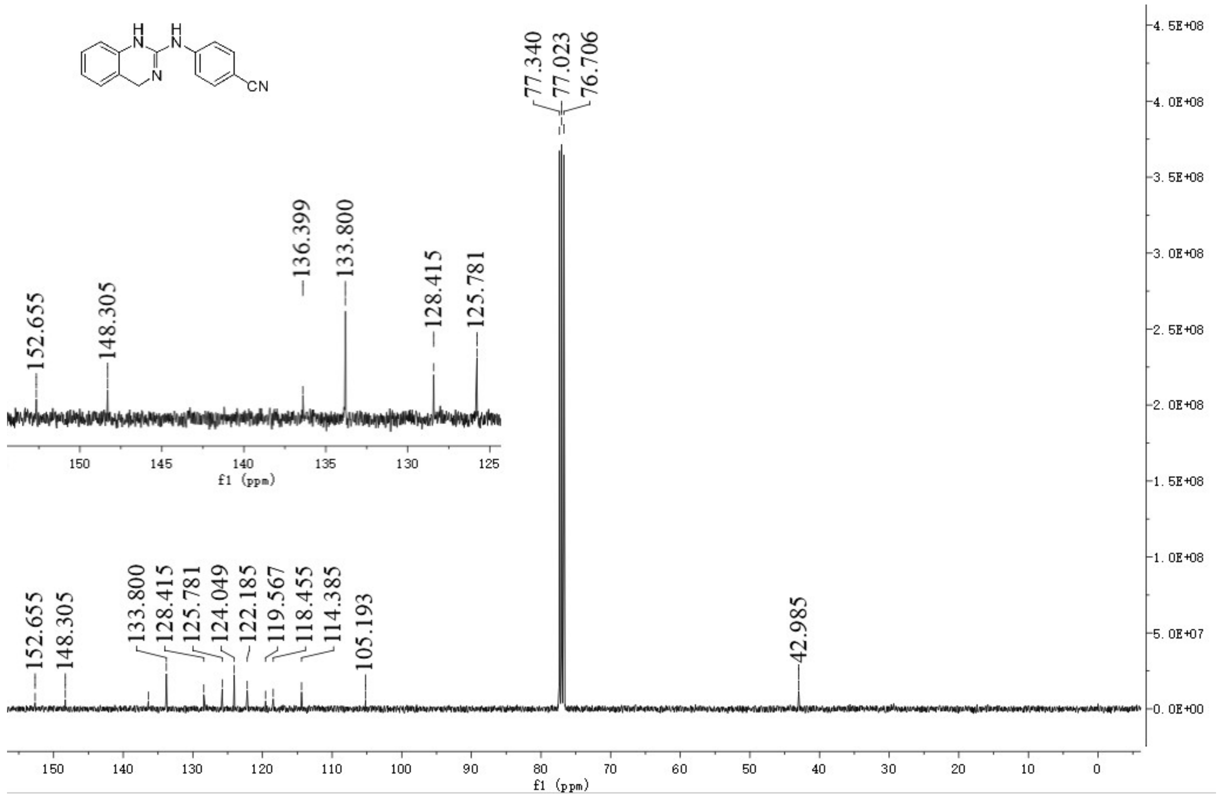

N-(3-Methoxyphenyl)-1,4-dihydroquinazolin-2-amine (58)

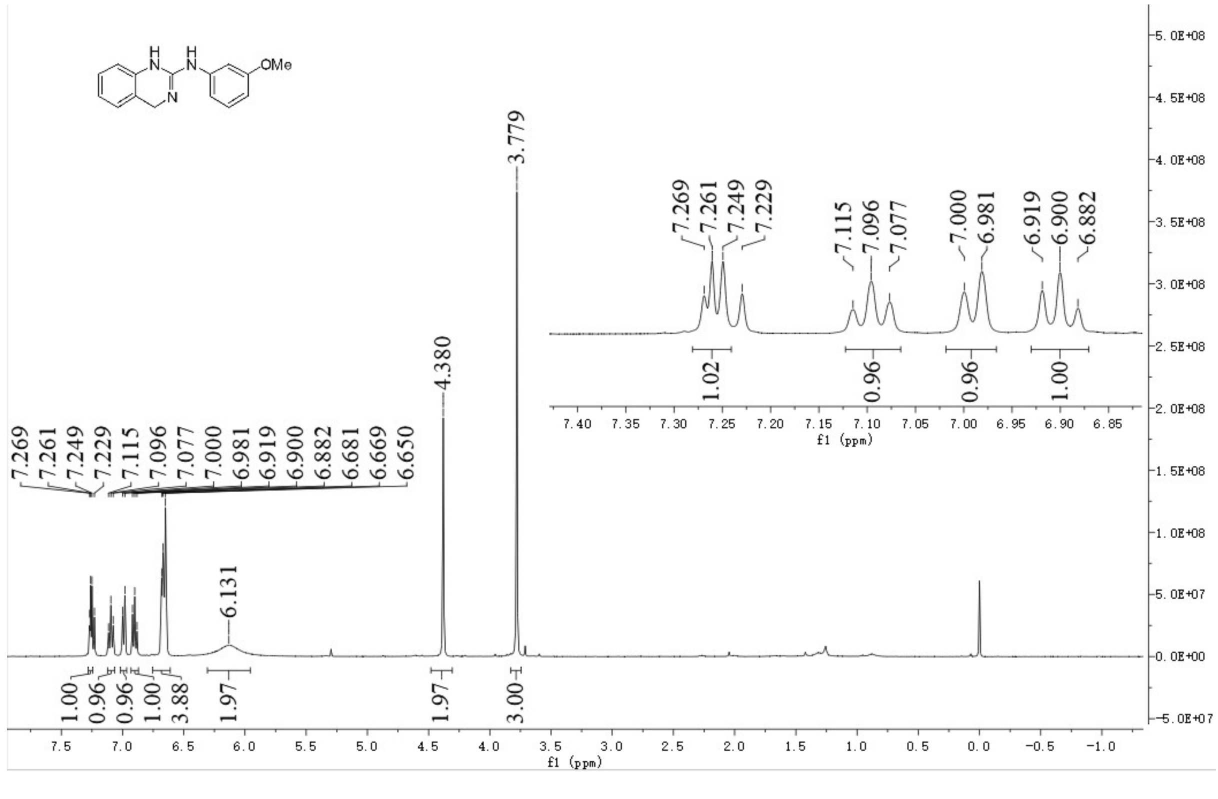

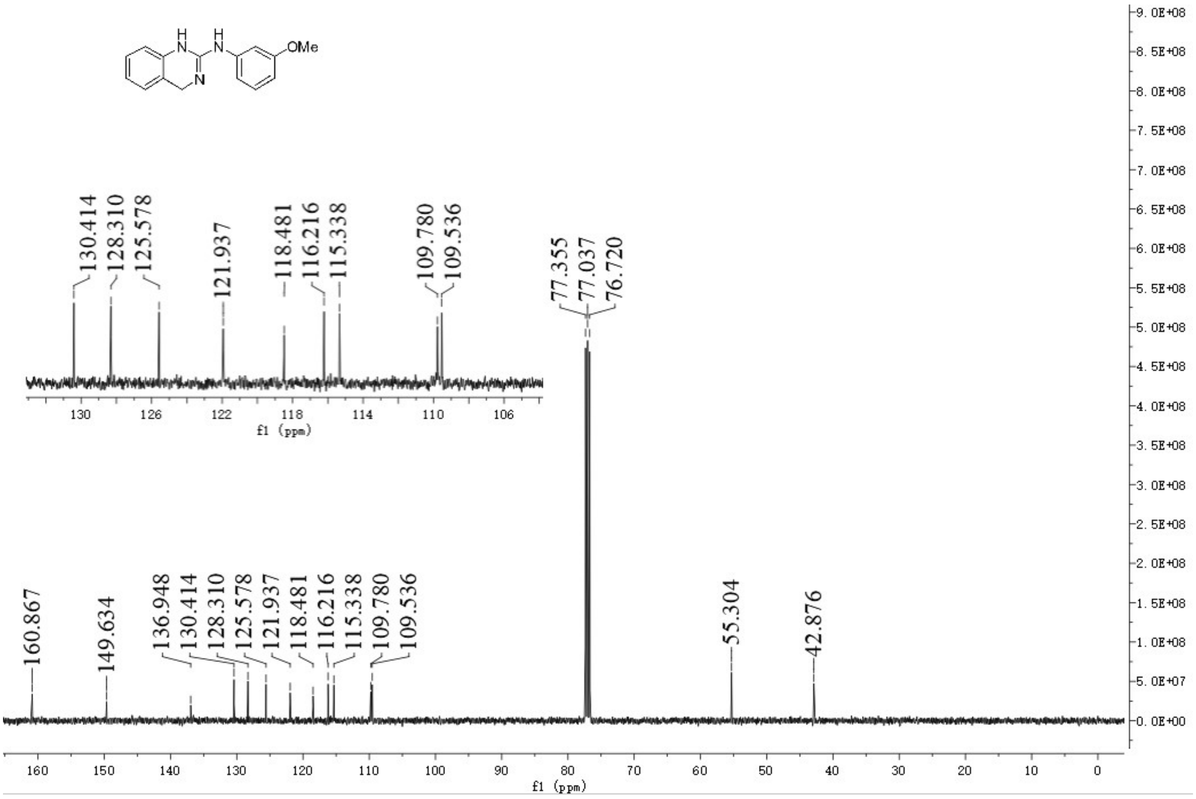

N-Phenylquinazolin-2-amine (59)

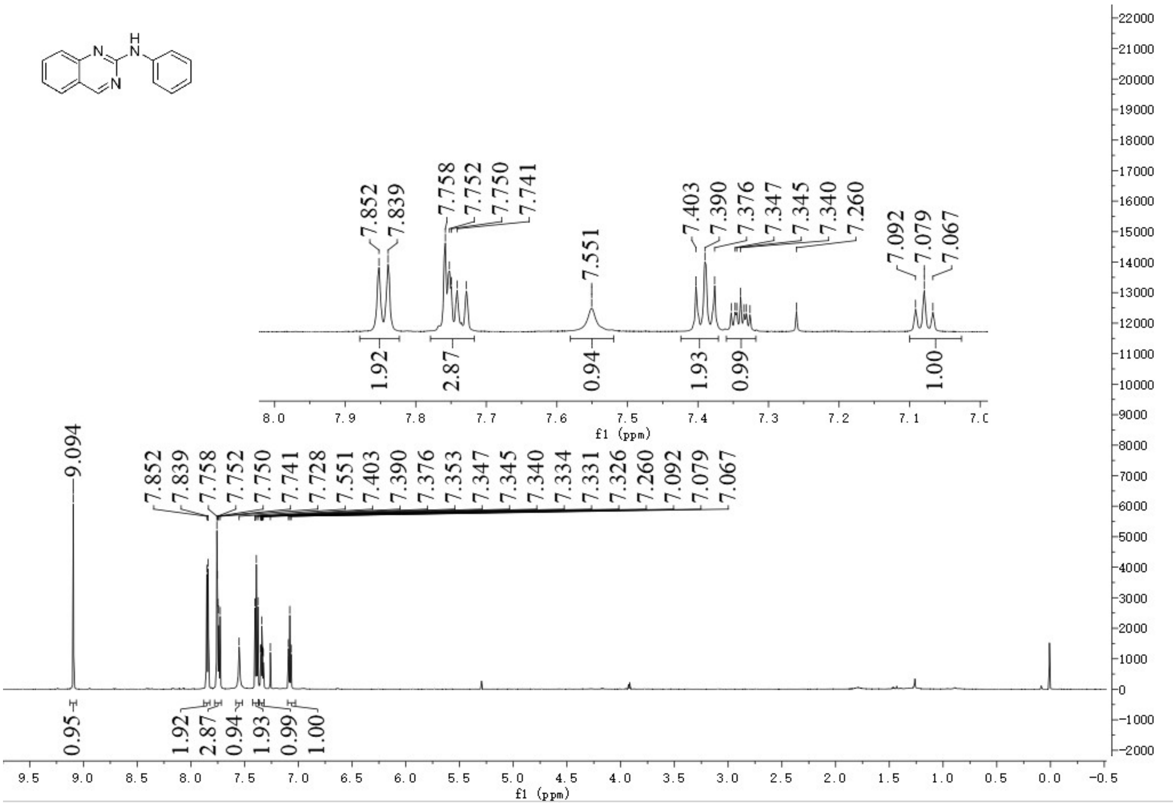

4-(Quinazolin-2-ylamino)benzonitrile (60)

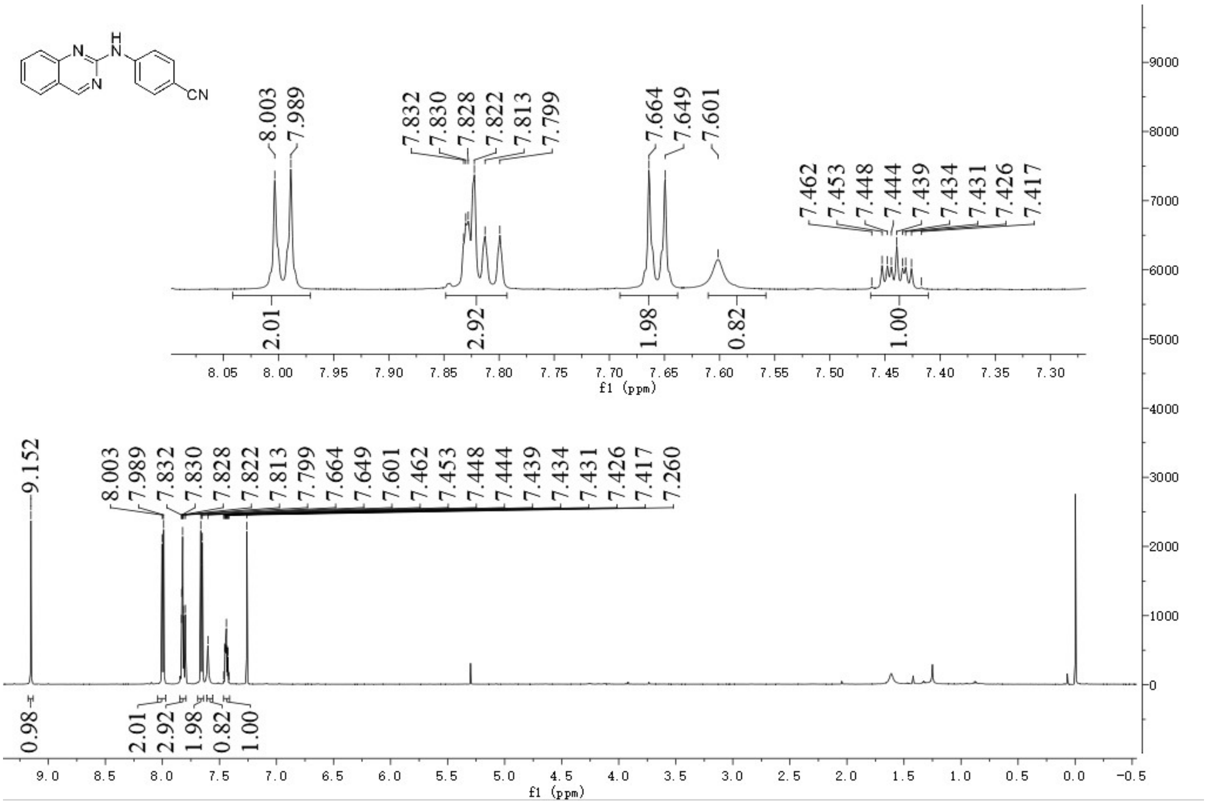

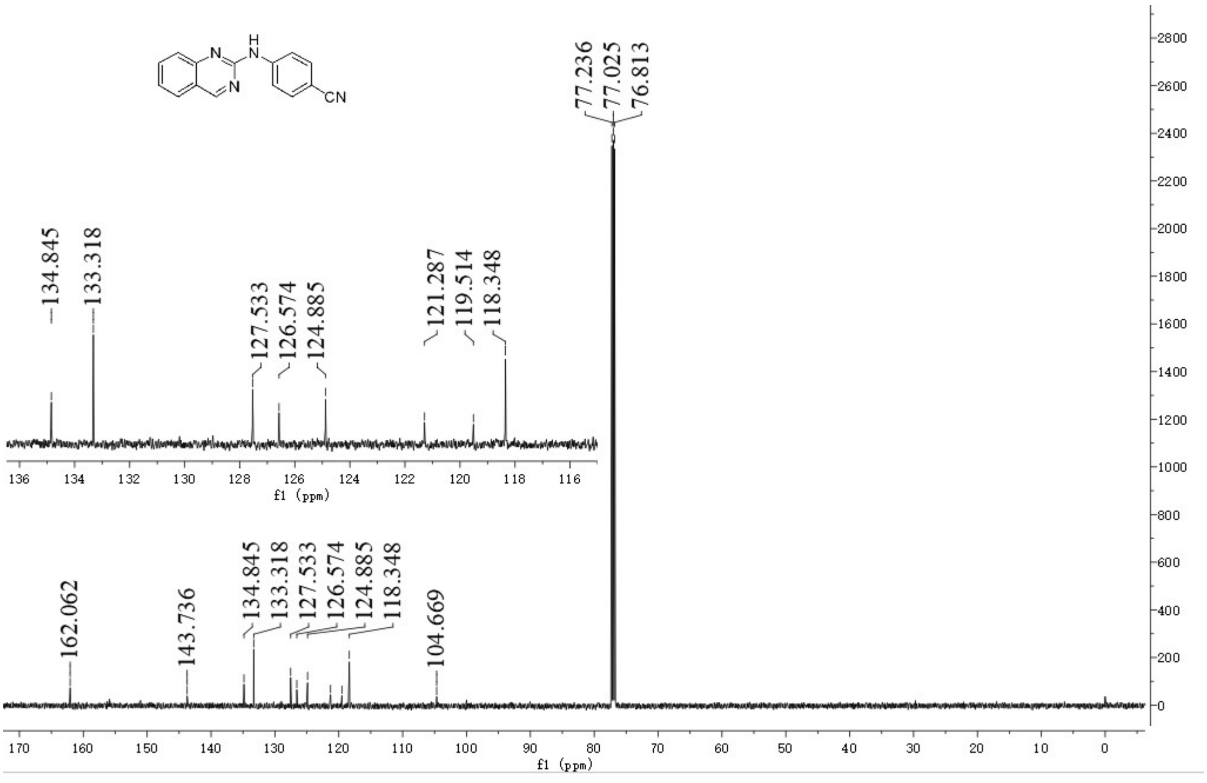

N-(3-Methoxyphenyl)quinazolin-2-amine (61)

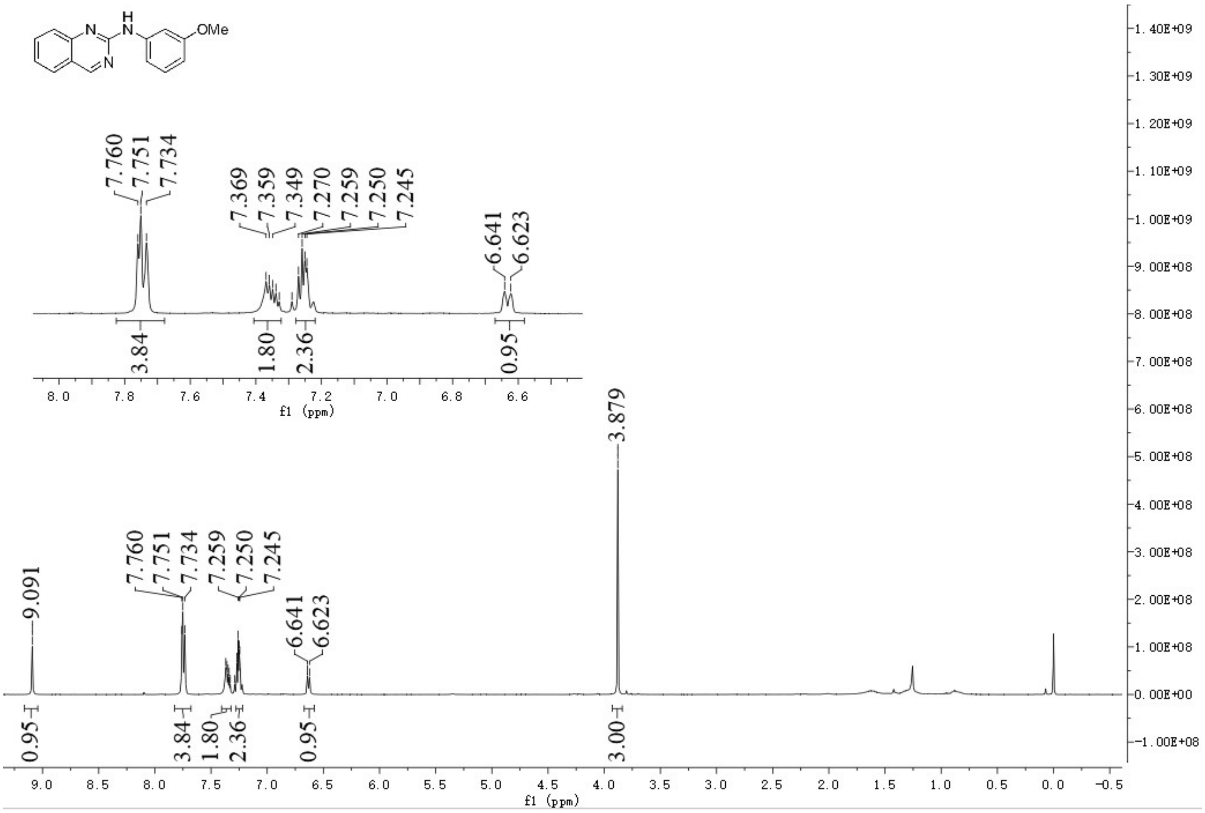

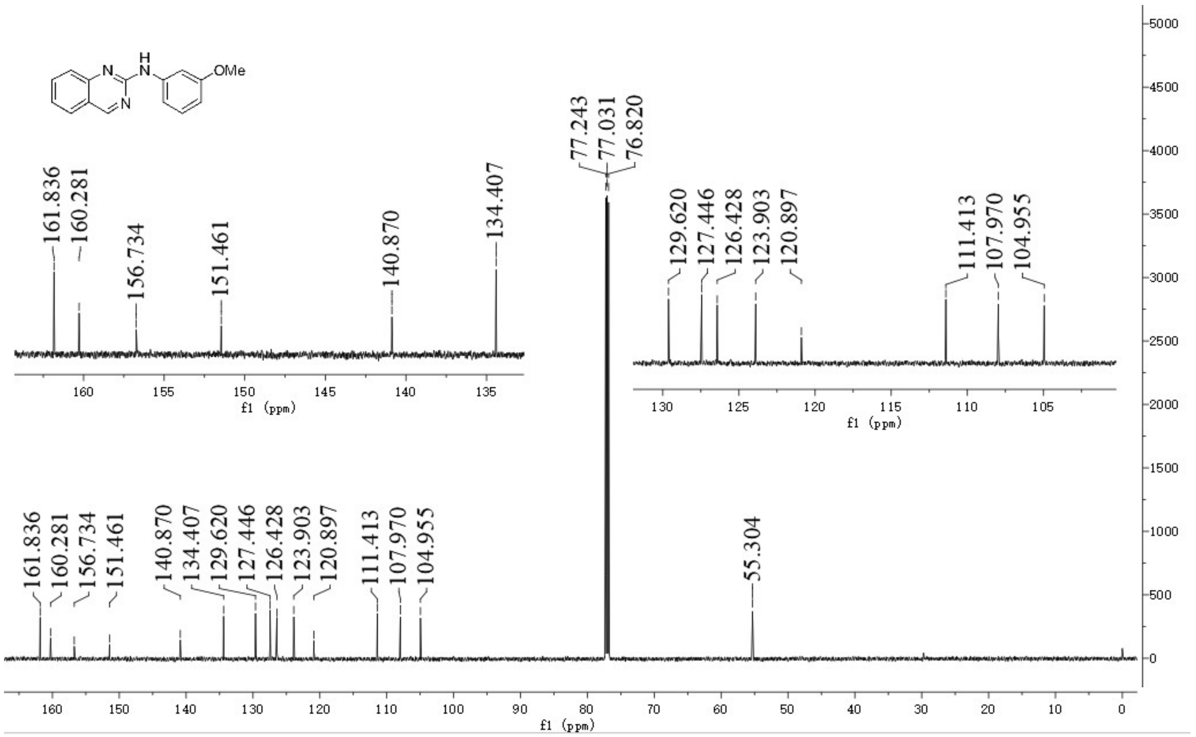

5-Methyl-*N*-1-diphenyl-1*H*-imidazol-2-amine (62)

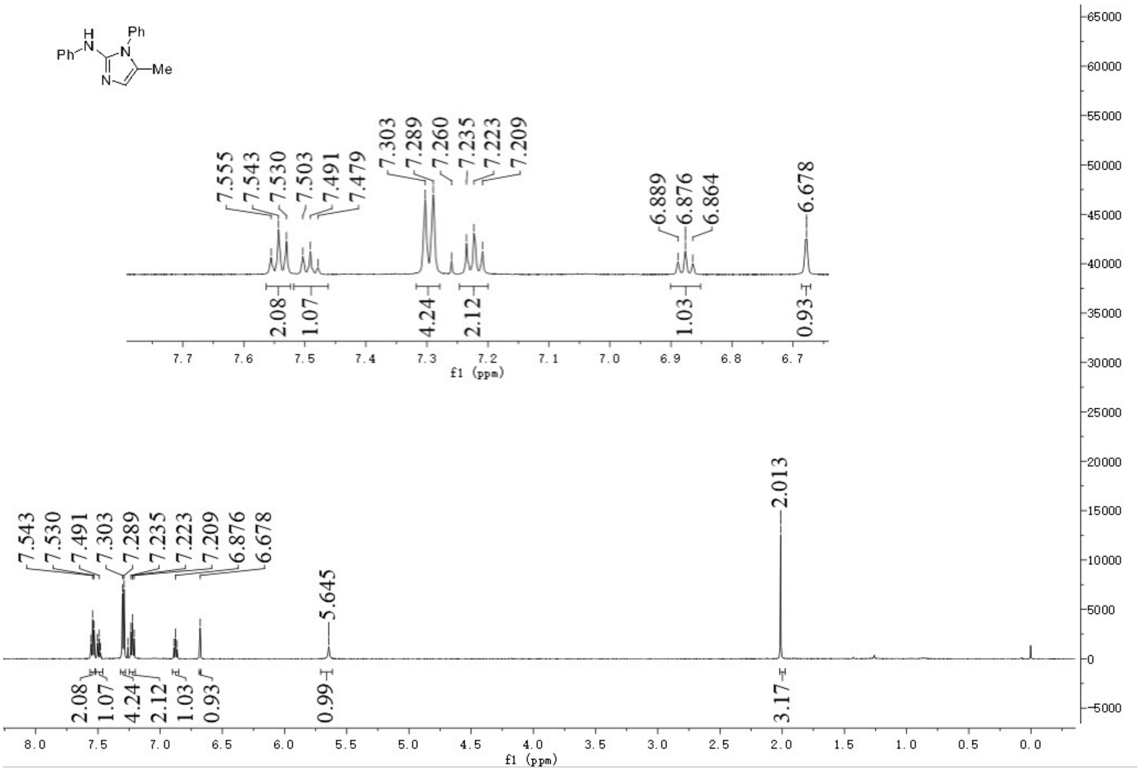

4,5-Dimethyl-*N*-1-diphenyl-1*H*-imidazol-2-amine (63)

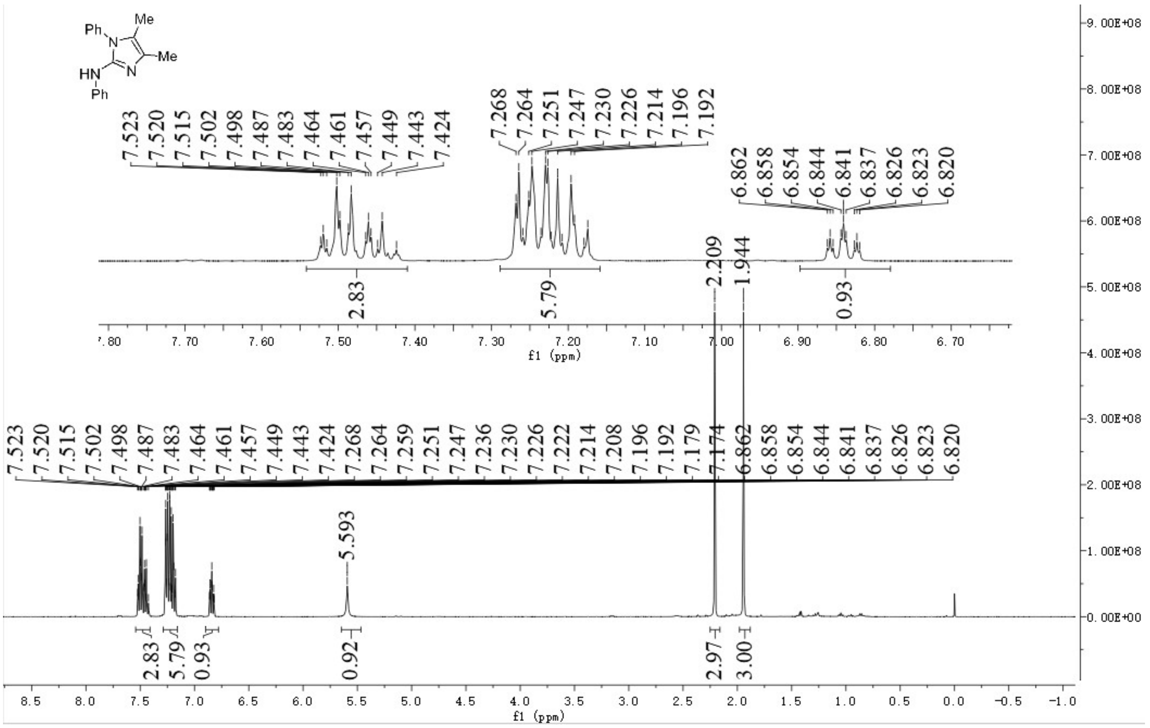

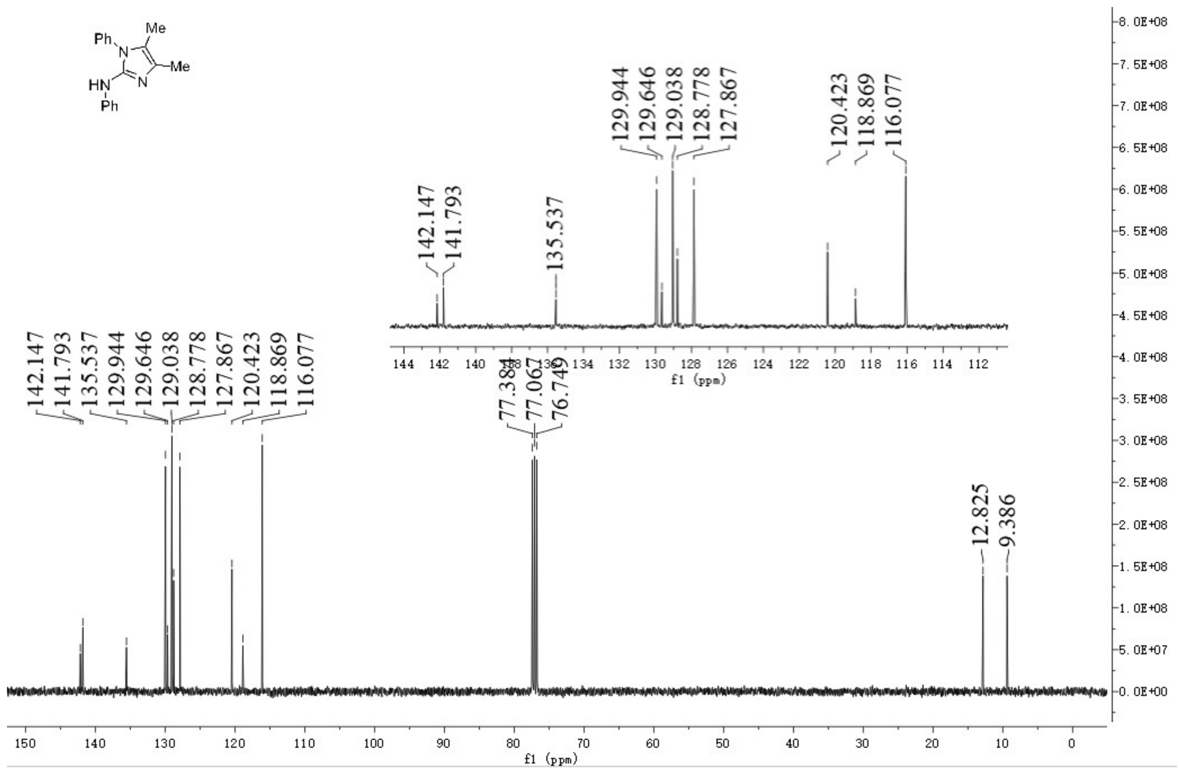

1,4-Dimethyl-*N*-3-diphenyl-1,3-dihydro-2*H*-imidazol-2-imine (64)

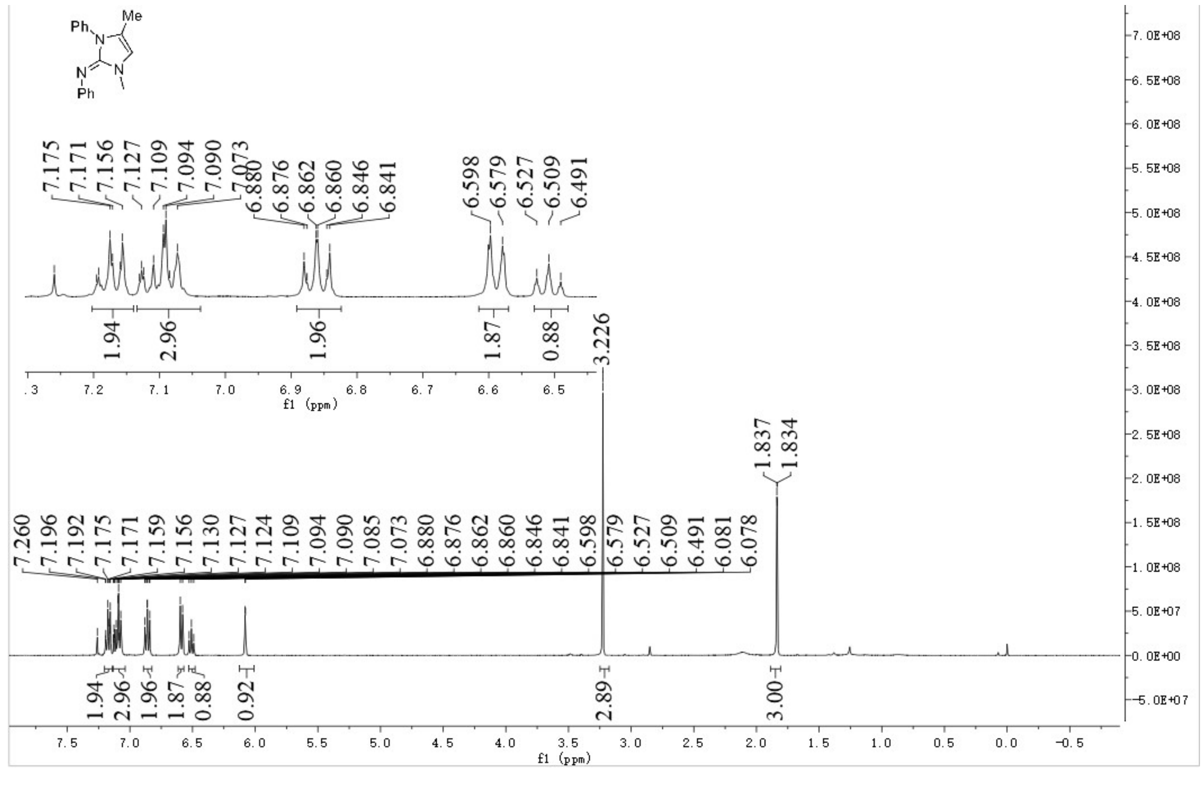

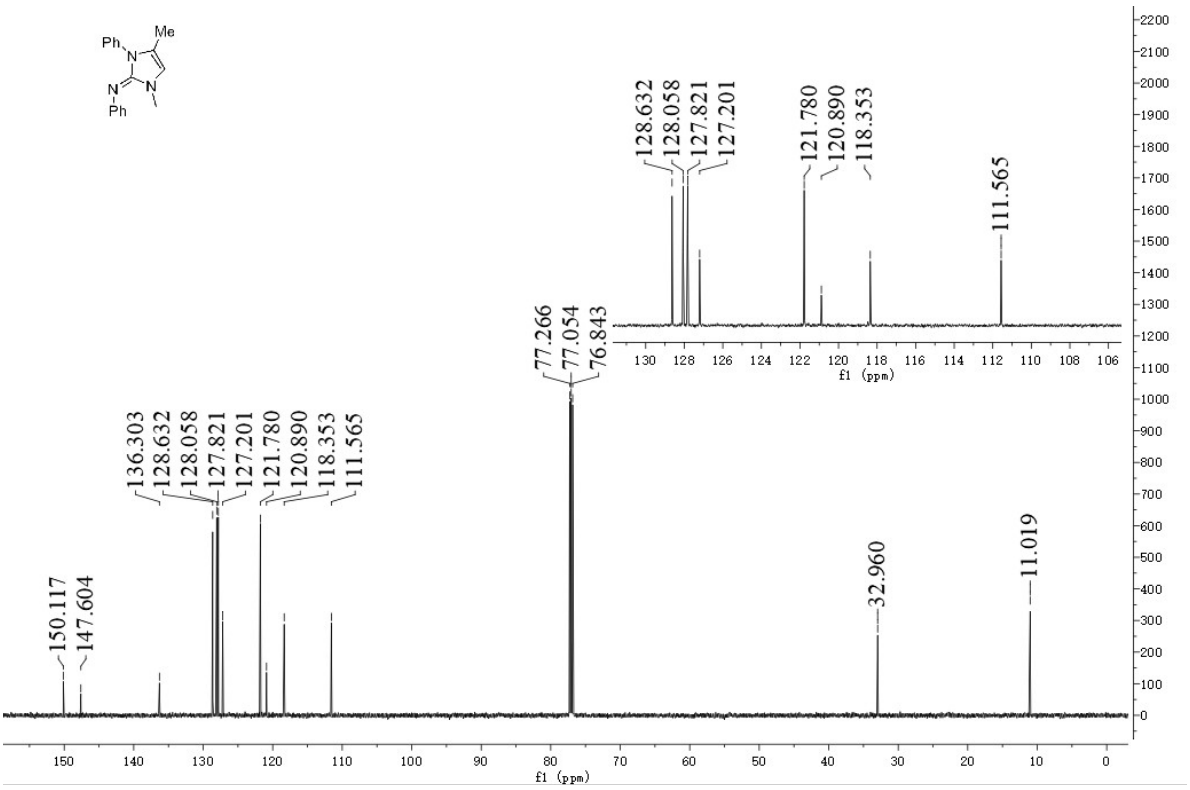

1-Benzyl-4-methyl-*N*,3-diphenyl-1,3-dihydro-2*H*-imidazol-2-imine (65)

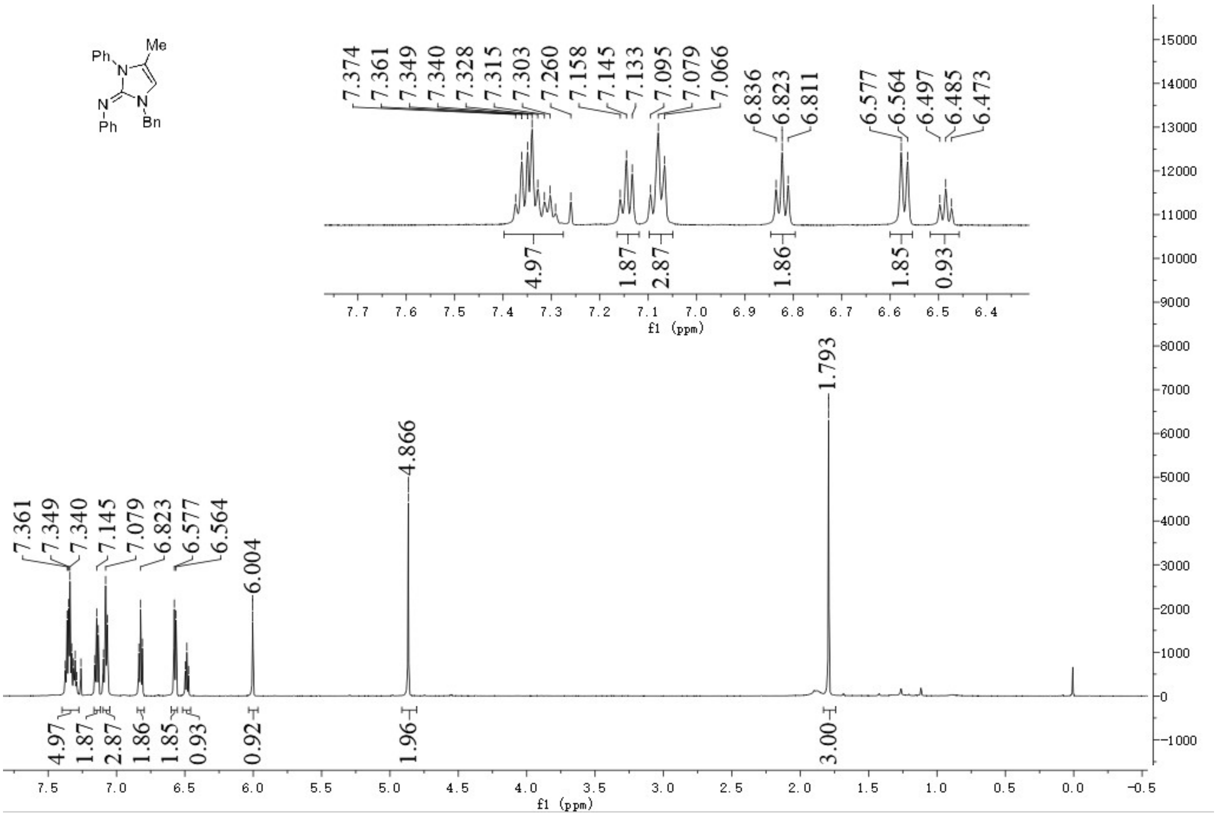

3-Phenyl-2-(phenylimino)imidazolidin-4-one (66)

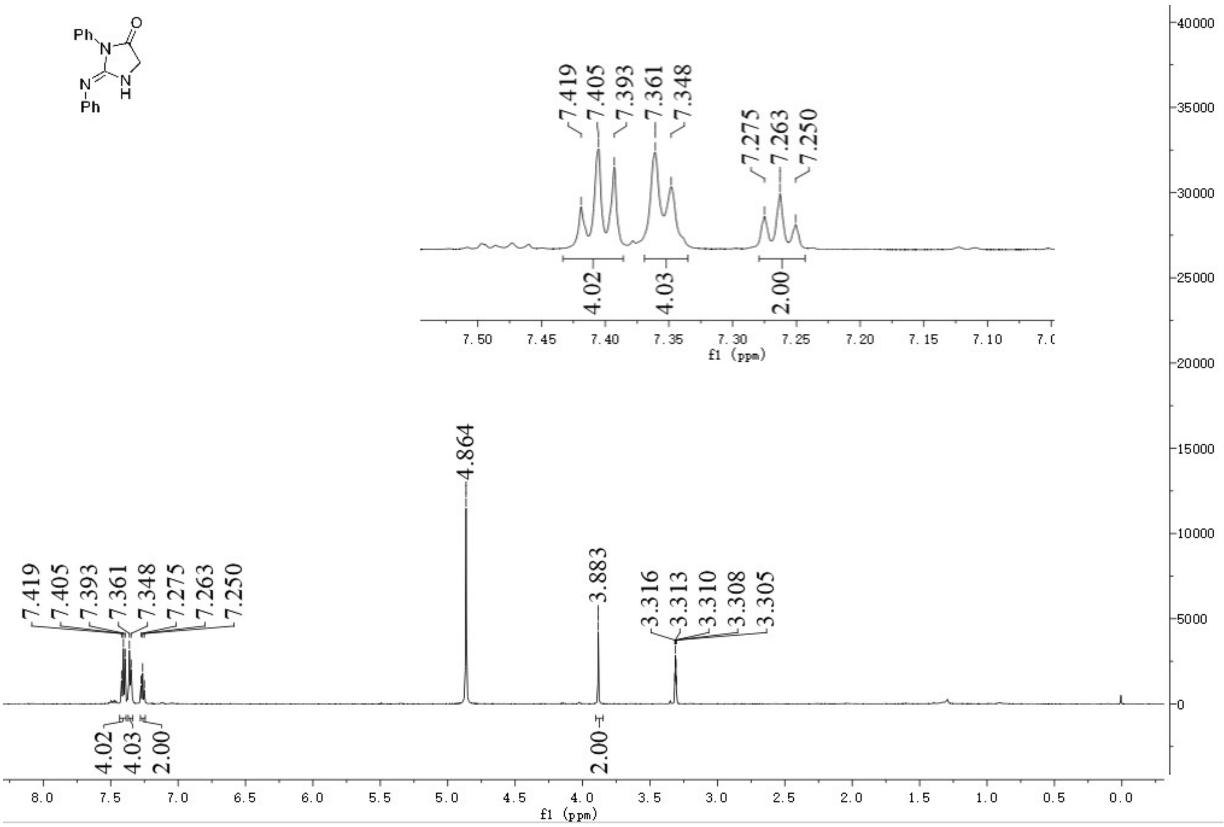

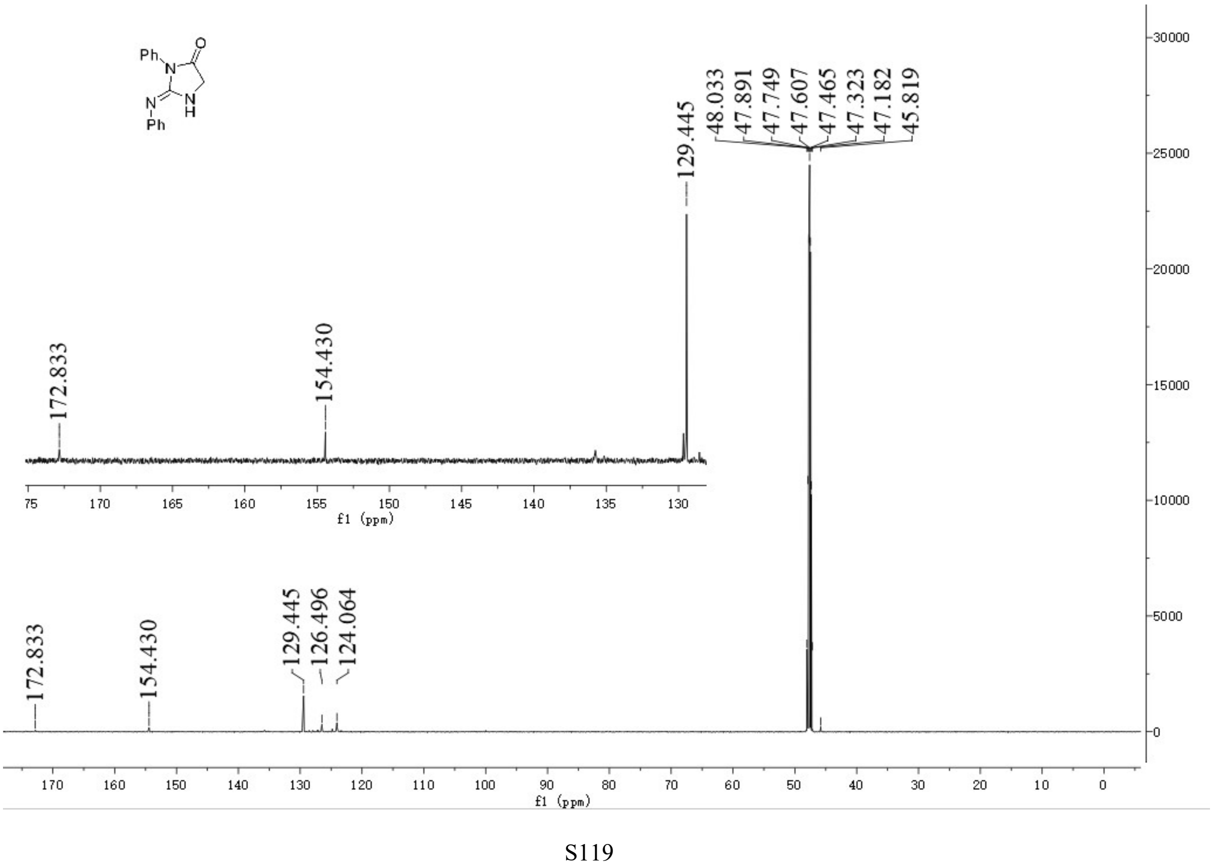

5-Methyl-3-phenyl-2-(phenylimino)imidazolidin-4-one (67)

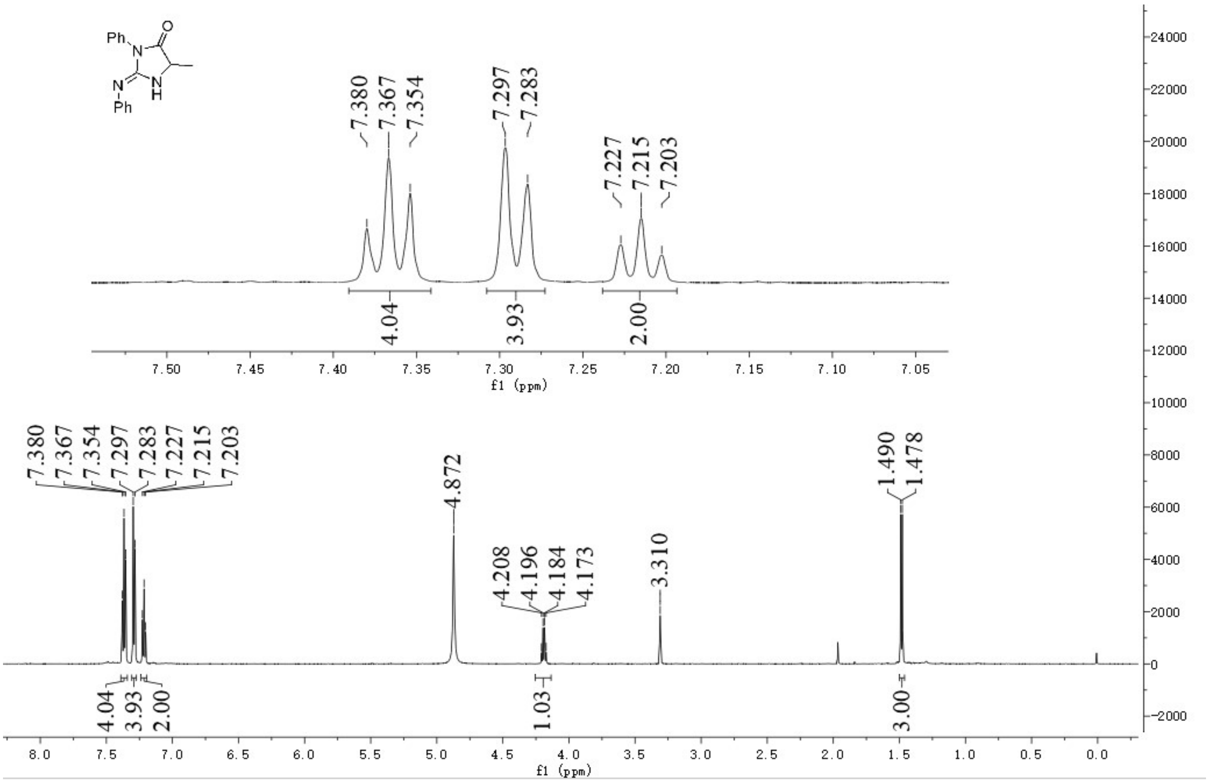

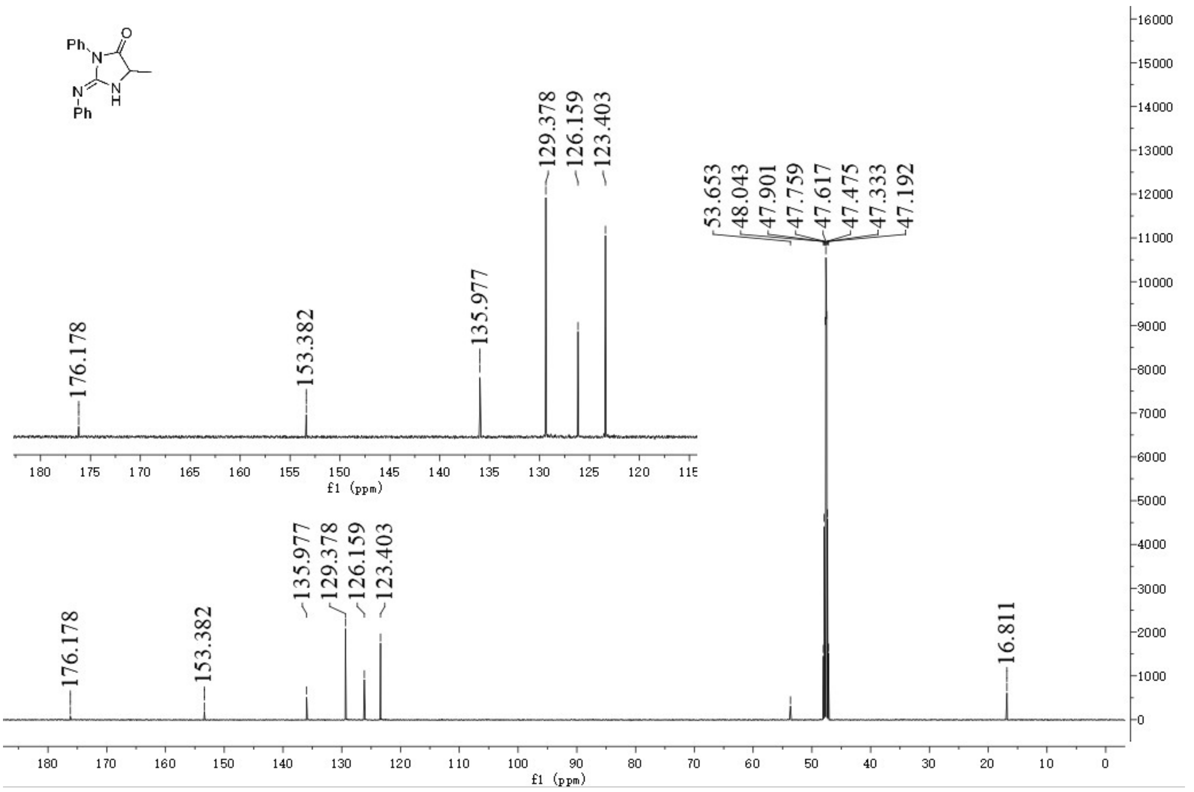

5-Benzyl-3-phenyl-2-(phenylimino)imidazolidin-4-one (68)

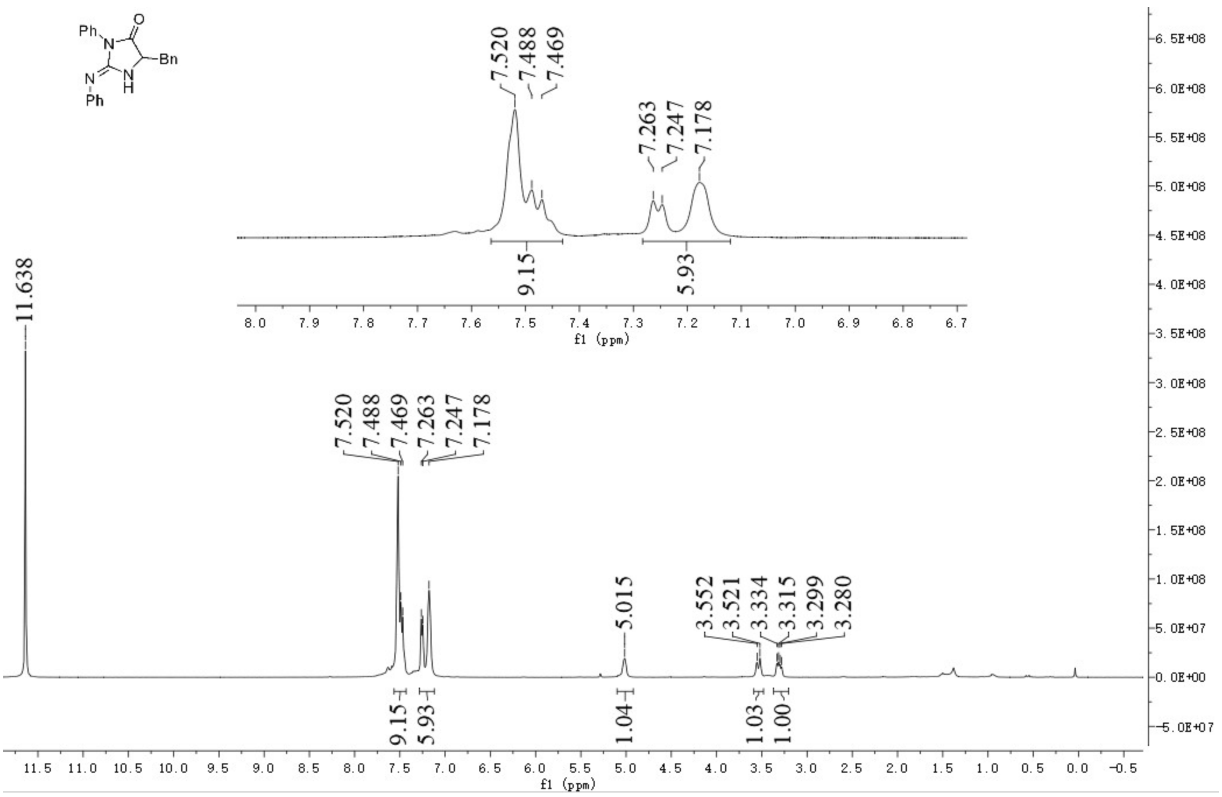

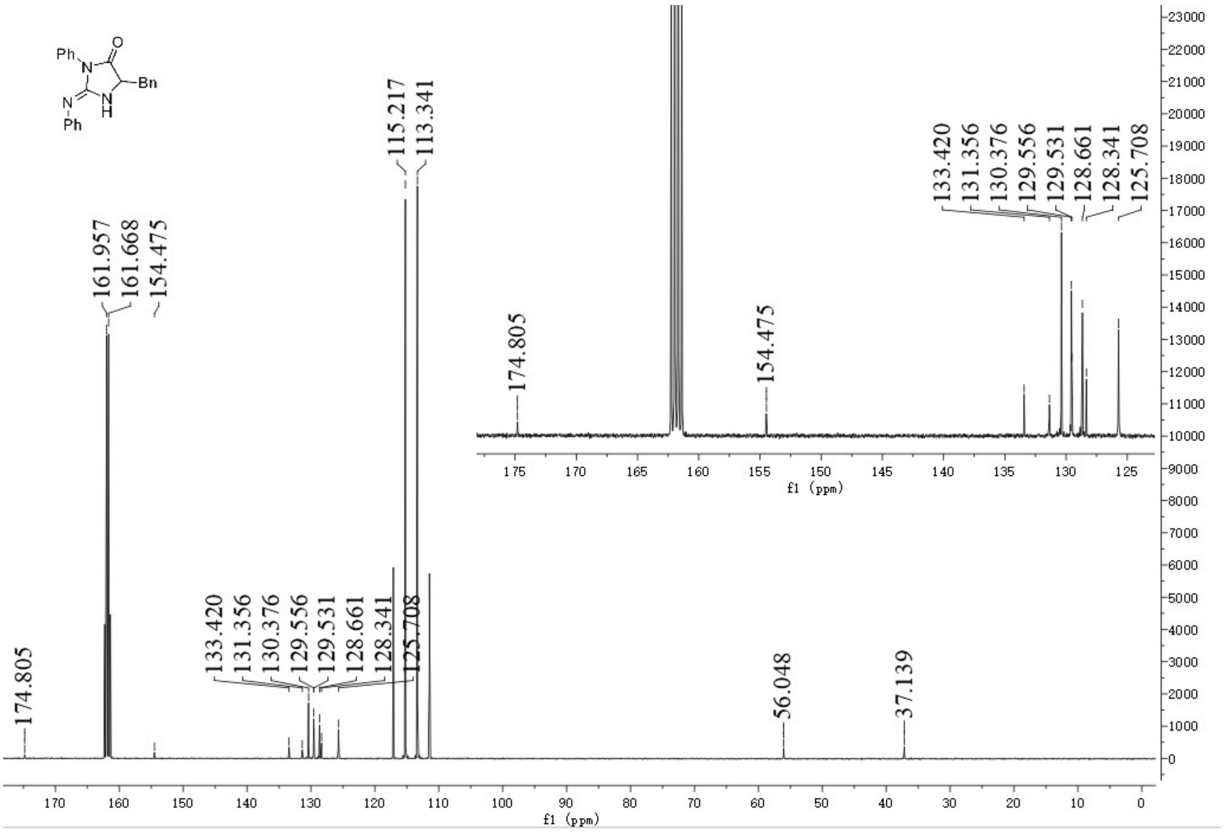

5-Isopropyl-3-phenyl-2-(phenylimino)imidazolidin-4-one (69)

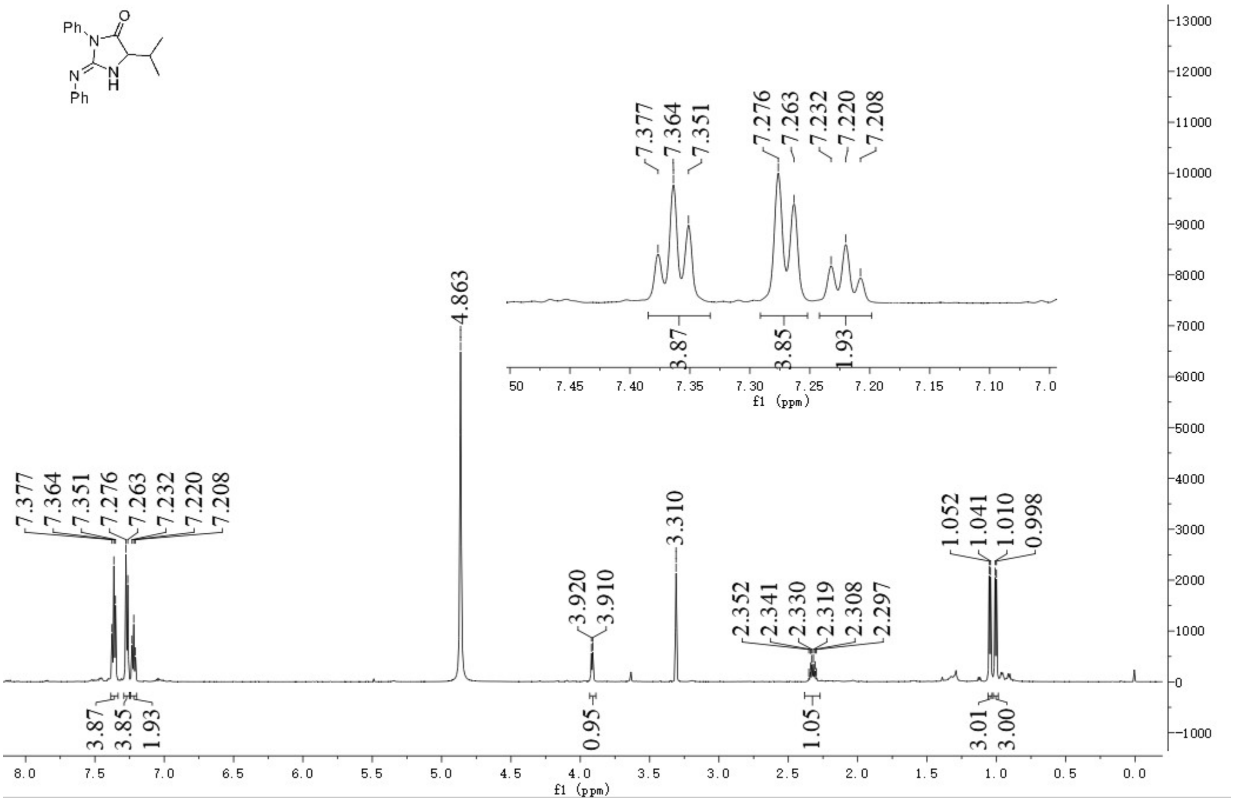

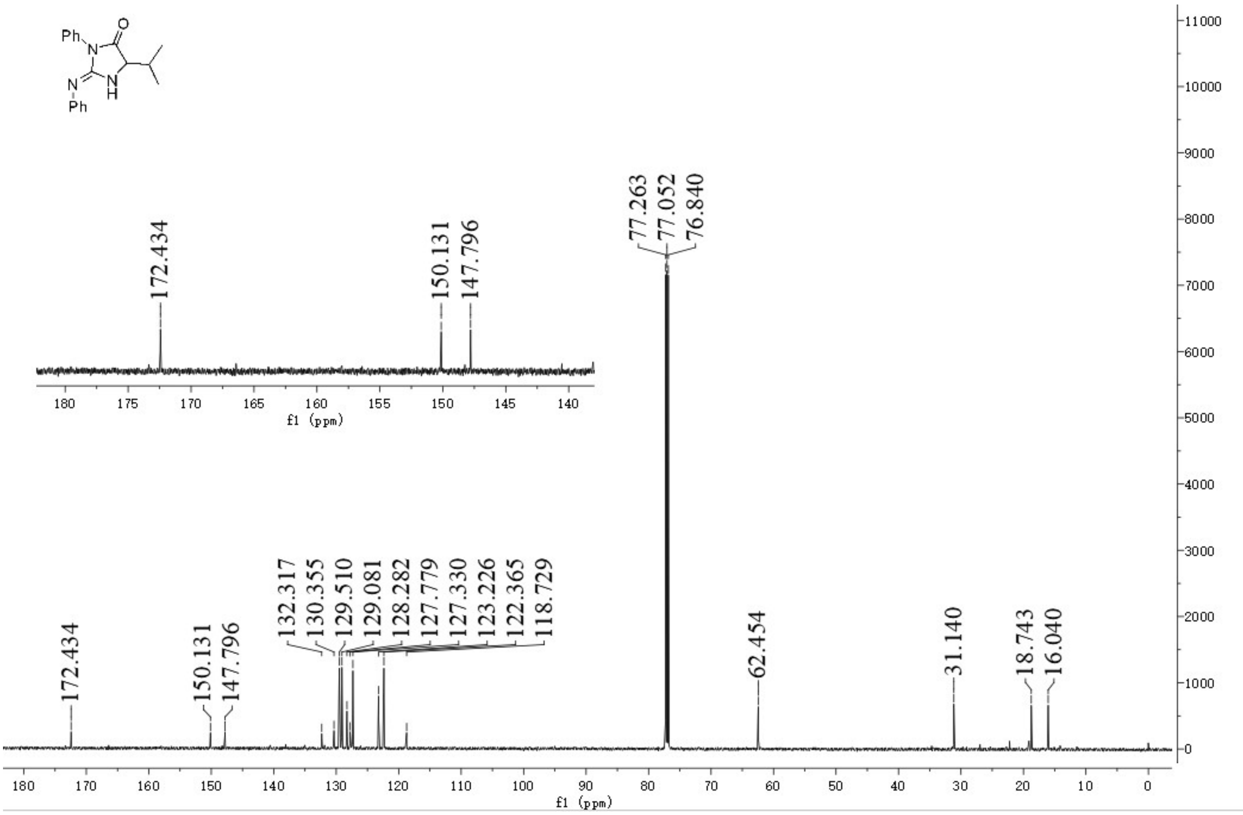

3-Phenyl-2-(phenylimino)-5-propylimidazolidin-4-one (70)

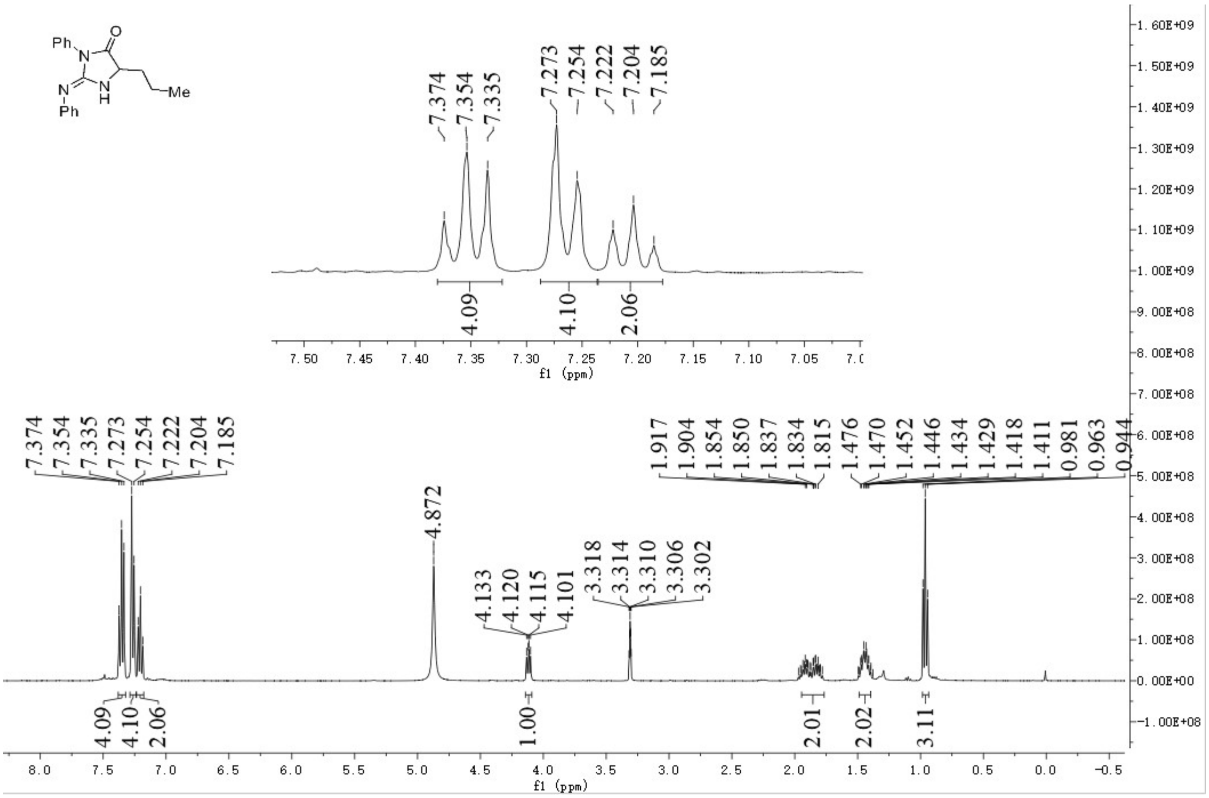

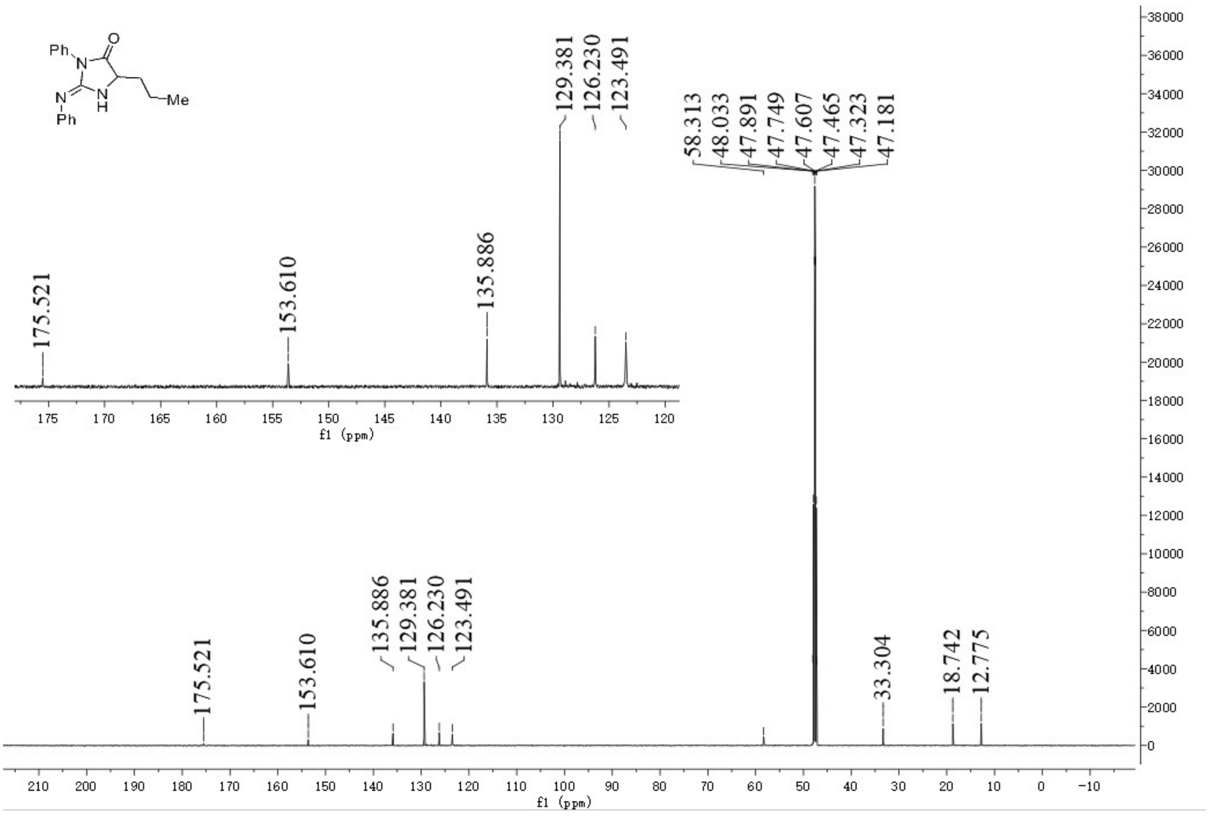

5-Isobutyl-3-phenyl-2-(phenylimino)imidazolidin-4-one (71)

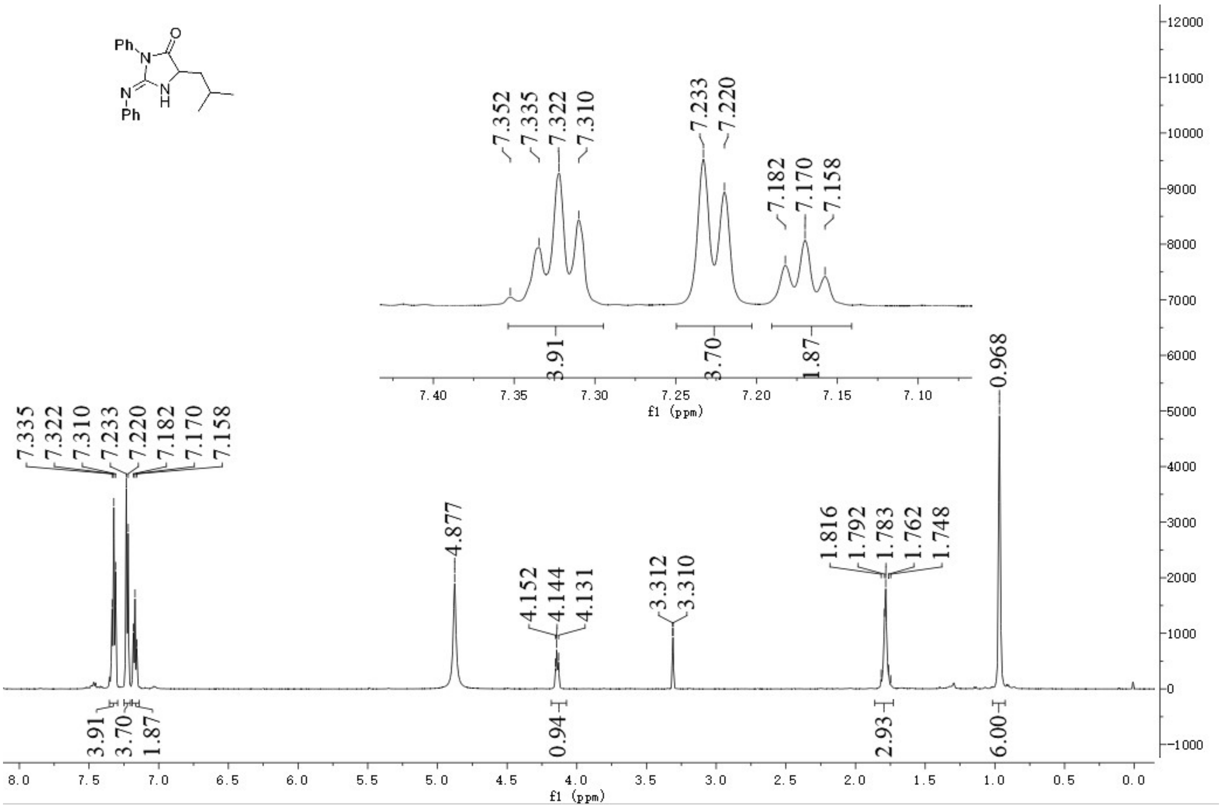

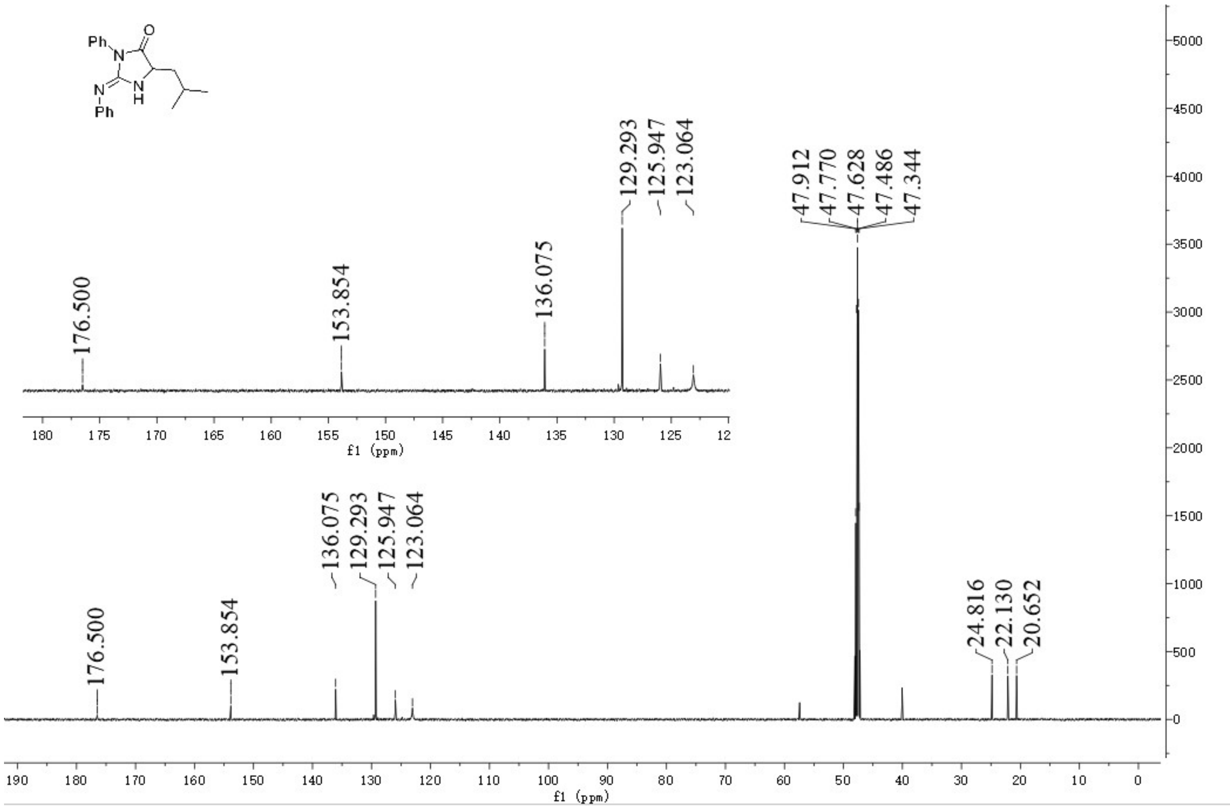

1-Methyl-3-phenyl-2-(phenylimino)imidazolidin-4-one (72)

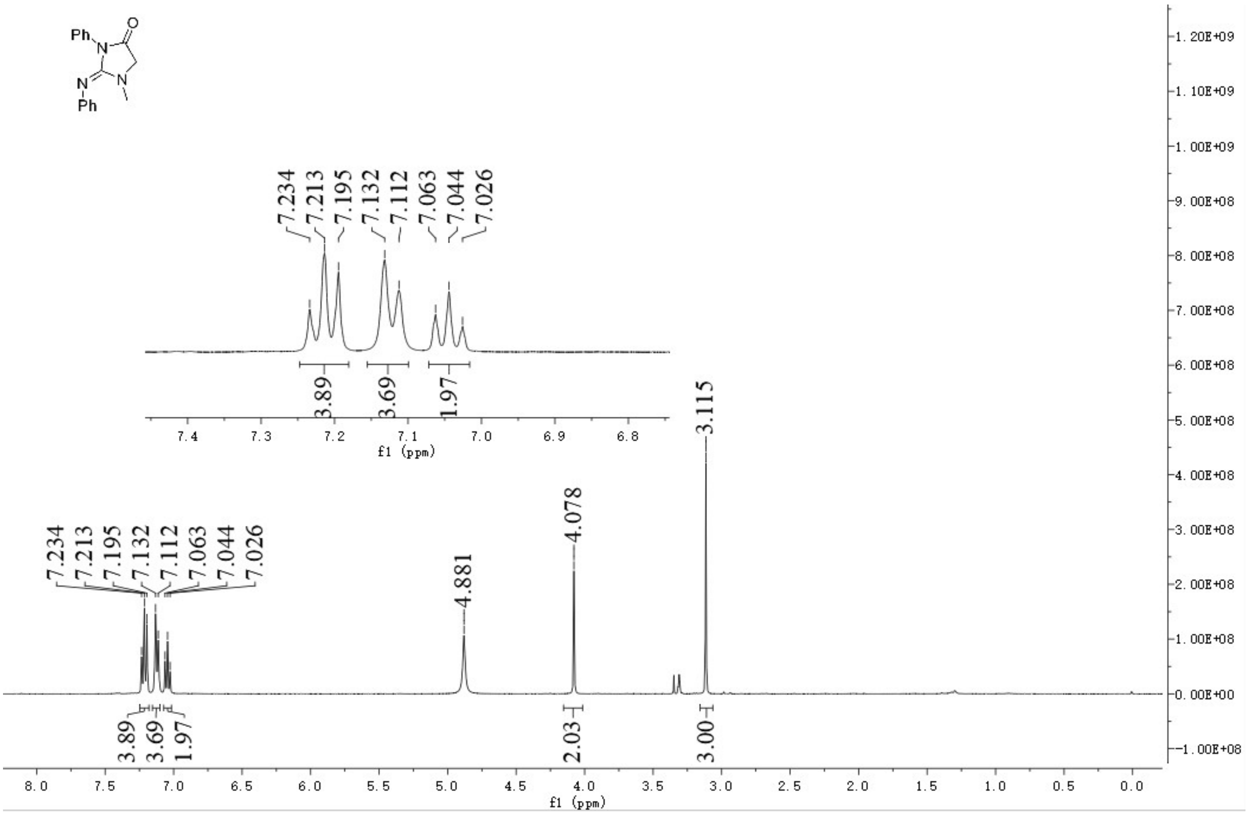

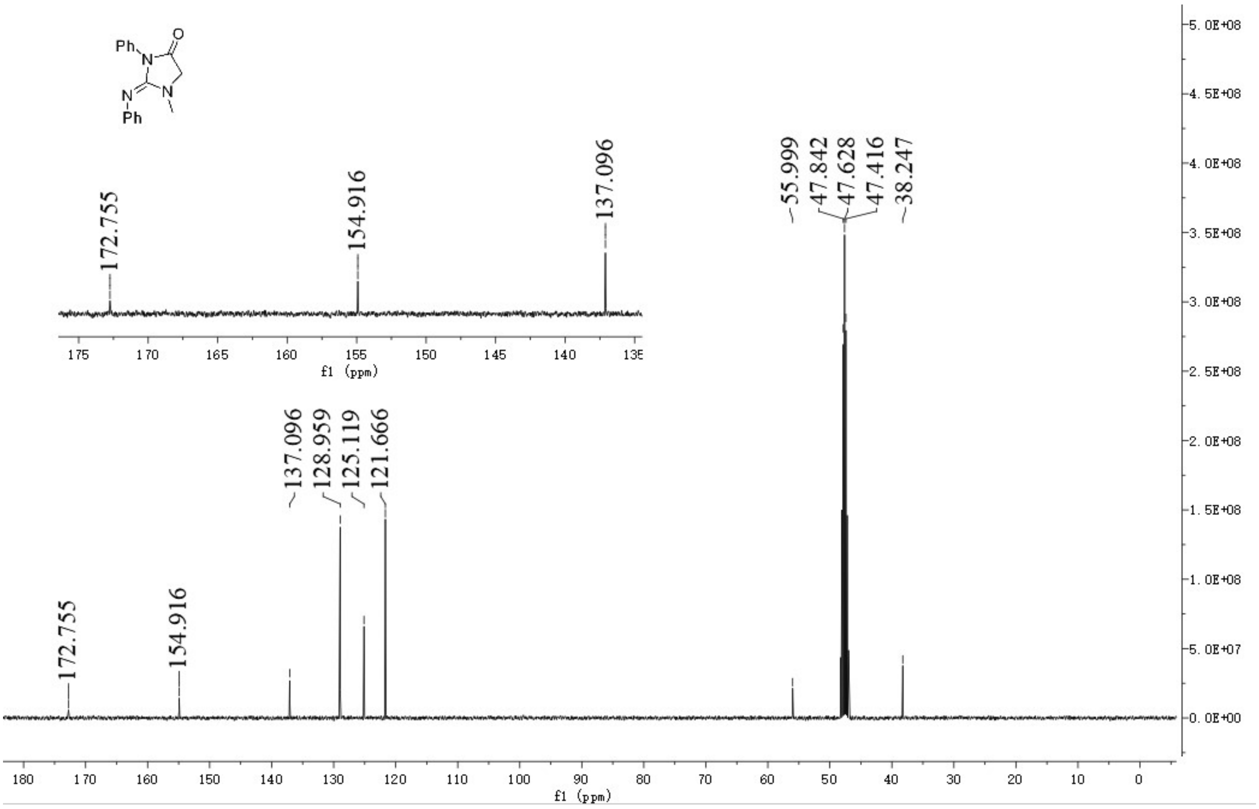

2-Phenyl-3-(phenylimino)hexahydro-1*H*-pyrrolo[1,2-*c*]imidazol-1-one (73)

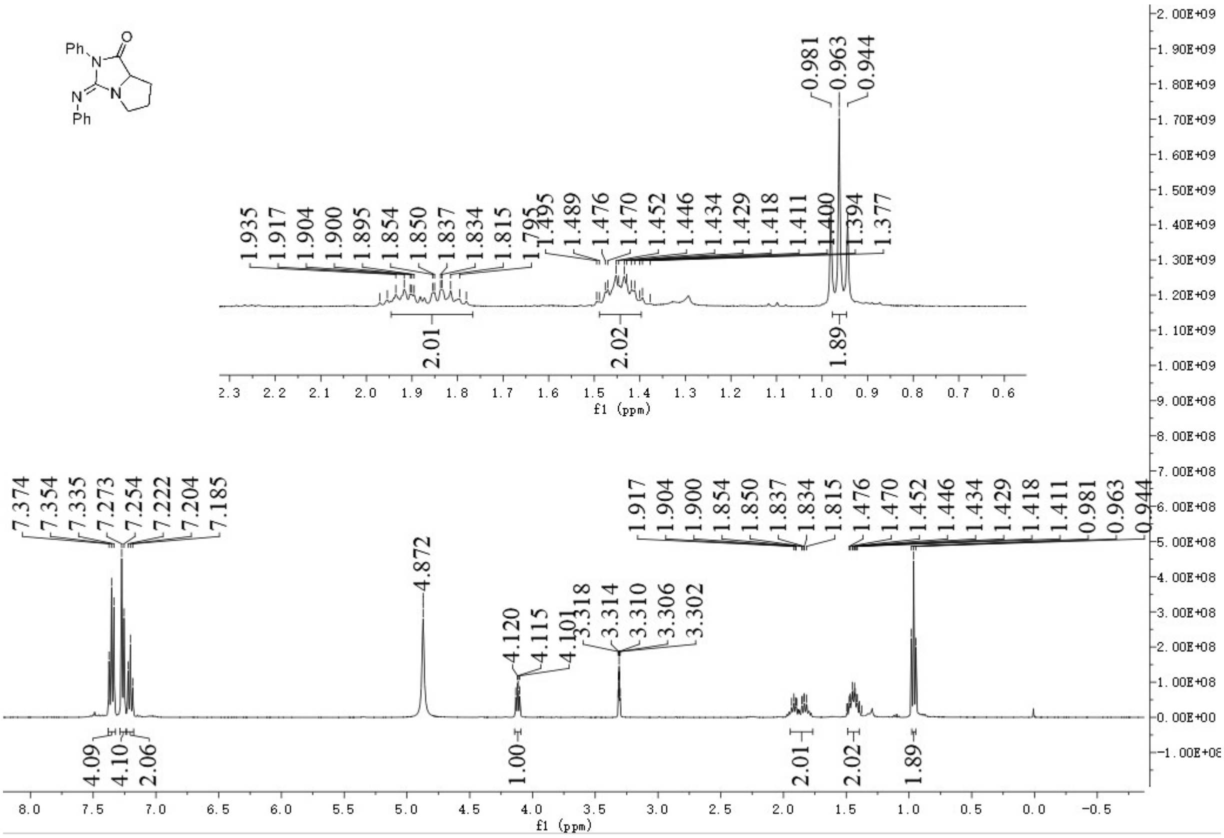

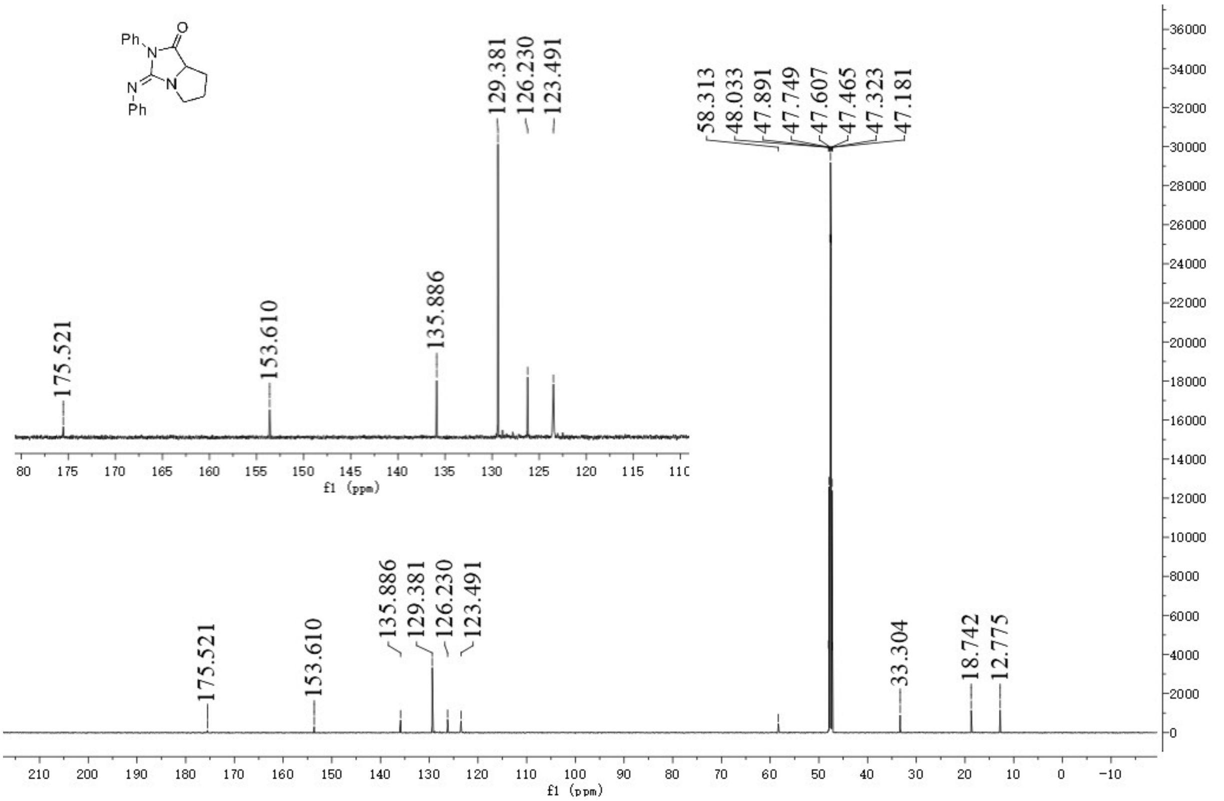

2-(2-(2,3-Diphenylguanidino)acetamido)-3-methylpentanoic acid (74)

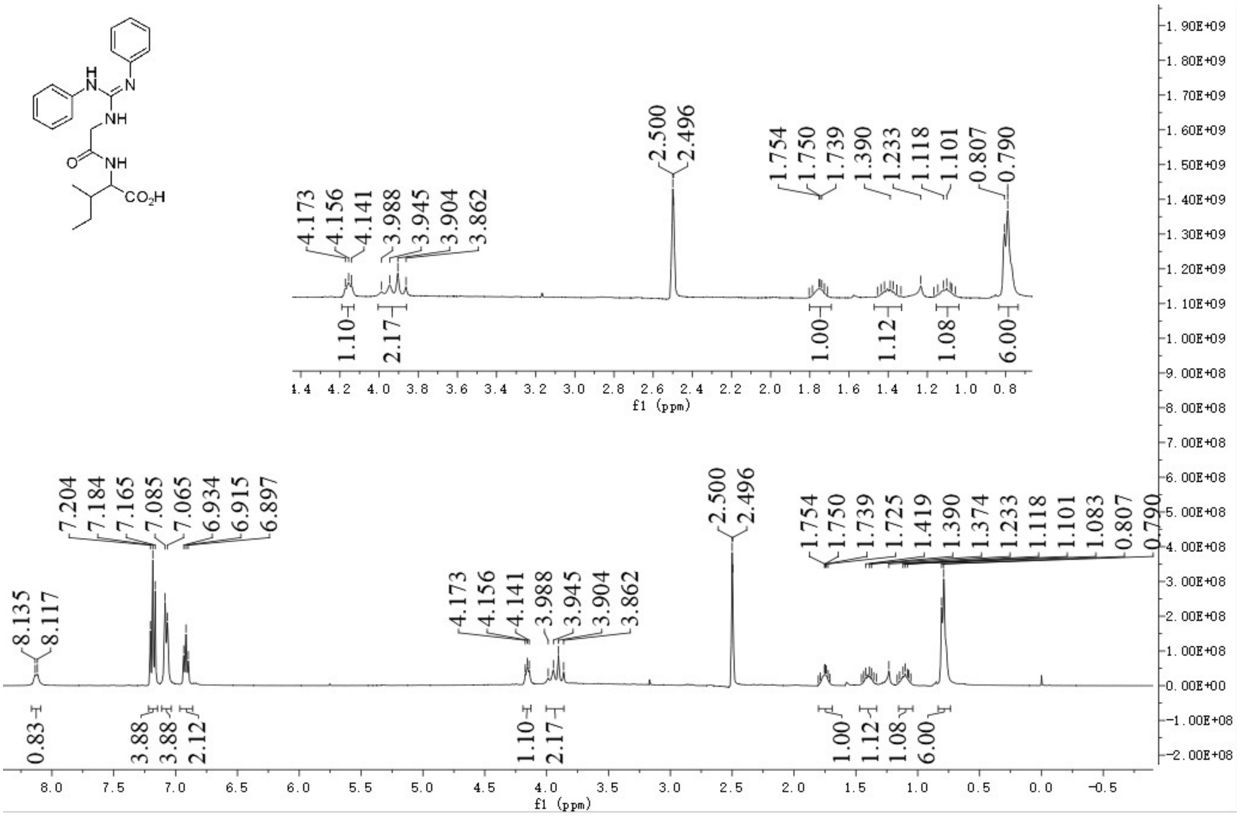

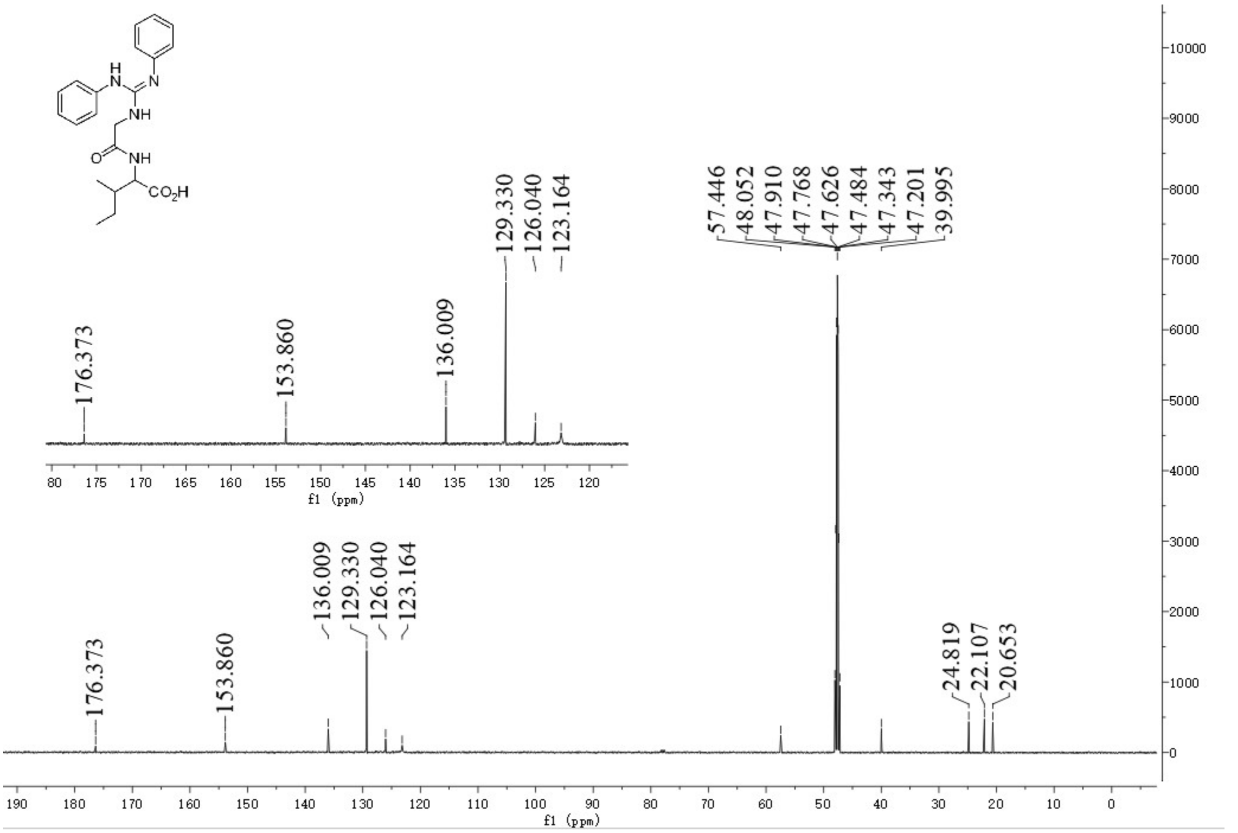

N,N'-Diphenylcarbamimidoyl)alanylalanine (75)

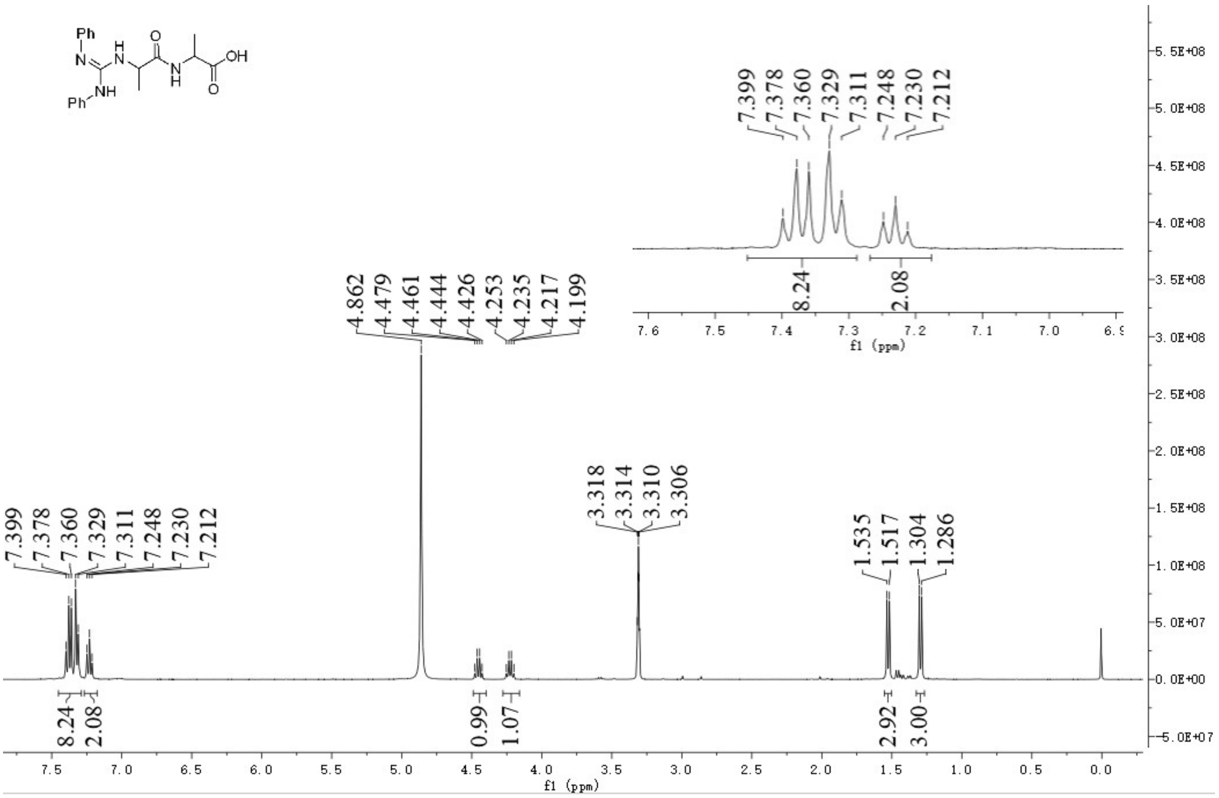

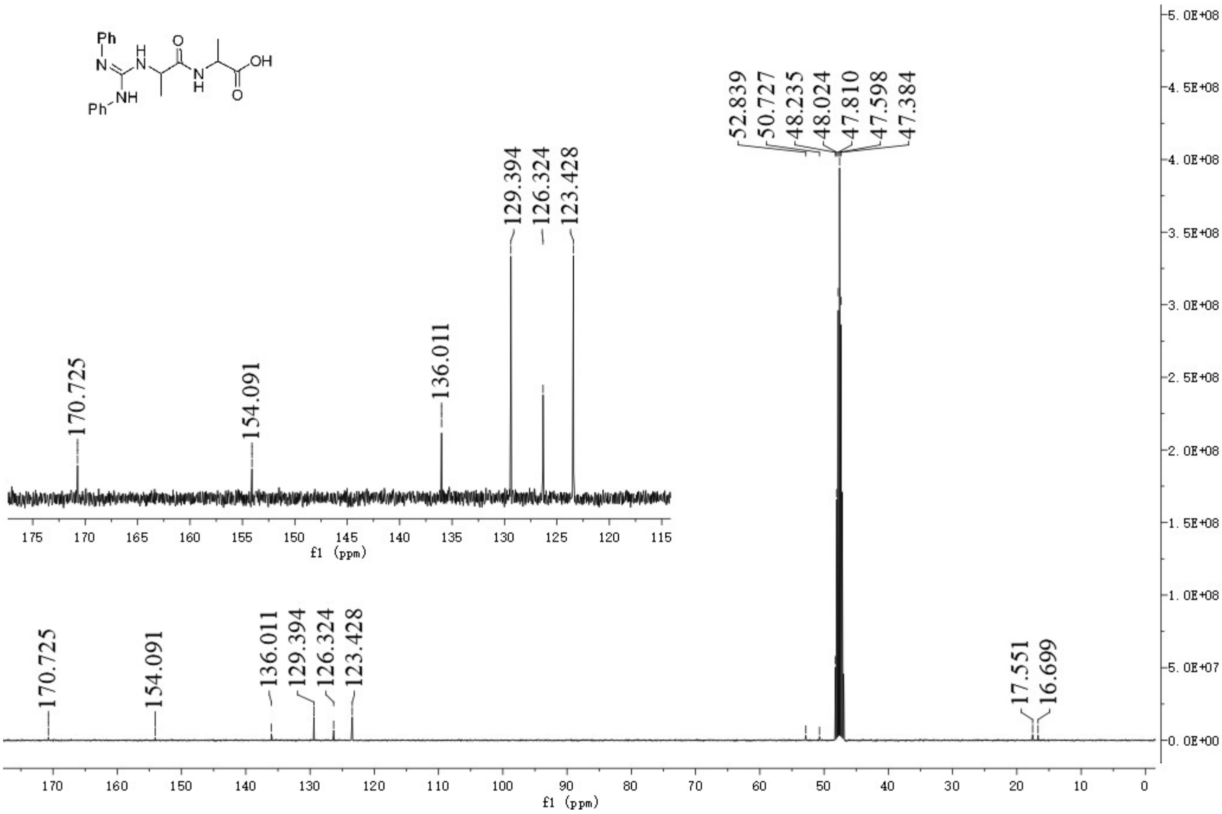

(*N*,*N′*-Diphenylcarbamimidoyl)phenylalanylphenylalanine (76)

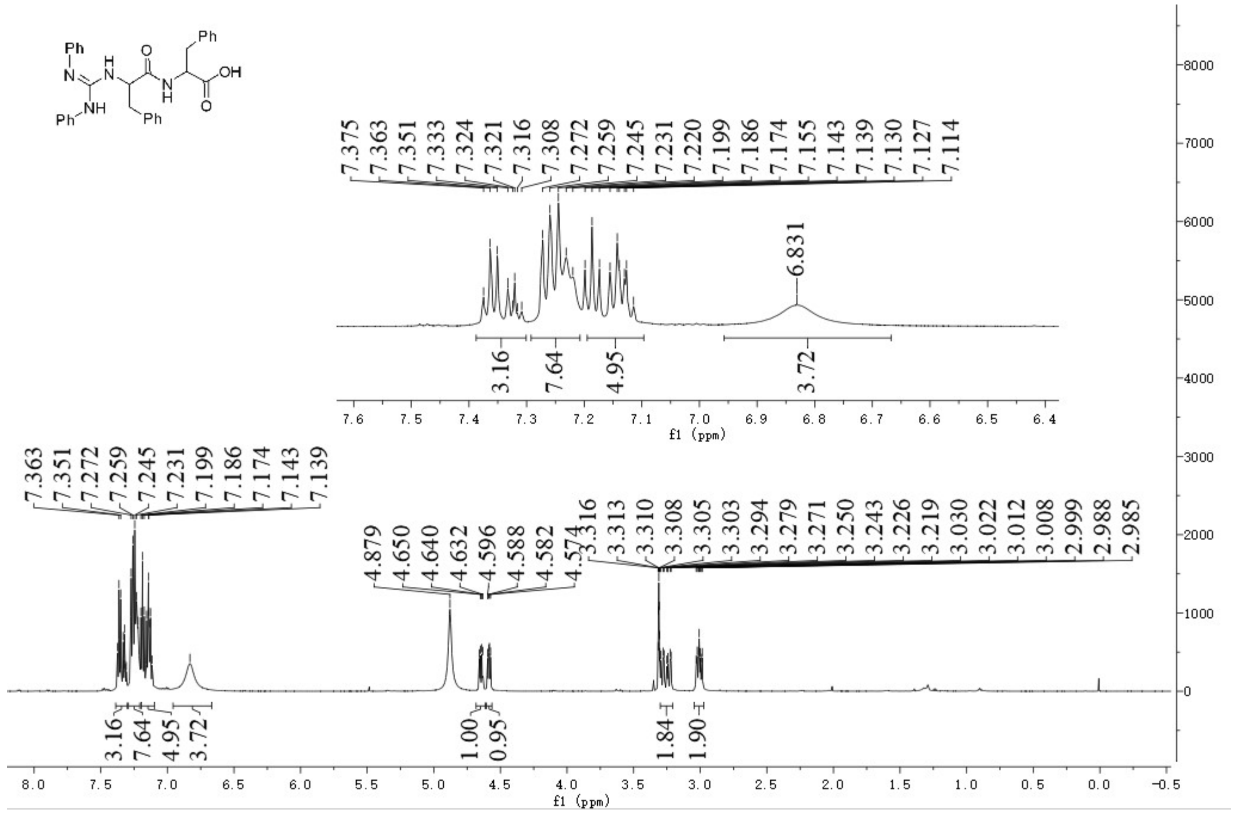

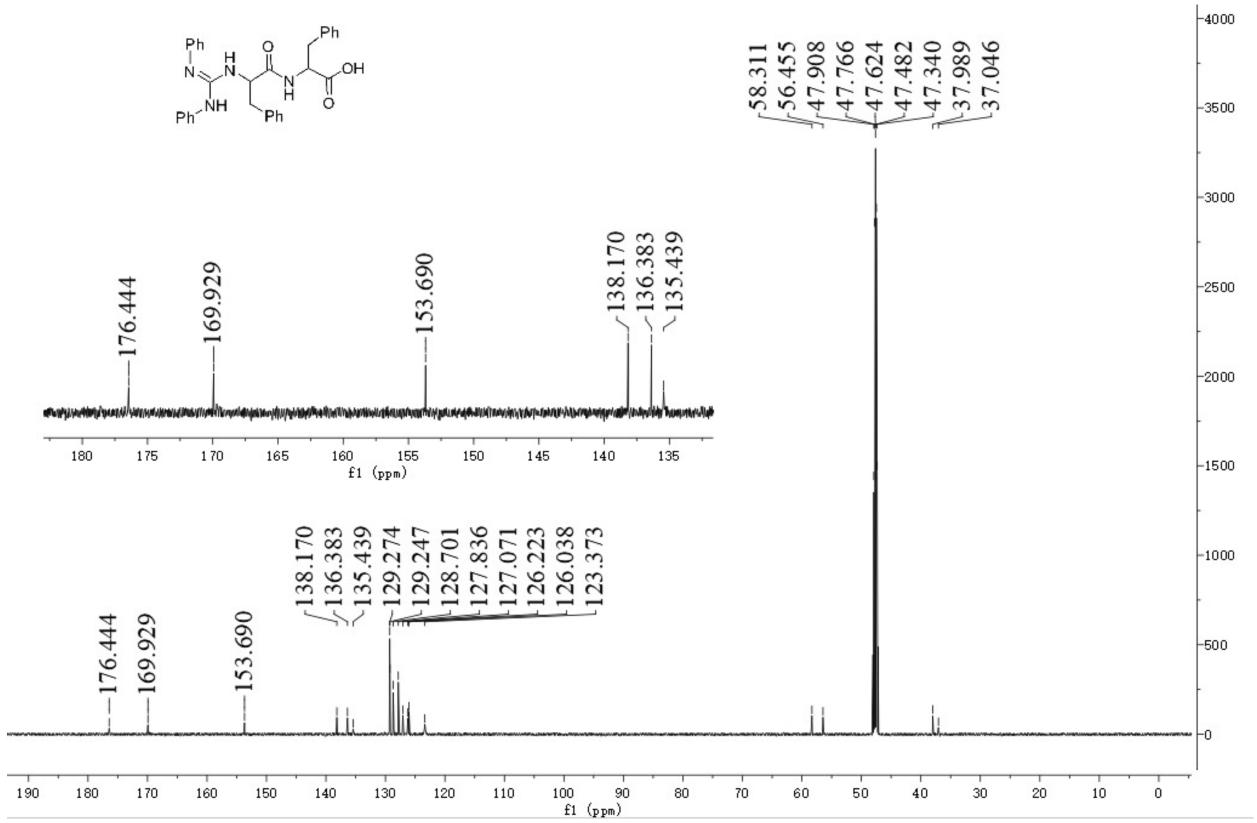

(*N*,*N′*-Diphenylcarbamimidoyl)alanylphenylalanylleucine (77)

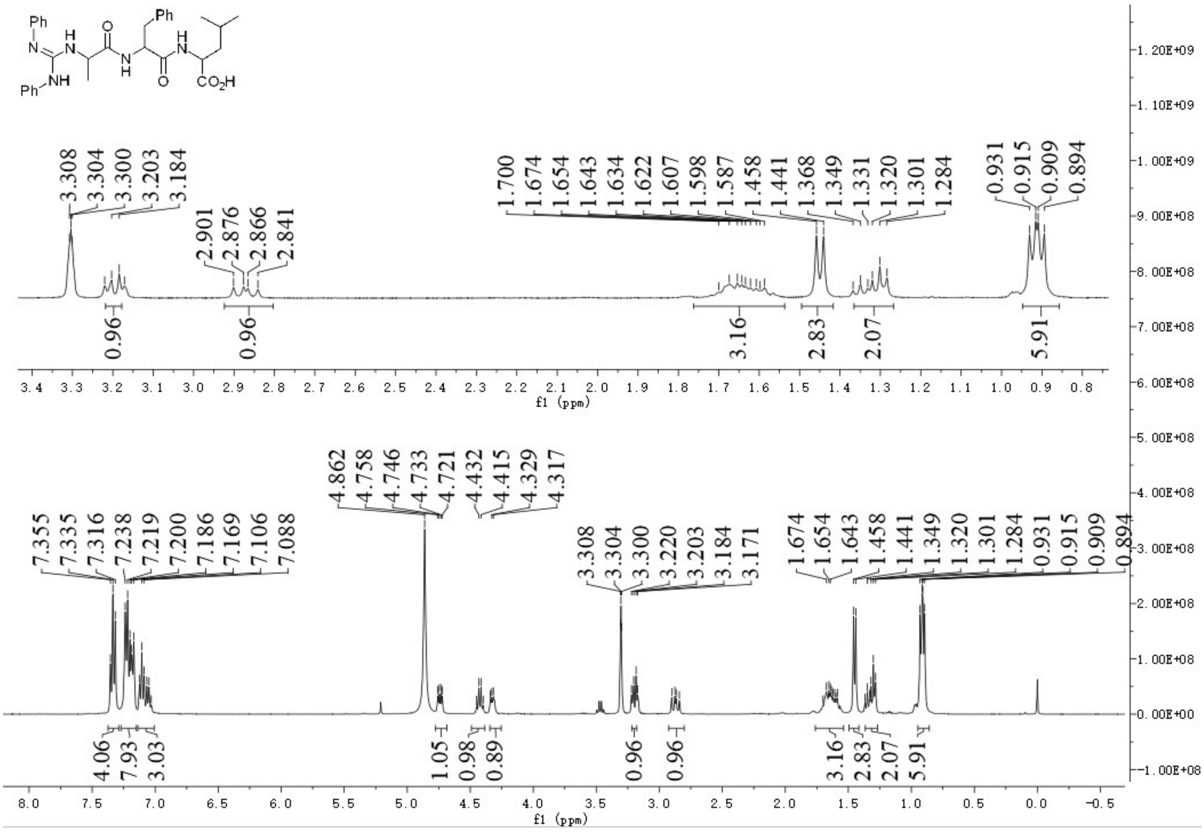

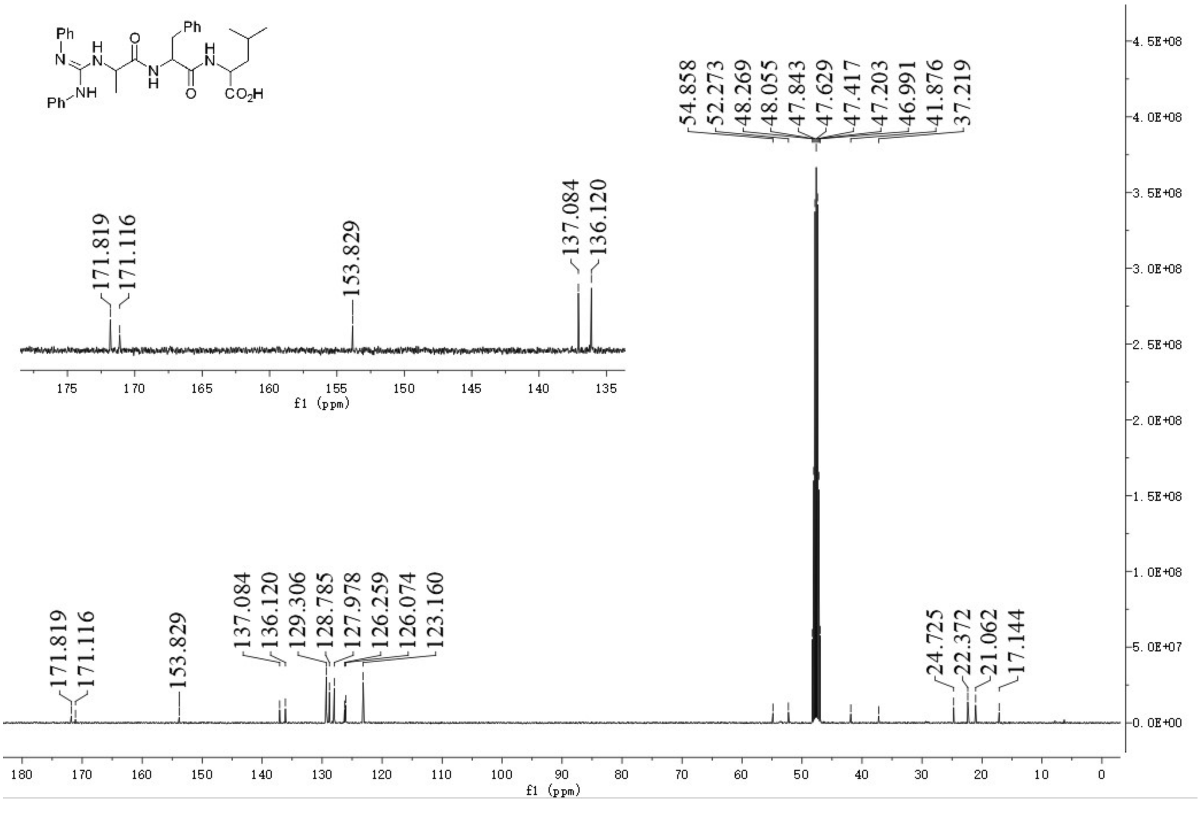

9-Benzyl-15-ethyl-12-(hydroxymethyl)-6-isobutyl-4,7,10,13-tetraoxo-1-(phenylamino)-1-(phenylimino)-2,5,8,11,14-pentaazahexadecan-16-oic acid (78)

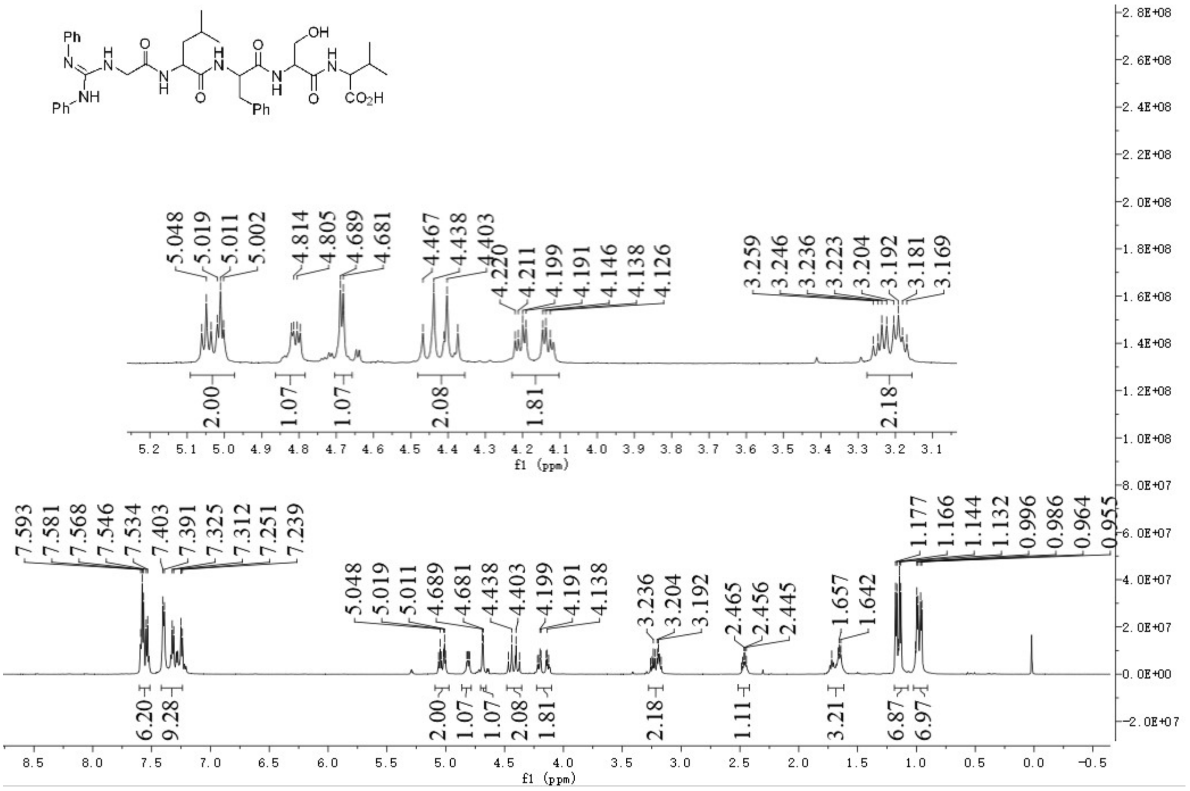

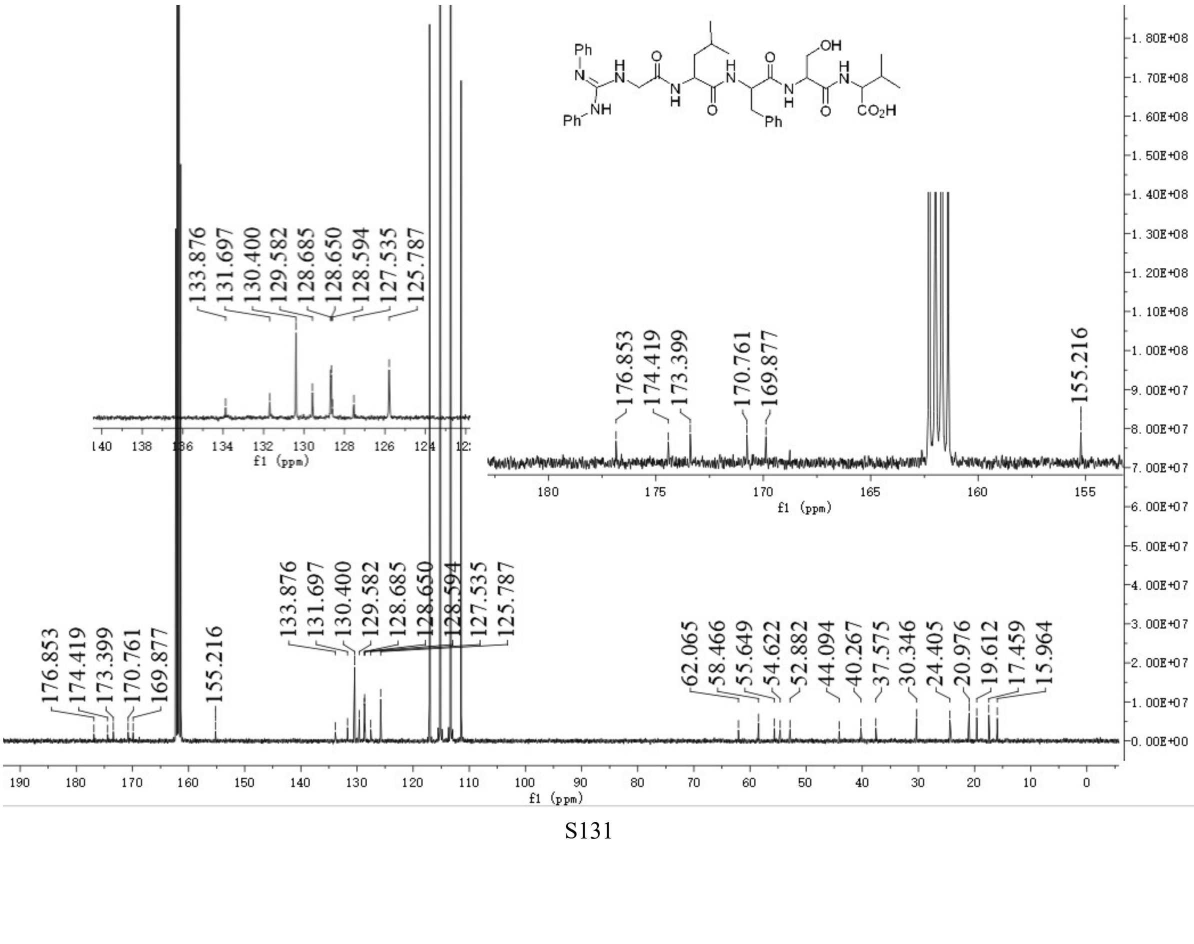

3-(4-Chlorophenyl)-4-(2,3-diphenylguanidino)butanoic acid (79)

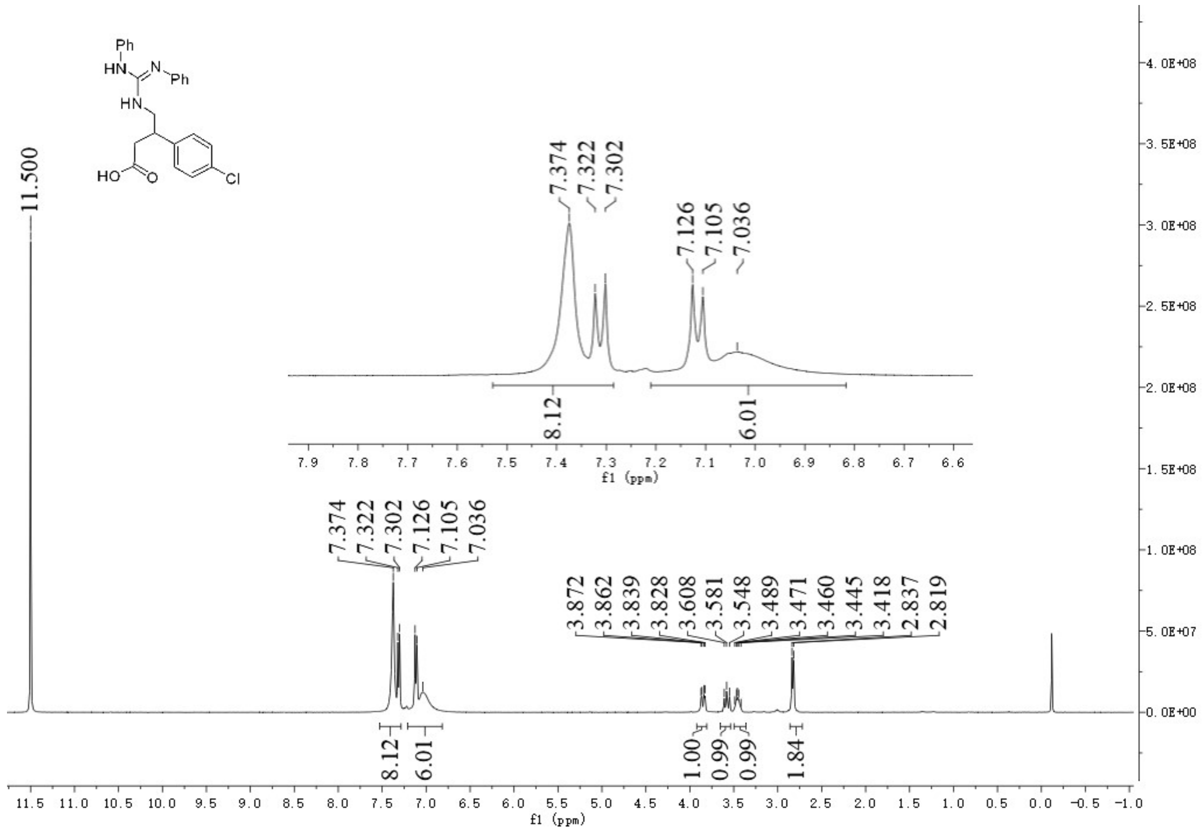

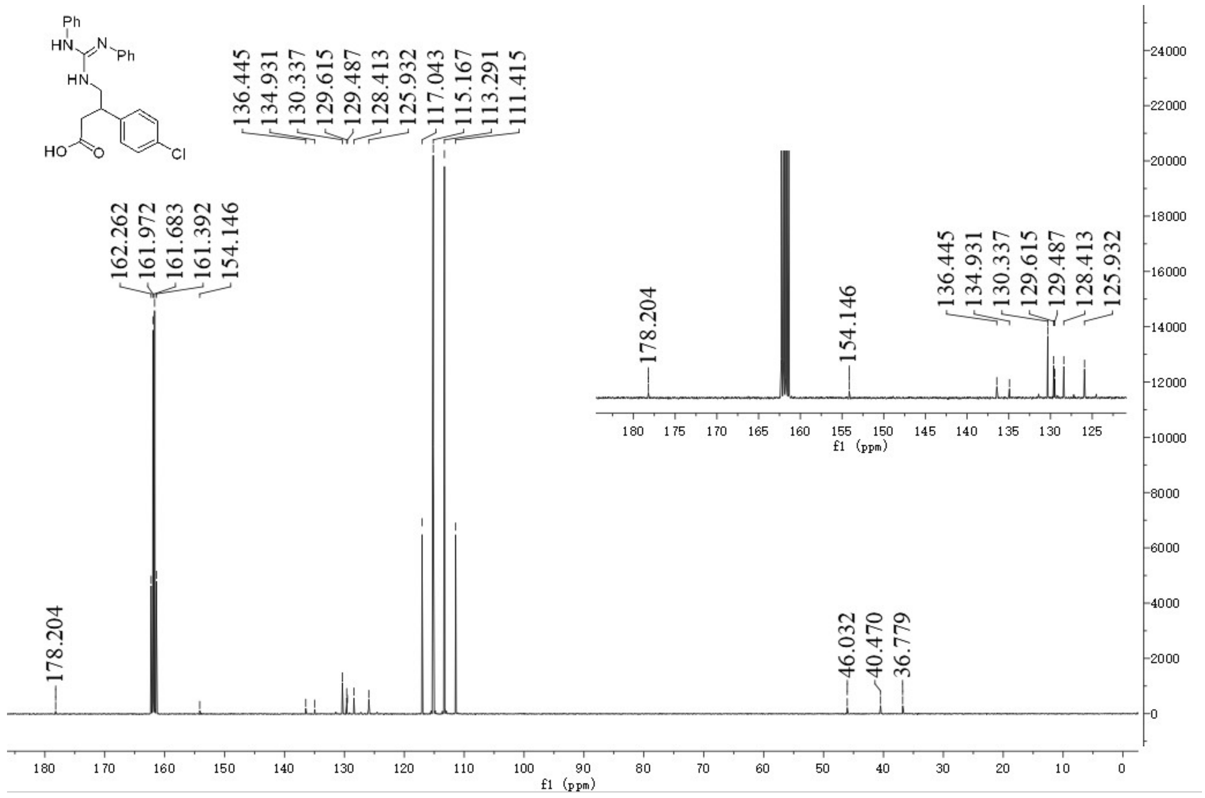

Tert-butyl-2-((4R,6R)-6-(2-(-2,3-diphenylguanidino)ethyl)-2,2-dimethyl-1,3-dioxan-4-yl)acetate (80)

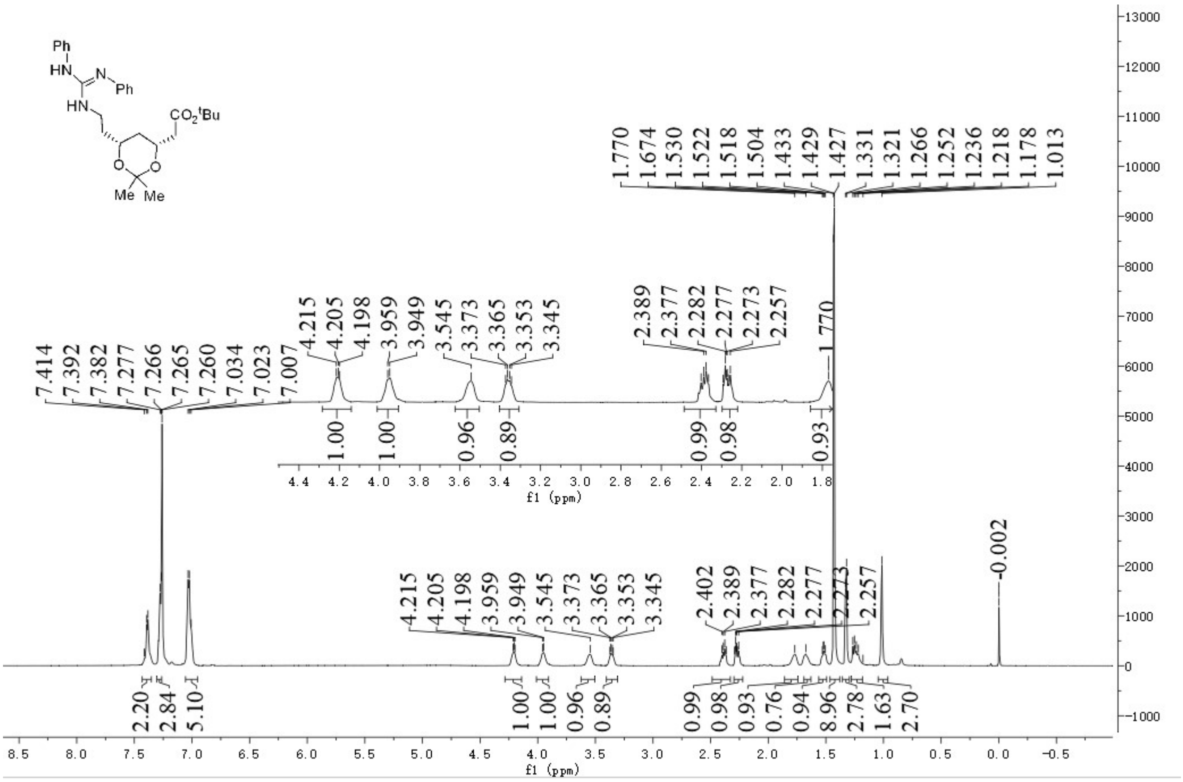

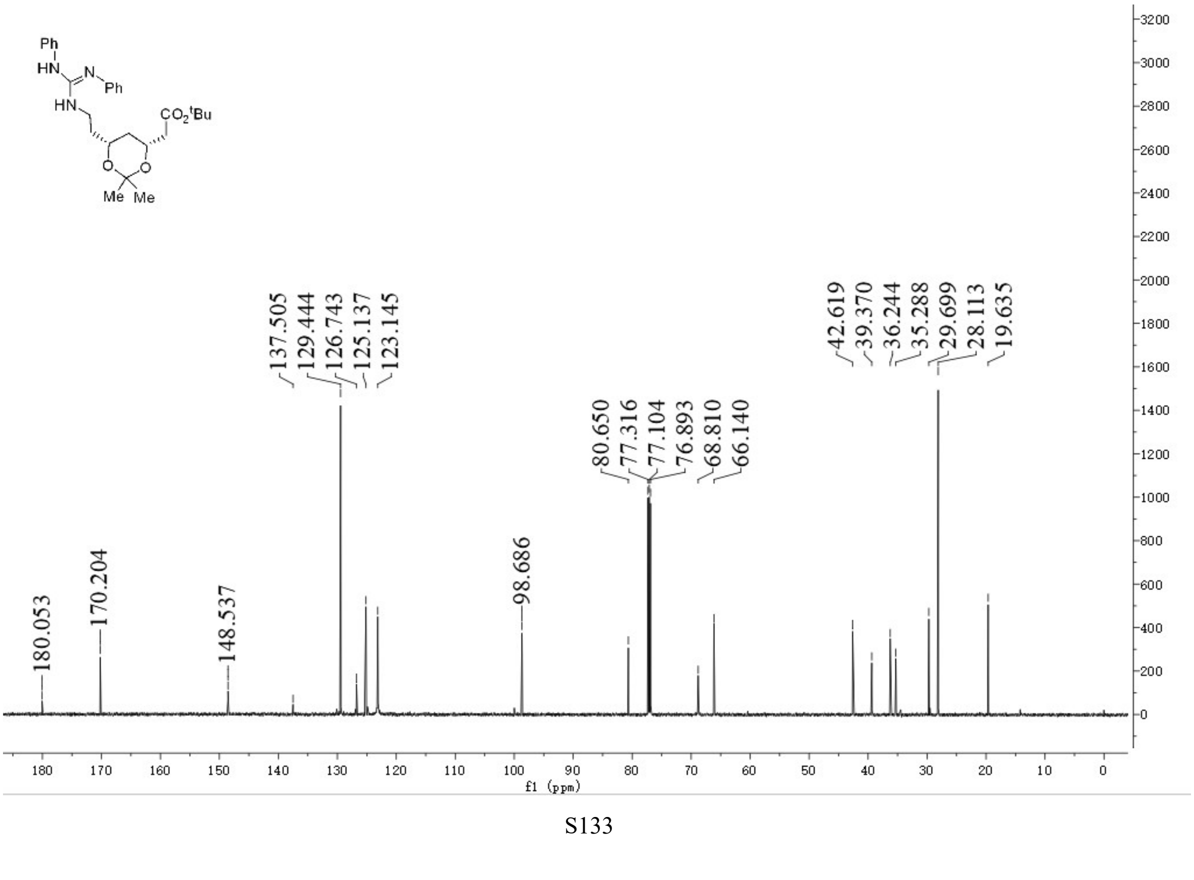

3-Ethyl-5-methyl-4-(2-chlorophenyl)-2-((2-(2,3-diphenylguanidino)ethoxy)methyl)-6-methyl-1,4-dihydropyridine-3,5-dicarboxylate (81)

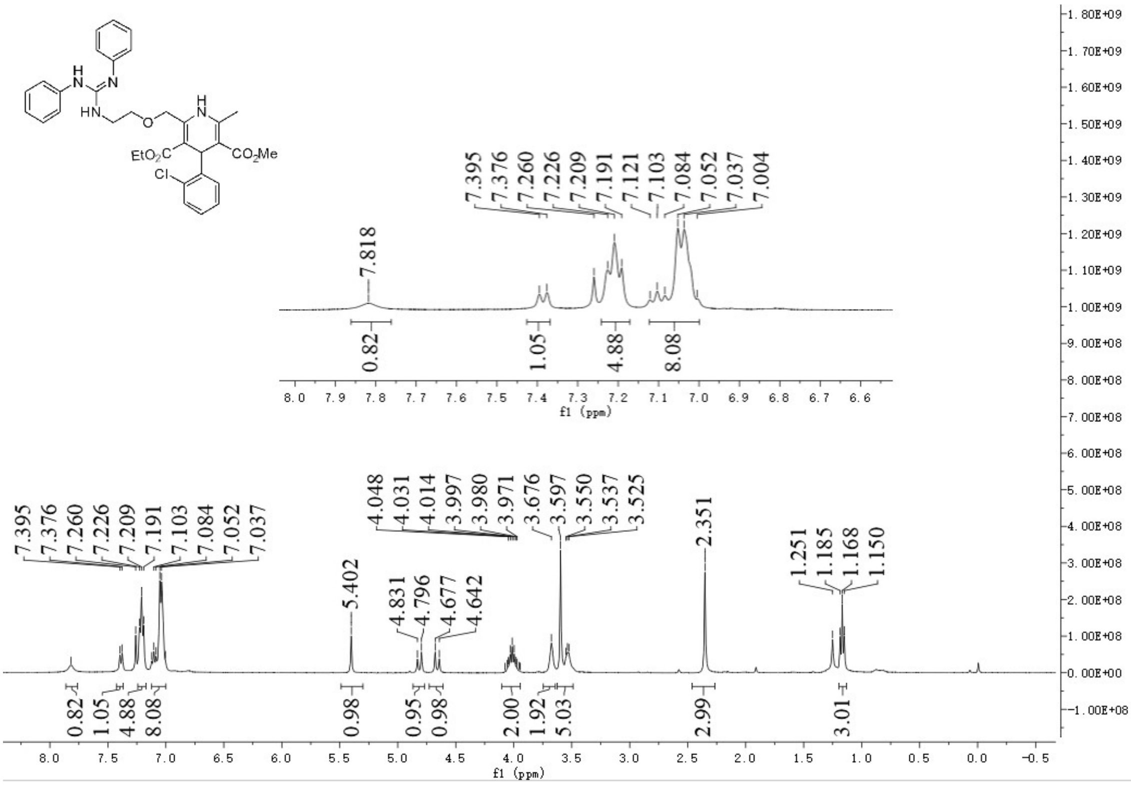

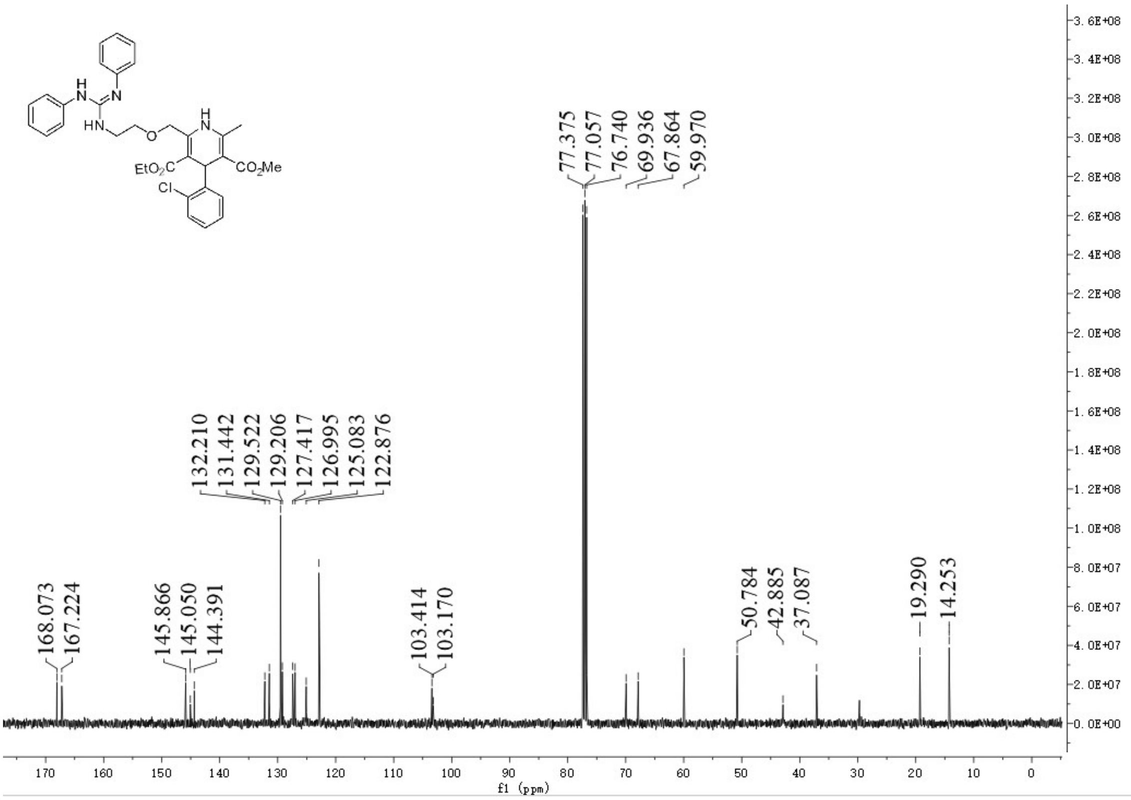

1-(1-(7-(But-2-yn-1-yl)-3-methyl-1-((4-methylquinazolin-2-yl)methyl)-2,6-dioxo-2,3,6,7-tetrahydro-1H-purin-8-yl)piperidin-3-yl)-2,3-diphenylguanidine (82)

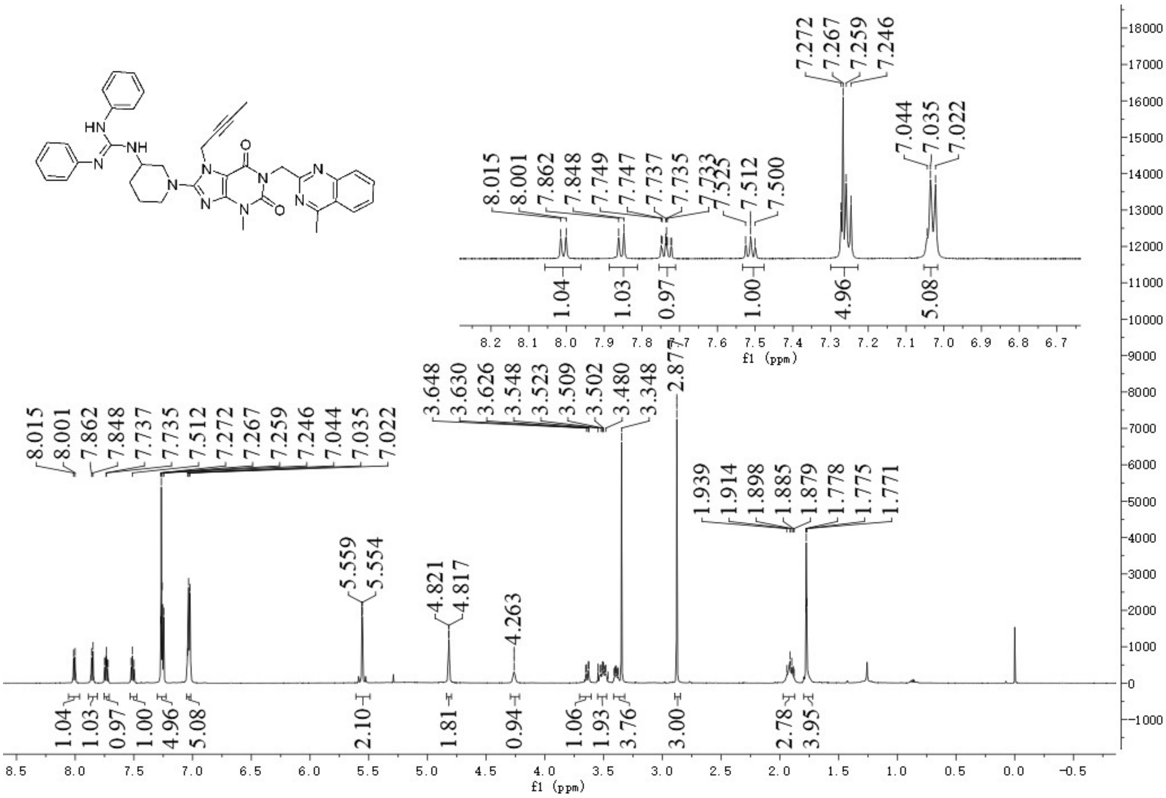

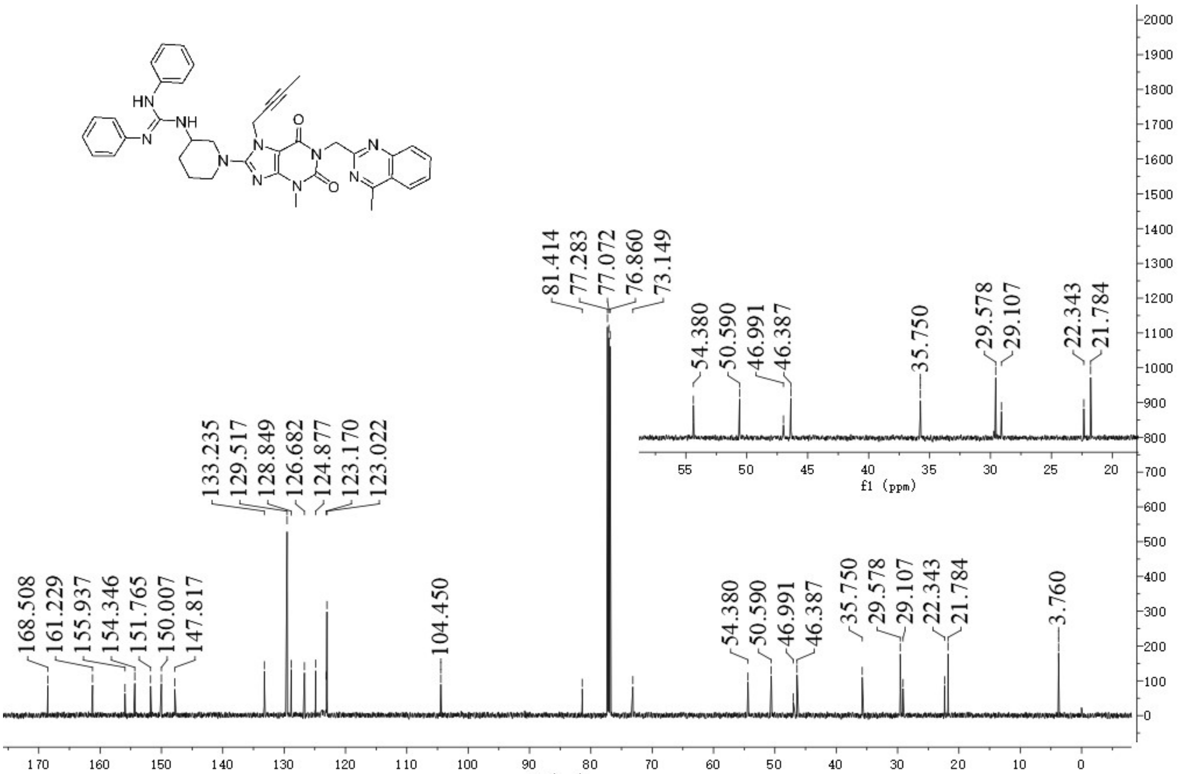

4-(8-Chloro-5,6-dihydro-11*H*-benzo[5,6]cyclohepta[1,2-*b*]pyridin-11-ylidene)-N,N'-diphenylpiperidine-1-carboximidamide (83)

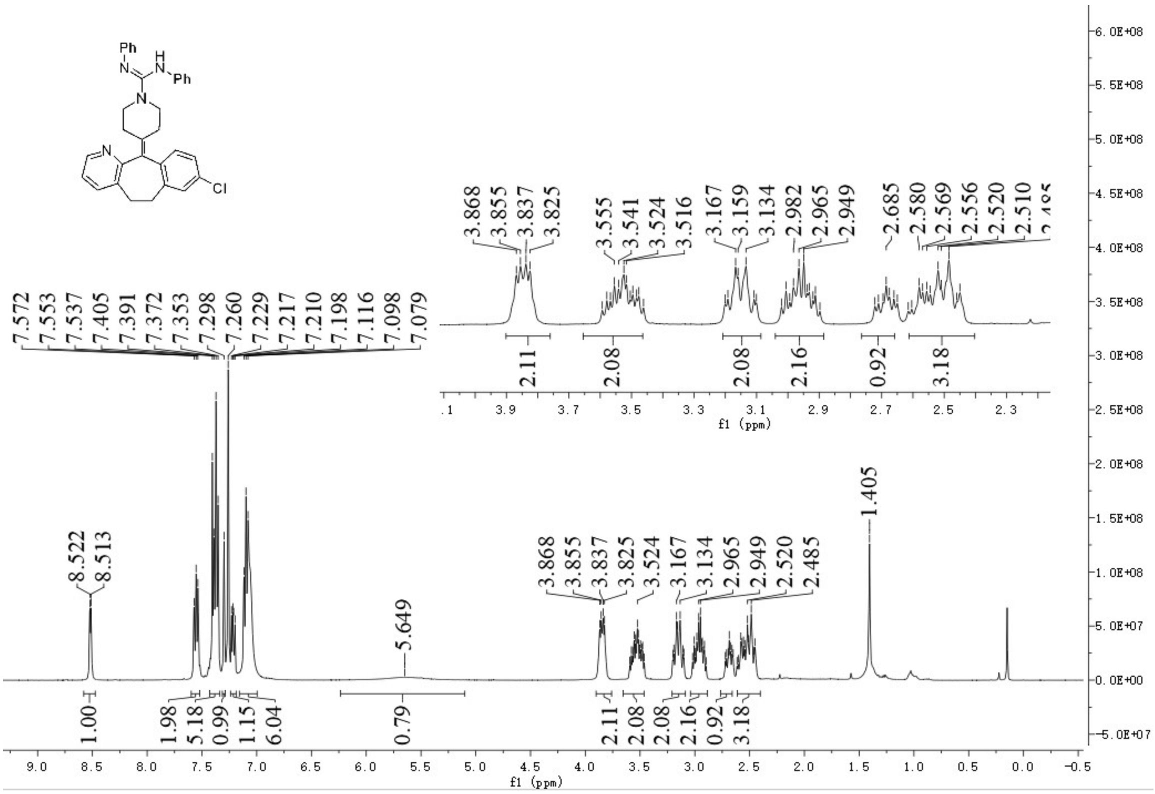

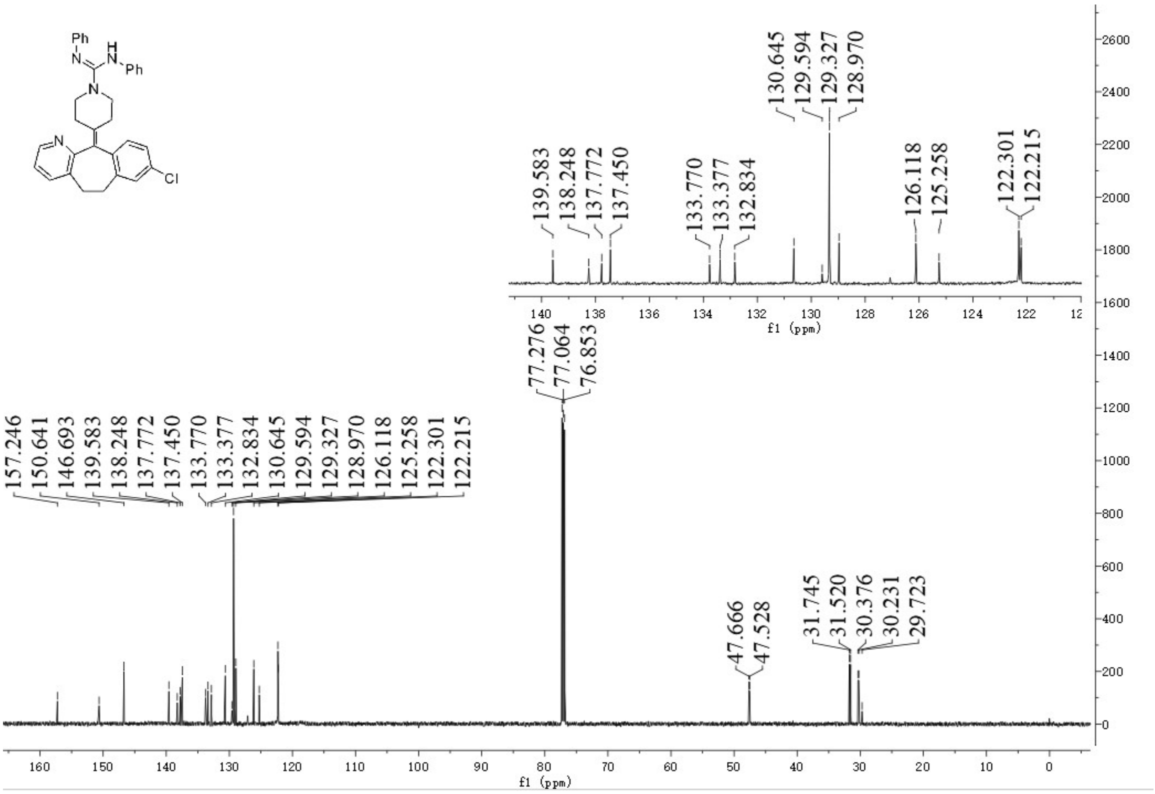

3-Ethyl-5-methyl-4-(2-chlorophenyl)-6-methyl-2-((2-(3-phenyl-2-(pyridin-4-yl)guanidino)ethoxy)methyl)-1,4-dihydropyridine-3,5-dicarboxylate (84)

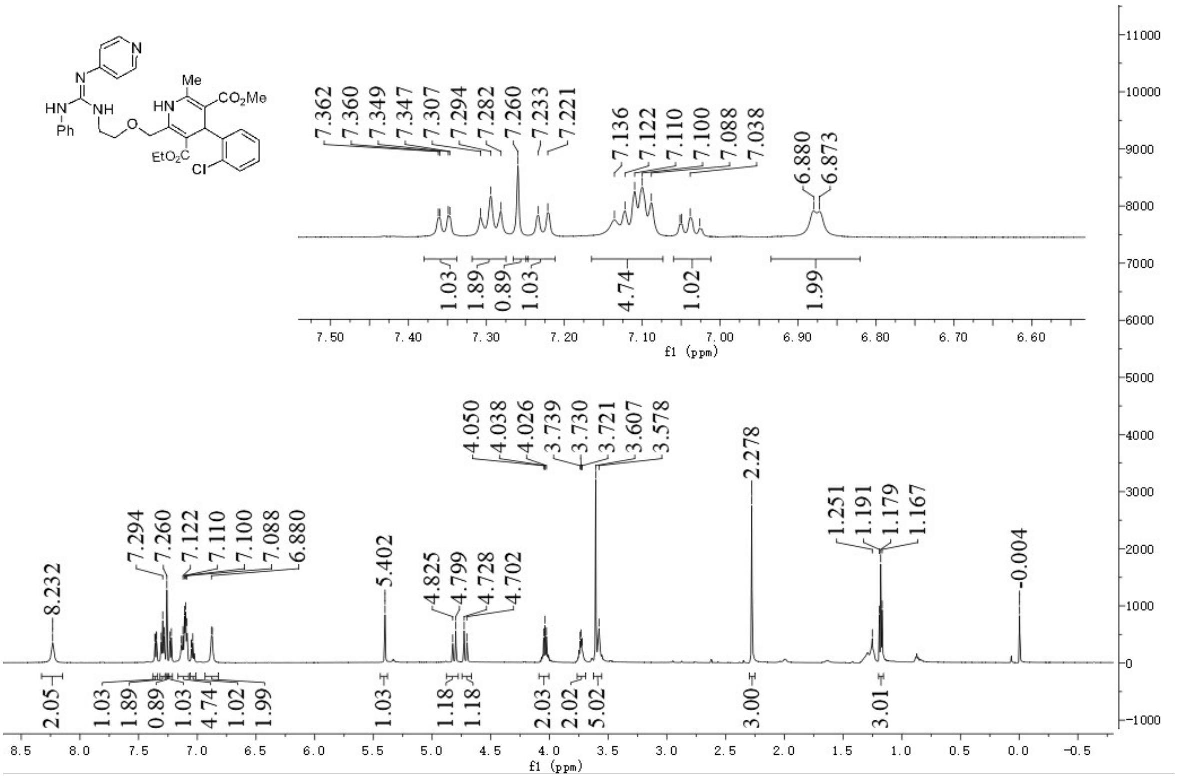

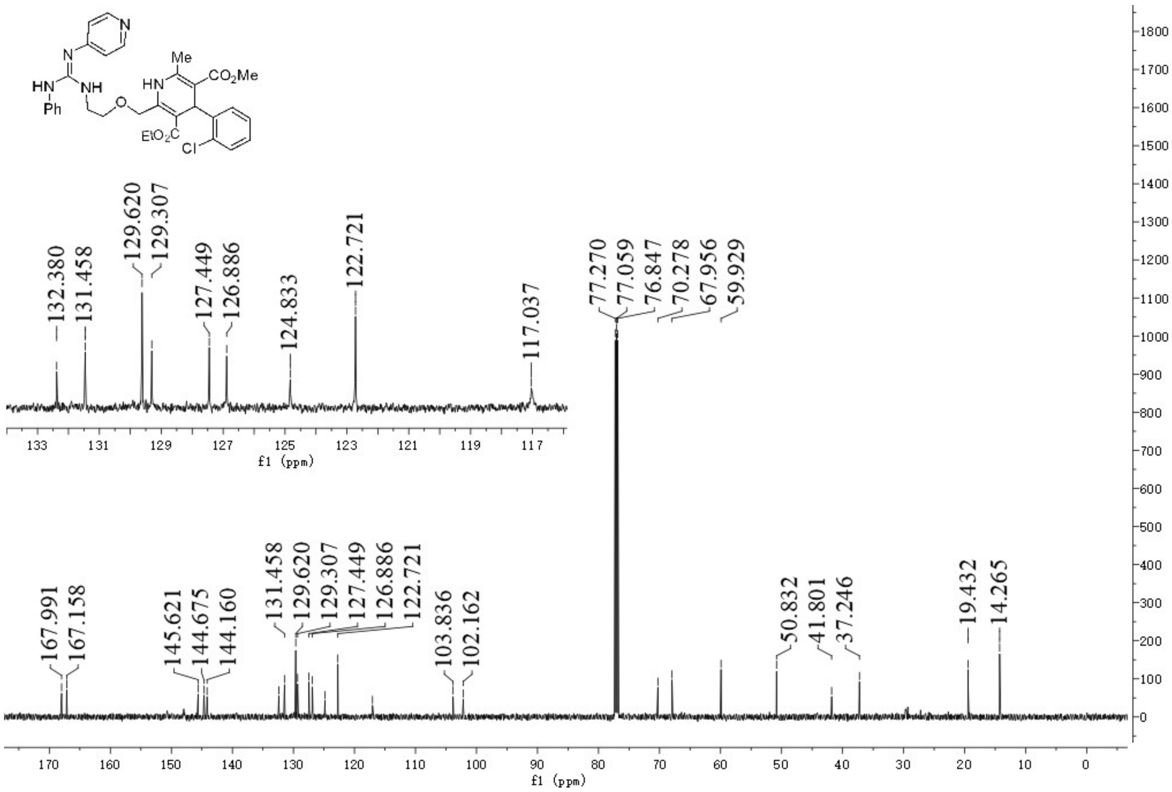

4-(8-Chloro-5,6-dihydro-11*H*-benzo[5,6]cyclohepta[1,2-*b*]pyridin-11-ylidene)-*N*-phenyl-*N'*-(pyridin-4-yl)piperidine-1-carboximidamide (85)

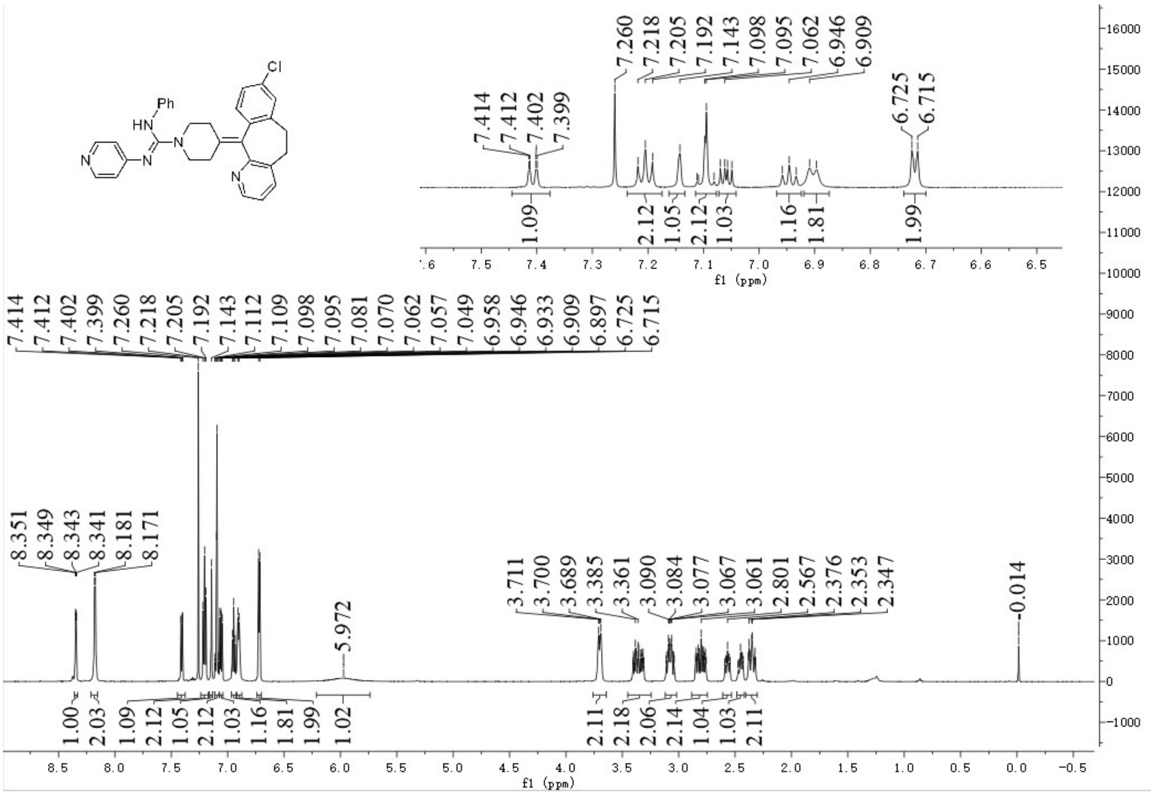

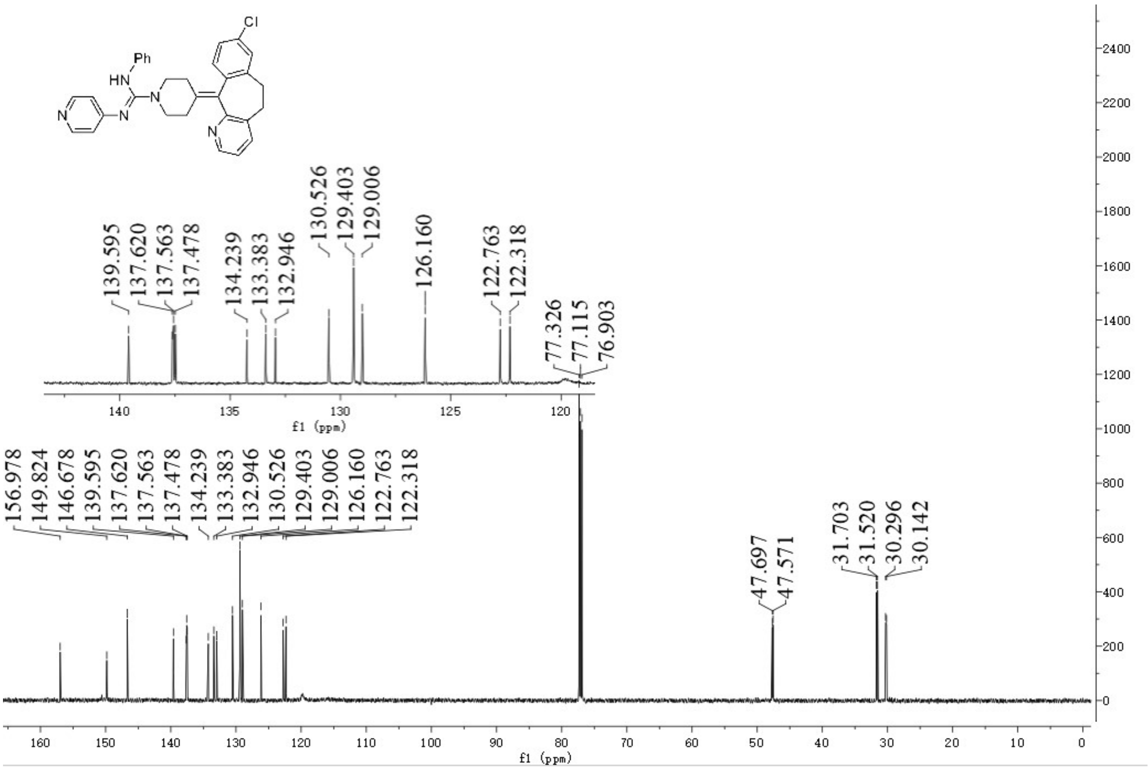

1-(1-(7-(But-2-yn-1-yl)-3-methyl-1-((4-methylquinazolin-2-yl)methyl)-2,6-dioxo-2,3,6,7-tetrahydro-1*H*-purin-8-yl)piperidin-3-yl)-3-phenyl-2-(pyridin-4-yl)guanidine (86)

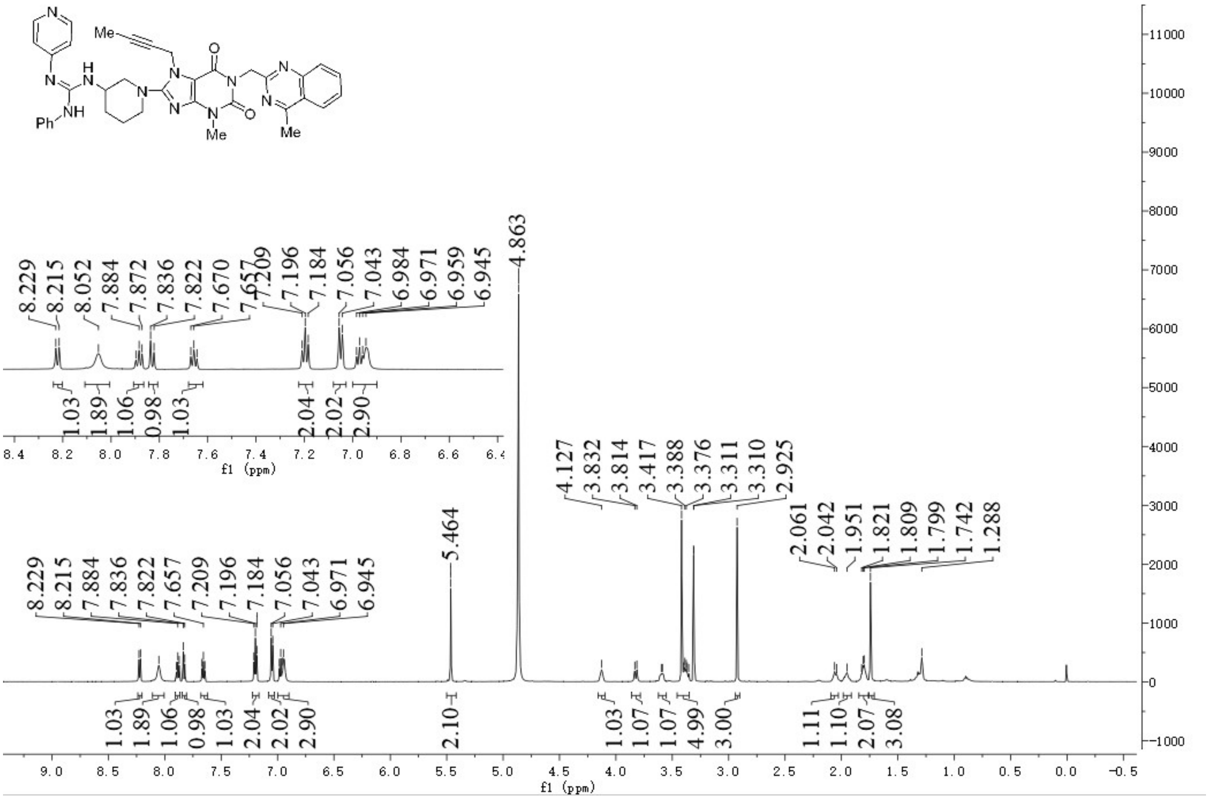

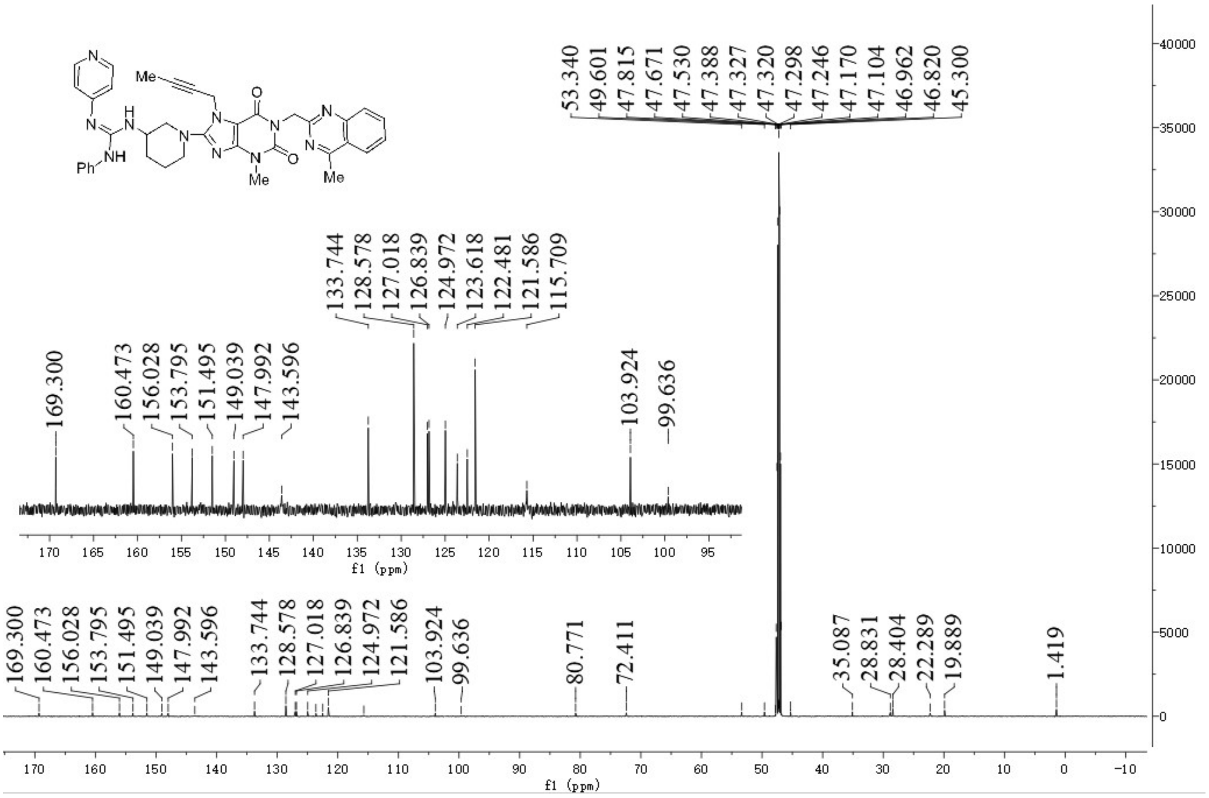

5-((((2-((4-(2-Chlorophenyl)-3-(ethoxycarbonyl)-5-(methoxycarbonyl)-6-methyl-1,4-dihydropyridin-2-yl)methoxy)ethyl)amino)(phenylamino)methylene)amino)-2-hydroxybenzoic acid (87)

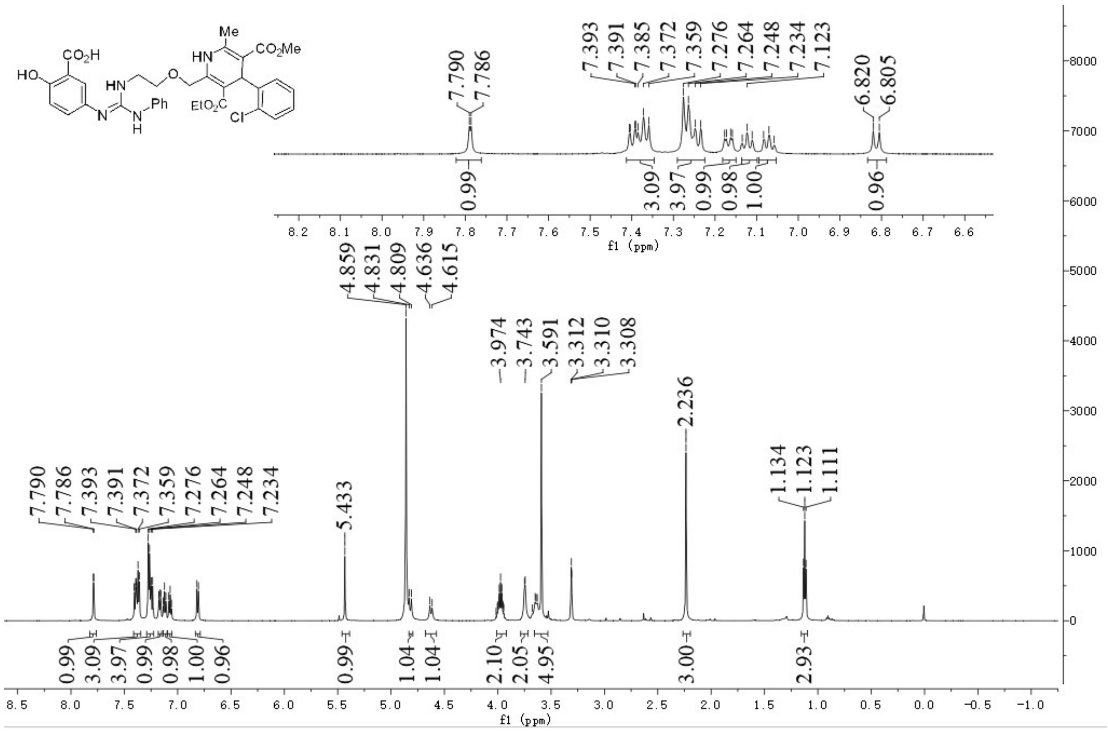

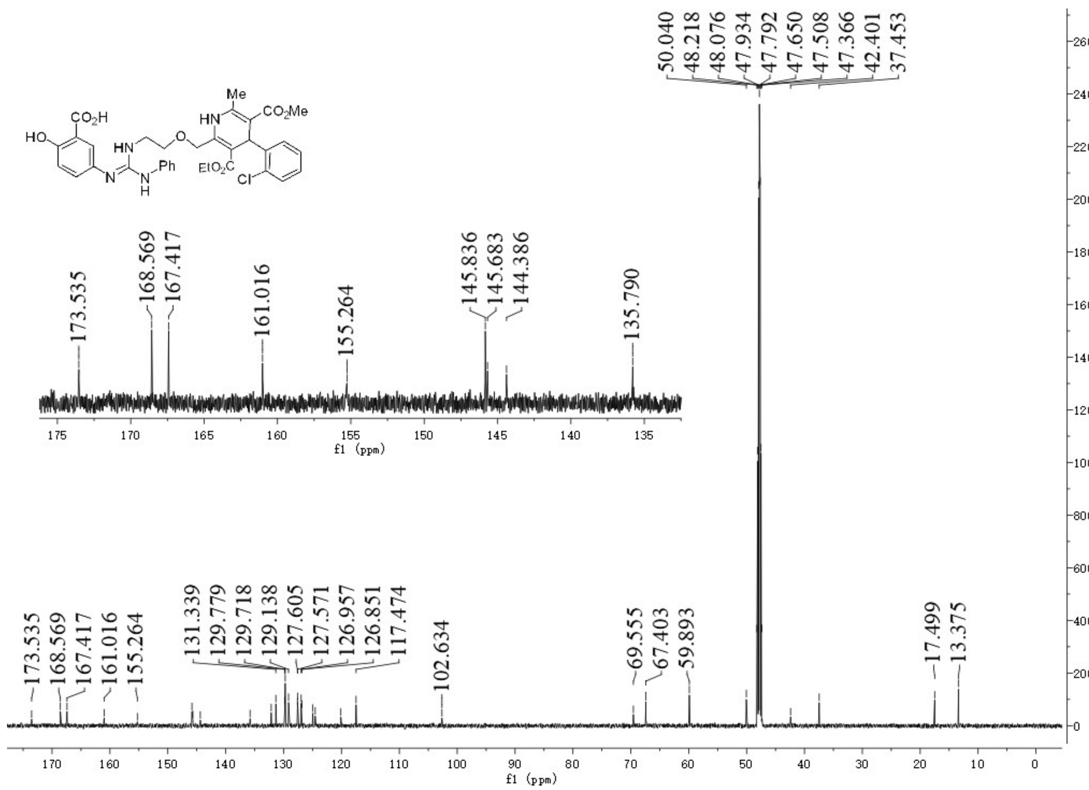

5-(((4-(8-Chloro-5,6-dihydro-11H-benzo[5,6]cyclohepta[1,2-*b*]pyridin-11-ylidene)piperidin-1-yl)(phenylamino)methylene)amino)-2-hydroxybenzoic acid (88)

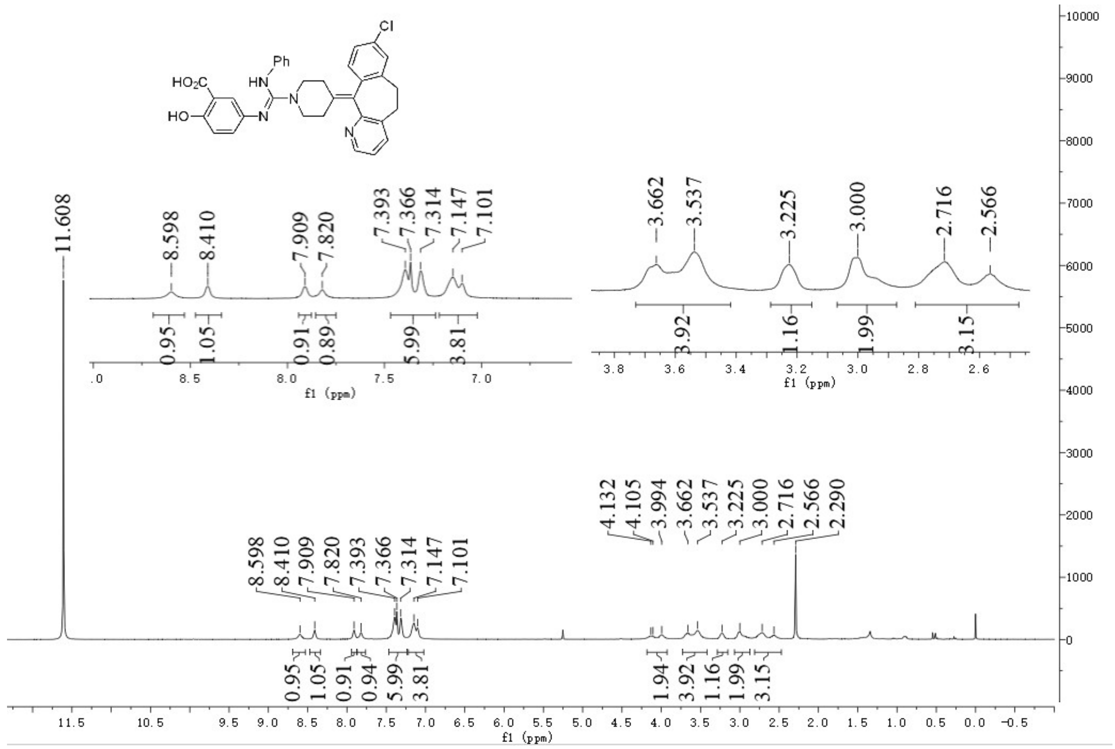

(*N*-Benzhydryl-*N'*-(4-cyanophenyl)carbamimidoyl)glycine (NC-174) (89)

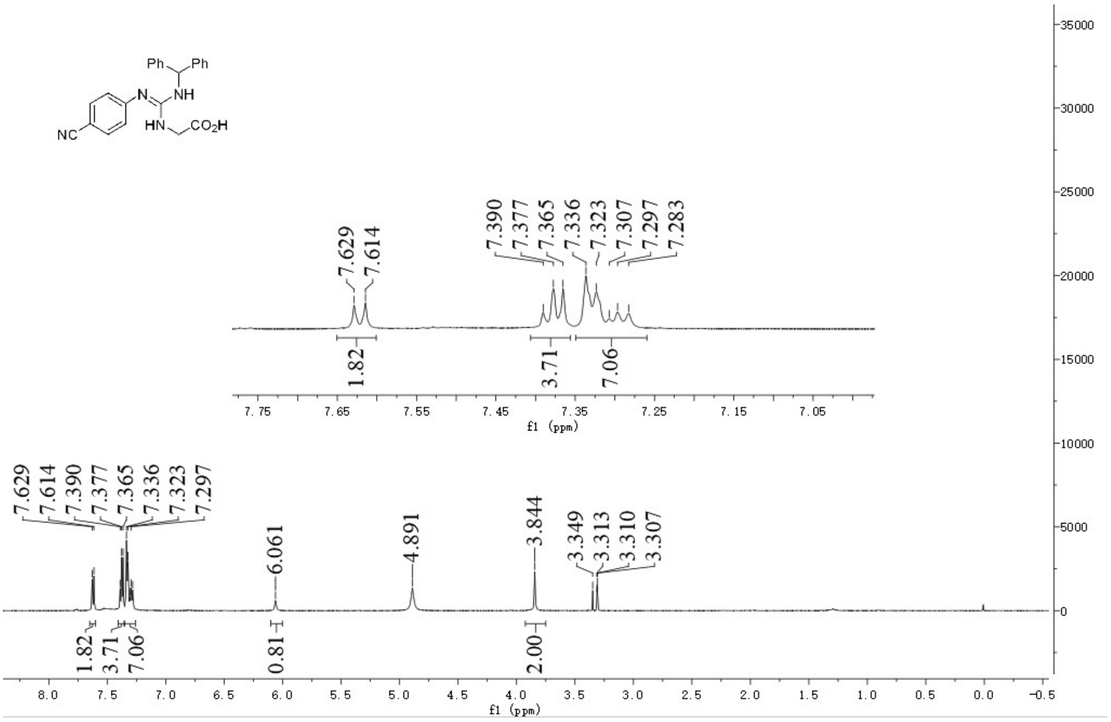

Pinacidil (90)

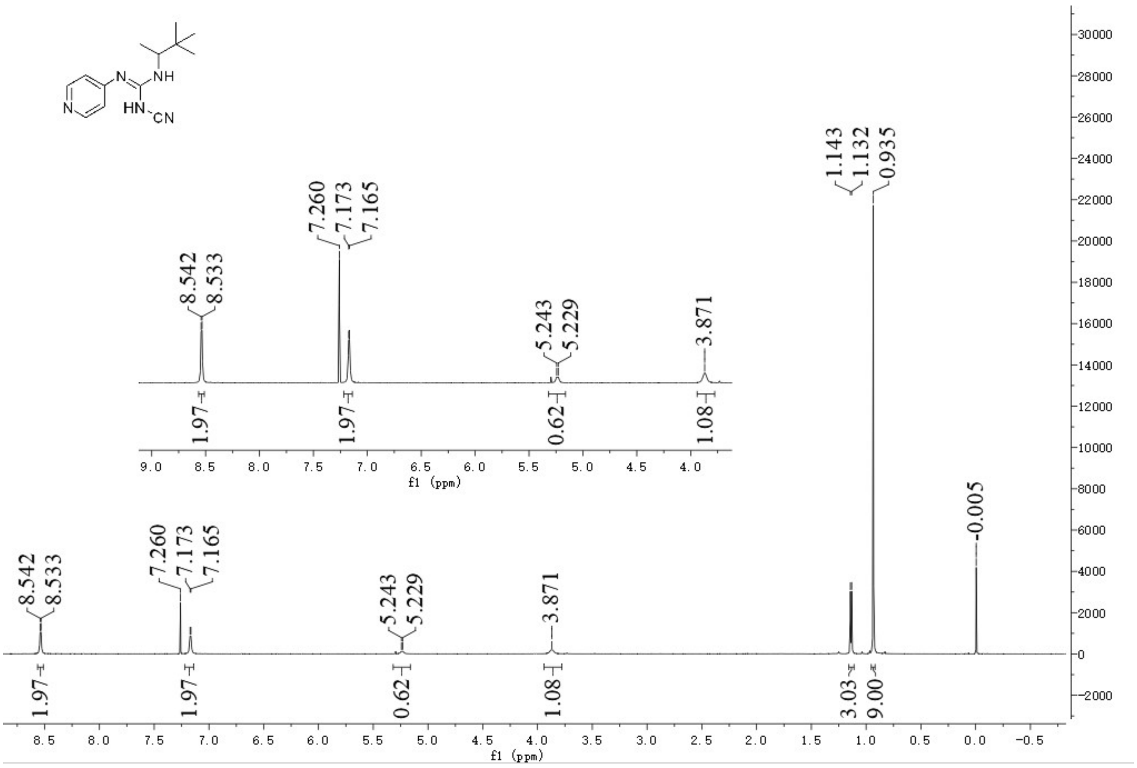

1,3-Diphenylurea (U1)

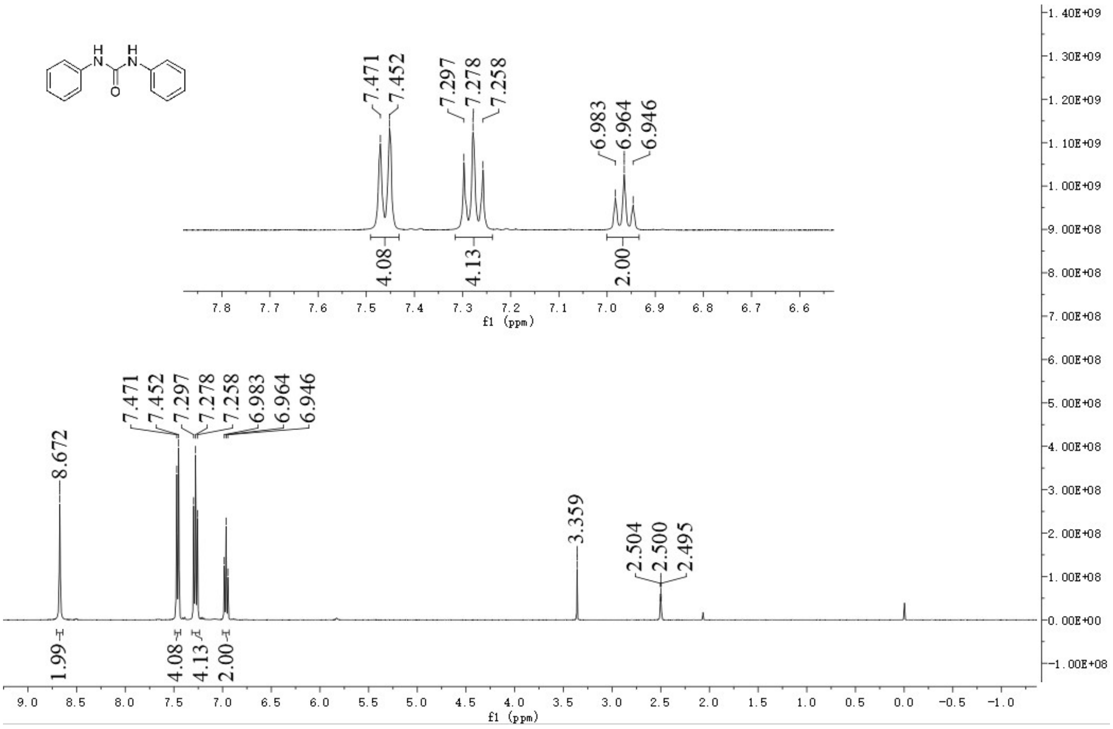
7.471
7.452
7.297
7.278
7.258
6.983
6.964
6.946
4.08
4.13
2.00
f1 (ppm)
8.672
3.359
2.504
2.500
2.495
1.99
4.08
4.13
2.00
f1 (ppm)
